# Supplementary material for: Reticulophagy receptor FAM134C restrains BMP receptor signaling
Source: EMBO J. 2025 Oct 20;44(23):7154–80. doi: 10.1038/s44318-025-00581-3 (PMC12669696; doi:10.1038/s44318-025-00581-3)
Supplement: Supplementary file 2 — Movie EV1 [file 44318_2025_581_MOESM2_ESM.zip › Movie EV1 Legends.docx]

**Movie Legends**

**Movie EV1. FAM134C directs membrane-bound BMPR1A for degradation.**

The live cell imaging experiment utilized FAM134C-KO U2OS cells stably expressing FAM134C-GFP and BMPR1A-mCherry. Lysotracker was employed to stain lysosomes, exhibiting blue fluorescence. Cells were analyzed using a Zeiss LSM880 confocal microscope (scale bar, 10 µm).
