# Supplementary material for: Reticulophagy receptor FAM134C restrains BMP receptor signaling
Source: EMBO J. 2025 Oct 20;44(23):7154–80. doi: 10.1038/s44318-025-00581-3 (PMC12669696; doi:10.1038/s44318-025-00581-3)
Supplement: Supplementary file 3 — Movie EV2 [file 44318_2025_581_MOESM3_ESM.zip › Movie EV2 Legends.docx]

**Movie Legends**

**Movie EV2. FAM134C targets BMPR1A into autophagosomes through LC3.**

Live cell imaging utilized FAM134C-KO U2OS cells that stably express FAM134C-GFP and BMPR1A-mCherry. Lysotracker, which exhibits blue fluorescence, was used to stain the lysosomes. Cells were analyzed with a Zeiss LSM880 confocal microscope (scale bar, 1 µm).
