# Supplementary figures and images for: Reticulophagy receptor FAM134C restrains BMP receptor signaling (part 1 of 3)
Source: EMBO J. 2025 Oct 20;44(23):7154–80. doi: 10.1038/s44318-025-00581-3 (PMC12669696; doi:10.1038/s44318-025-00581-3)

BMPR1a

75kD—  
50kD—

mTOR

250kD—

p-S1/5/8

75kD—  
50kD—

Smad1

75kD—  
50kD—

GAPDH

50kD—  
36kD—

p-mTOR

250kD—

Id-1

20kD—  
15kD—

p-S6K

75kD—  
50kD—

S6K

75kD—  
50kD—

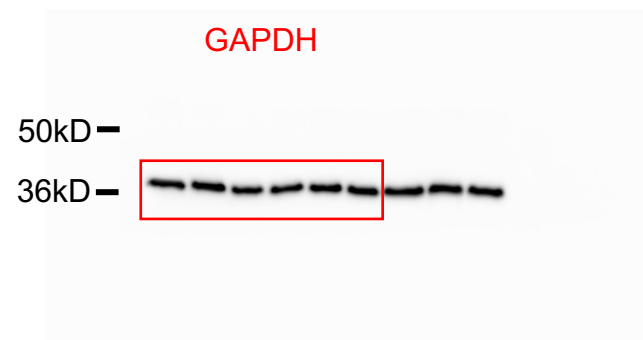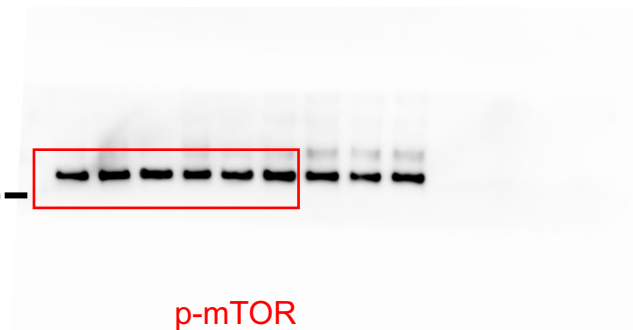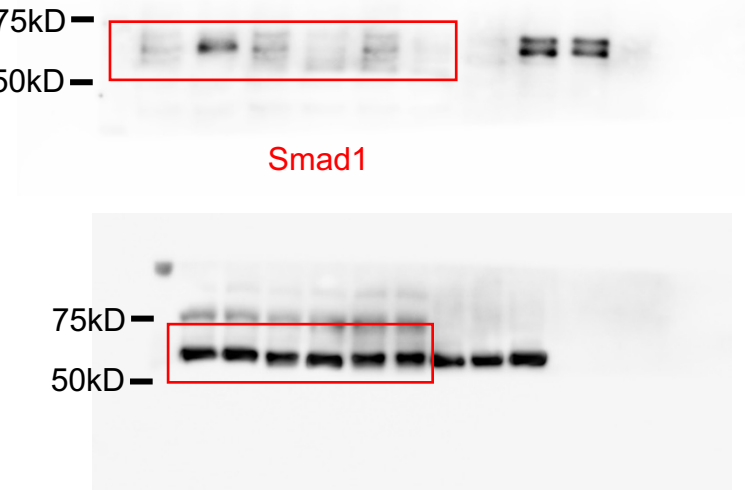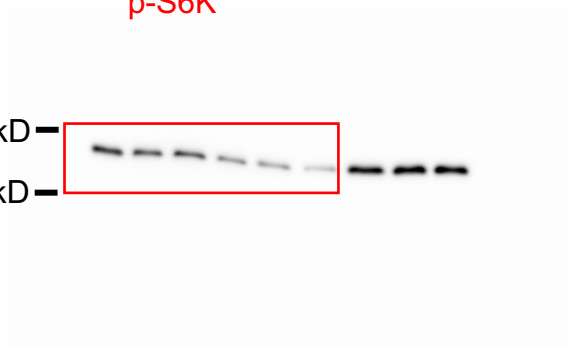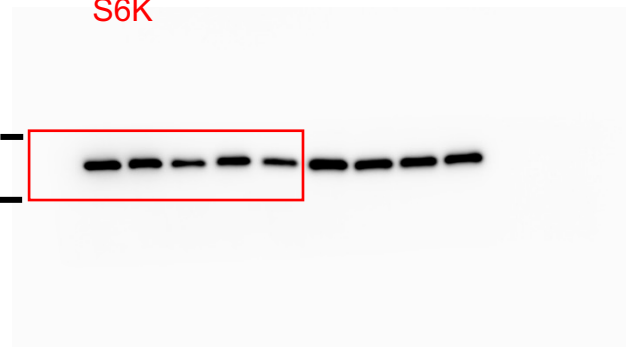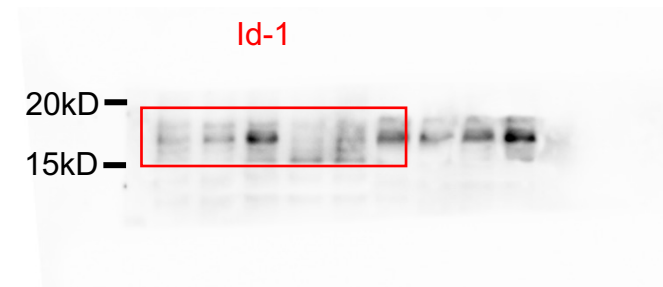

Supplement: Supplementary file 4 — Source data Fig. 1 [file 44318_2025_581_MOESM4_ESM.zip › Fig1/1C.pdf]

BMPR1a

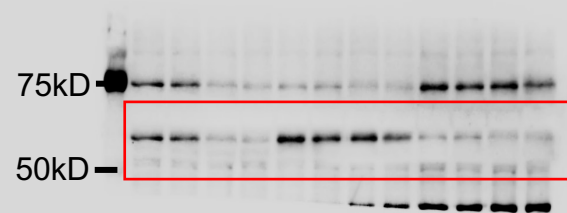

GAPDH

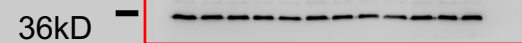

p-S1/5/8

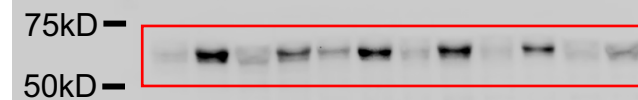

Flag

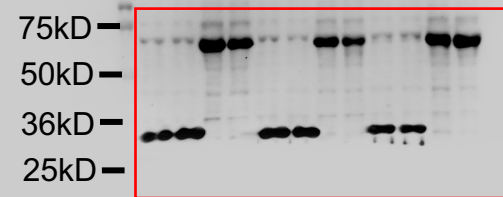

LC3B

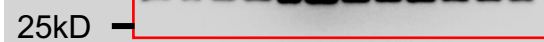

Smad1

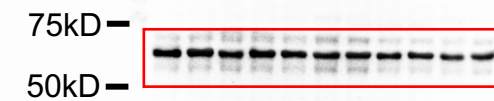

Supplement: Supplementary file 4 — Source data Fig. 1 [file 44318_2025_581_MOESM4_ESM.zip › Fig1/1F.pdf]

BMPR1a

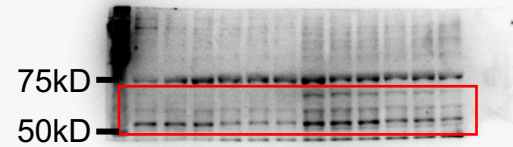

FAM134C

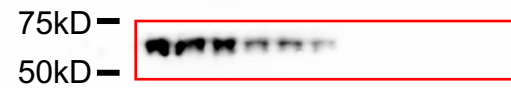

p-S1/5/8

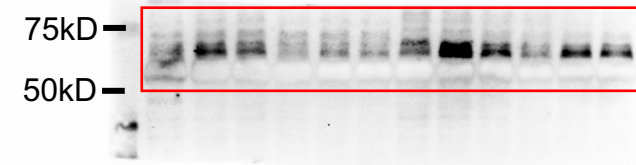

Smad1

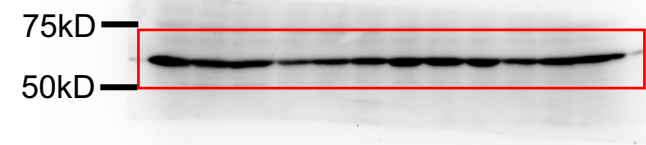

GAPDH

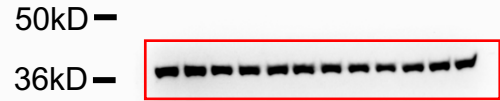

ID-1

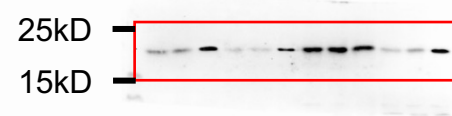

LC3

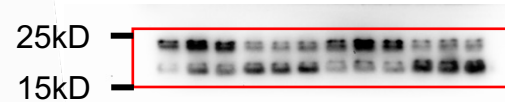

SQSTM1

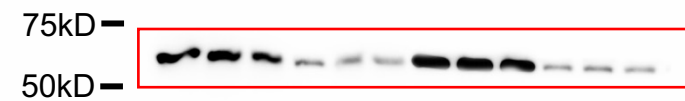

Supplement: Supplementary file 4 — Source data Fig. 1 [file 44318_2025_581_MOESM4_ESM.zip › Fig1/1G.pdf]

Flag

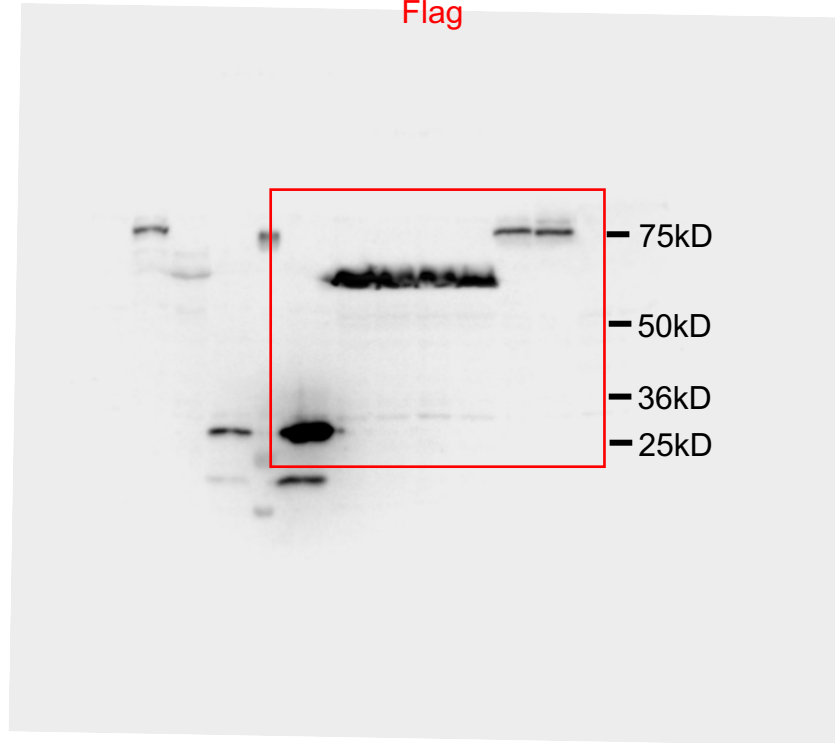

HA

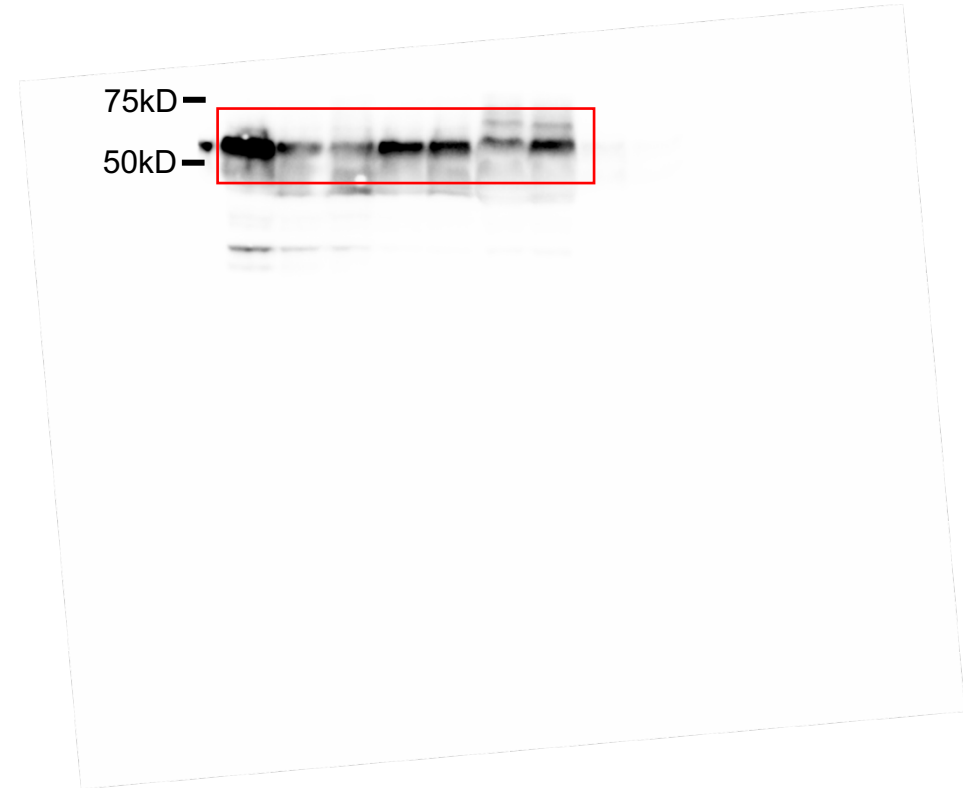

Supplement: Supplementary file 4 — Source data Fig. 1 [file 44318_2025_581_MOESM4_ESM.zip › Fig1/1E.pdf]

BMPR1a

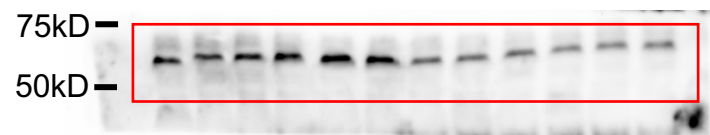

mTOR

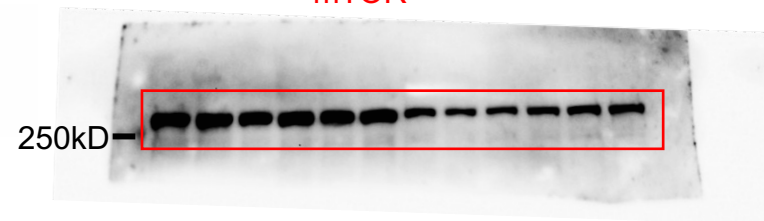

p-S1/5/8

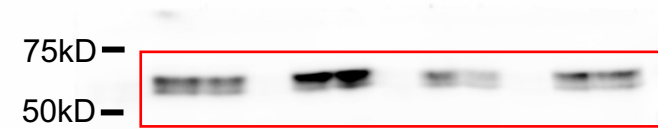

GAPDH

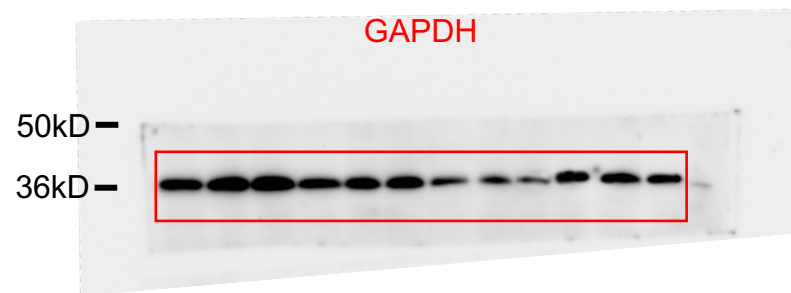

p-mTOR

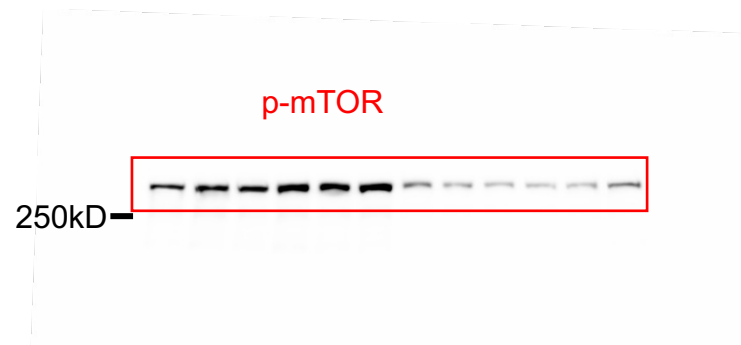

Smad1

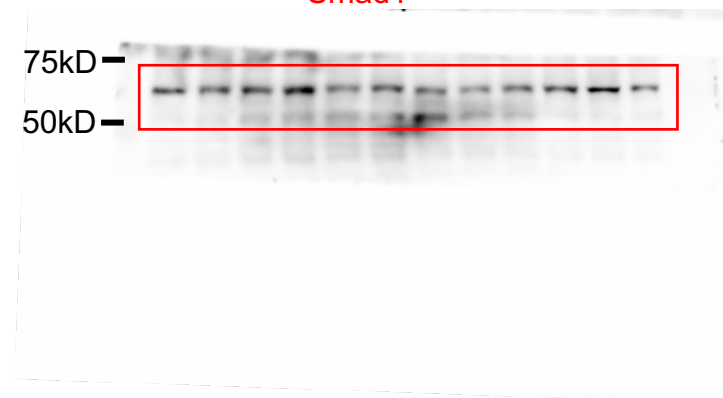

p-S6K

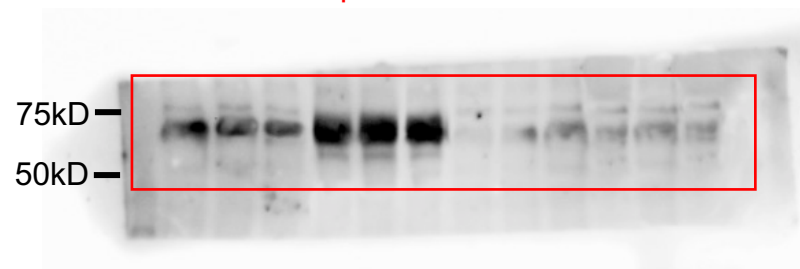

S6K

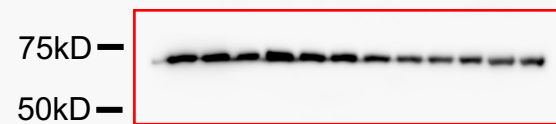

Supplement: Supplementary file 4 — Source data Fig. 1 [file 44318_2025_581_MOESM4_ESM.zip › Fig1/1D.pdf]

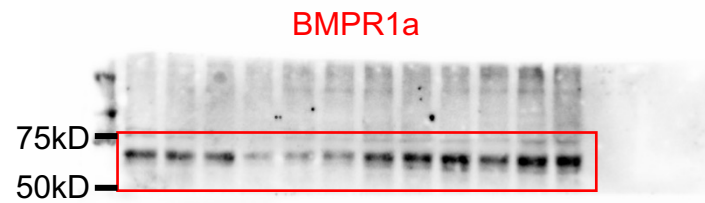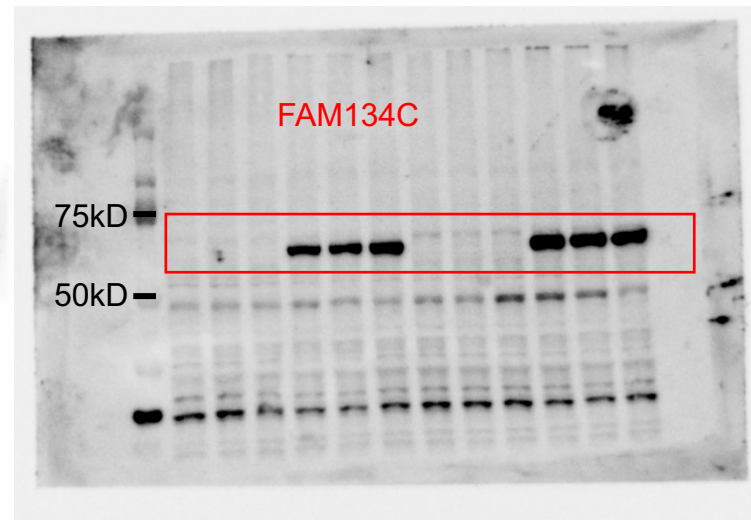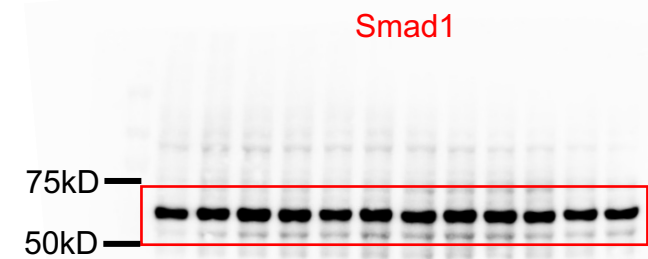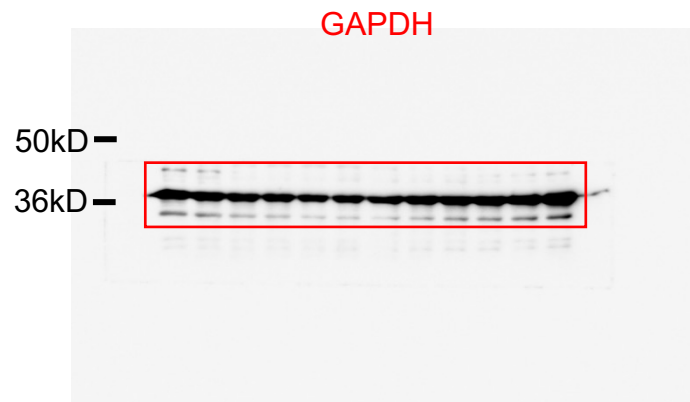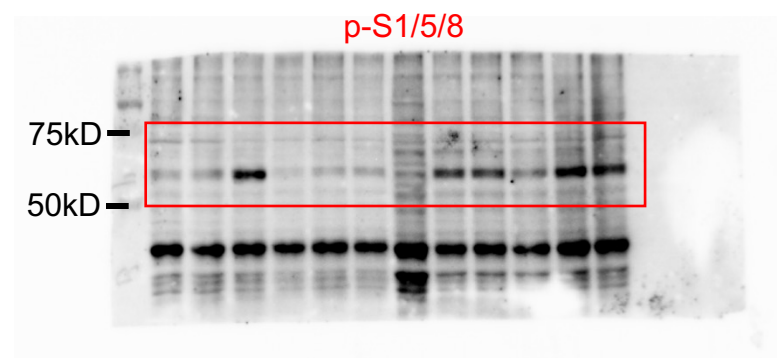

Supplement: Supplementary file 4 — Source data Fig. 1 [file 44318_2025_581_MOESM4_ESM.zip › Fig1/1H.pdf]

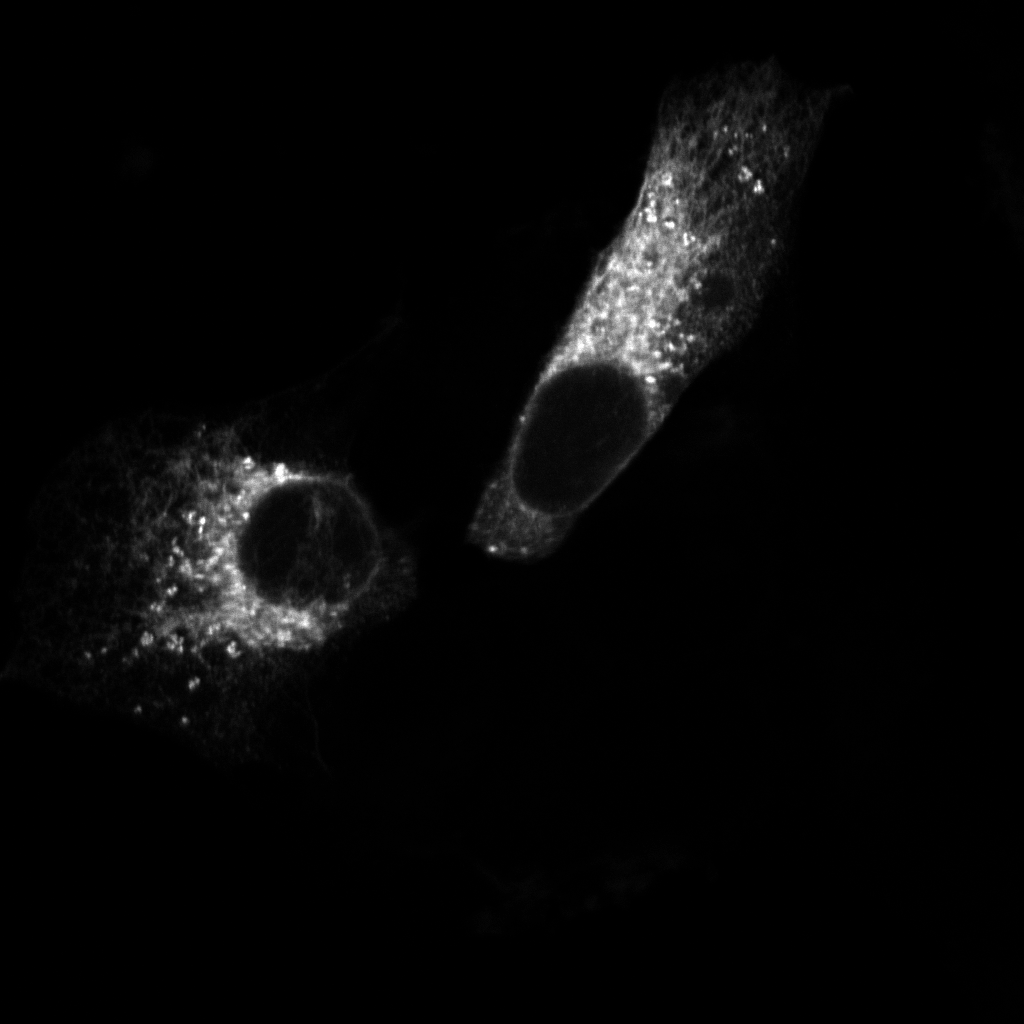

Supplement: Supplementary file 4 — Source data Fig. 1 [file 44318_2025_581_MOESM4_ESM.zip › Fig1/1I/U2OS FAM134C-GFP group FAM134C-GFP.tif]

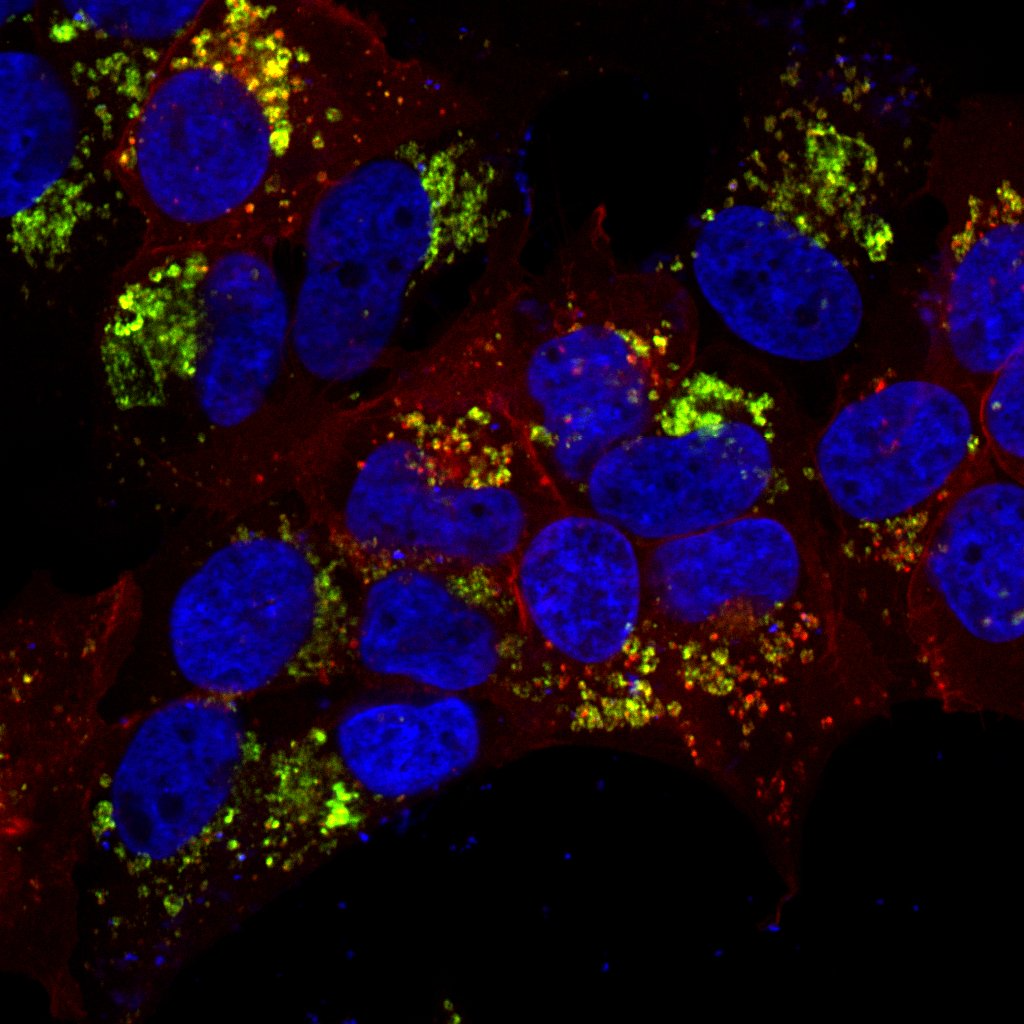

Supplement: Supplementary file 4 — Source data Fig. 1 [file 44318_2025_581_MOESM4_ESM.zip › Fig1/1I/U2OS GFP group Merged.tiff]

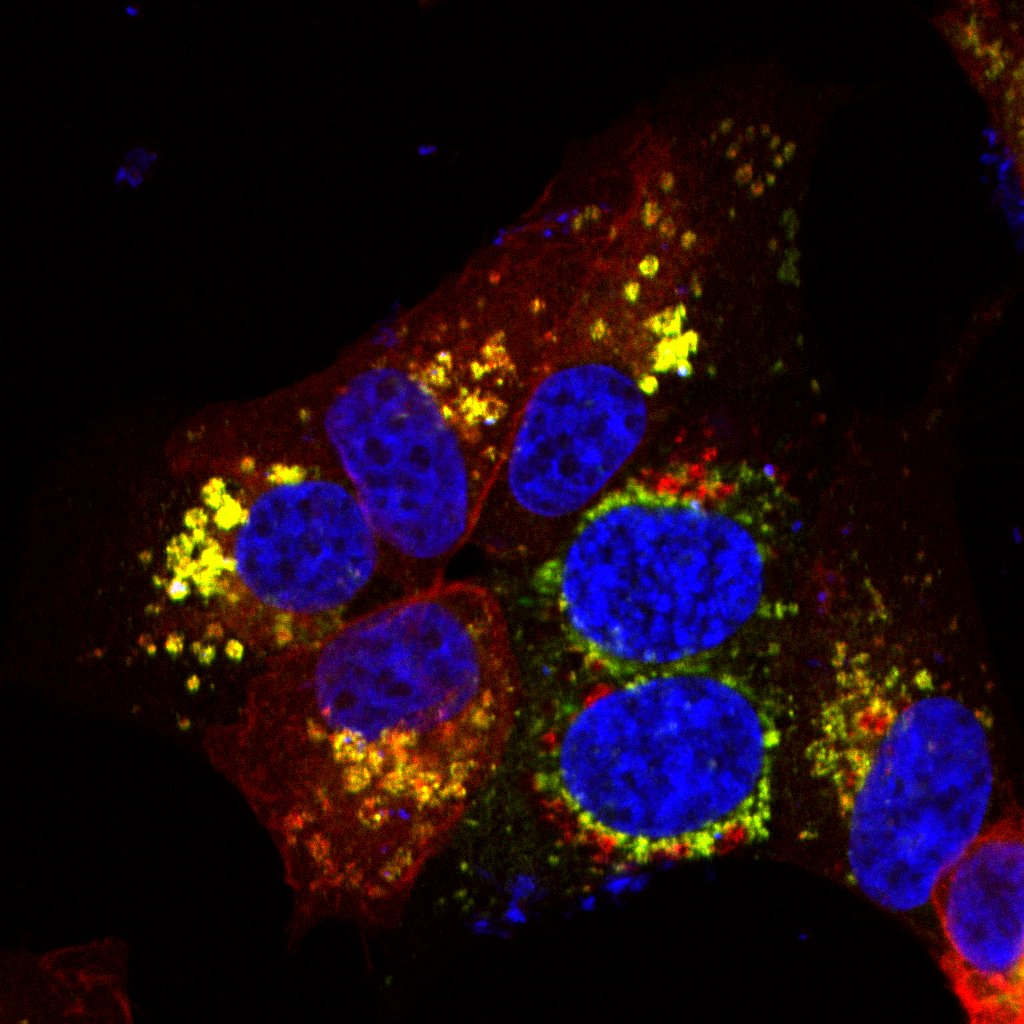

Supplement: Supplementary file 4 — Source data Fig. 1 [file 44318_2025_581_MOESM4_ESM.zip › Fig1/1I/U2OS FAM134C-GFP group Merged.tiff]

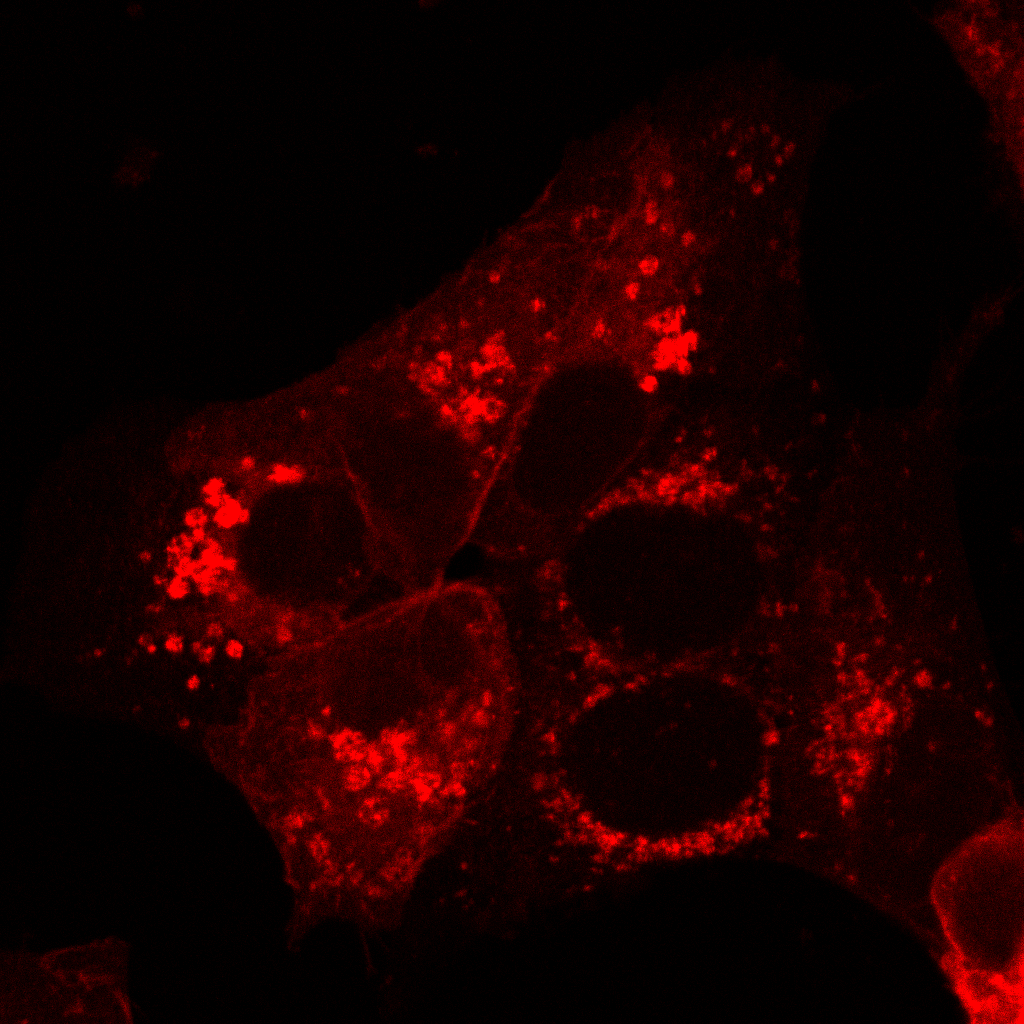

Supplement: Supplementary file 4 — Source data Fig. 1 [file 44318_2025_581_MOESM4_ESM.zip › Fig1/1I/U2OS FAM134C-GFP group BMPR1A-mCherry 1.tif]

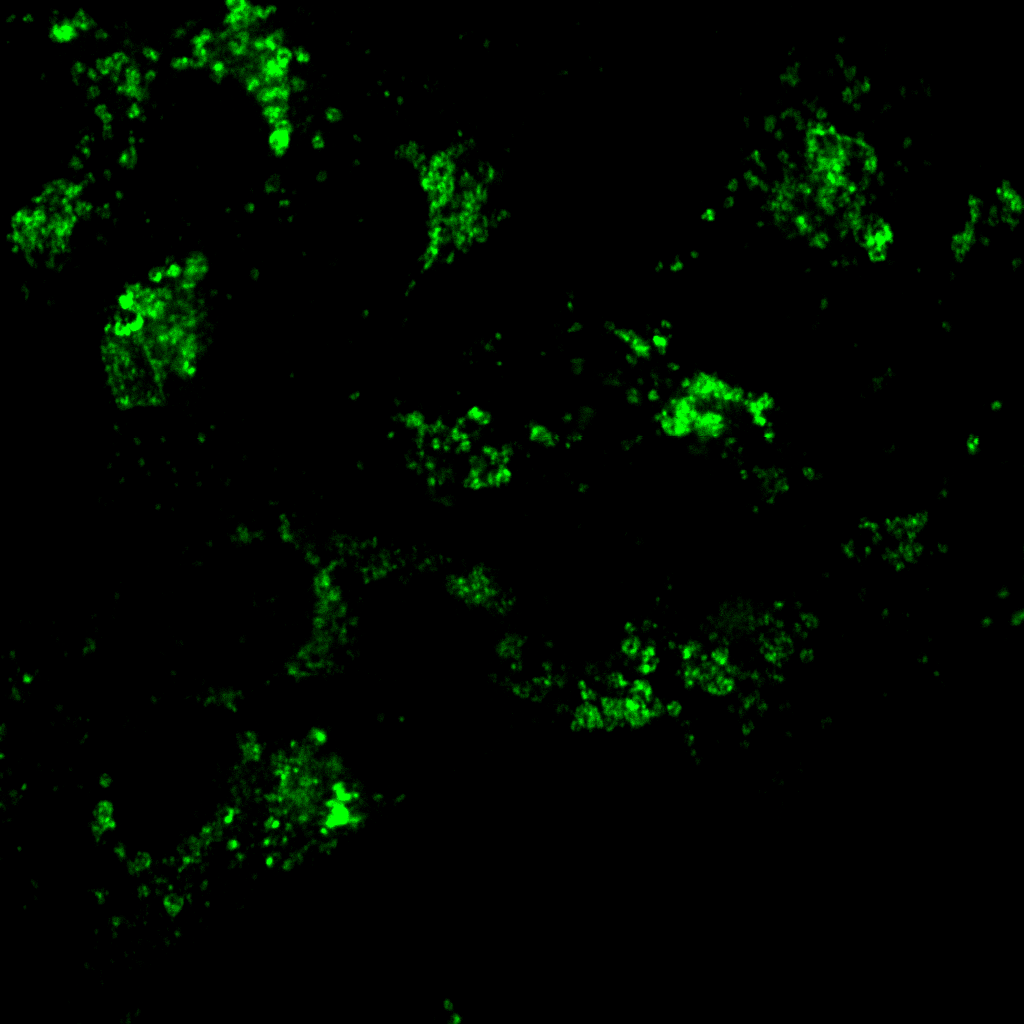

Supplement: Supplementary file 4 — Source data Fig. 1 [file 44318_2025_581_MOESM4_ESM.zip › Fig1/1I/U2OS U2OS GFP group LAMP1.tif]

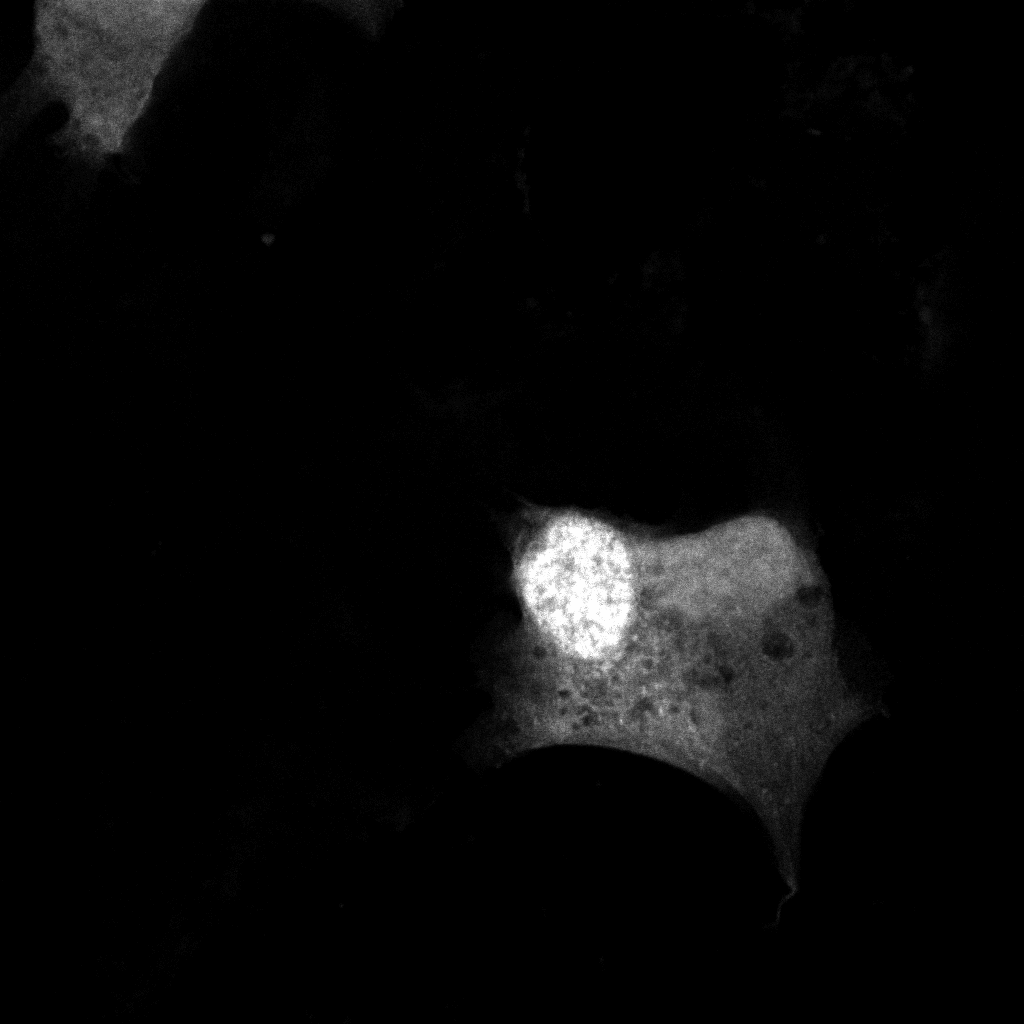

Supplement: Supplementary file 4 — Source data Fig. 1 [file 44318_2025_581_MOESM4_ESM.zip › Fig1/1I/U2OS U2OS GFP group GFP.tif]

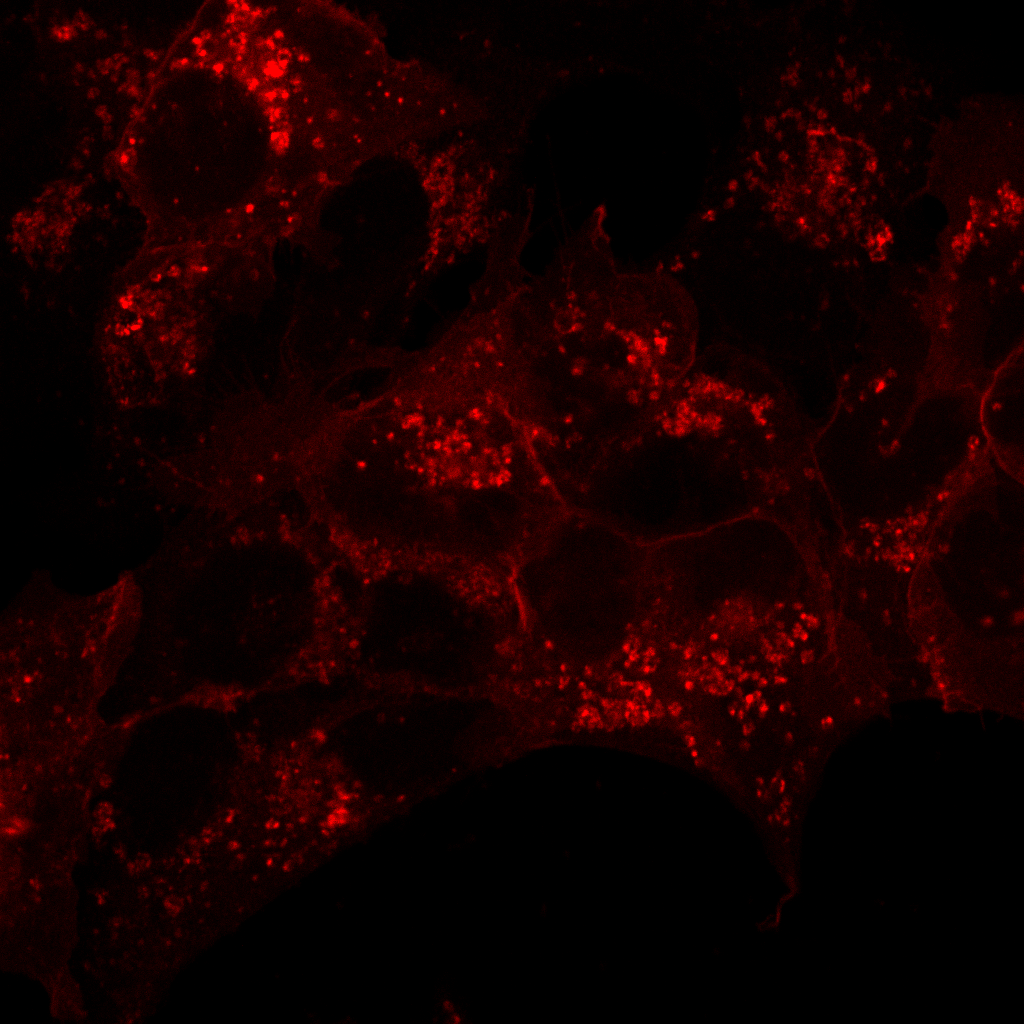

Supplement: Supplementary file 4 — Source data Fig. 1 [file 44318_2025_581_MOESM4_ESM.zip › Fig1/1I/U2OS GFP group BMPR1A-mCherry.tif]

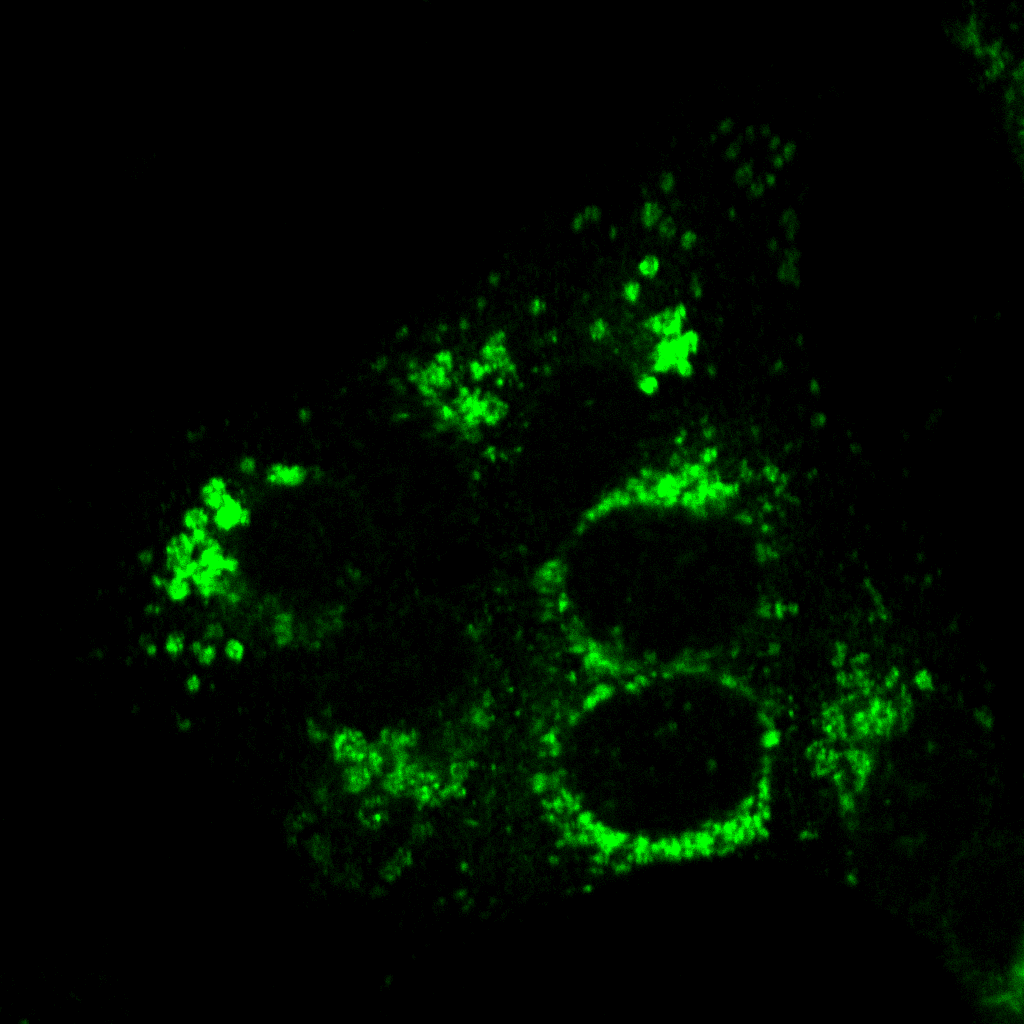

Supplement: Supplementary file 4 — Source data Fig. 1 [file 44318_2025_581_MOESM4_ESM.zip › Fig1/1I/U2OS FAM134C-GFP group LAMP1.tif]

BMPR1a

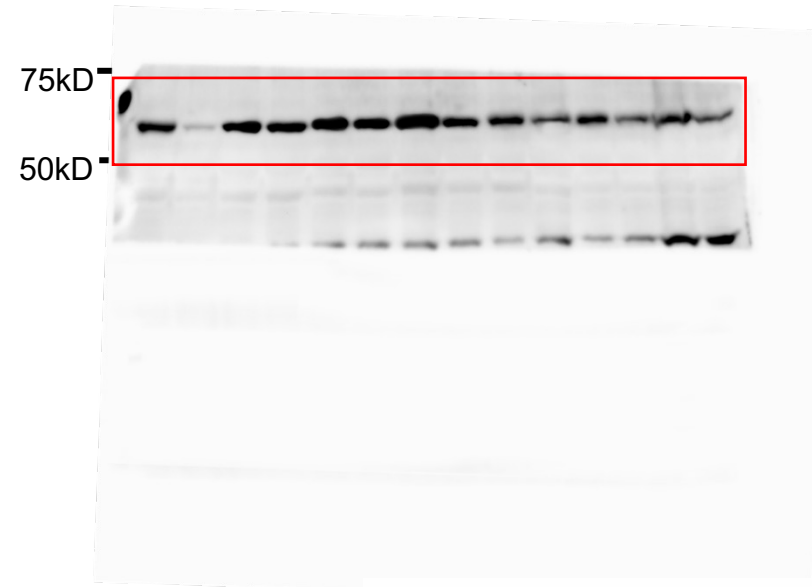

Flag

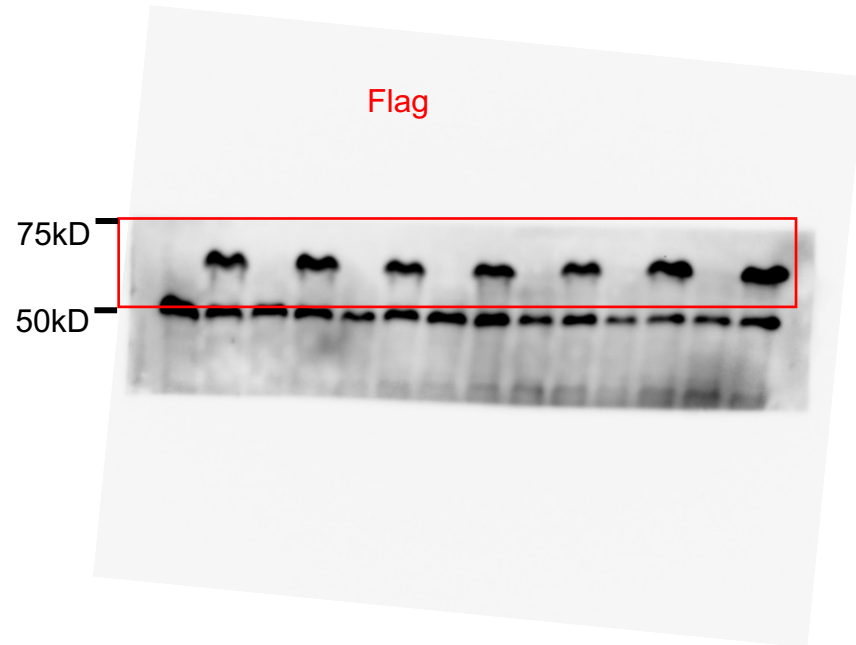

GAPDH

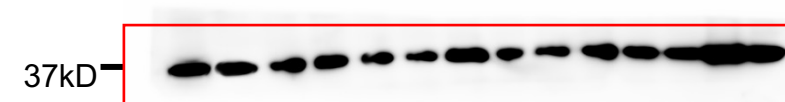

Supplement: Supplementary file 5 — Source data Fig. 2 [file 44318_2025_581_MOESM5_ESM.zip › Fig 2/2H.pdf]

BMPRI1A

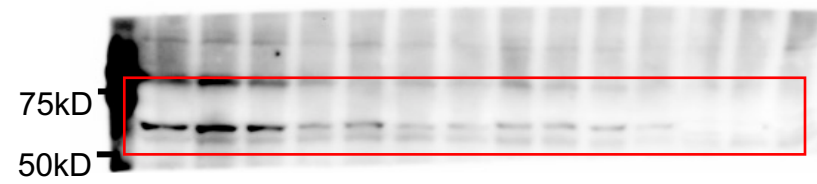

FAM134C

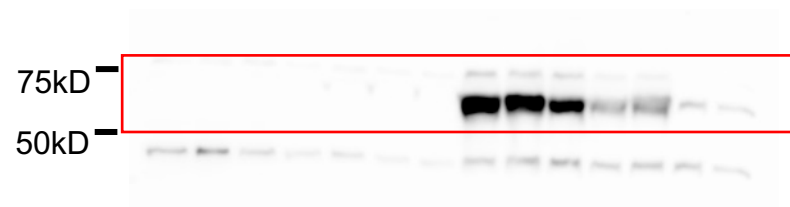

GAPDH

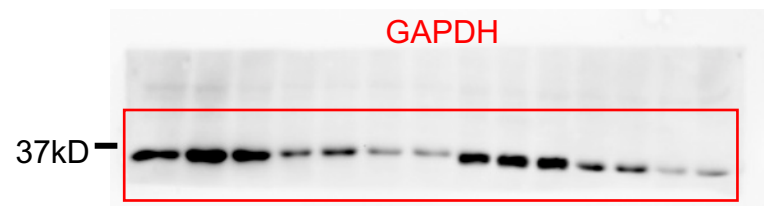

Supplement: Supplementary file 5 — Source data Fig. 2 [file 44318_2025_581_MOESM5_ESM.zip › Fig 2/2E.pdf]

Streptavidin Pulldown

BMPR1a

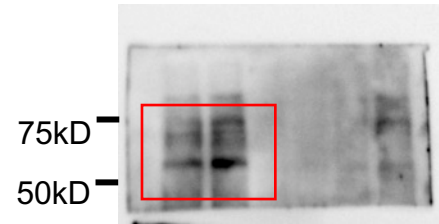

Actin

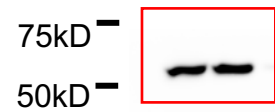

Flag

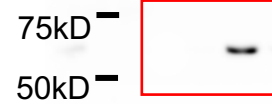

Whole Cell Lysate

BMPR1a

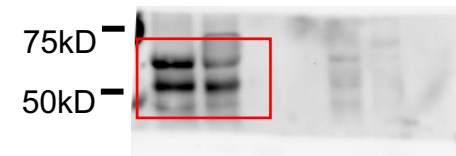

Actin

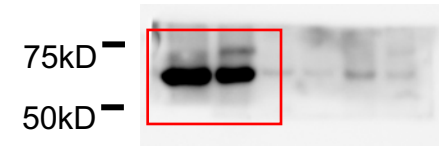

Flag

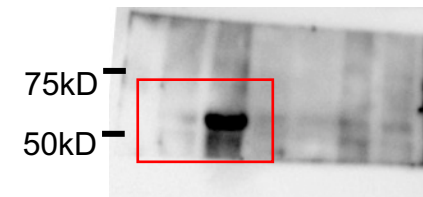

Supplement: Supplementary file 5 — Source data Fig. 2 [file 44318_2025_581_MOESM5_ESM.zip › Fig 2/2D.pdf]

BMPR1A

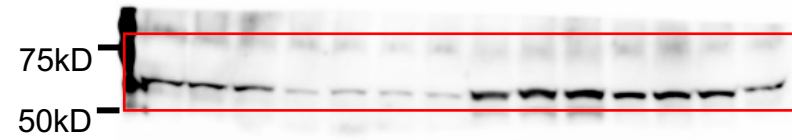

GAPDH

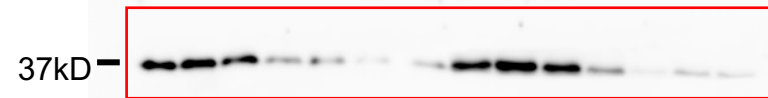

FAM134C

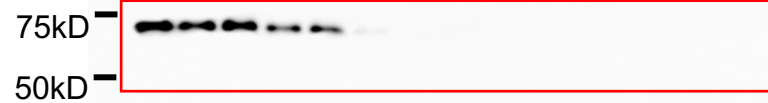

Supplement: Supplementary file 5 — Source data Fig. 2 [file 44318_2025_581_MOESM5_ESM.zip › Fig 2/2F.pdf]

BMPR1A

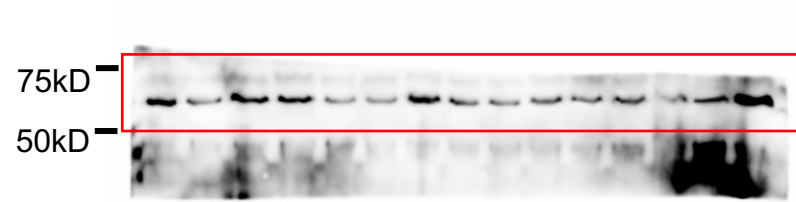

FAM134C

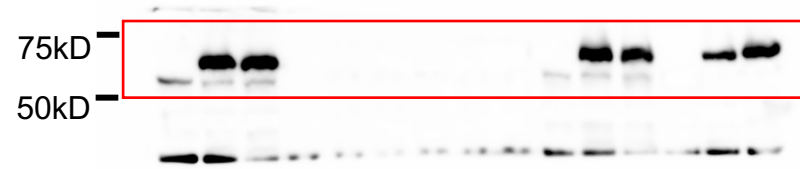

E-Cadherin

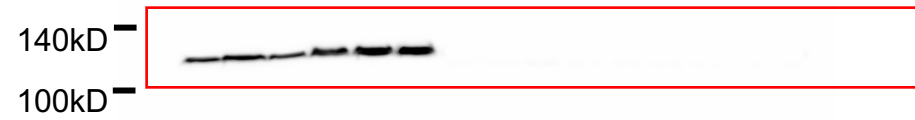

lamp1

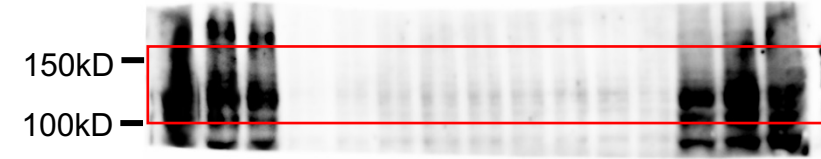

EEA1

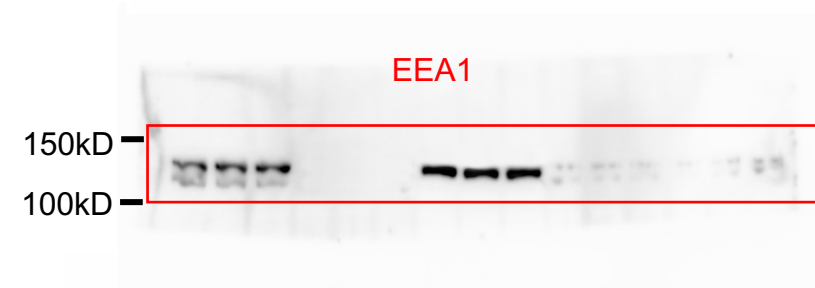

PDAI3

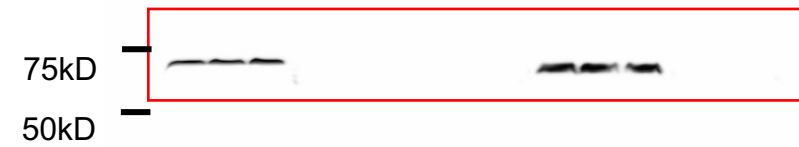

Supplement: Supplementary file 5 — Source data Fig. 2 [file 44318_2025_581_MOESM5_ESM.zip › Fig 2/2G.pdf]

Flag

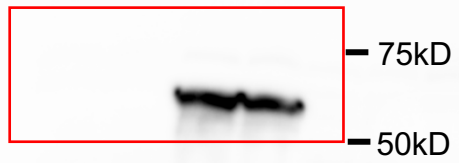

GFP

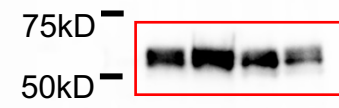

Supplement: Supplementary file 5 — Source data Fig. 2 [file 44318_2025_581_MOESM5_ESM.zip › Fig 2/2C.pdf]

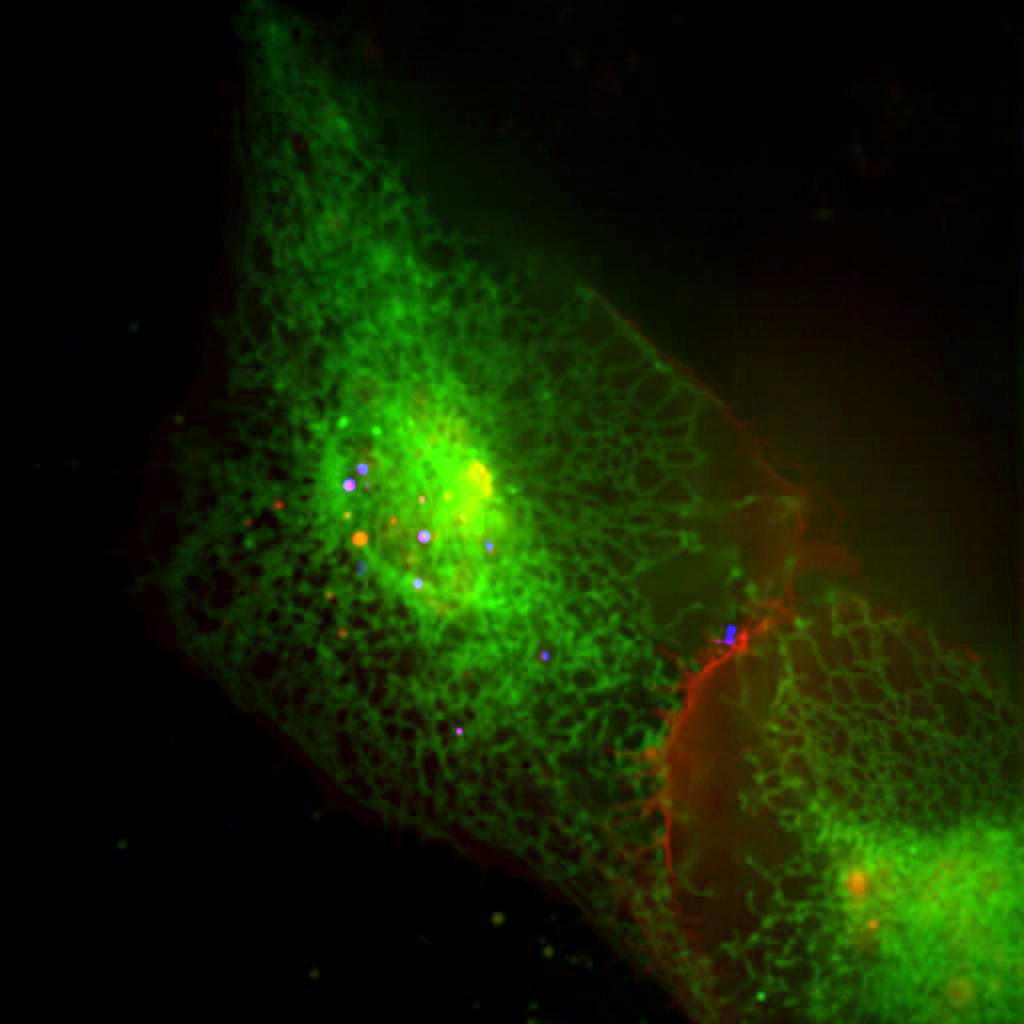

Supplement: Supplementary file 5 — Source data Fig. 2 [file 44318_2025_581_MOESM5_ESM.zip › Fig 2/2I/00∩╝Ü00.64.tif]

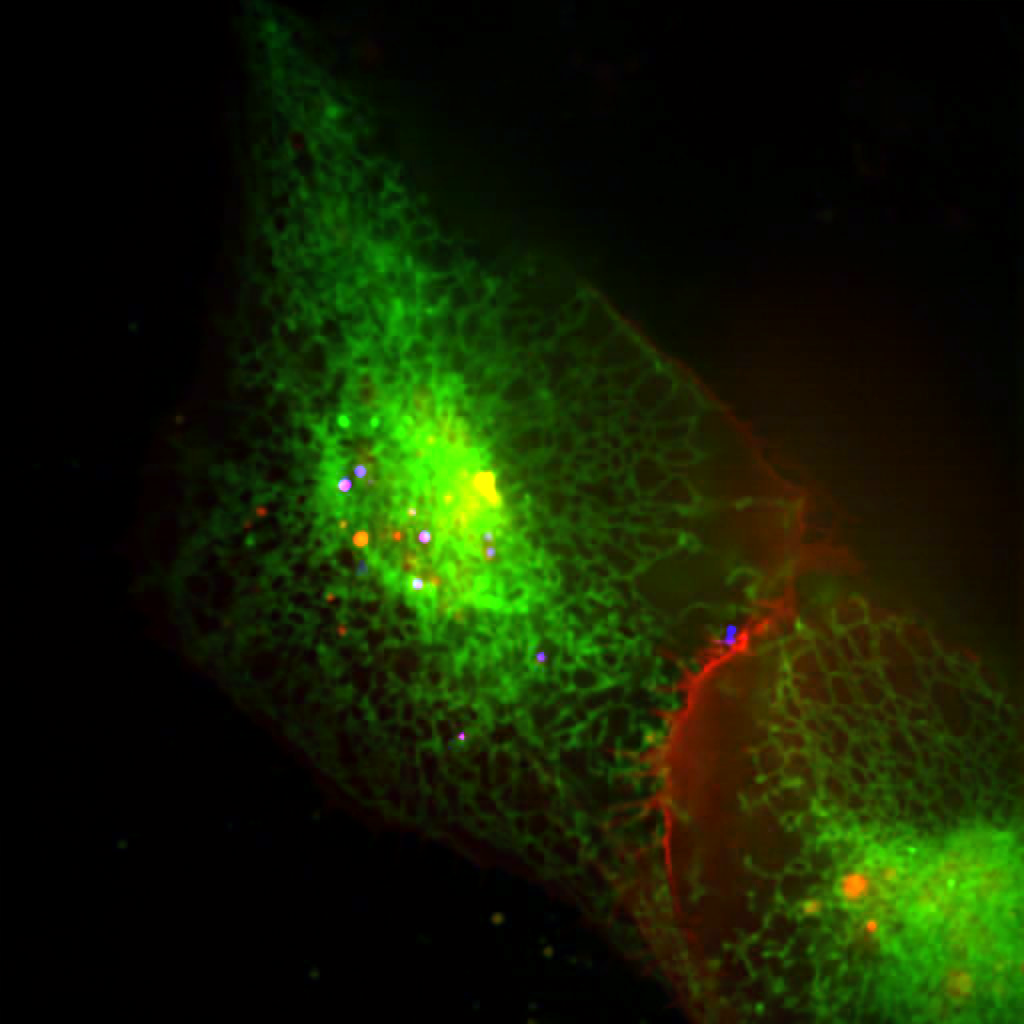

Supplement: Supplementary file 5 — Source data Fig. 2 [file 44318_2025_581_MOESM5_ESM.zip › Fig 2/2I/00∩╝Ü01.31.tif]

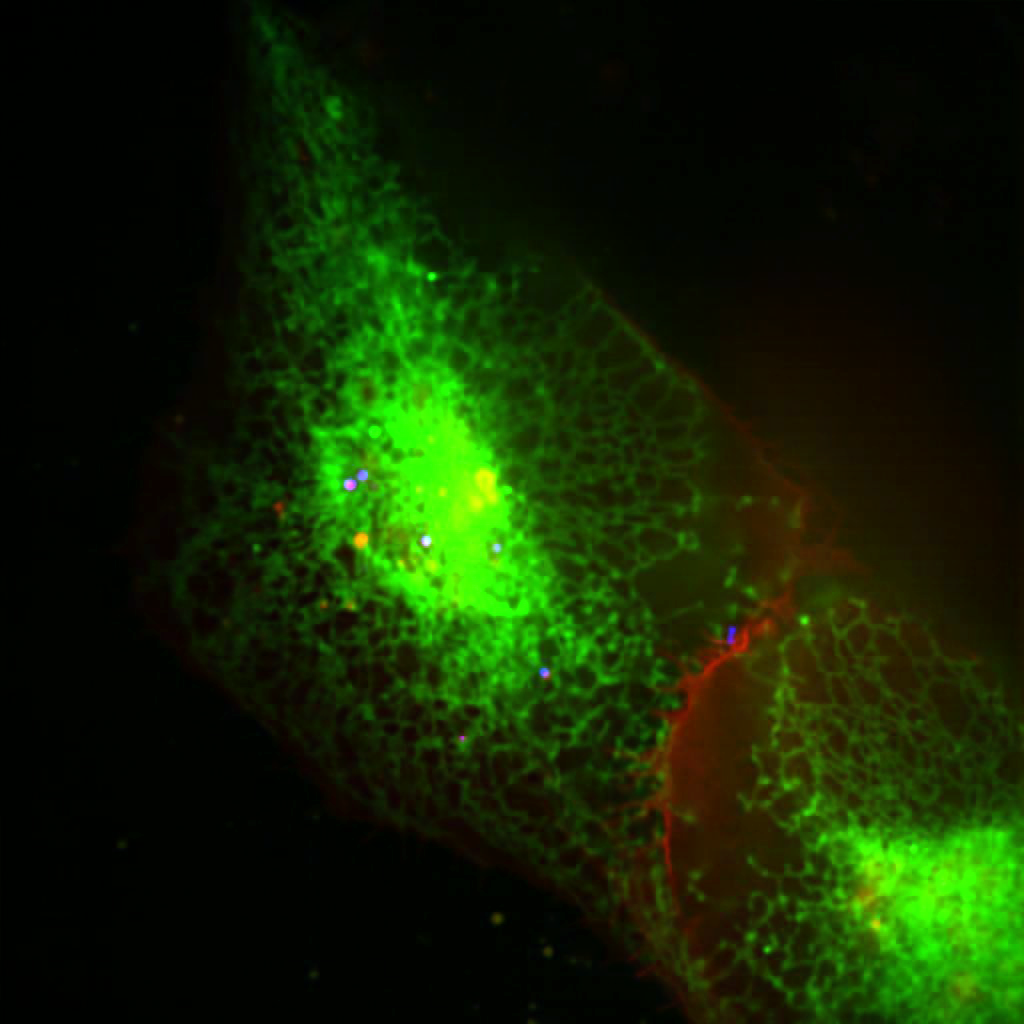

Supplement: Supplementary file 5 — Source data Fig. 2 [file 44318_2025_581_MOESM5_ESM.zip › Fig 2/2I/00∩╝Ü01.91.tif]

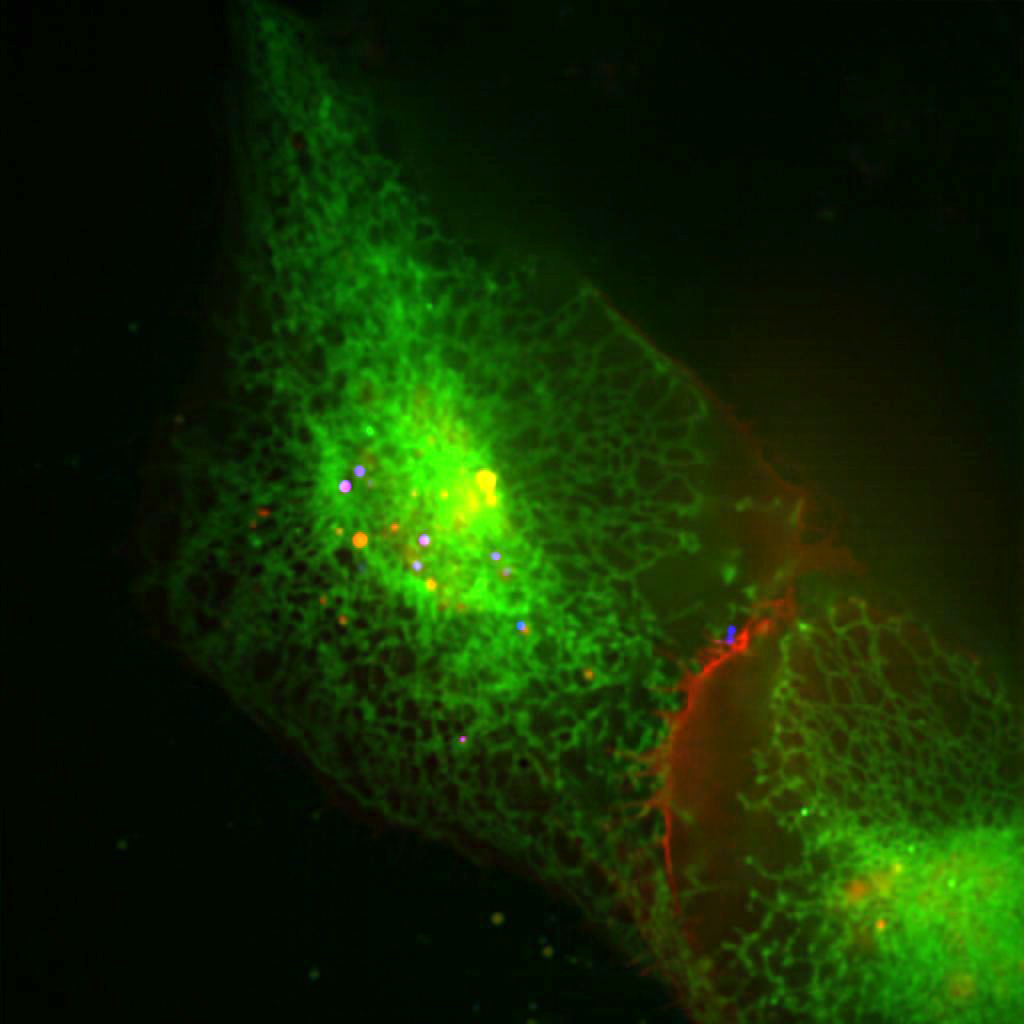

Supplement: Supplementary file 5 — Source data Fig. 2 [file 44318_2025_581_MOESM5_ESM.zip › Fig 2/2I/00∩╝Ü01.59.tif]

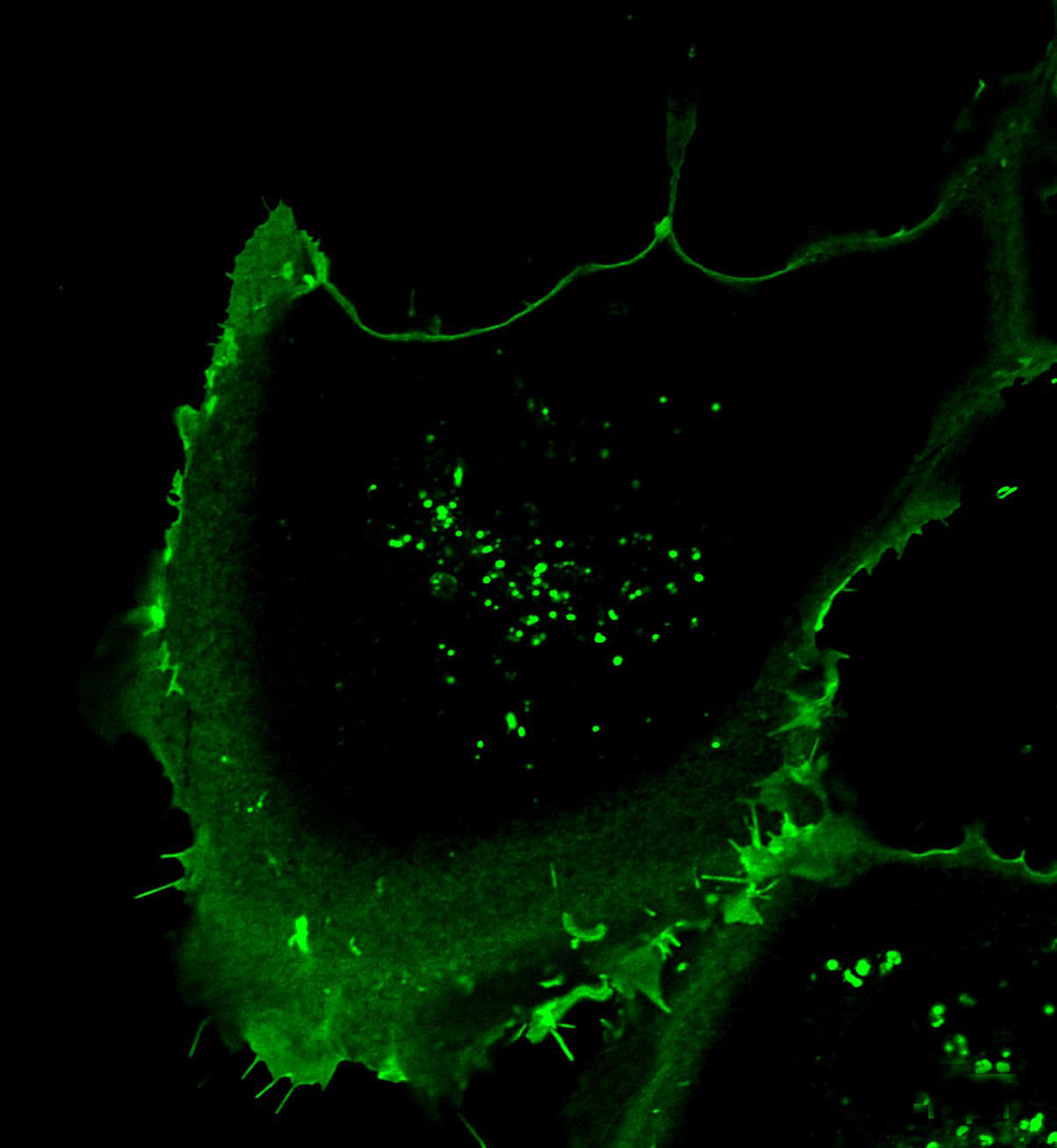

Supplement: Supplementary file 5 — Source data Fig. 2 [file 44318_2025_581_MOESM5_ESM.zip › Fig 2/2B/BMPR1a-Rush with biotin.tiff]

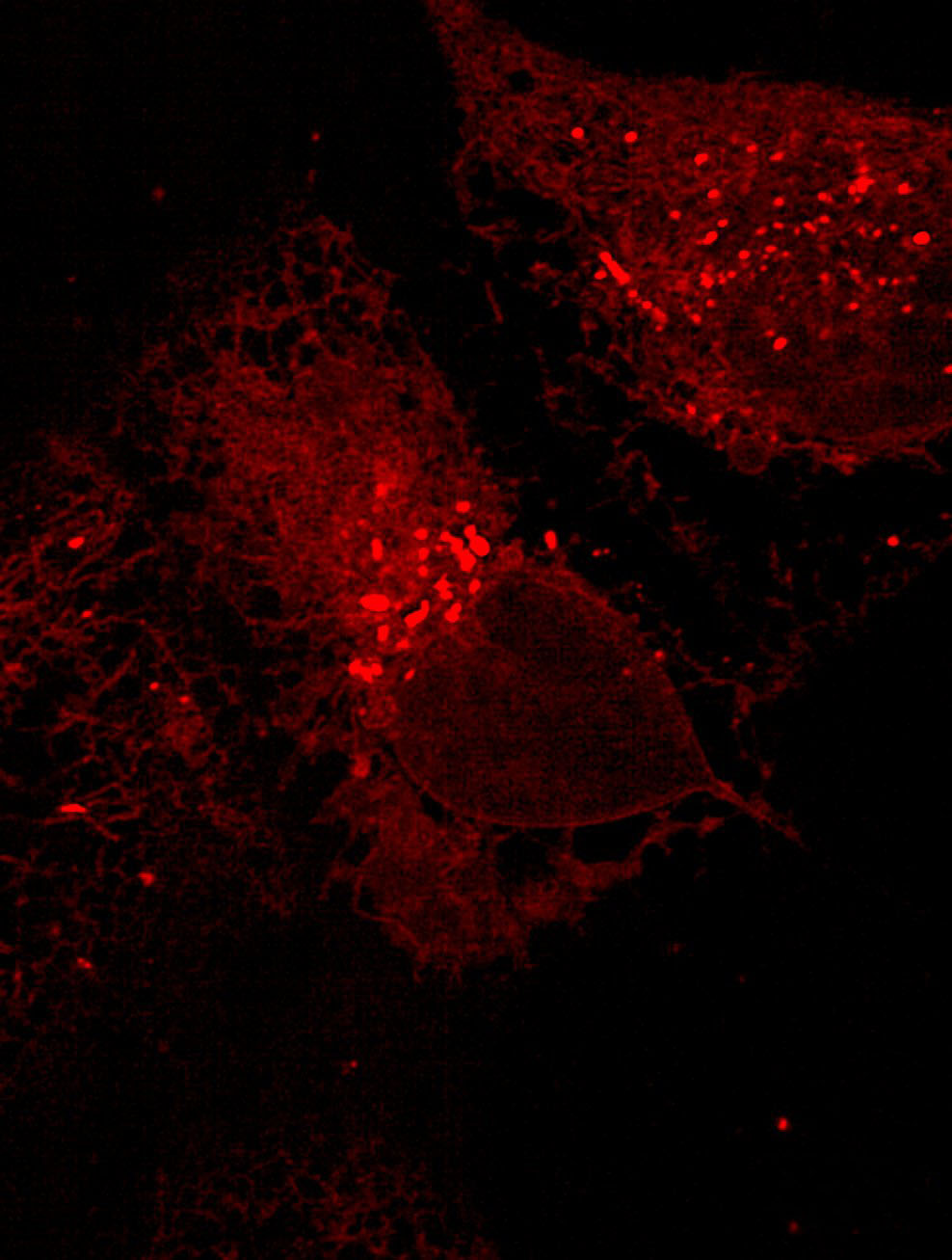

Supplement: Supplementary file 5 — Source data Fig. 2 [file 44318_2025_581_MOESM5_ESM.zip › Fig 2/2B/ER tracker without biotin.tiff]

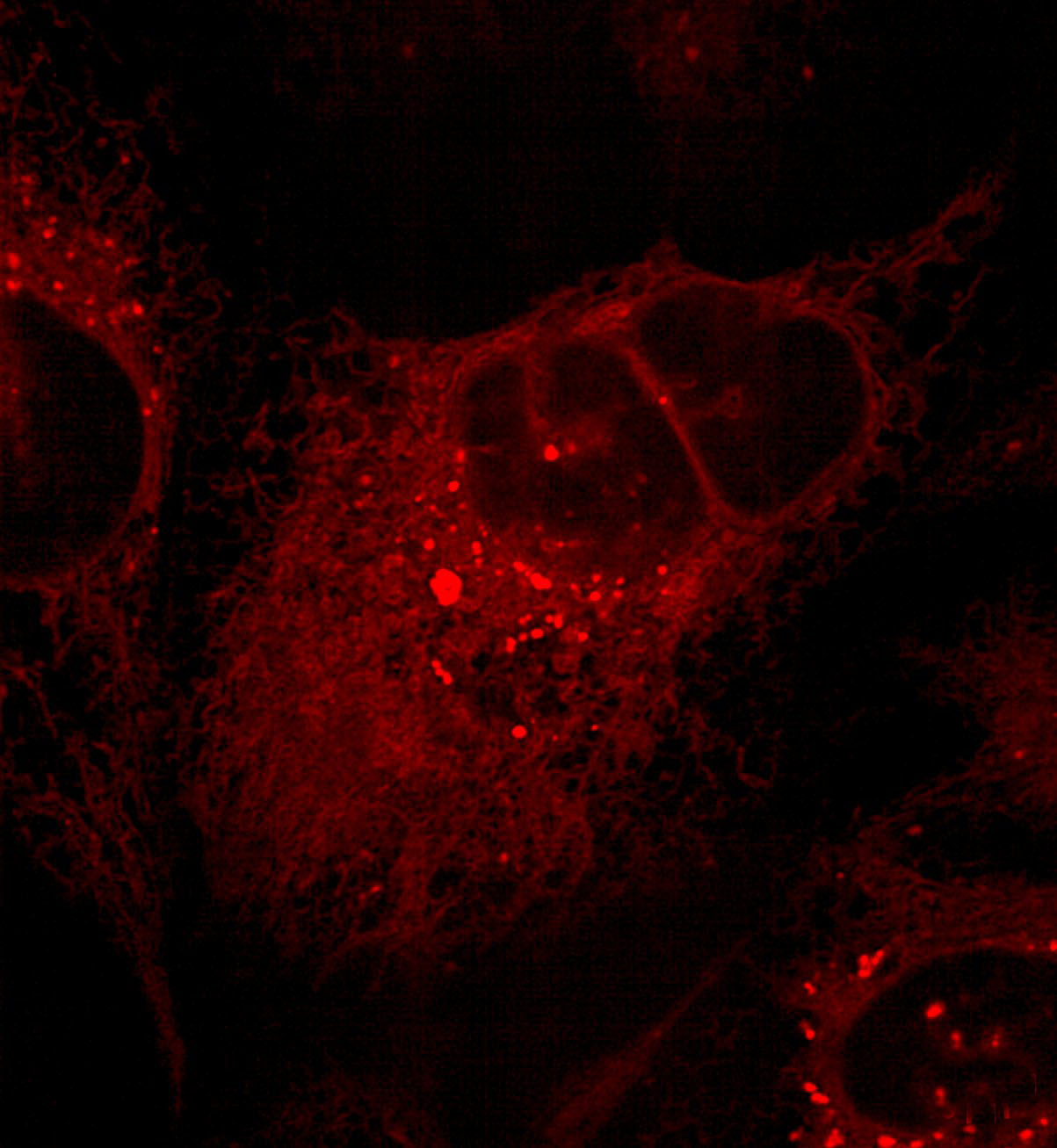

Supplement: Supplementary file 5 — Source data Fig. 2 [file 44318_2025_581_MOESM5_ESM.zip › Fig 2/2B/ER trakcer with biotin.tiff]

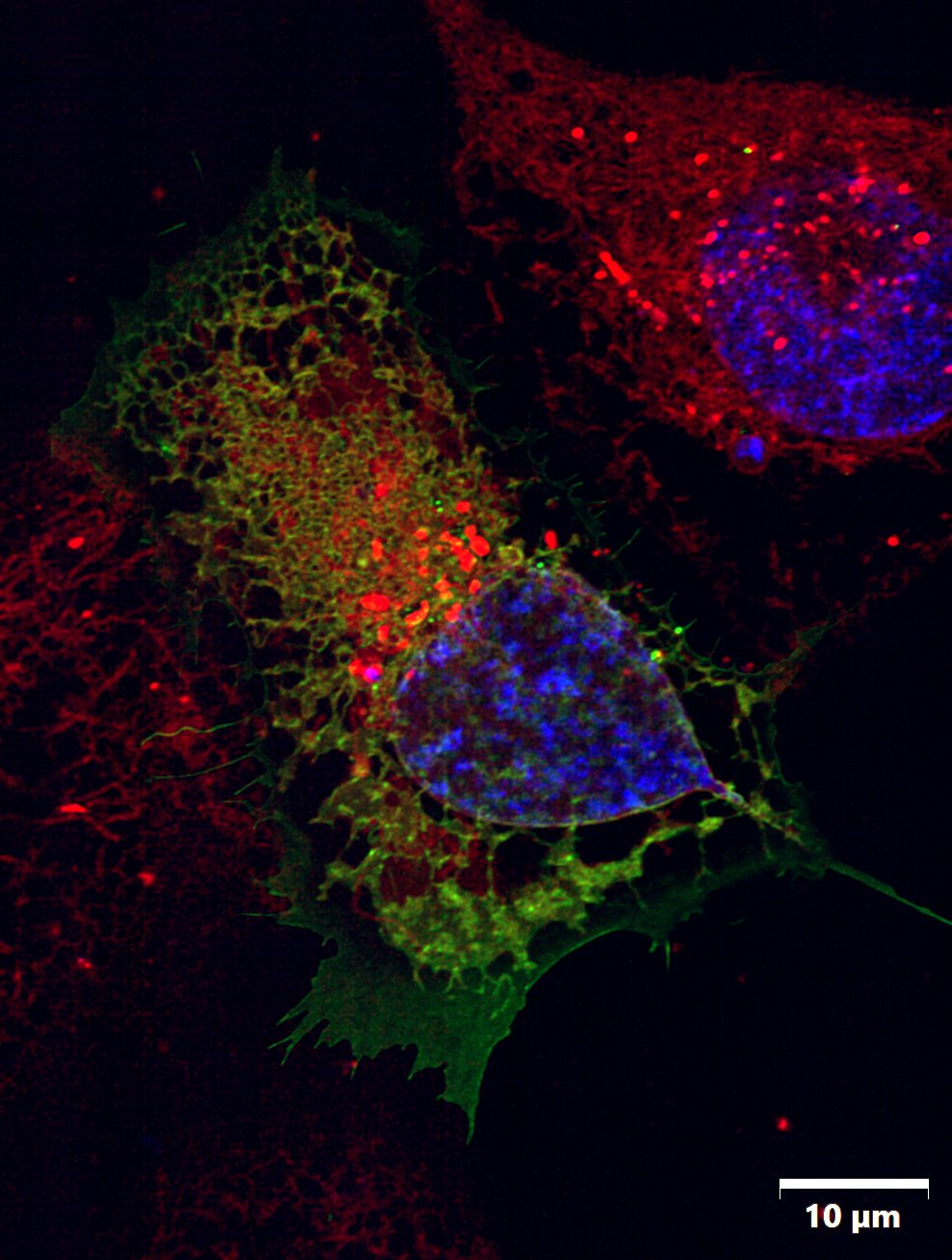

Supplement: Supplementary file 5 — Source data Fig. 2 [file 44318_2025_581_MOESM5_ESM.zip › Fig 2/2B/merged without biotin.tiff]

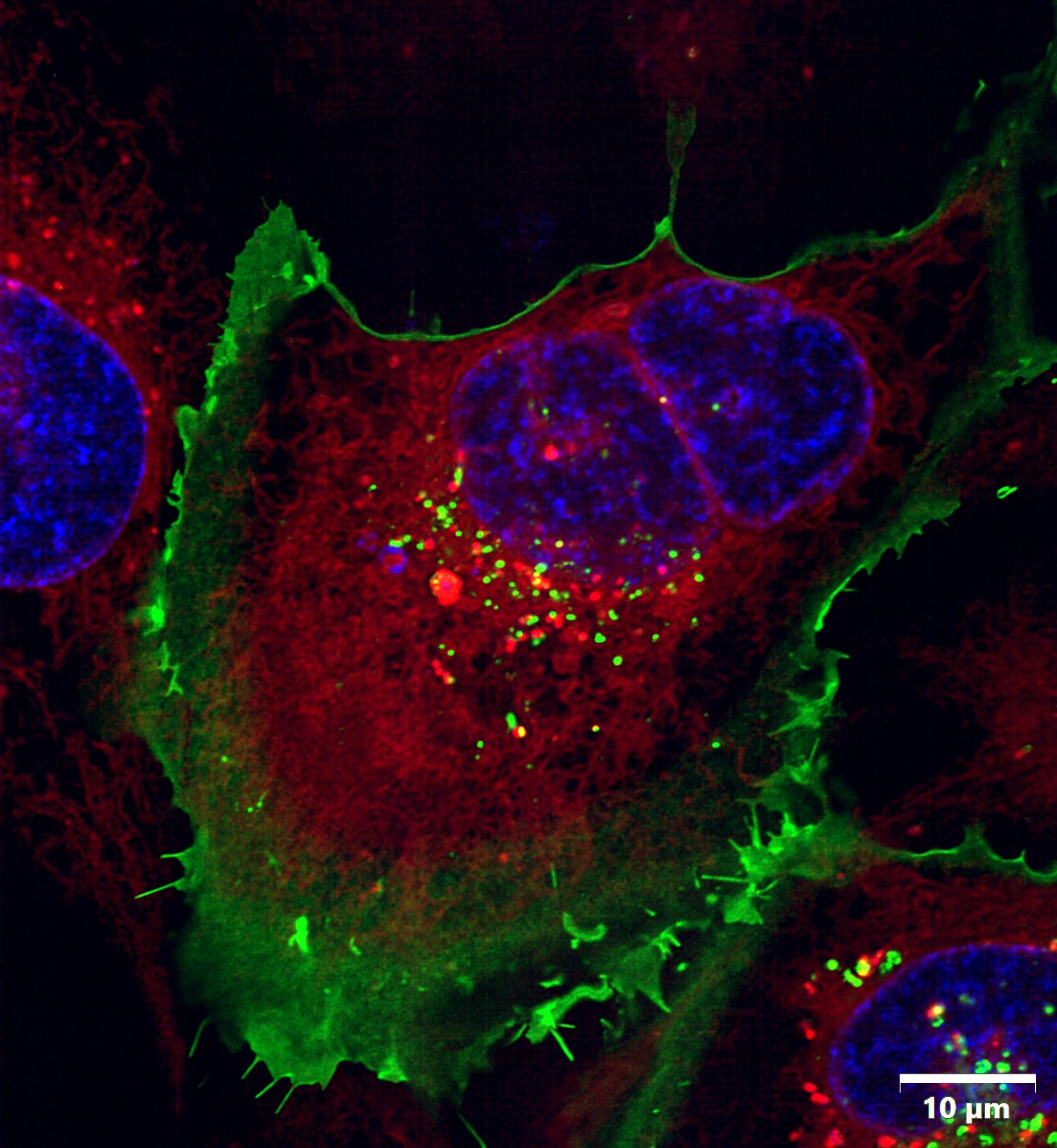

Supplement: Supplementary file 5 — Source data Fig. 2 [file 44318_2025_581_MOESM5_ESM.zip › Fig 2/2B/merged with biotin.tiff]

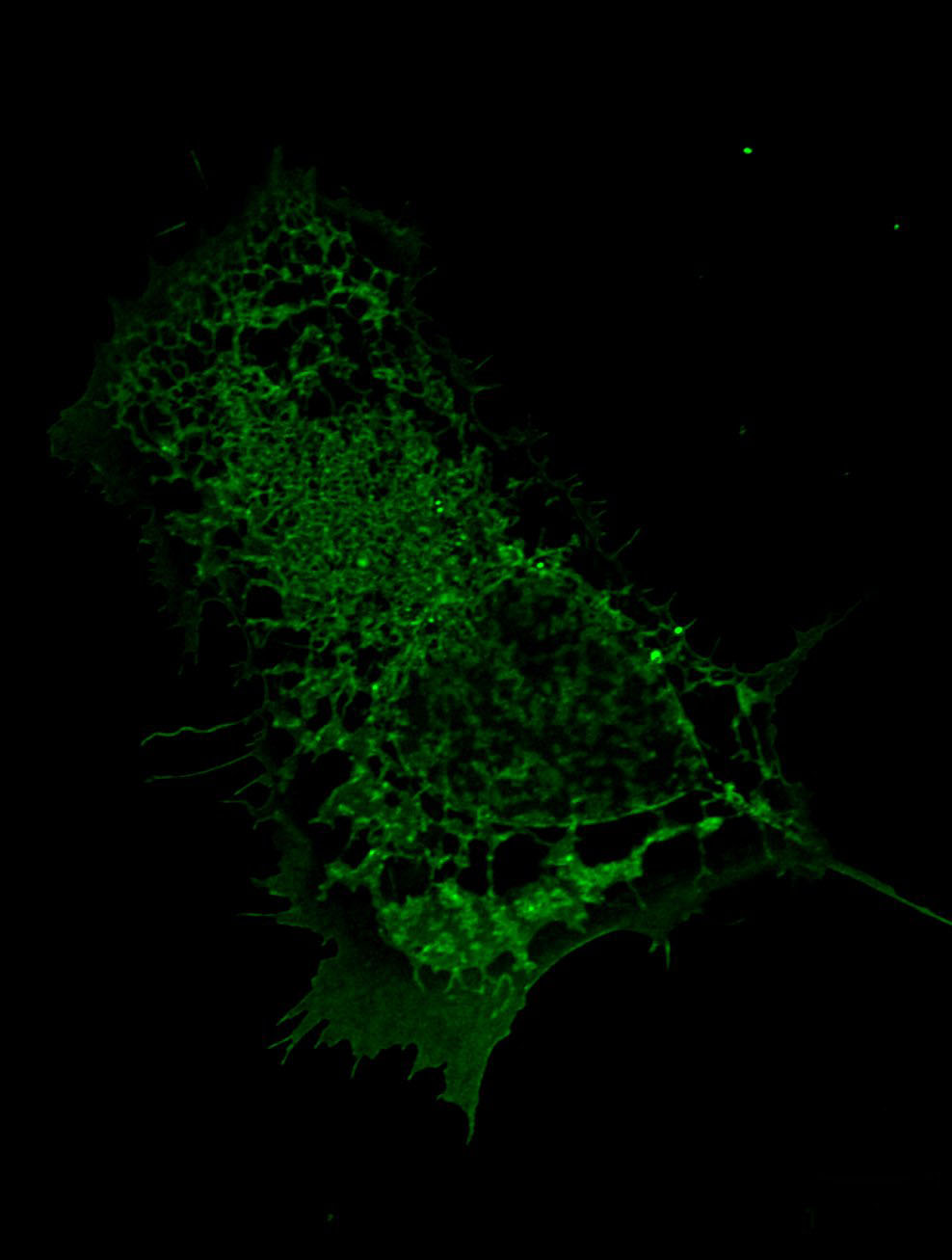

Supplement: Supplementary file 5 — Source data Fig. 2 [file 44318_2025_581_MOESM5_ESM.zip › Fig 2/2B/BMPR1a-Rush without biotin.tiff]

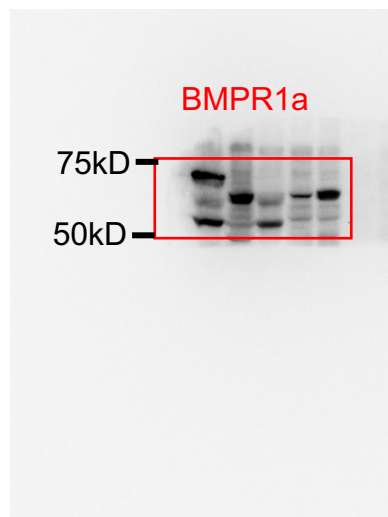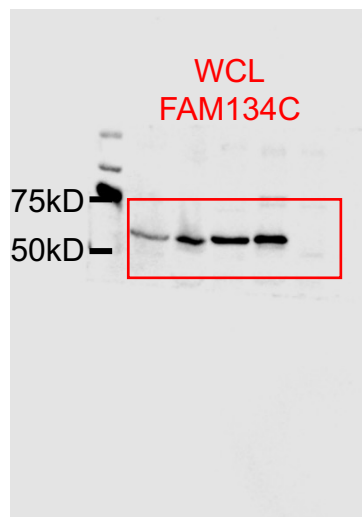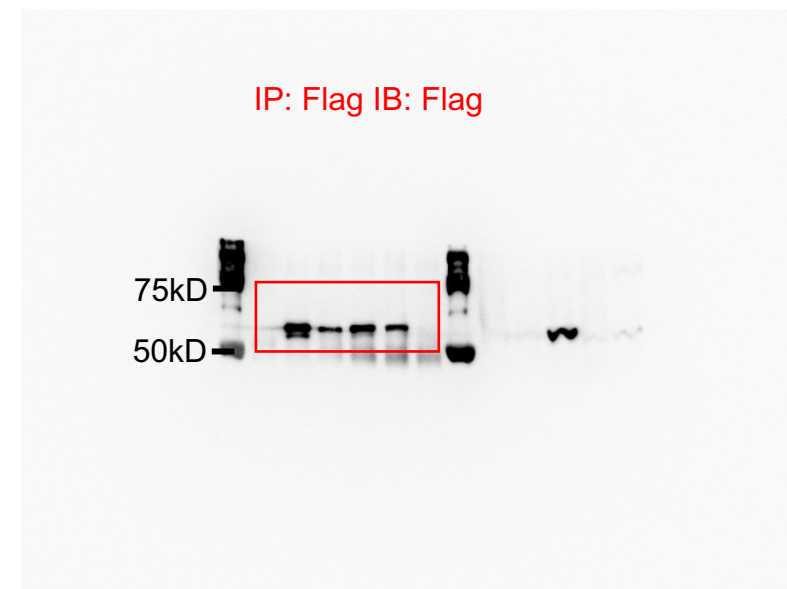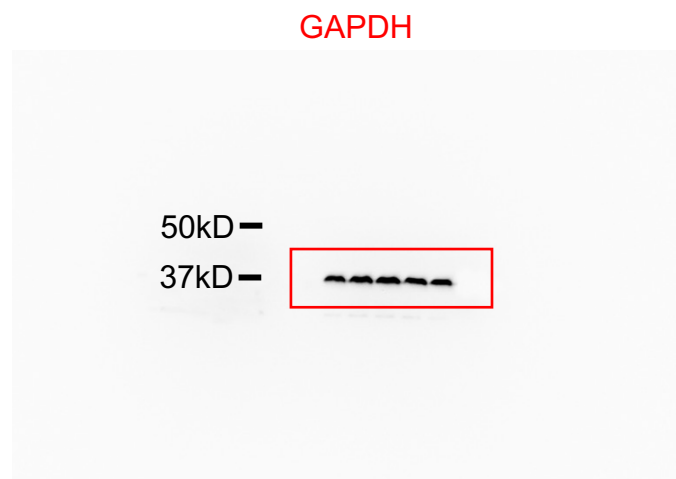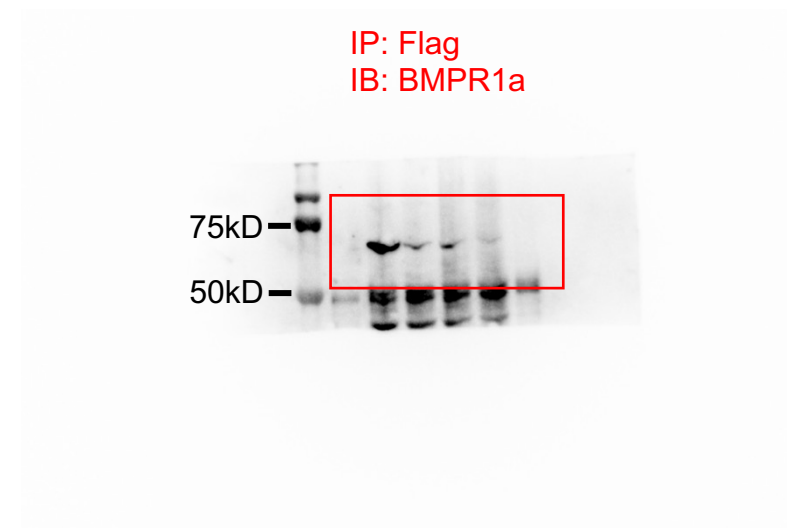

Supplement: Supplementary file 6 — Source data Fig. 3 [file 44318_2025_581_MOESM6_ESM.zip › Fig 3/3A.pdf]

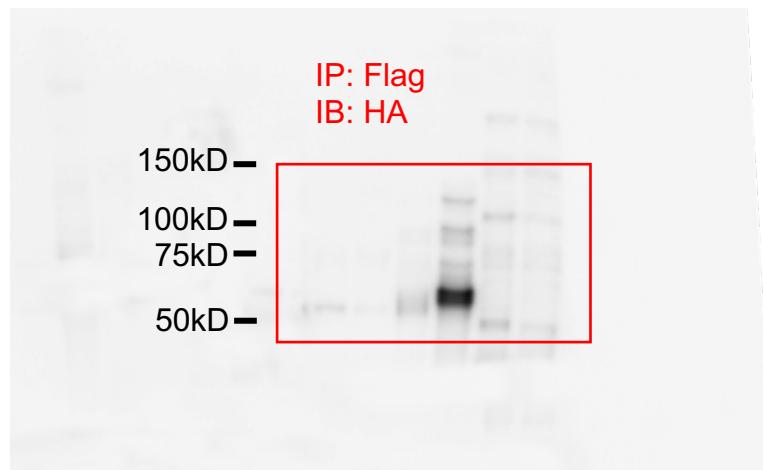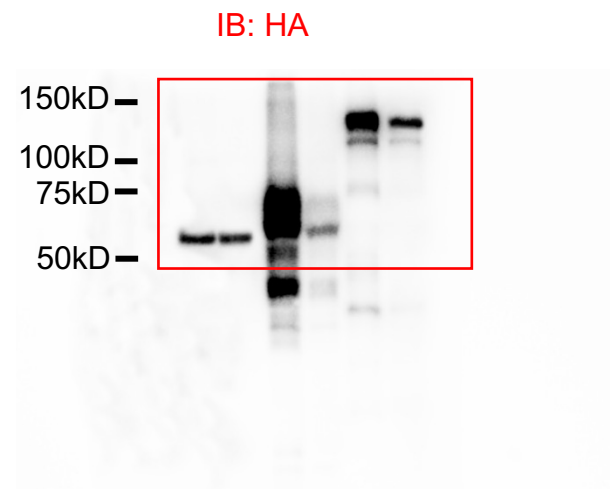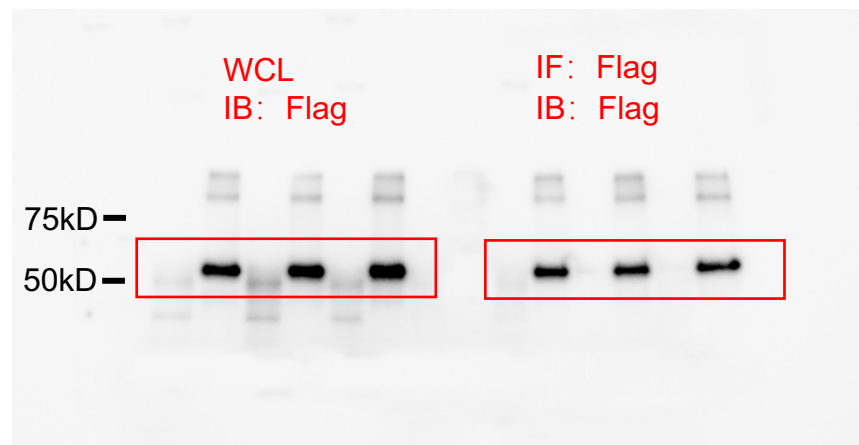

Supplement: Supplementary file 6 — Source data Fig. 3 [file 44318_2025_581_MOESM6_ESM.zip › Fig 3/3B.pdf]

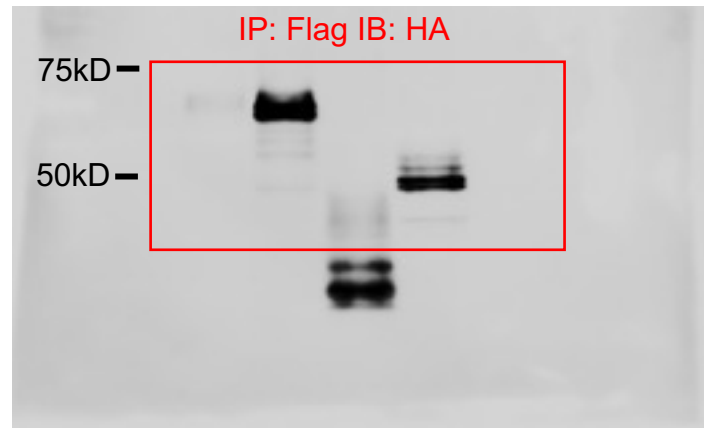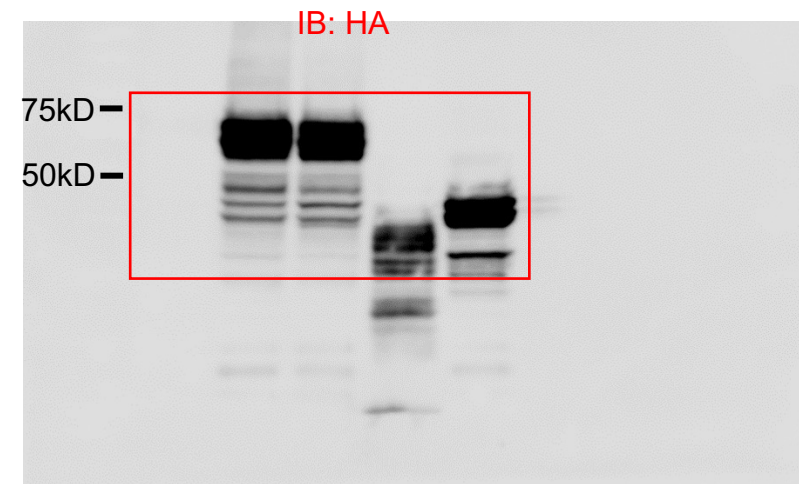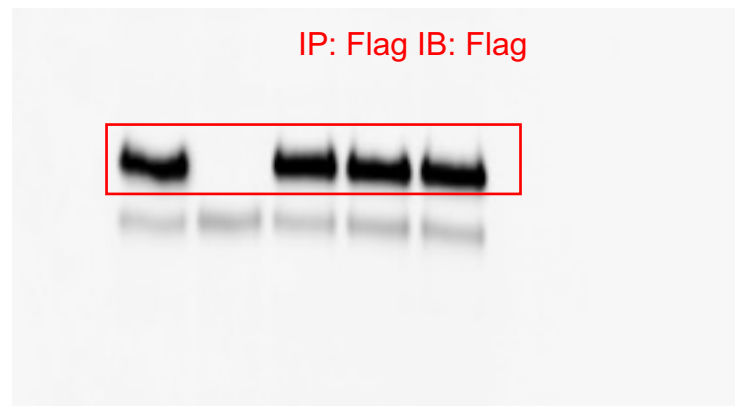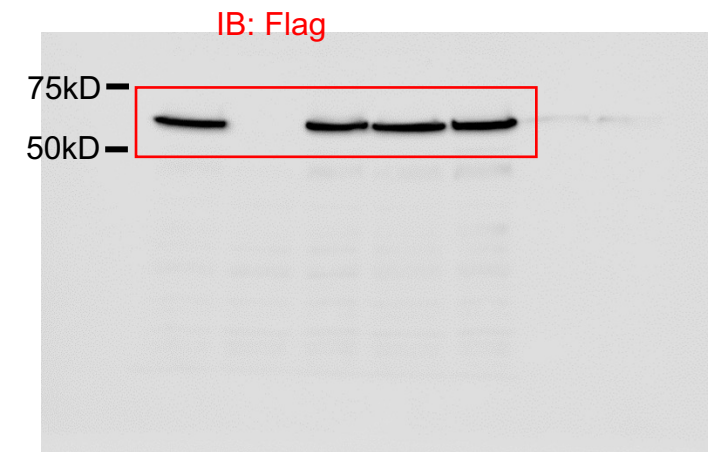

Supplement: Supplementary file 6 — Source data Fig. 3 [file 44318_2025_581_MOESM6_ESM.zip › Fig 3/3C.pdf]

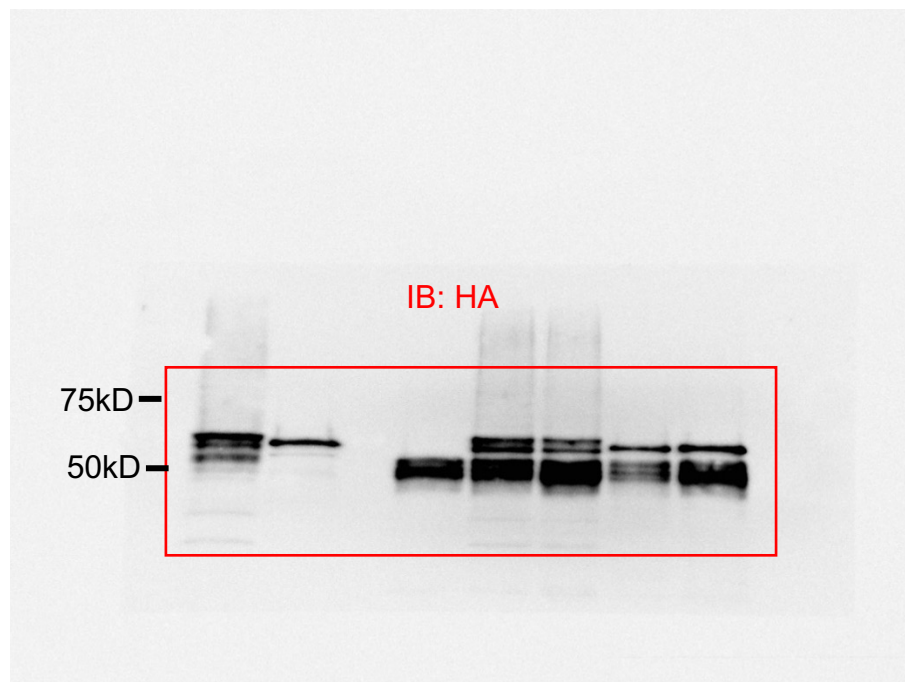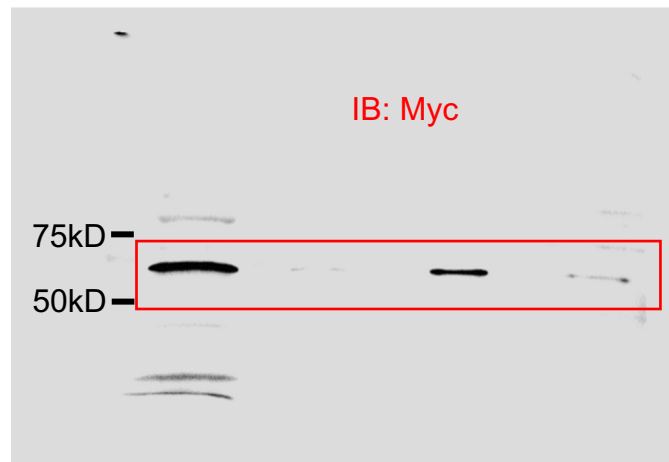

Supplement: Supplementary file 6 — Source data Fig. 3 [file 44318_2025_581_MOESM6_ESM.zip › Fig 3/3D.pdf]

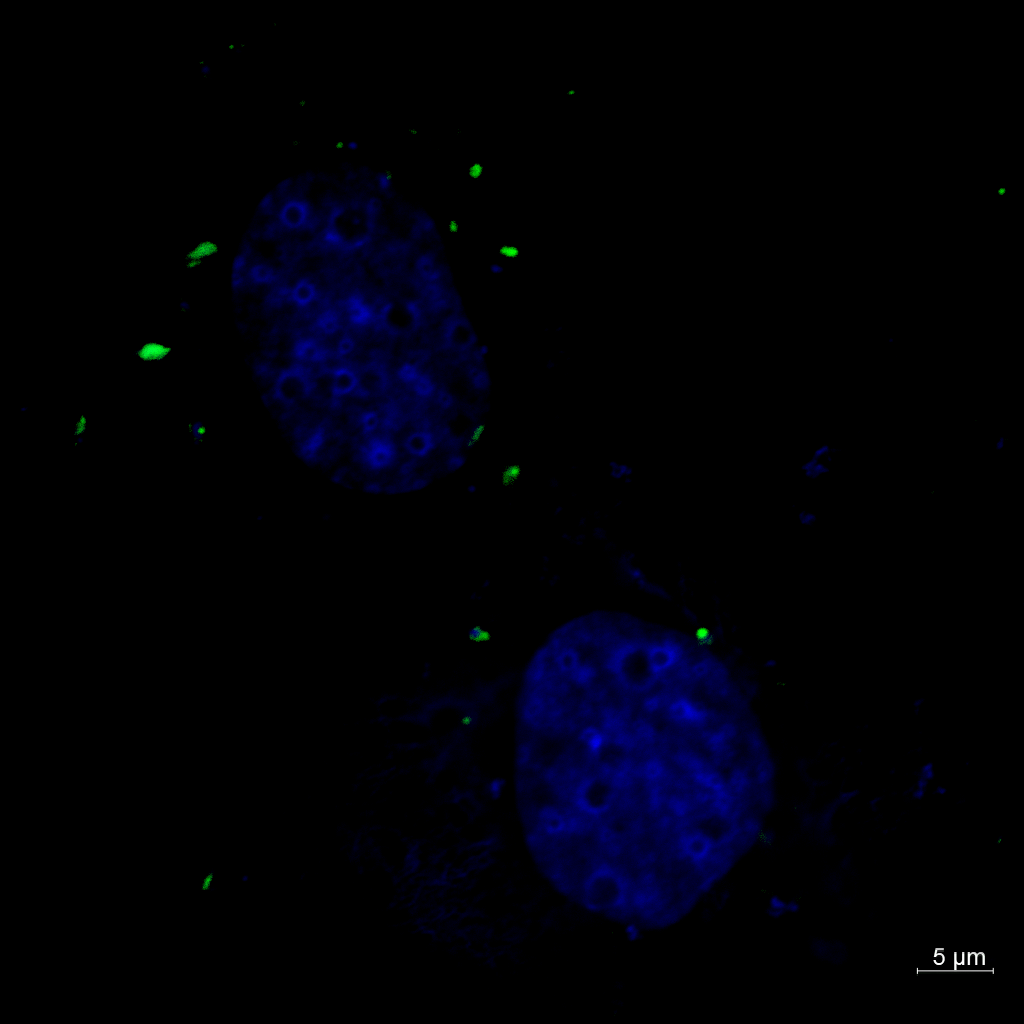

Supplement: Supplementary file 6 — Source data Fig. 3 [file 44318_2025_581_MOESM6_ESM.zip › Fig 3/3E/U2OS pXF-FAM134C-2YC pRK-BMPR1A-2YN.tif]

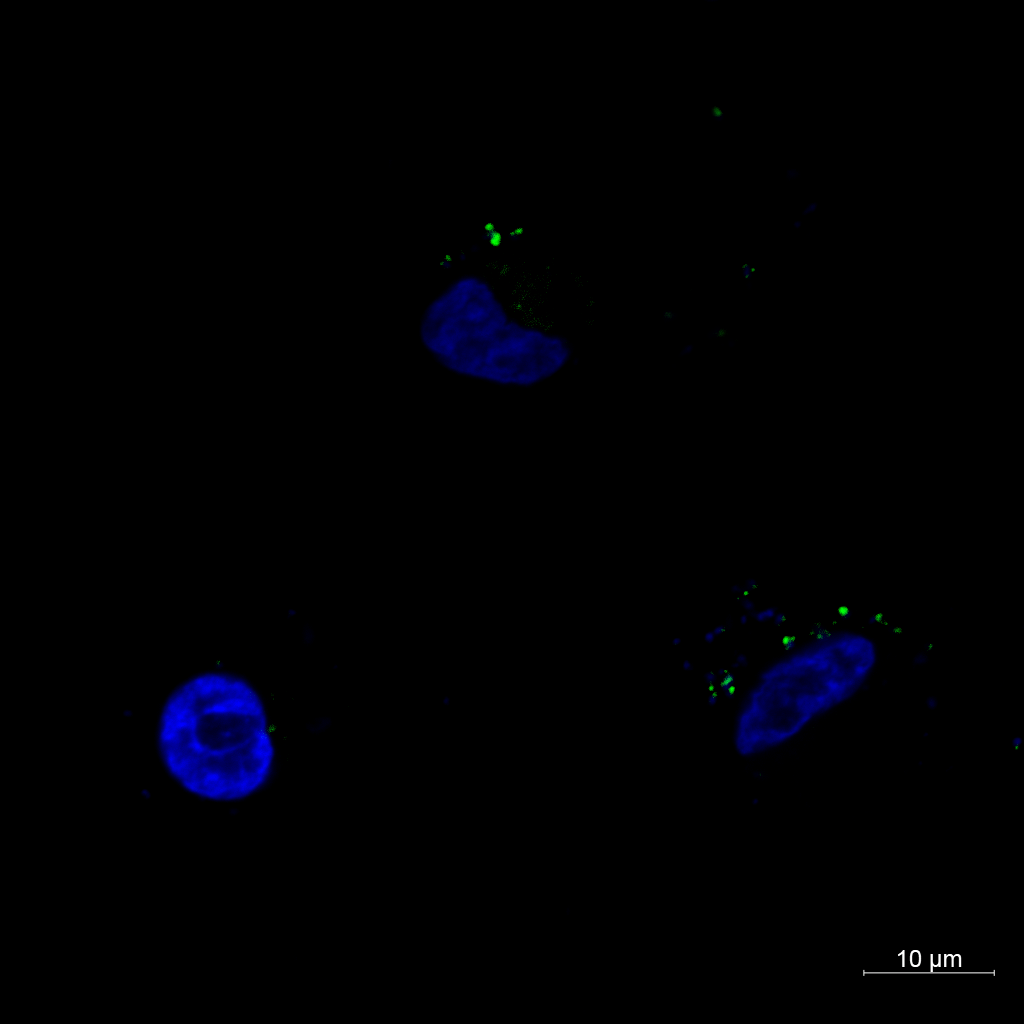

Supplement: Supplementary file 6 — Source data Fig. 3 [file 44318_2025_581_MOESM6_ESM.zip › Fig 3/3E/A549 pXF-FAM134C-2YN pRK-BMPR1a-2YC.tif]

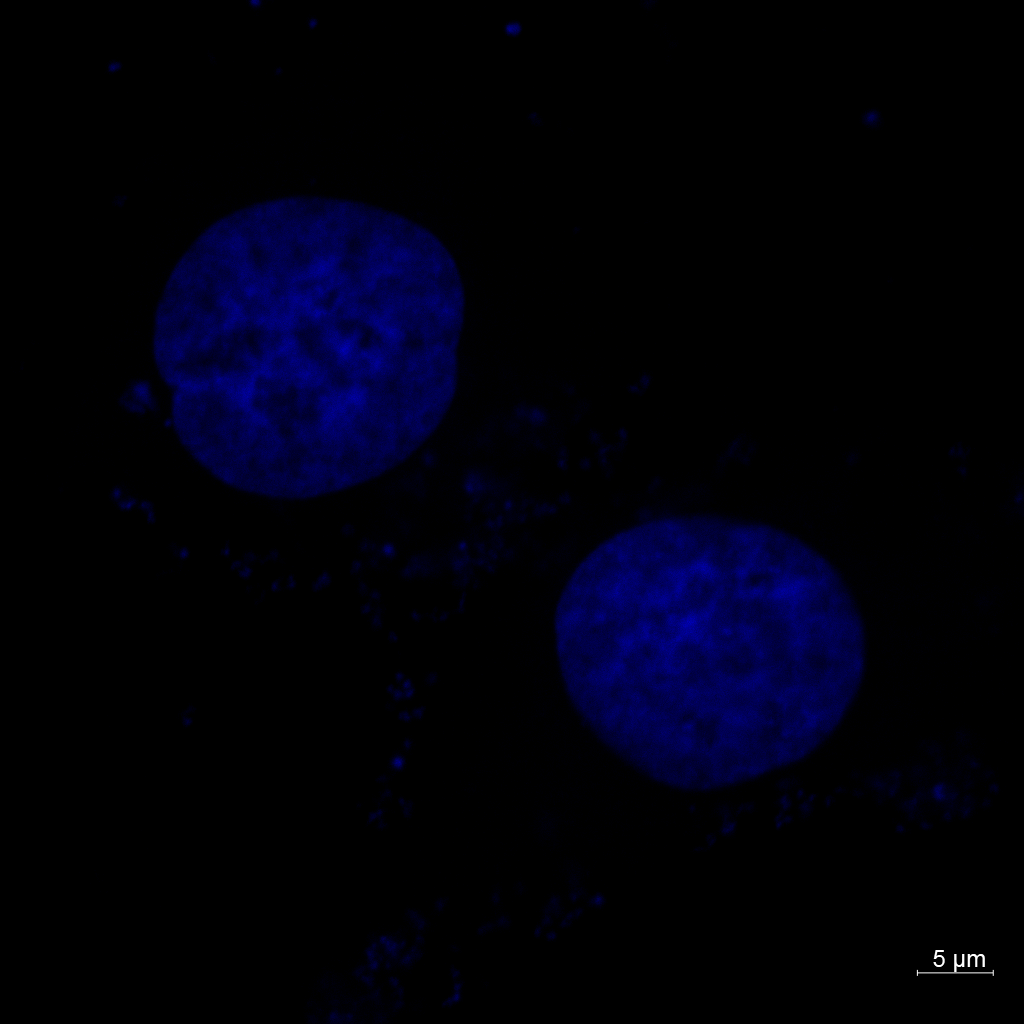

Supplement: Supplementary file 6 — Source data Fig. 3 [file 44318_2025_581_MOESM6_ESM.zip › Fig 3/3E/U2OS pxf-2YC pRK-BMPR1A-2YN.tif]

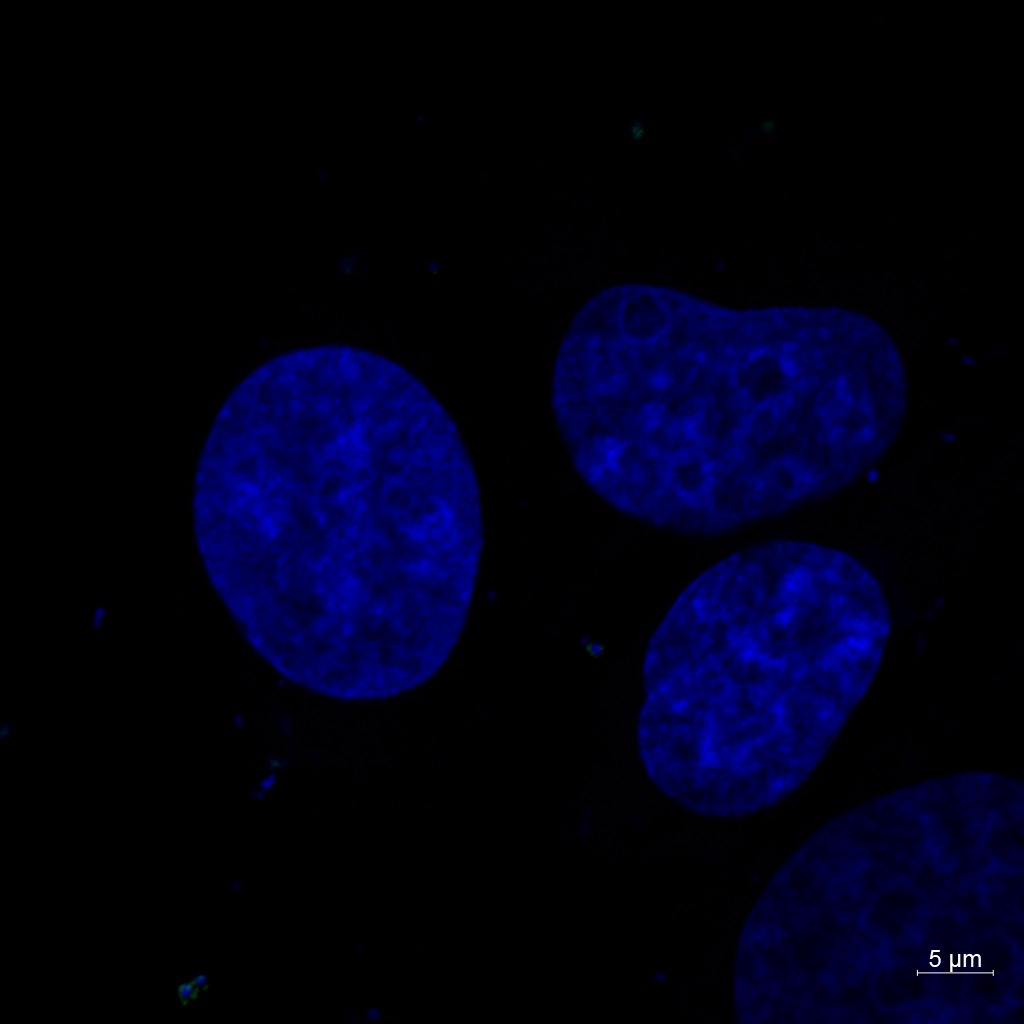

Supplement: Supplementary file 6 — Source data Fig. 3 [file 44318_2025_581_MOESM6_ESM.zip › Fig 3/3E/A549 pXF-2YN pRK-BMPR1a-2YC.tif]

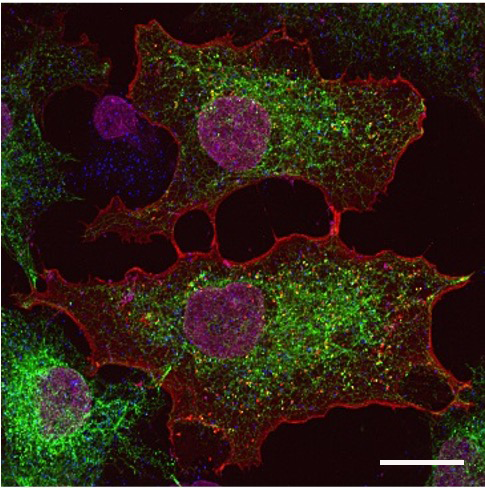

Supplement: Supplementary file 6 — Source data Fig. 3 [file 44318_2025_581_MOESM6_ESM.zip › Fig 3/3H/STV 0h Merged.tif]

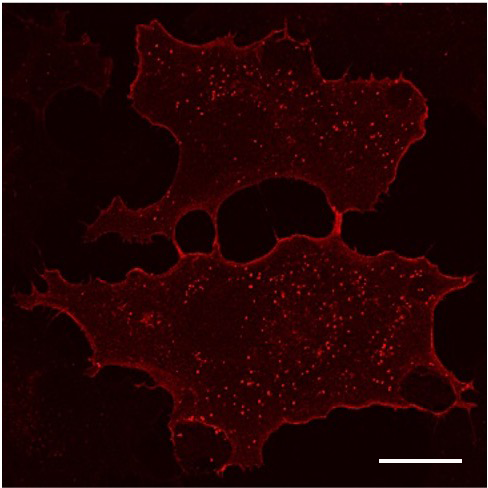

Supplement: Supplementary file 6 — Source data Fig. 3 [file 44318_2025_581_MOESM6_ESM.zip › Fig 3/3H/STV 0h BMPR1A-mcherry.tif]

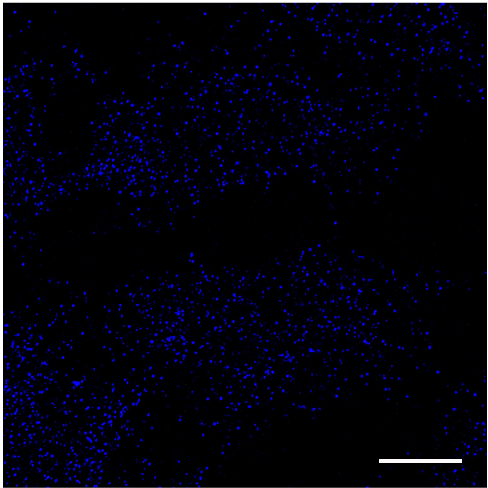

Supplement: Supplementary file 6 — Source data Fig. 3 [file 44318_2025_581_MOESM6_ESM.zip › Fig 3/3H/STV 0h LC3.tif]

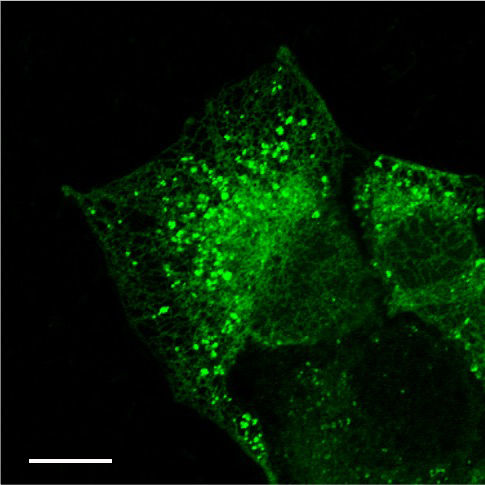

Supplement: Supplementary file 6 — Source data Fig. 3 [file 44318_2025_581_MOESM6_ESM.zip › Fig 3/3H/STV 2h FAM134C-GFP.tif]

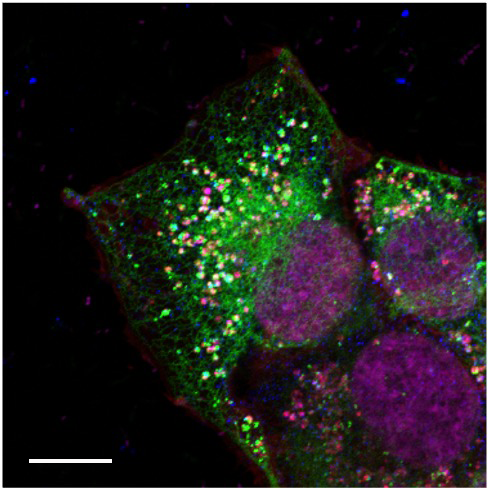

Supplement: Supplementary file 6 — Source data Fig. 3 [file 44318_2025_581_MOESM6_ESM.zip › Fig 3/3H/STV 2h Merged.tif]

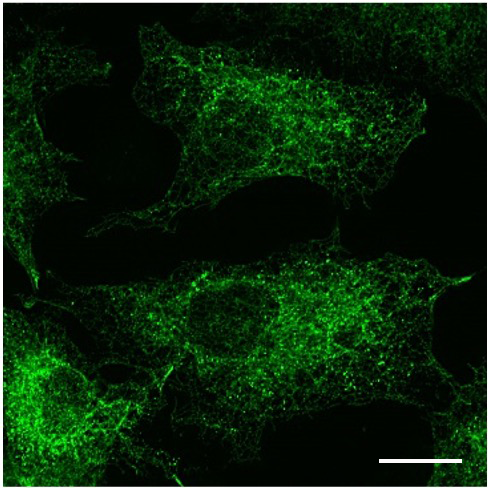

Supplement: Supplementary file 6 — Source data Fig. 3 [file 44318_2025_581_MOESM6_ESM.zip › Fig 3/3H/STV 0h FAM134C-GFP.tif]

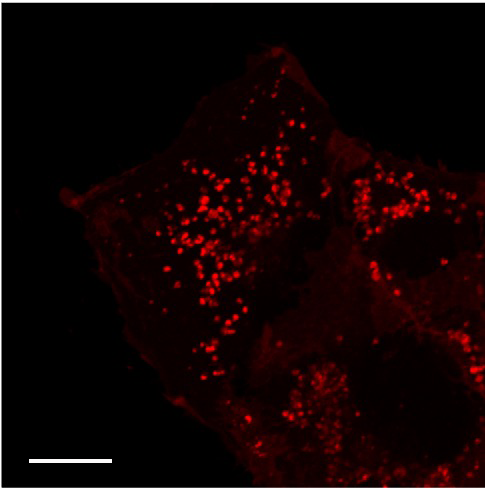

Supplement: Supplementary file 6 — Source data Fig. 3 [file 44318_2025_581_MOESM6_ESM.zip › Fig 3/3H/STV2h BMPR1A-mCherry.tif]

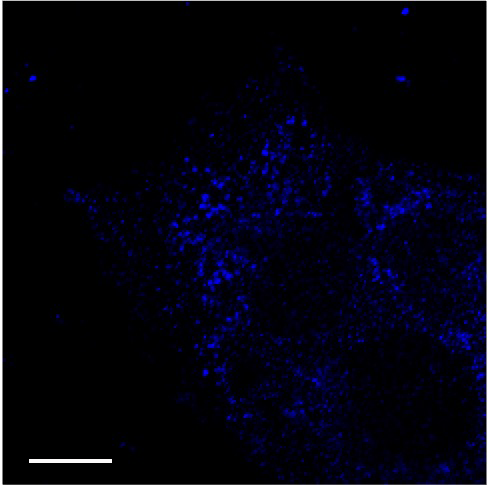

Supplement: Supplementary file 6 — Source data Fig. 3 [file 44318_2025_581_MOESM6_ESM.zip › Fig 3/3H/STV 2h LC3.tif]

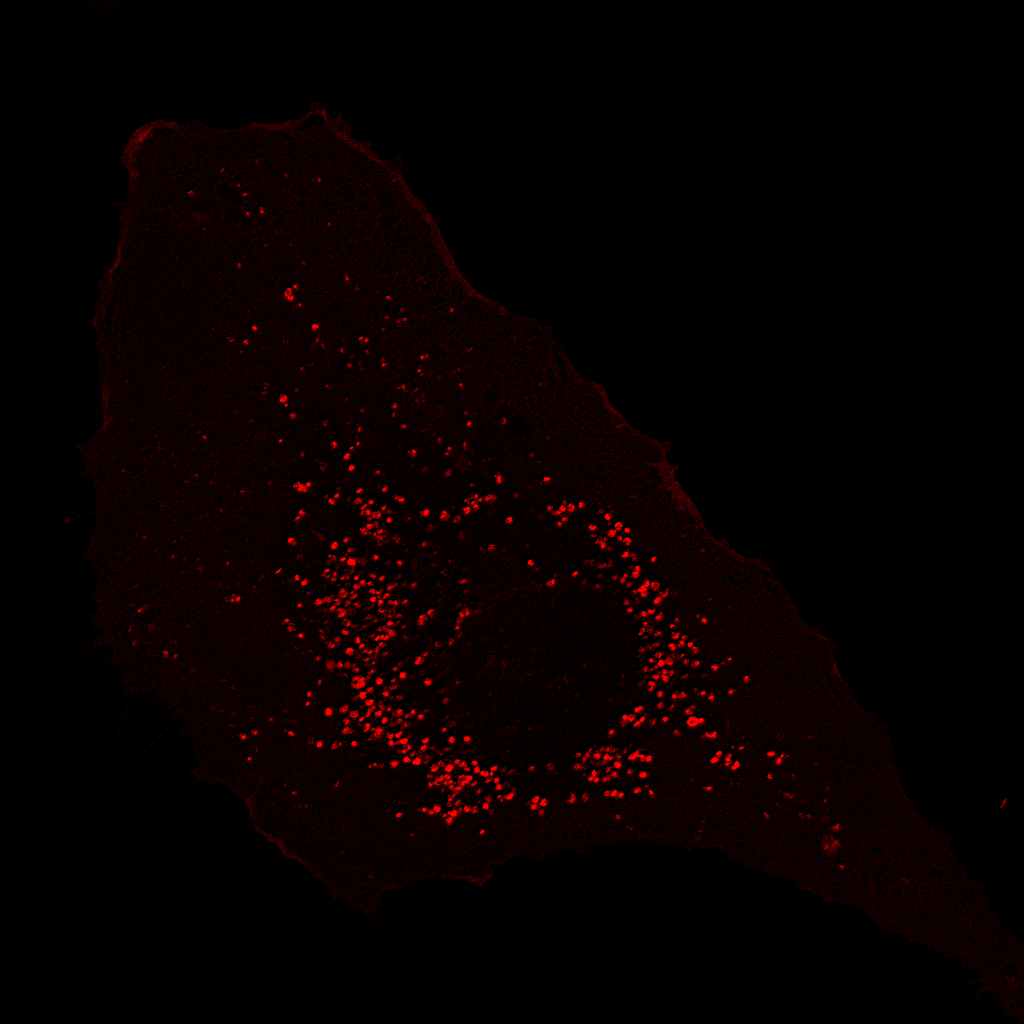

Supplement: Supplementary file 6 — Source data Fig. 3 [file 44318_2025_581_MOESM6_ESM.zip › Fig 3/3F/U2OS FAM134C KO BMPR1A-mCherry.tif]

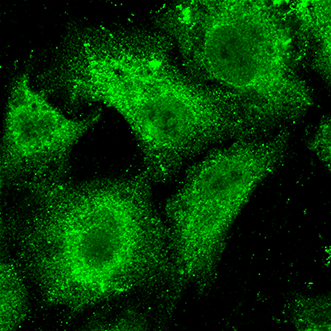

Supplement: Supplementary file 6 — Source data Fig. 3 [file 44318_2025_581_MOESM6_ESM.zip › Fig 3/3F/A549 FAM134C.tif]

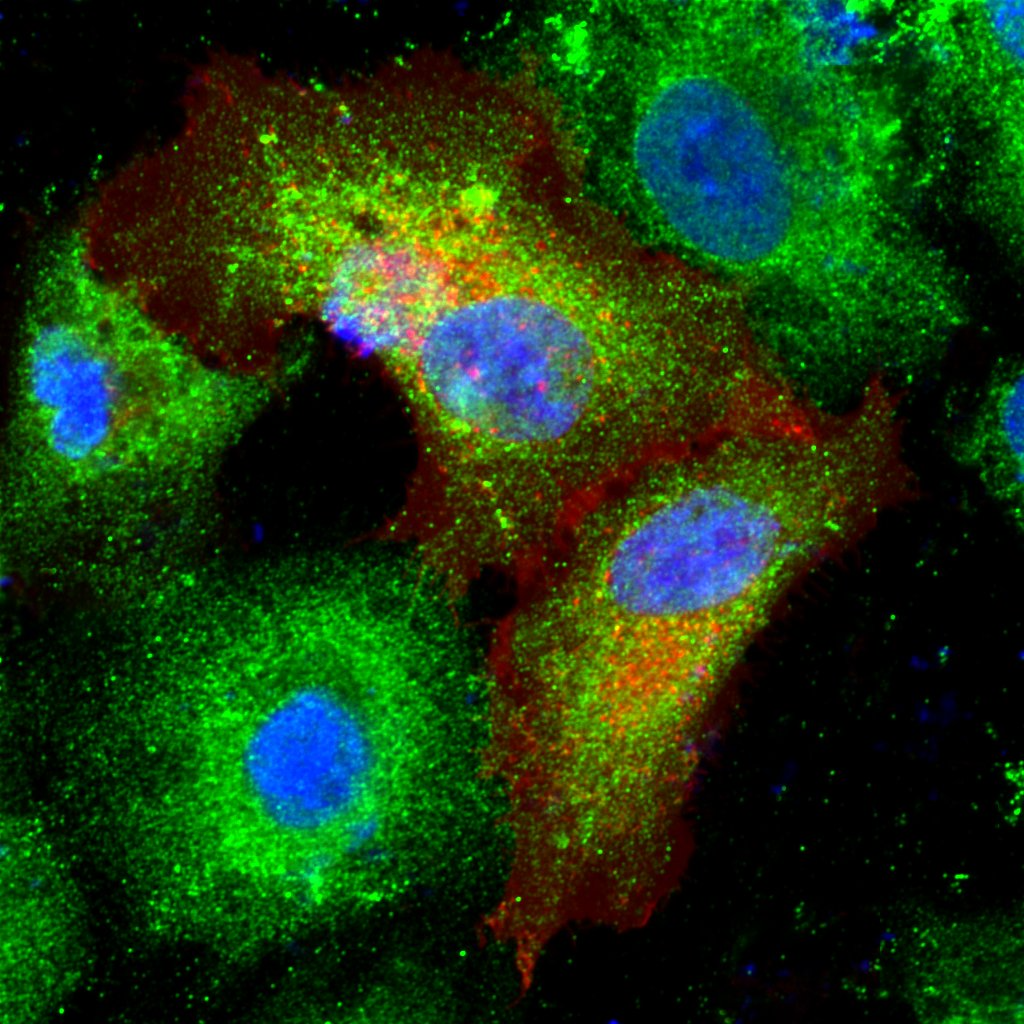

Supplement: Supplementary file 6 — Source data Fig. 3 [file 44318_2025_581_MOESM6_ESM.zip › Fig 3/3F/A549 Merged.tiff]

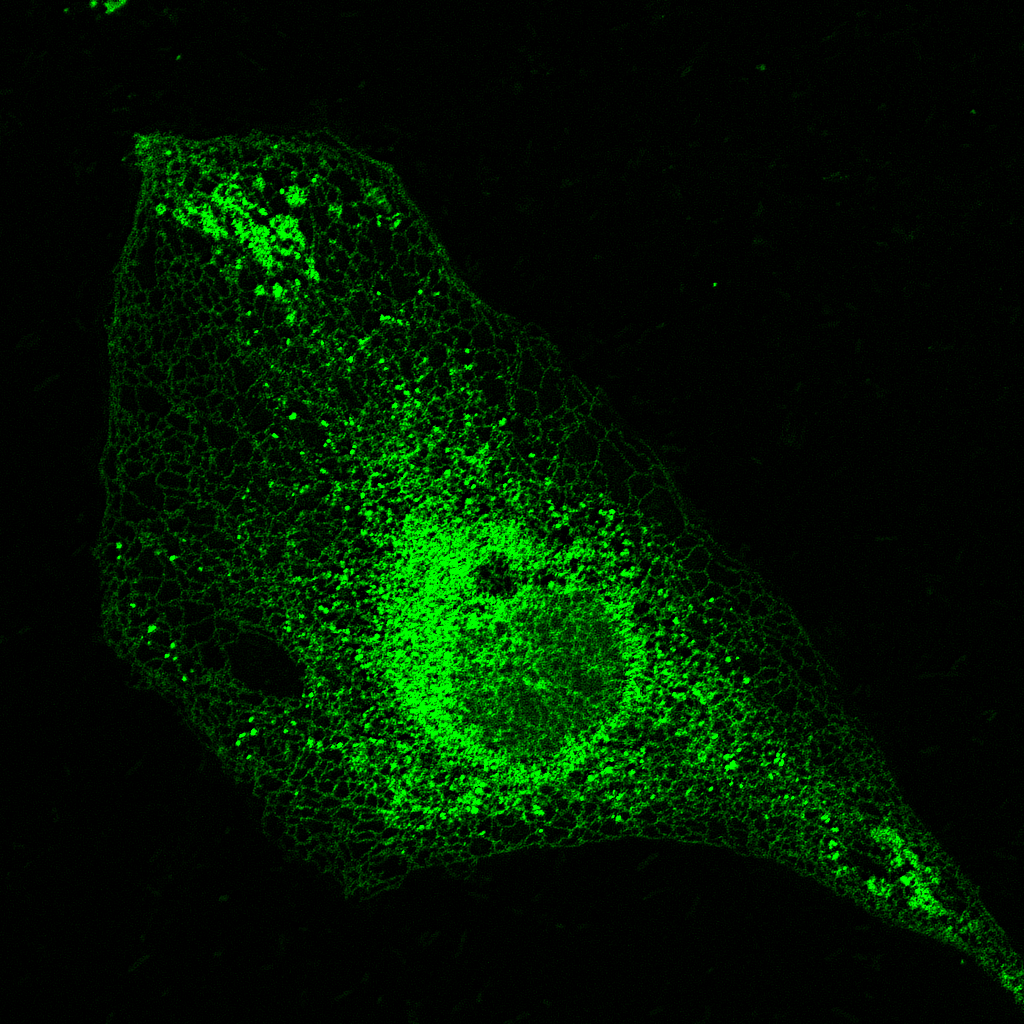

Supplement: Supplementary file 6 — Source data Fig. 3 [file 44318_2025_581_MOESM6_ESM.zip › Fig 3/3F/U2OS FAM134C KO FAM134C-GFP.tif]

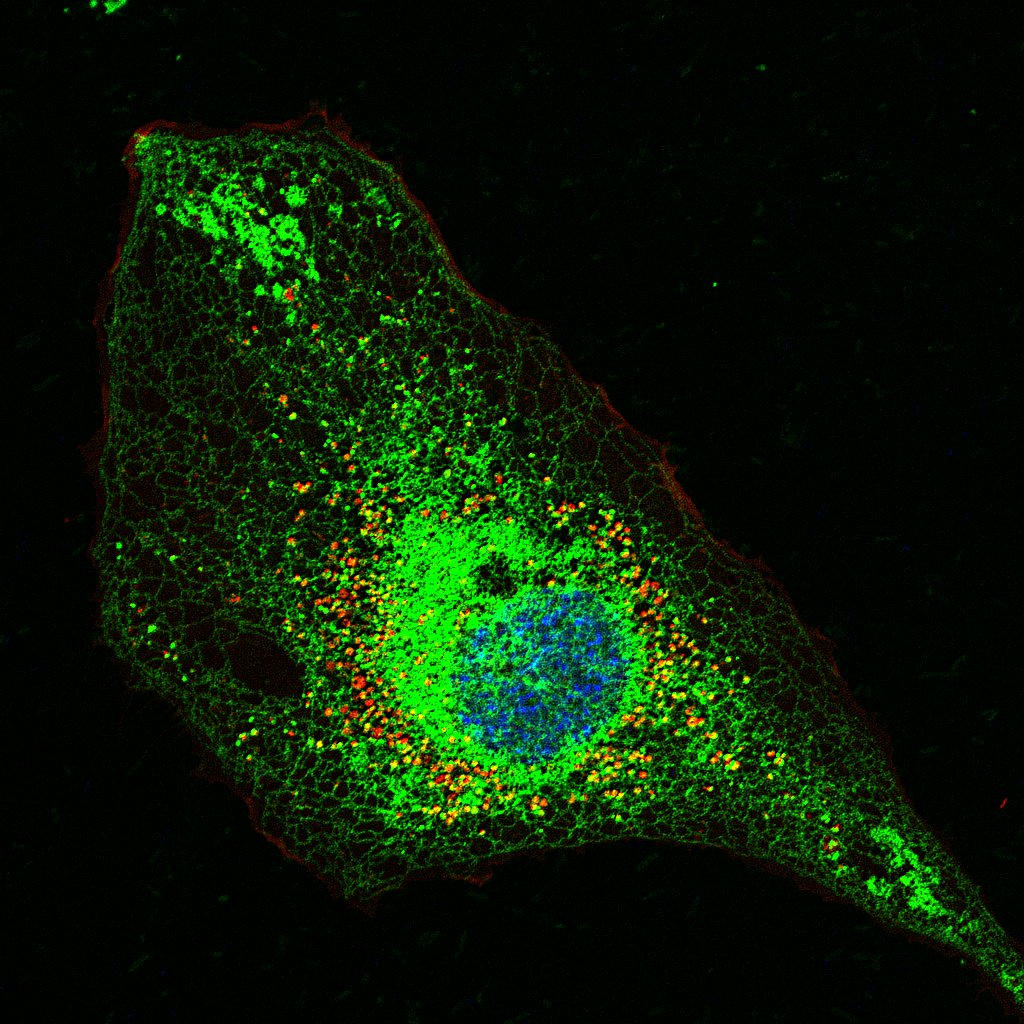

Supplement: Supplementary file 6 — Source data Fig. 3 [file 44318_2025_581_MOESM6_ESM.zip › Fig 3/3F/U2OS FAM134C KO merged.tiff]

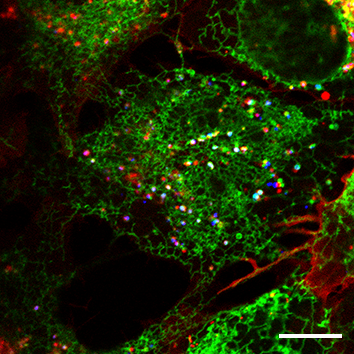

Supplement: Supplementary file 6 — Source data Fig. 3 [file 44318_2025_581_MOESM6_ESM.zip › Fig 3/3G/STV 0h Merged.tif]

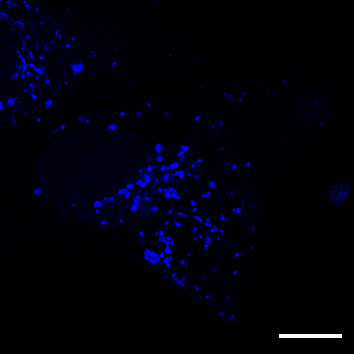

Supplement: Supplementary file 6 — Source data Fig. 3 [file 44318_2025_581_MOESM6_ESM.zip › Fig 3/3G/STV 2h lysotracker.tif]

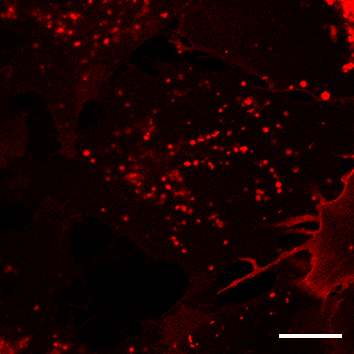

Supplement: Supplementary file 6 — Source data Fig. 3 [file 44318_2025_581_MOESM6_ESM.zip › Fig 3/3G/STV 0h BMPR1A-mCherry.tif]

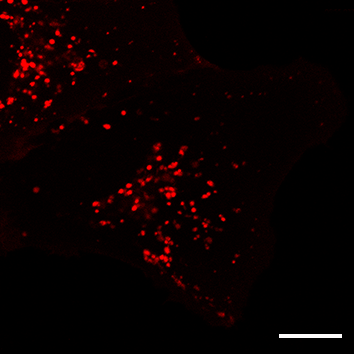

Supplement: Supplementary file 6 — Source data Fig. 3 [file 44318_2025_581_MOESM6_ESM.zip › Fig 3/3G/STV 2h BMPR1A-mCherry.tif]

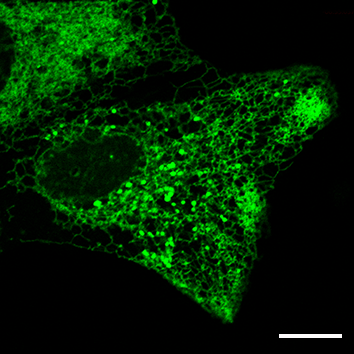

Supplement: Supplementary file 6 — Source data Fig. 3 [file 44318_2025_581_MOESM6_ESM.zip › Fig 3/3G/STV 2h FAM134C-GFP.tif]

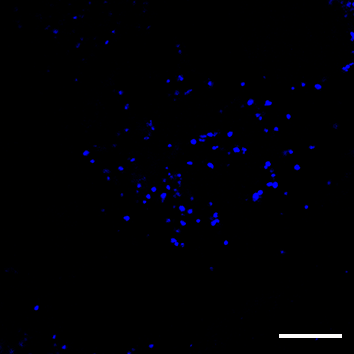

Supplement: Supplementary file 6 — Source data Fig. 3 [file 44318_2025_581_MOESM6_ESM.zip › Fig 3/3G/STV 0h lysotracker.tif]

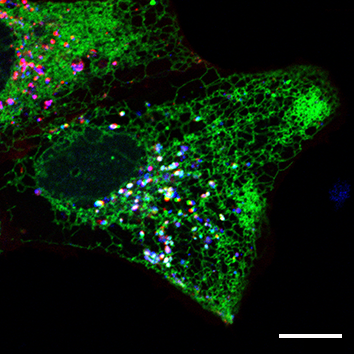

Supplement: Supplementary file 6 — Source data Fig. 3 [file 44318_2025_581_MOESM6_ESM.zip › Fig 3/3G/STV 2h Merged.tif]

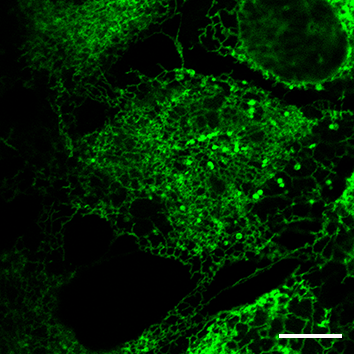

Supplement: Supplementary file 6 — Source data Fig. 3 [file 44318_2025_581_MOESM6_ESM.zip › Fig 3/3G/STV 0h FAM134C-GFP.tif]

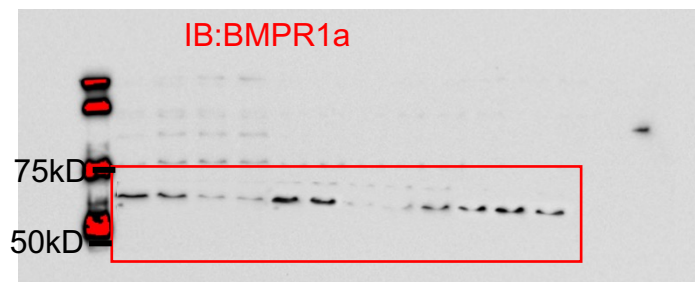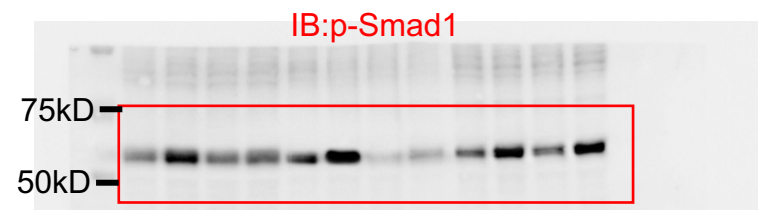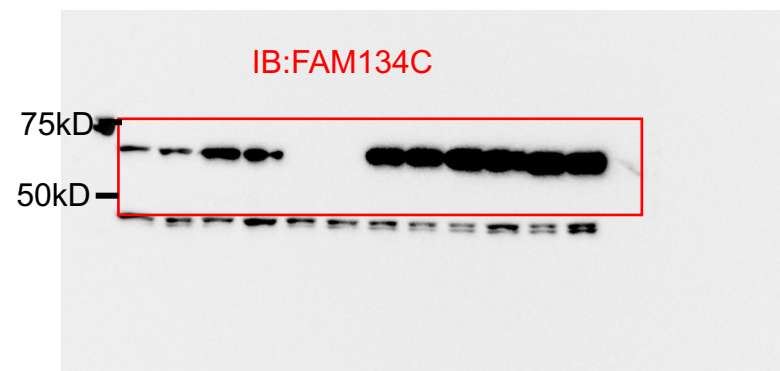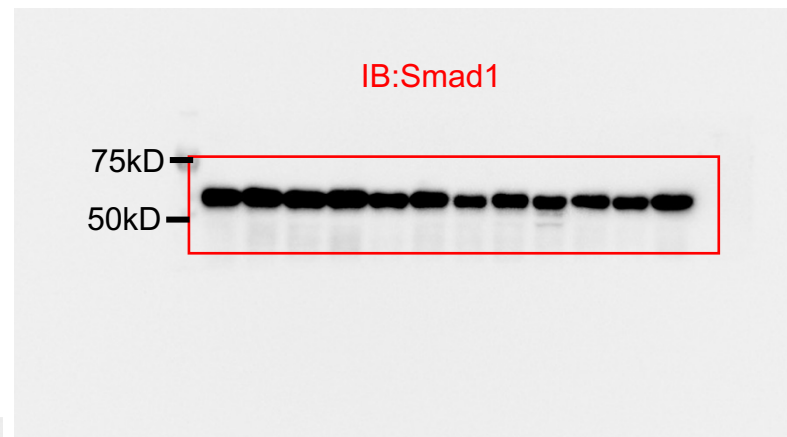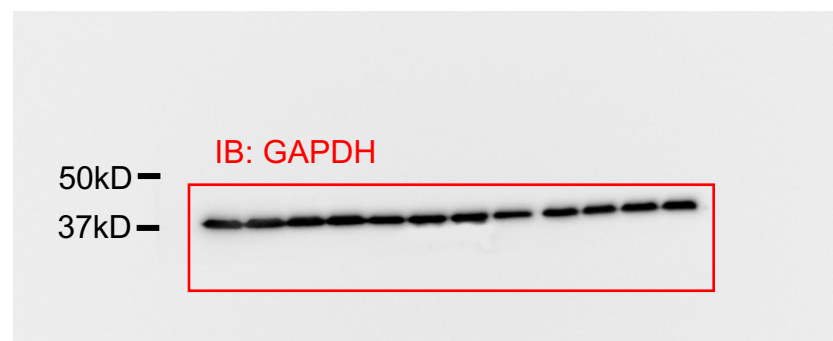

Supplement: Supplementary file 7 — Source data Fig. 4 [file 44318_2025_581_MOESM7_ESM.zip › Fig 4/4F.pdf]

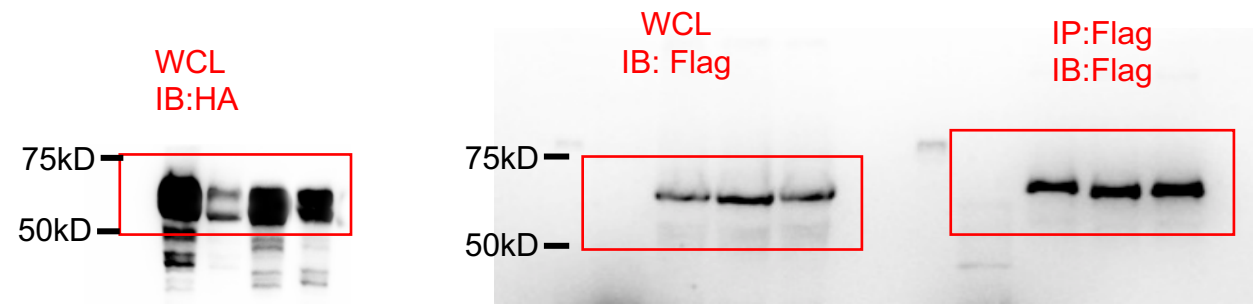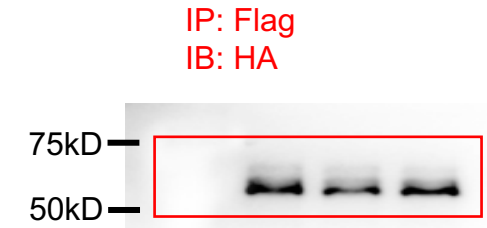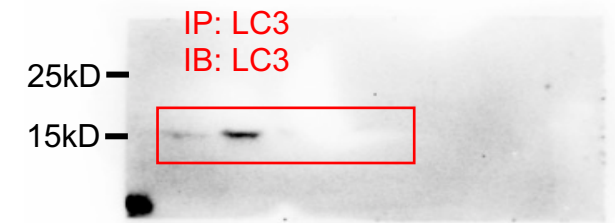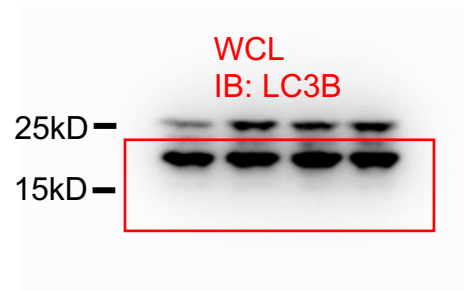

Supplement: Supplementary file 7 — Source data Fig. 4 [file 44318_2025_581_MOESM7_ESM.zip › Fig 4/4D.pdf]

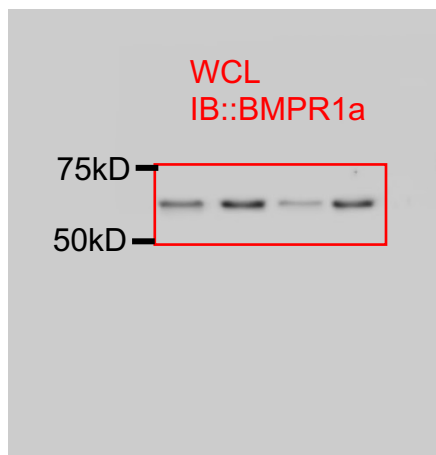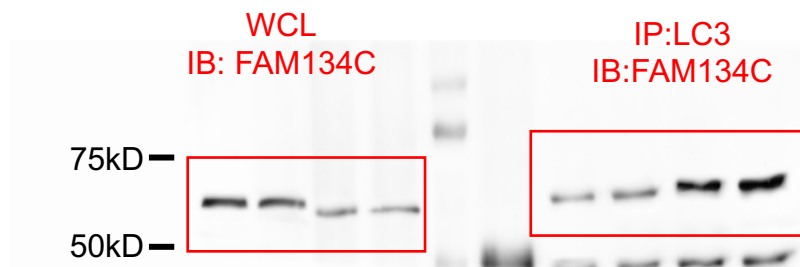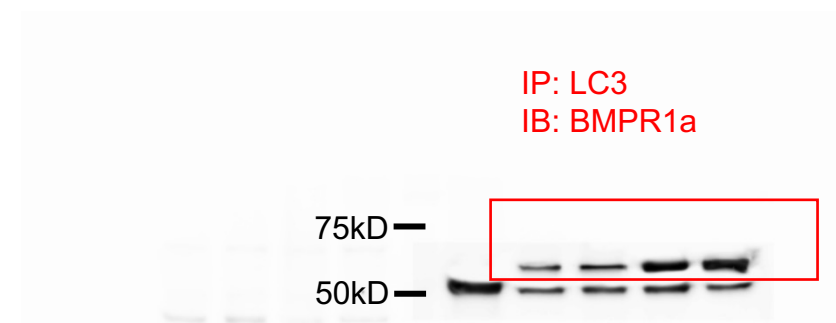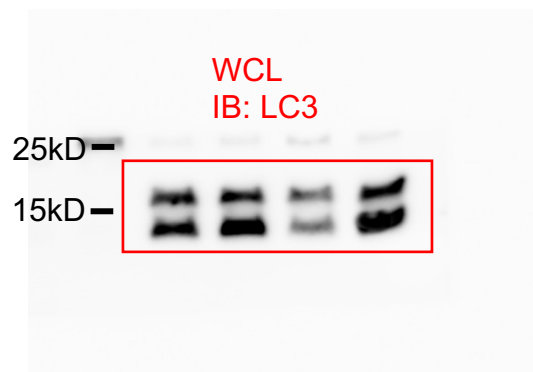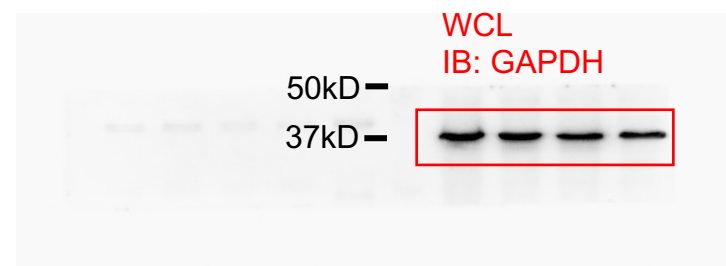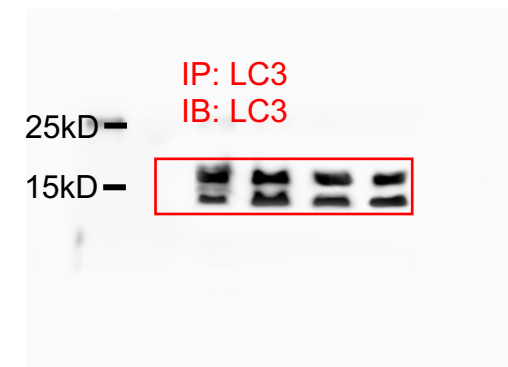

Supplement: Supplementary file 7 — Source data Fig. 4 [file 44318_2025_581_MOESM7_ESM.zip › Fig 4/4C.pdf]

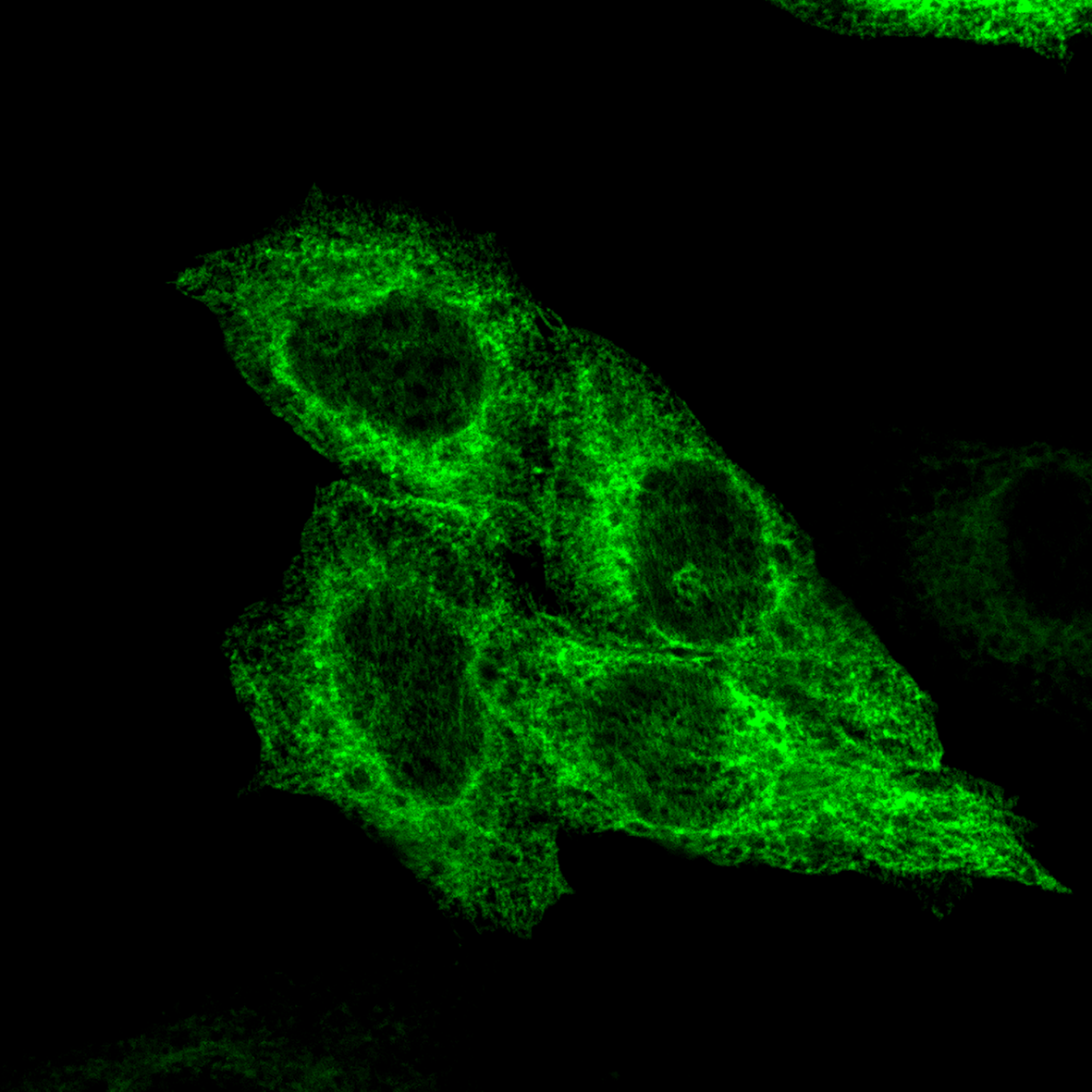

Supplement: Supplementary file 7 — Source data Fig. 4 [file 44318_2025_581_MOESM7_ESM.zip › Fig 4/4E/FAM134C-Flag Flag-488 STV 0h.tif]

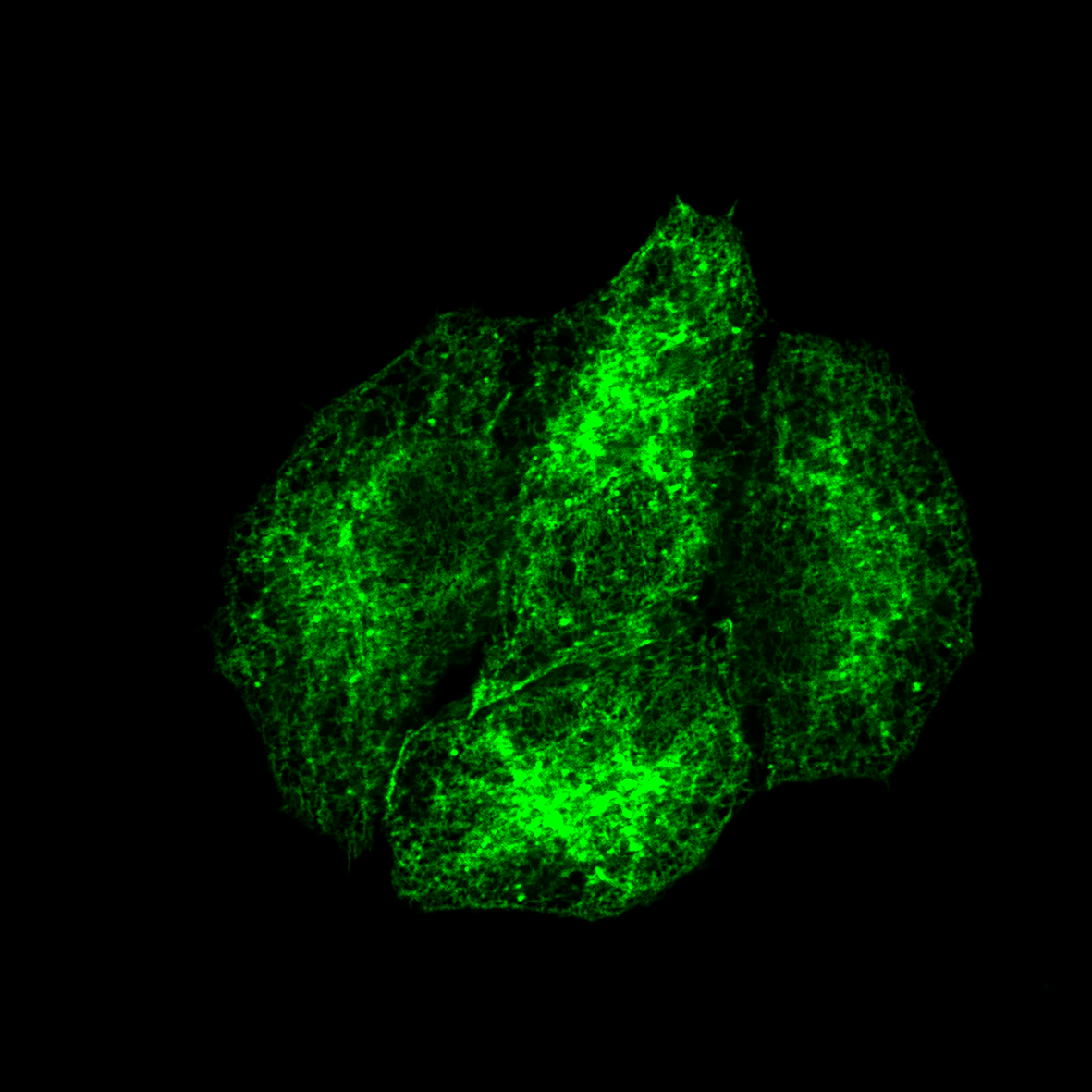

Supplement: Supplementary file 7 — Source data Fig. 4 [file 44318_2025_581_MOESM7_ESM.zip › Fig 4/4E/FAM134C-Flag Flag-488 STV 1h.tif]

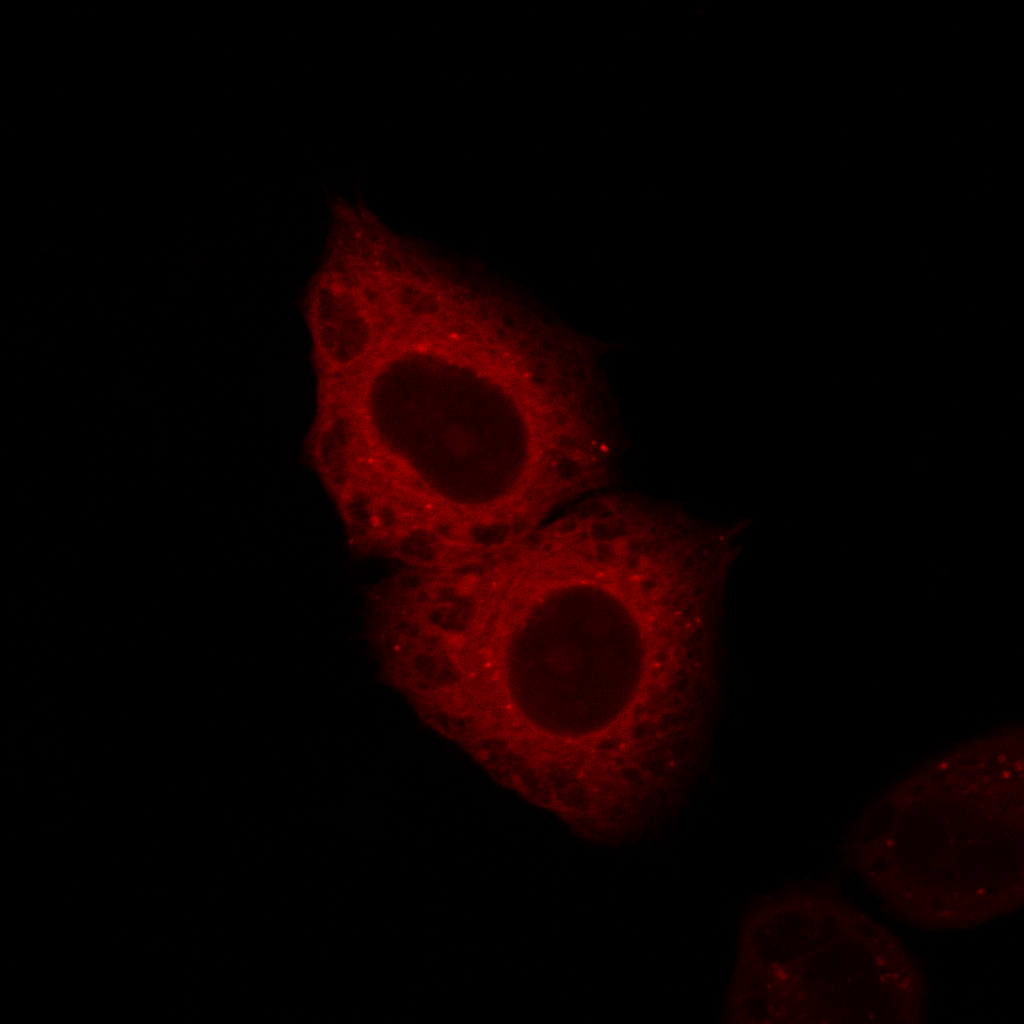

Supplement: Supplementary file 7 — Source data Fig. 4 [file 44318_2025_581_MOESM7_ESM.zip › Fig 4/4E/FAM134C-M-Flag LC3-mcherry STV 0h.tif]

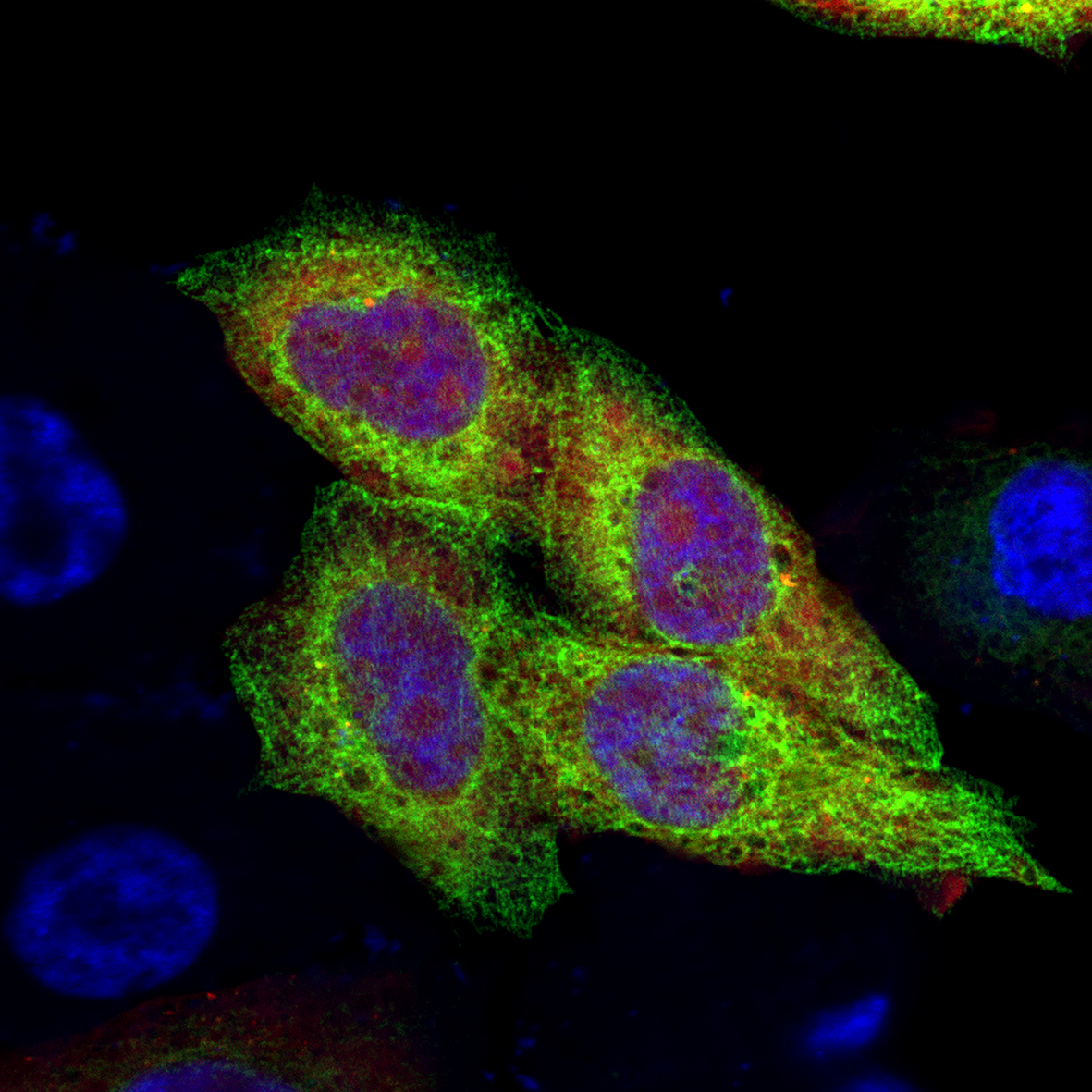

Supplement: Supplementary file 7 — Source data Fig. 4 [file 44318_2025_581_MOESM7_ESM.zip › Fig 4/4E/FAM134C-Flag merged STV 0h.tif]

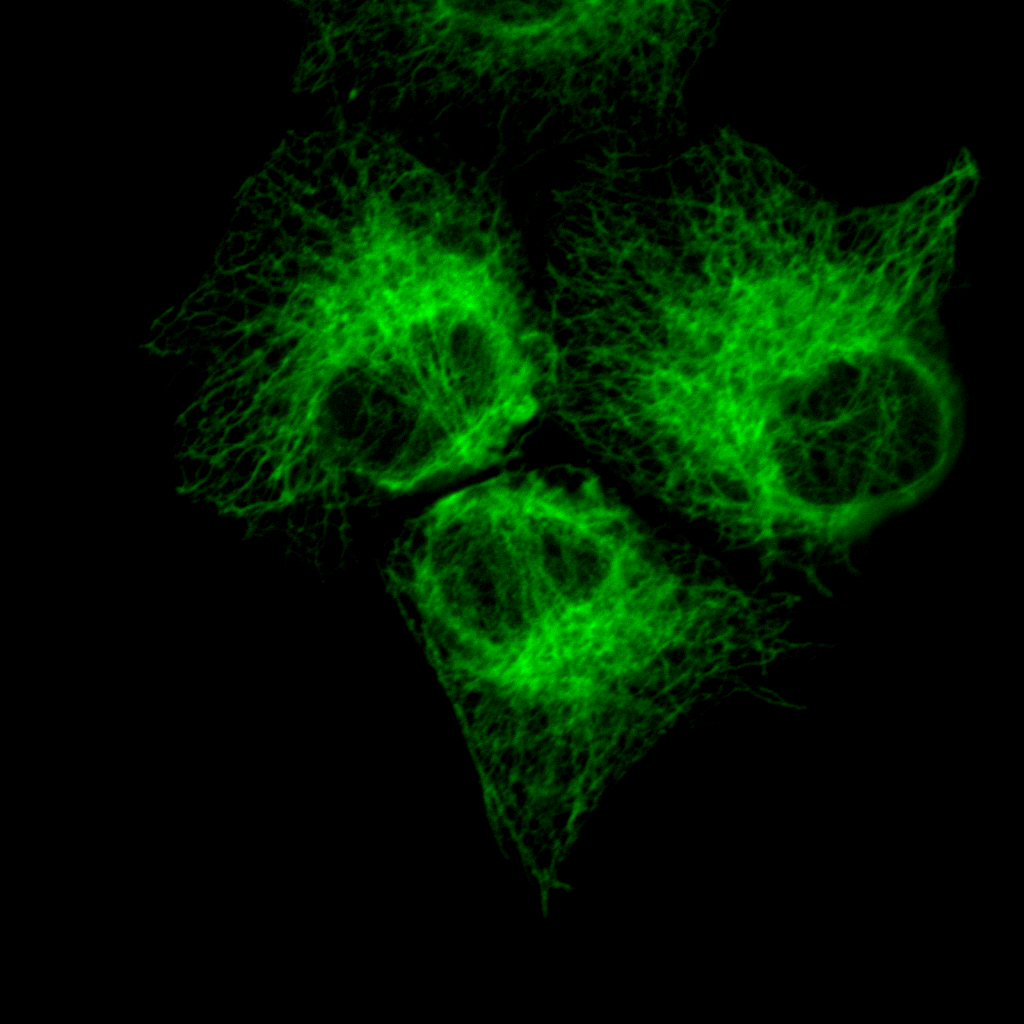

Supplement: Supplementary file 7 — Source data Fig. 4 [file 44318_2025_581_MOESM7_ESM.zip › Fig 4/4E/FAM134C-D-Flag Flag-488 STV 0h.tif]

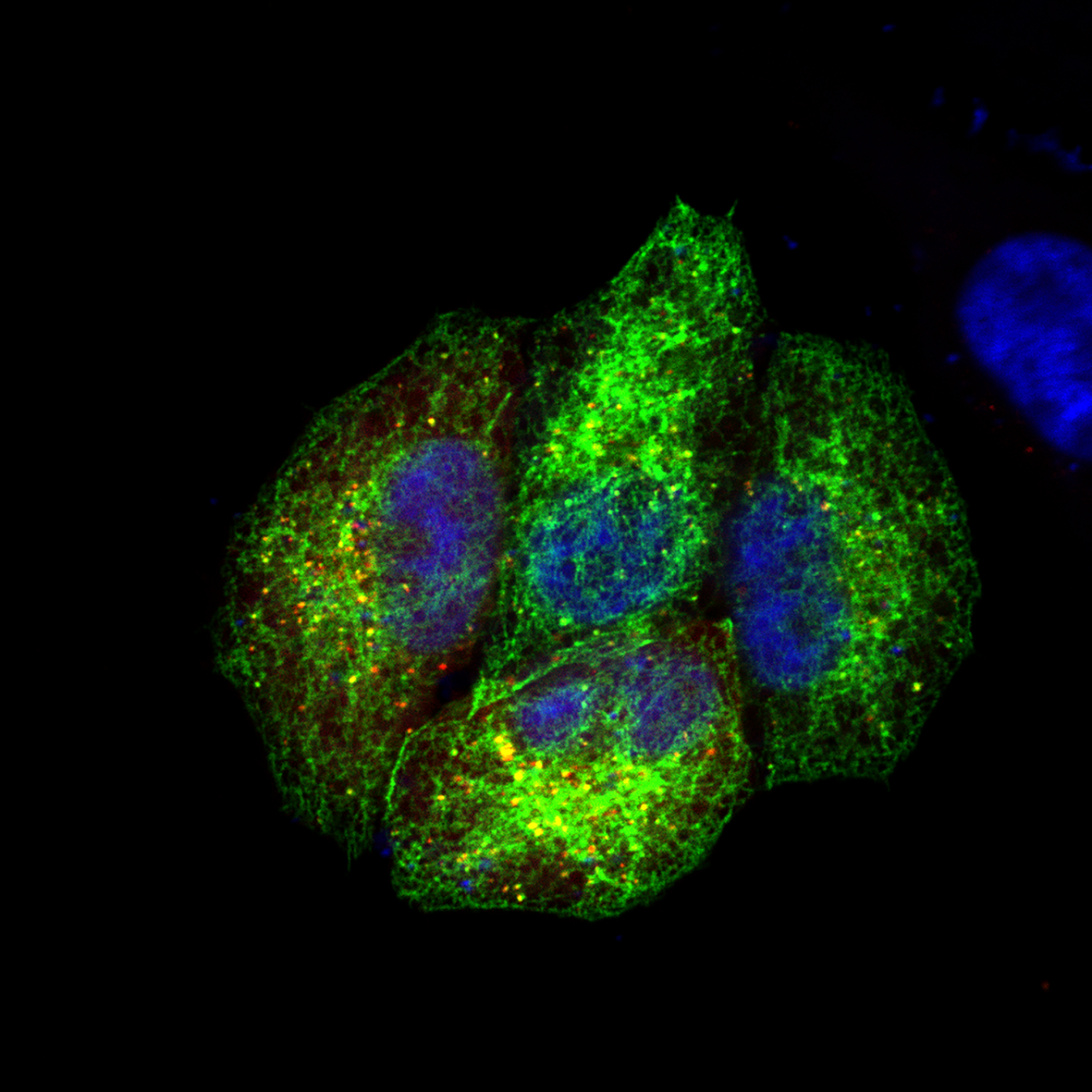

Supplement: Supplementary file 7 — Source data Fig. 4 [file 44318_2025_581_MOESM7_ESM.zip › Fig 4/4E/FAM134C-Flag merged STV 1h.tif]

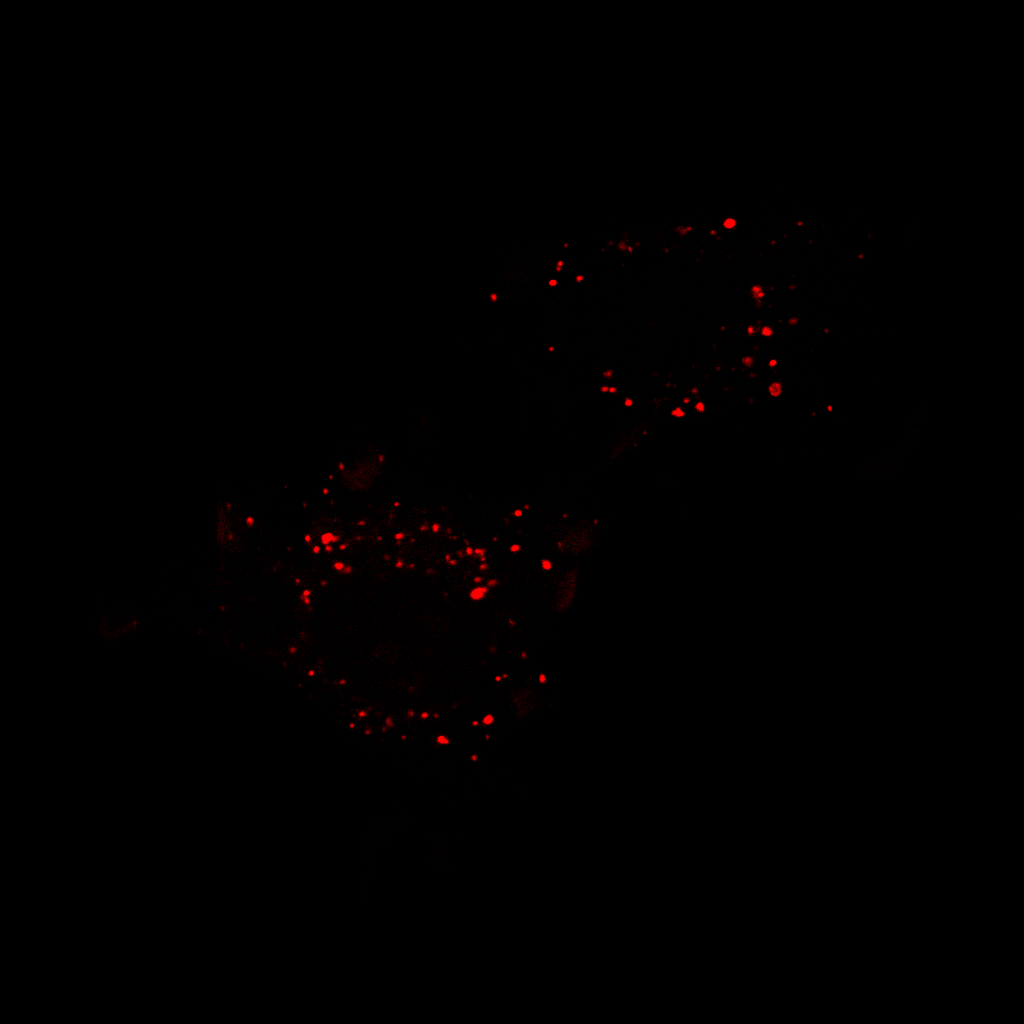

Supplement: Supplementary file 7 — Source data Fig. 4 [file 44318_2025_581_MOESM7_ESM.zip › Fig 4/4E/FAM134C-M-Flag LC3-mcherry STV 1h.tif]

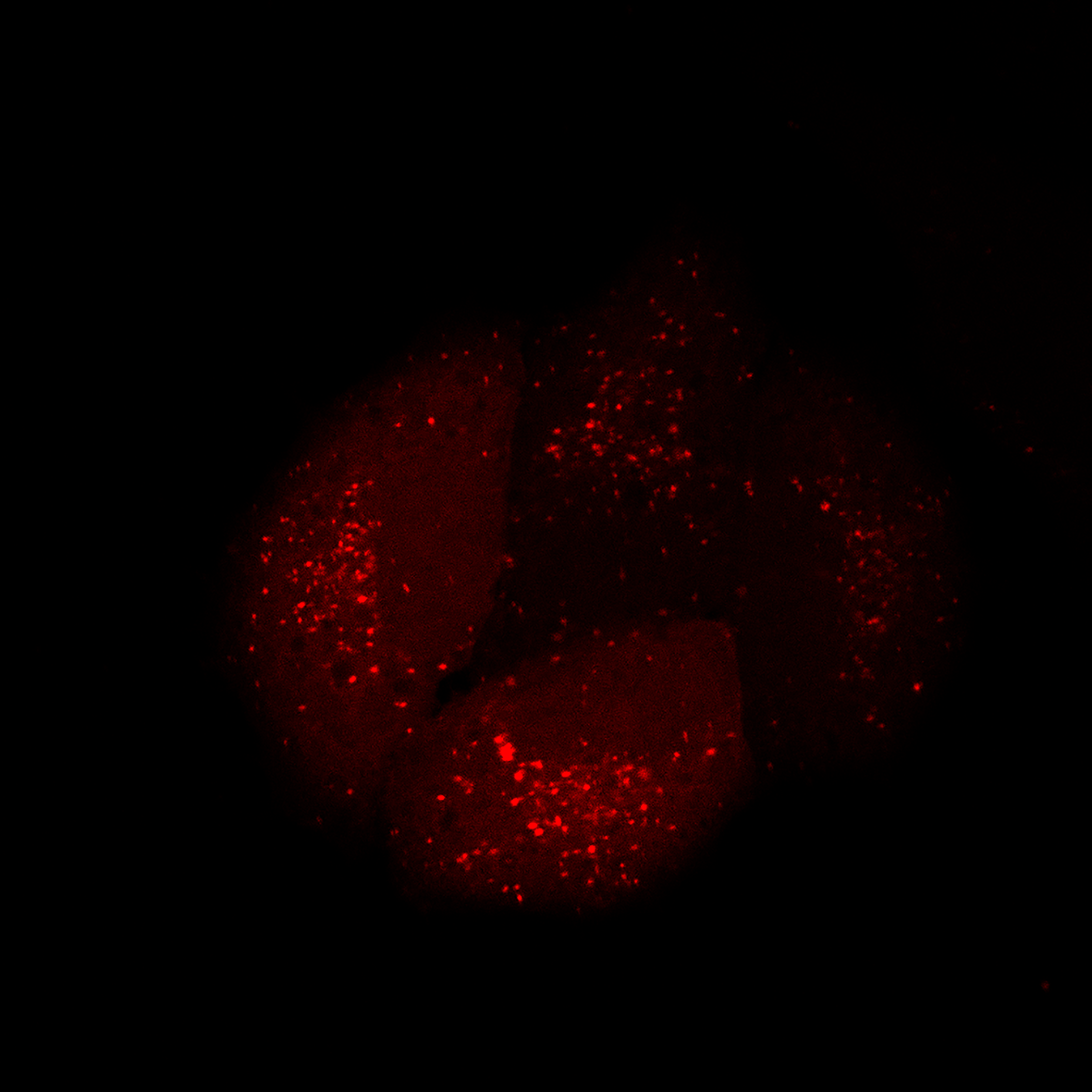

Supplement: Supplementary file 7 — Source data Fig. 4 [file 44318_2025_581_MOESM7_ESM.zip › Fig 4/4E/FAM134C-Flag LC3-mcherry STV 1h.tif]

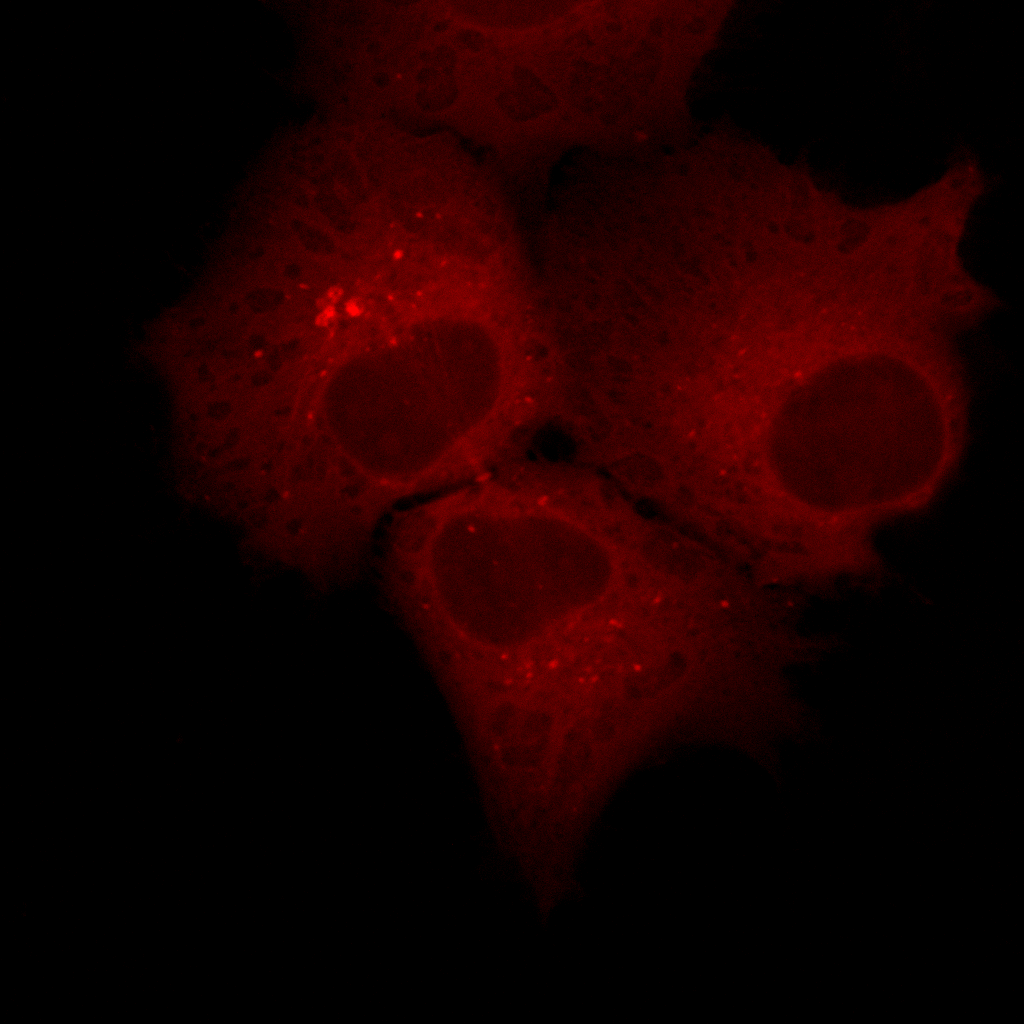

Supplement: Supplementary file 7 — Source data Fig. 4 [file 44318_2025_581_MOESM7_ESM.zip › Fig 4/4E/FAM134C-D-Flag LC3-mcherry STV 0h.tif]

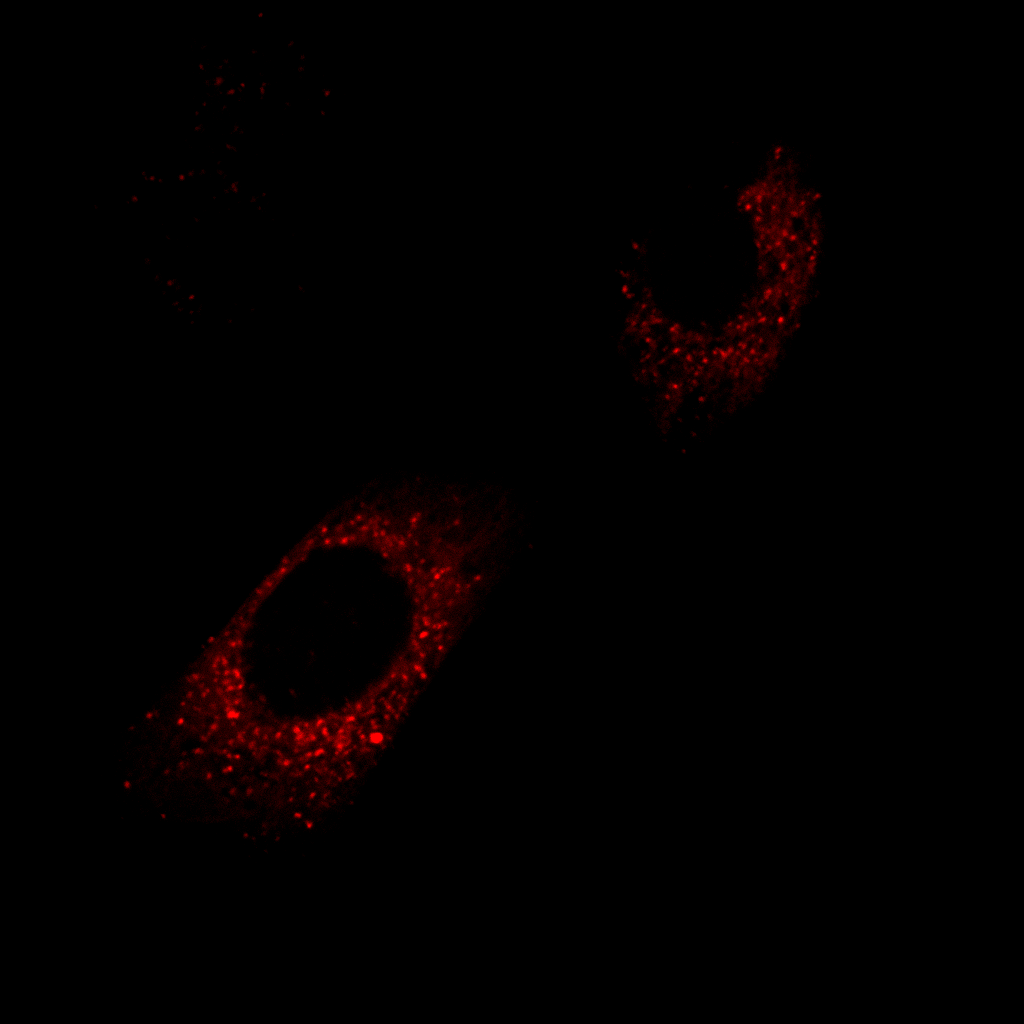

Supplement: Supplementary file 7 — Source data Fig. 4 [file 44318_2025_581_MOESM7_ESM.zip › Fig 4/4E/FAM134C-D-Flag LC3-mcherry STV 1h.tif]

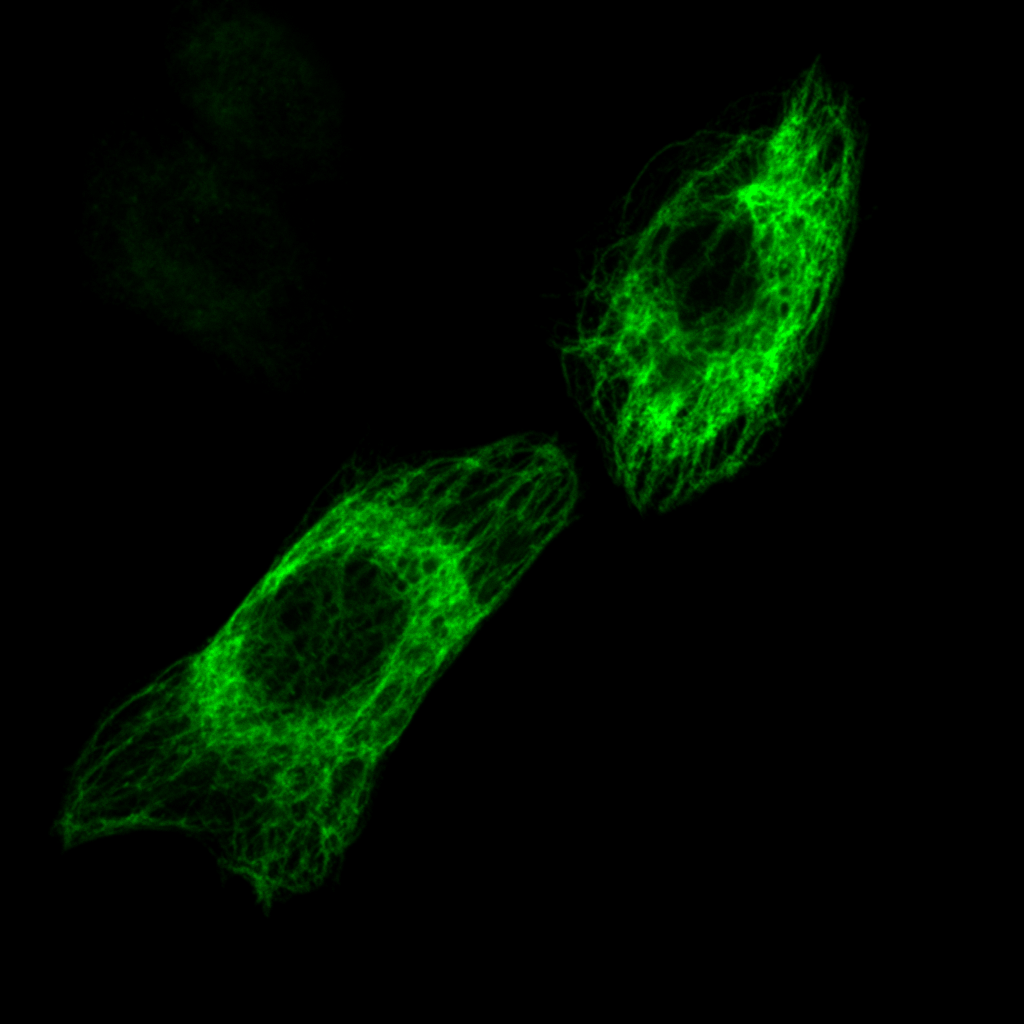

Supplement: Supplementary file 7 — Source data Fig. 4 [file 44318_2025_581_MOESM7_ESM.zip › Fig 4/4E/FAM134C-D-Flag Flag-488 STV 1h.tif]

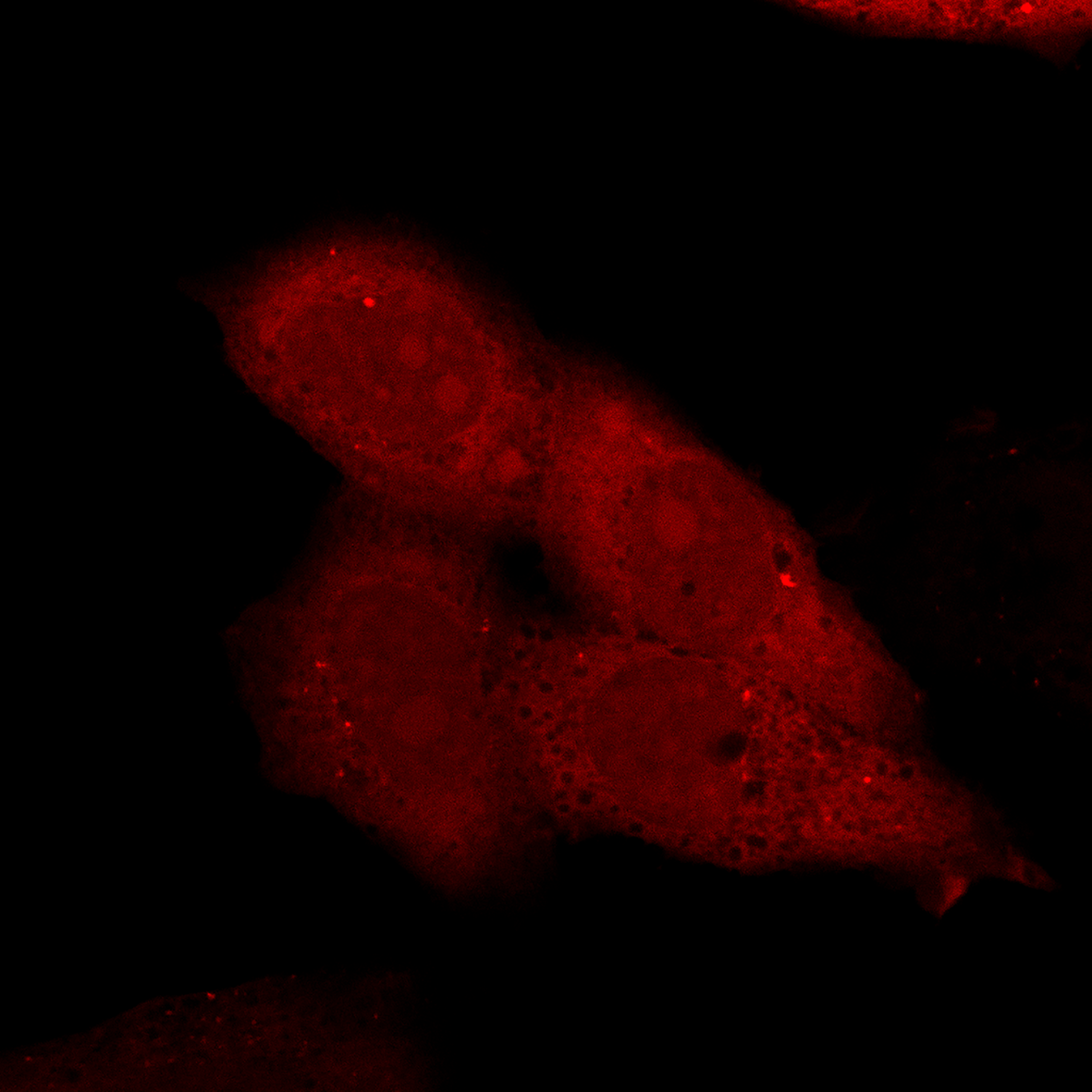

Supplement: Supplementary file 7 — Source data Fig. 4 [file 44318_2025_581_MOESM7_ESM.zip › Fig 4/4E/FAM134C-Flag LC3-mcherry STV 0h.tif]

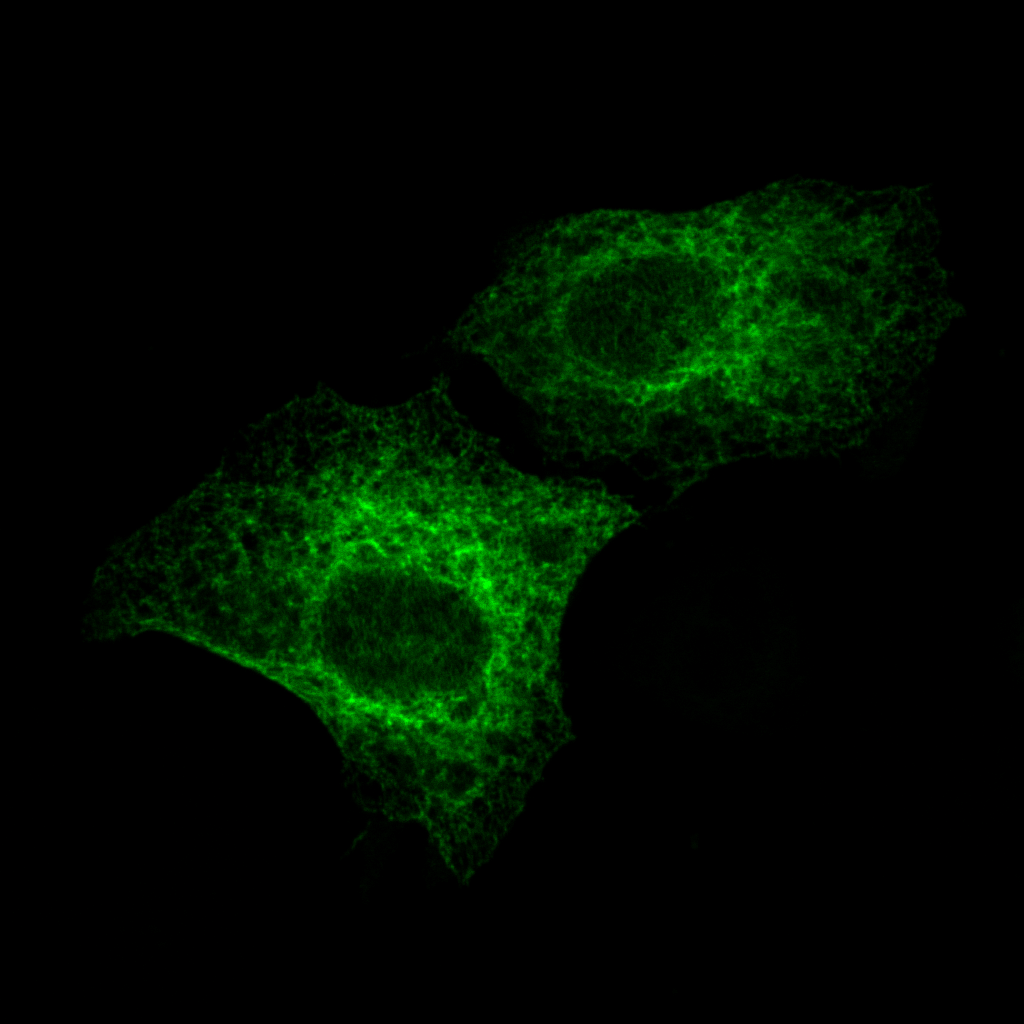

Supplement: Supplementary file 7 — Source data Fig. 4 [file 44318_2025_581_MOESM7_ESM.zip › Fig 4/4E/FAM134C-M-Flag Flag-488 STV 1h.tif]

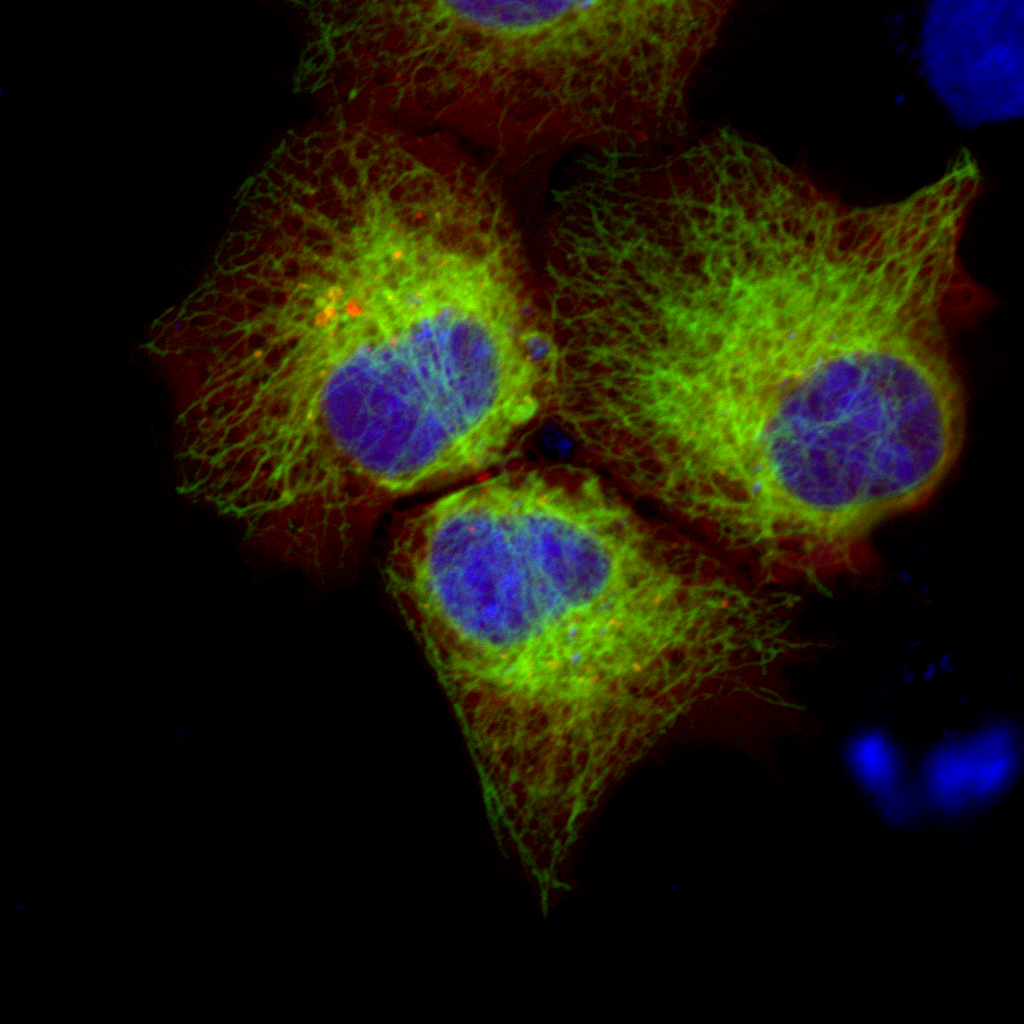

Supplement: Supplementary file 7 — Source data Fig. 4 [file 44318_2025_581_MOESM7_ESM.zip › Fig 4/4E/FAM123C-D-Flag merged STV 0htif.tif]

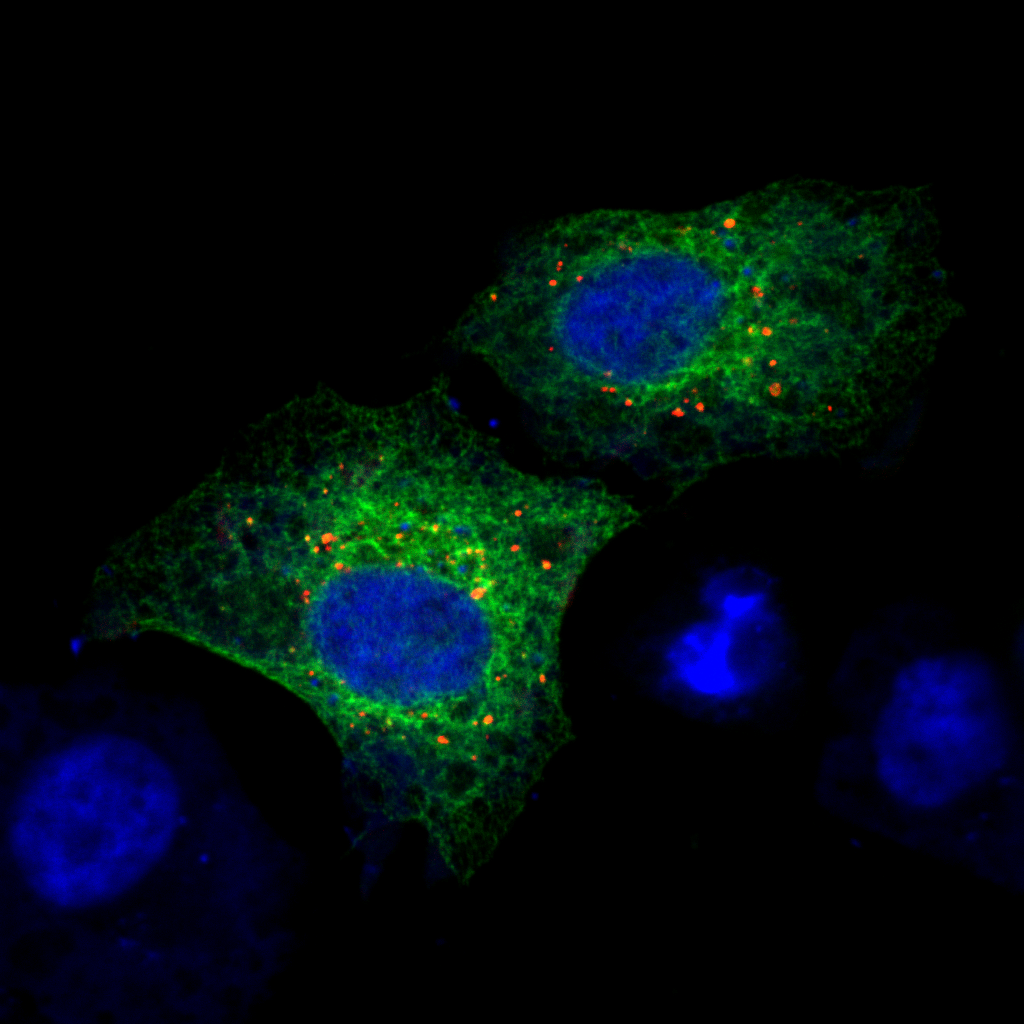

Supplement: Supplementary file 7 — Source data Fig. 4 [file 44318_2025_581_MOESM7_ESM.zip › Fig 4/4E/FAM134C-M-Flag Merged STV 1h.tif]

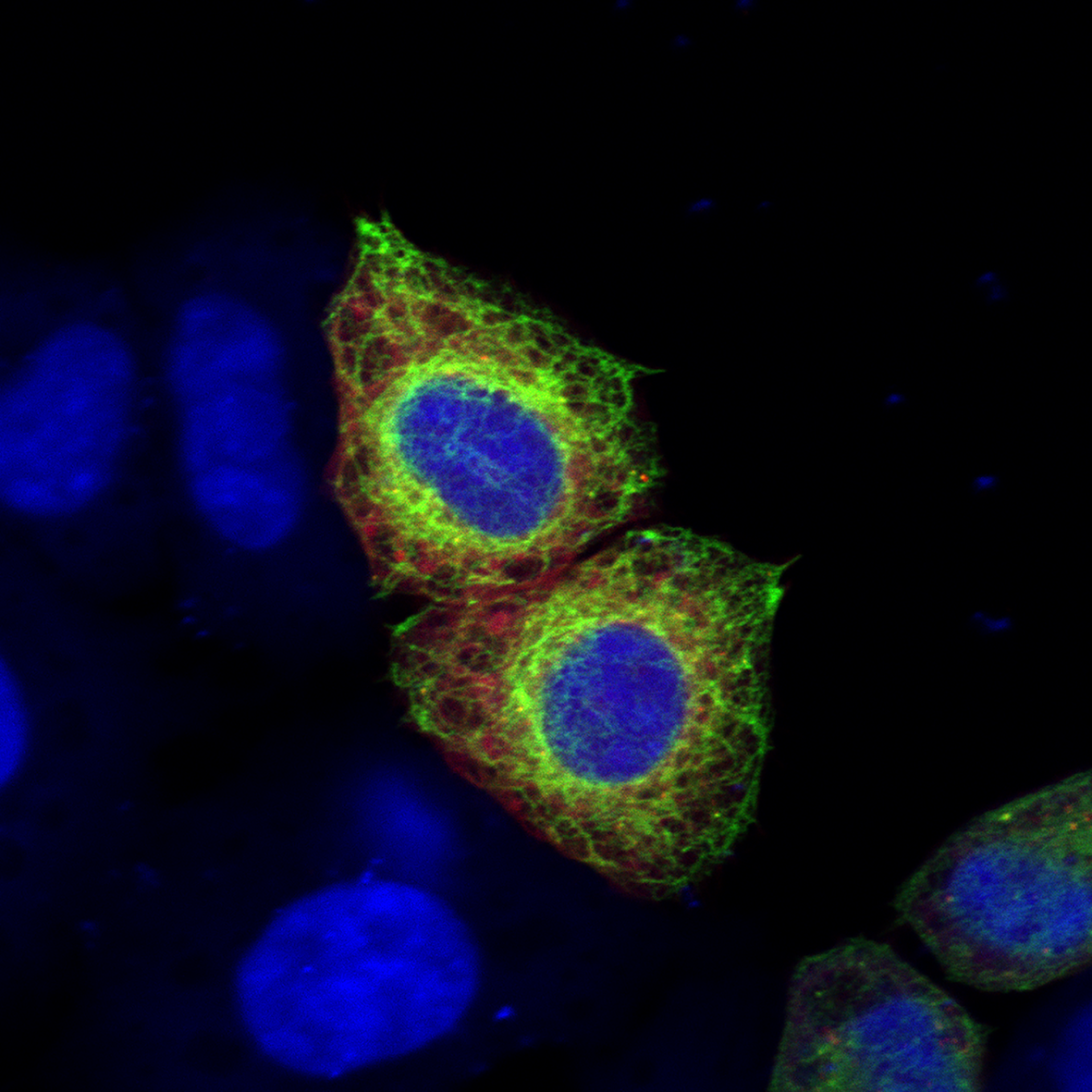

Supplement: Supplementary file 7 — Source data Fig. 4 [file 44318_2025_581_MOESM7_ESM.zip › Fig 4/4E/FAM134C-M-Flag merged STV 0h.tif]

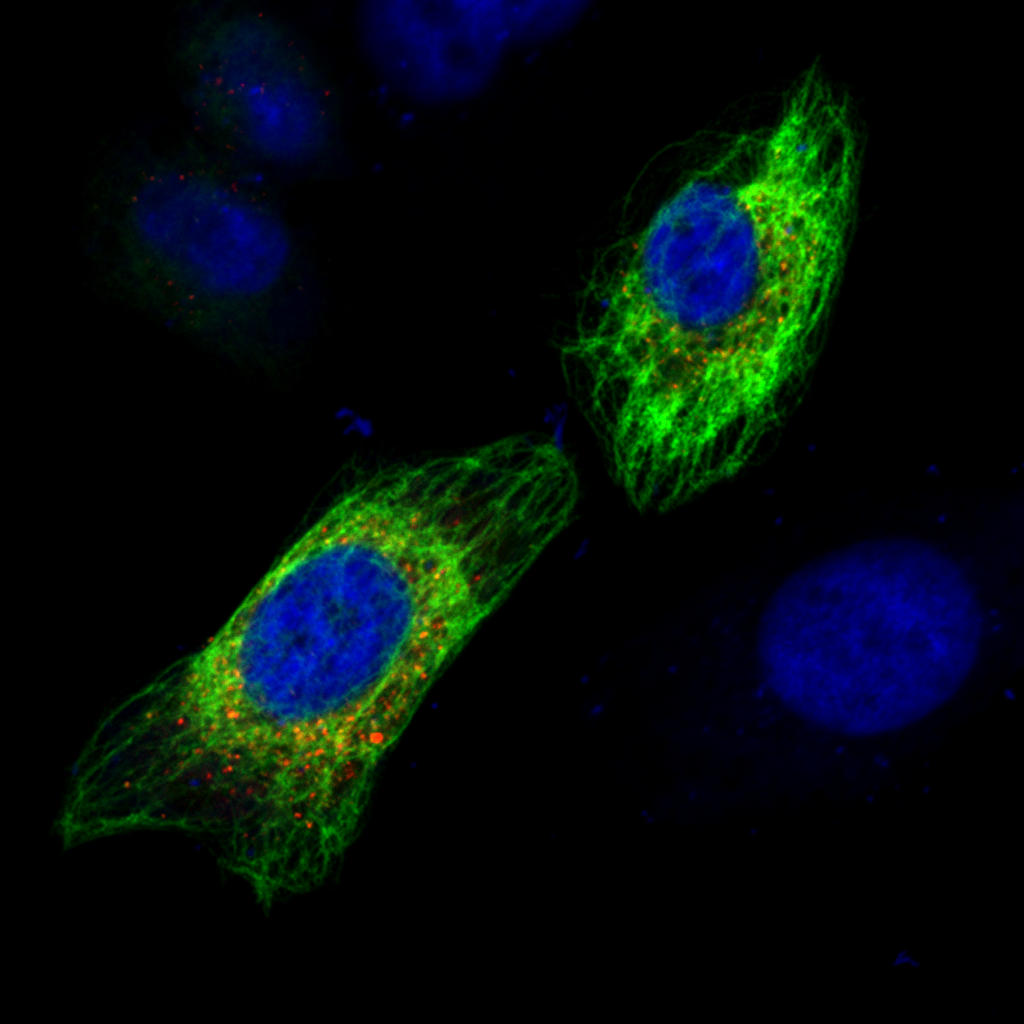

Supplement: Supplementary file 7 — Source data Fig. 4 [file 44318_2025_581_MOESM7_ESM.zip › Fig 4/4E/FAM134C-D-Flag merged STV 1h.tif]

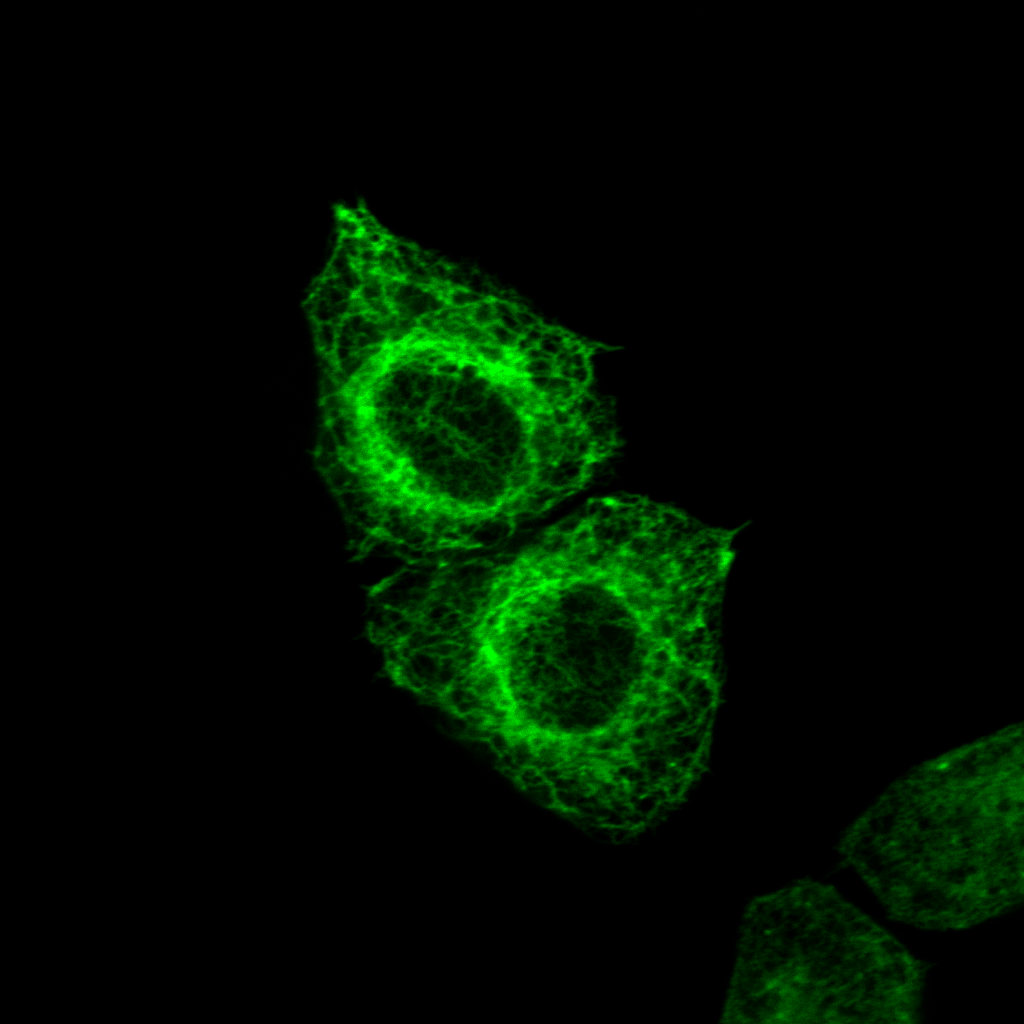

Supplement: Supplementary file 7 — Source data Fig. 4 [file 44318_2025_581_MOESM7_ESM.zip › Fig 4/4E/FAM134C-M-Flag Flag-488 STV 0h.tif]

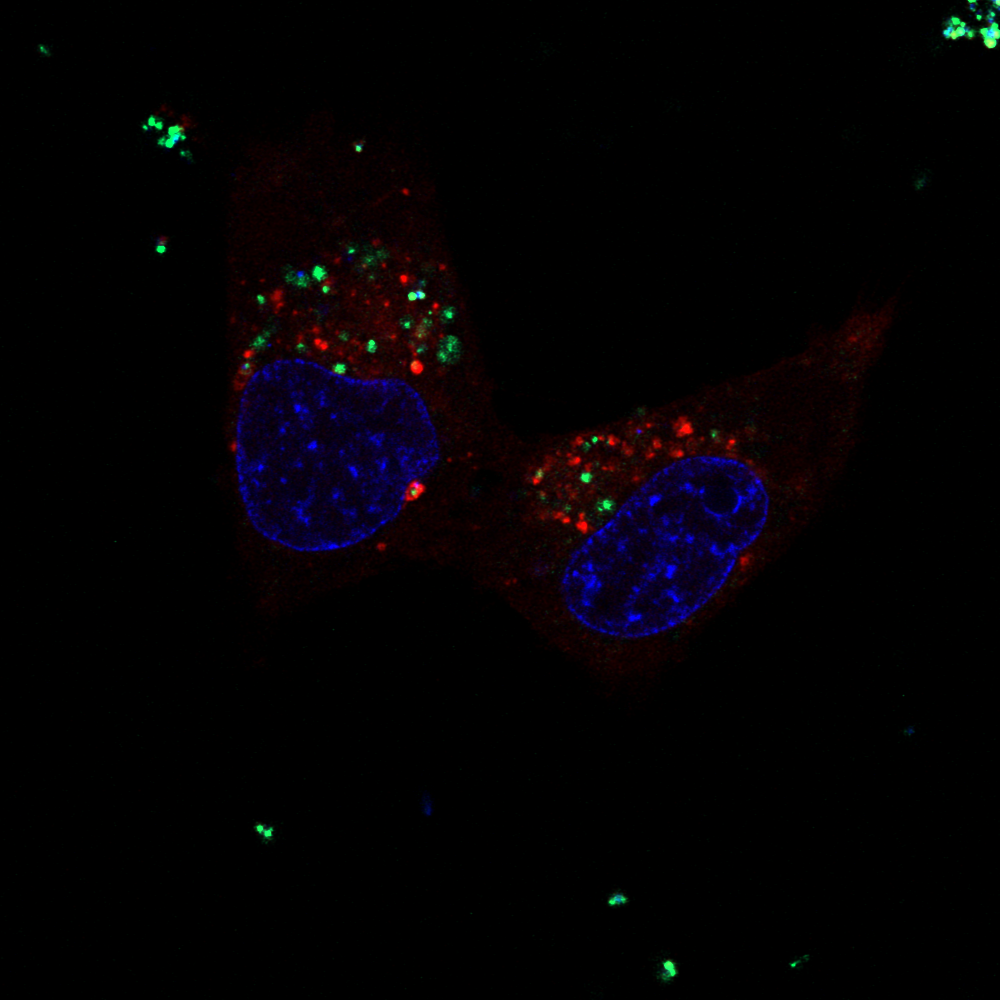

Supplement: Supplementary file 7 — Source data Fig. 4 [file 44318_2025_581_MOESM7_ESM.zip › Fig 4/4B/STV 0h merged.tif]

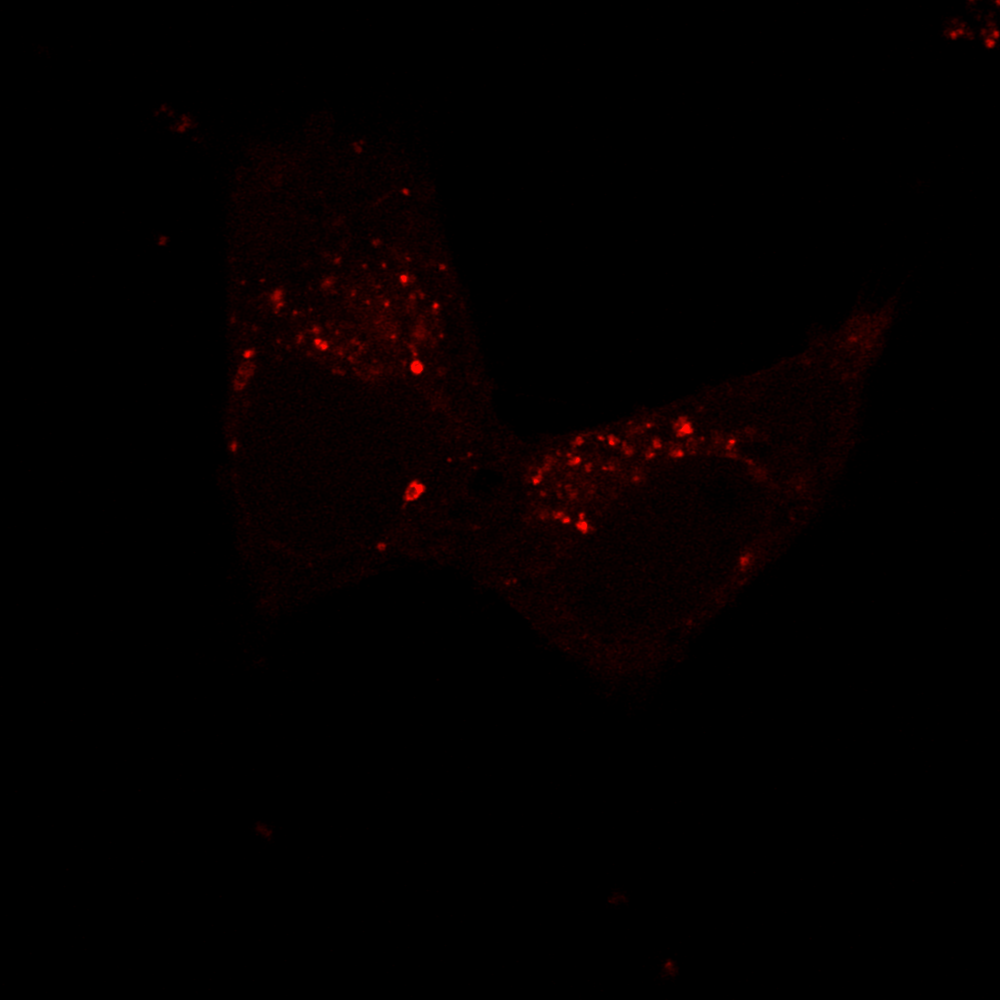

Supplement: Supplementary file 7 — Source data Fig. 4 [file 44318_2025_581_MOESM7_ESM.zip › Fig 4/4B/STV 0h LC3-mcherry.tif]

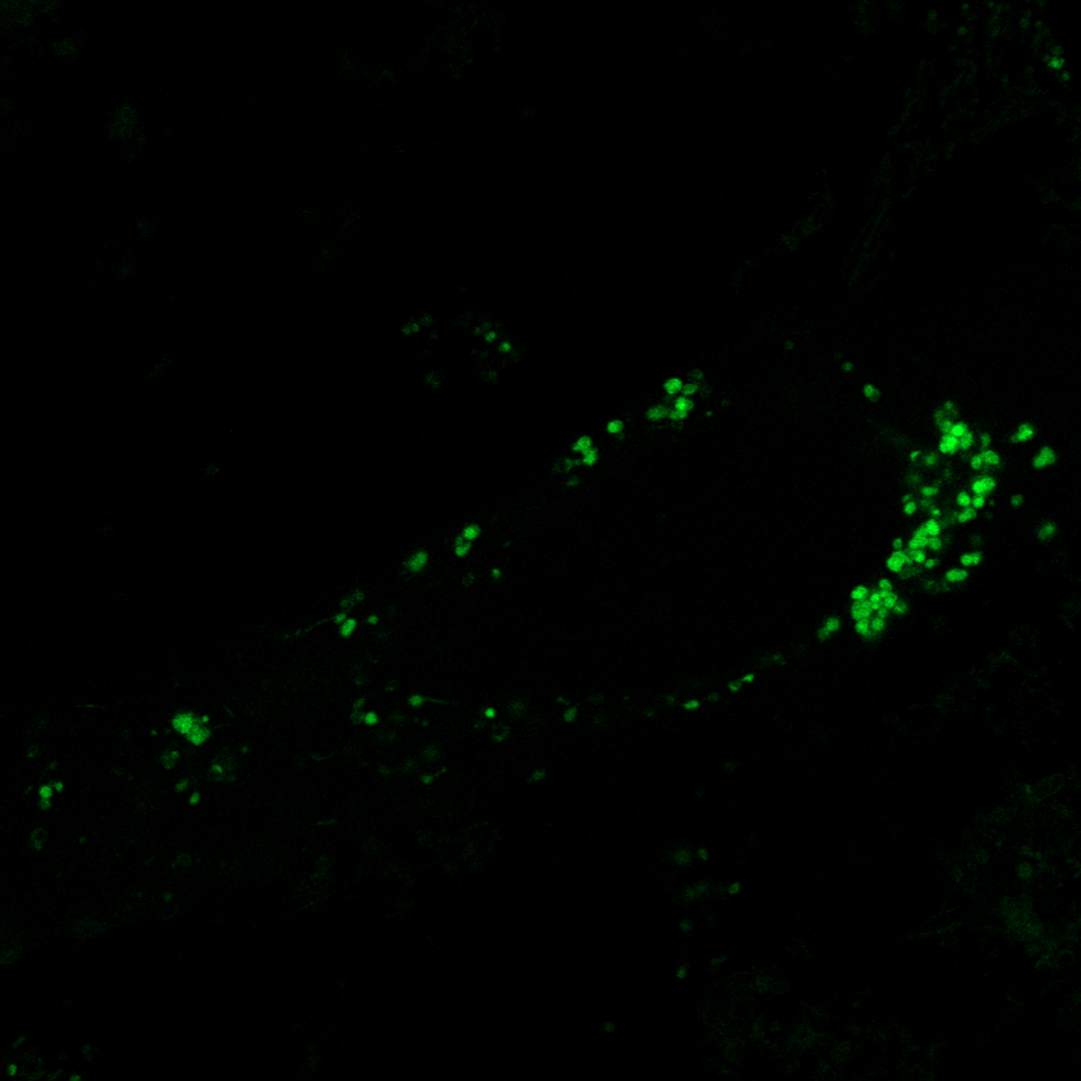

Supplement: Supplementary file 7 — Source data Fig. 4 [file 44318_2025_581_MOESM7_ESM.zip › Fig 4/4B/STV 2h FAM134C-2YC BMPR1a-2YN.tif]

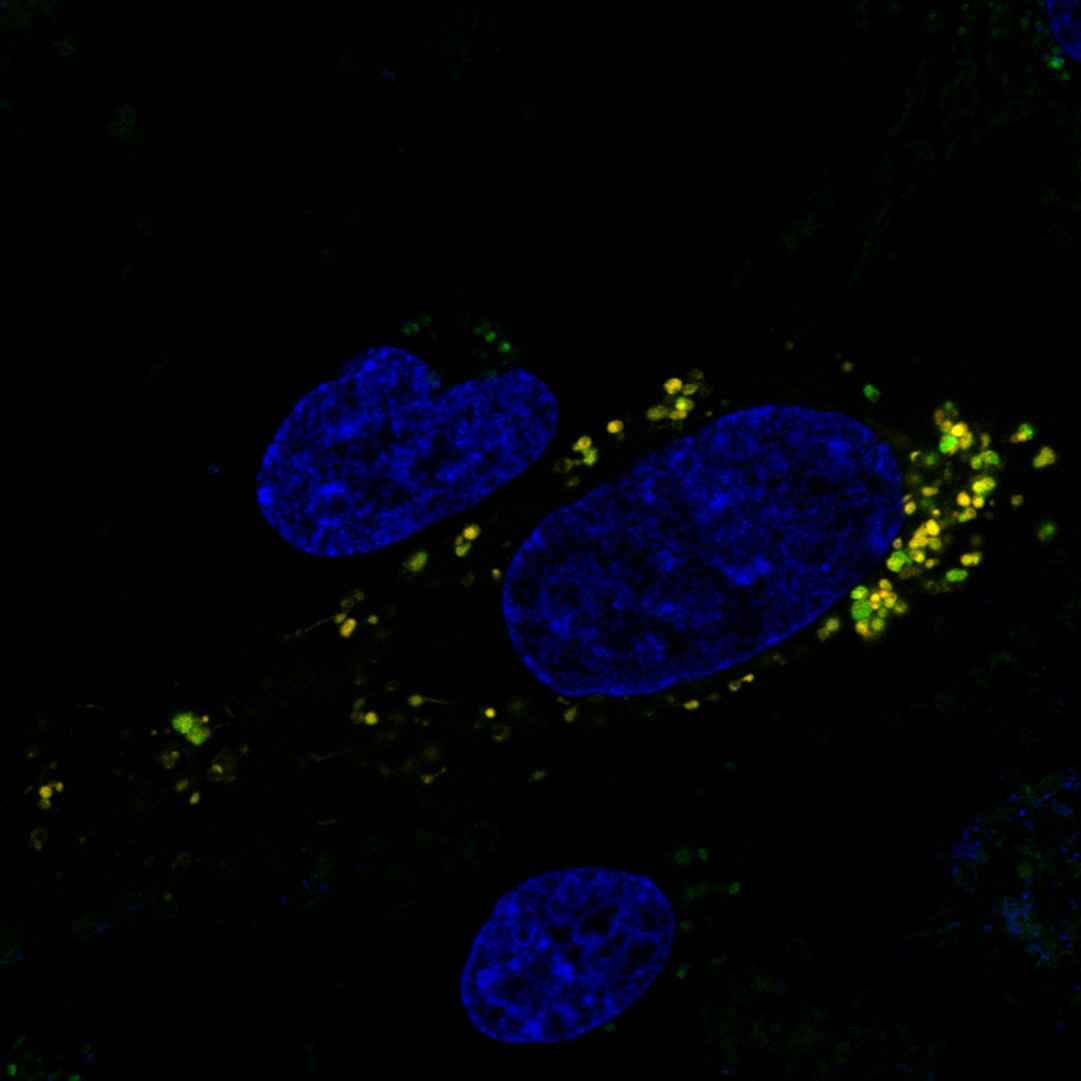

Supplement: Supplementary file 7 — Source data Fig. 4 [file 44318_2025_581_MOESM7_ESM.zip › Fig 4/4B/STV 2h merged.tiff]

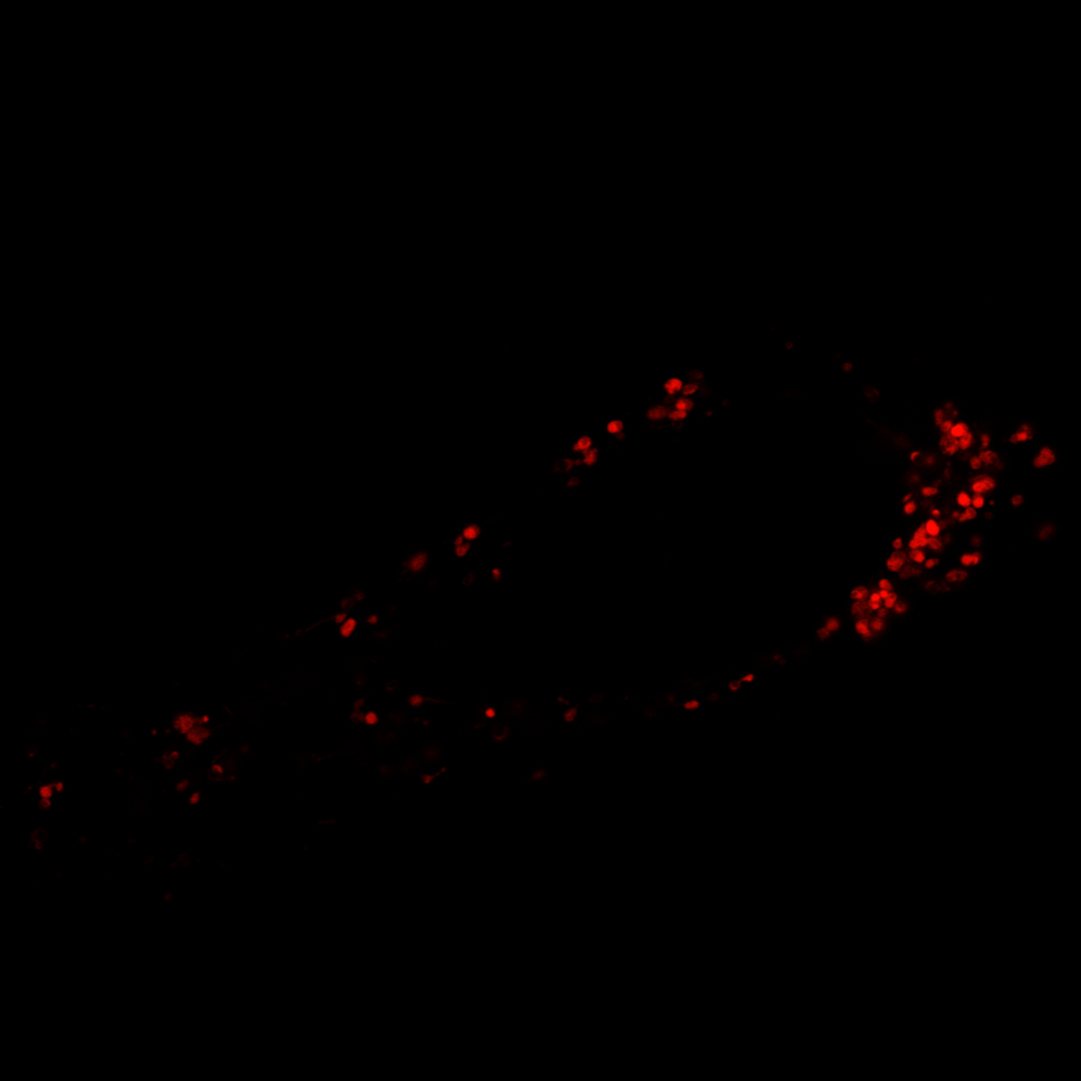

Supplement: Supplementary file 7 — Source data Fig. 4 [file 44318_2025_581_MOESM7_ESM.zip › Fig 4/4B/STV 2h LC3-mcherry.tif]

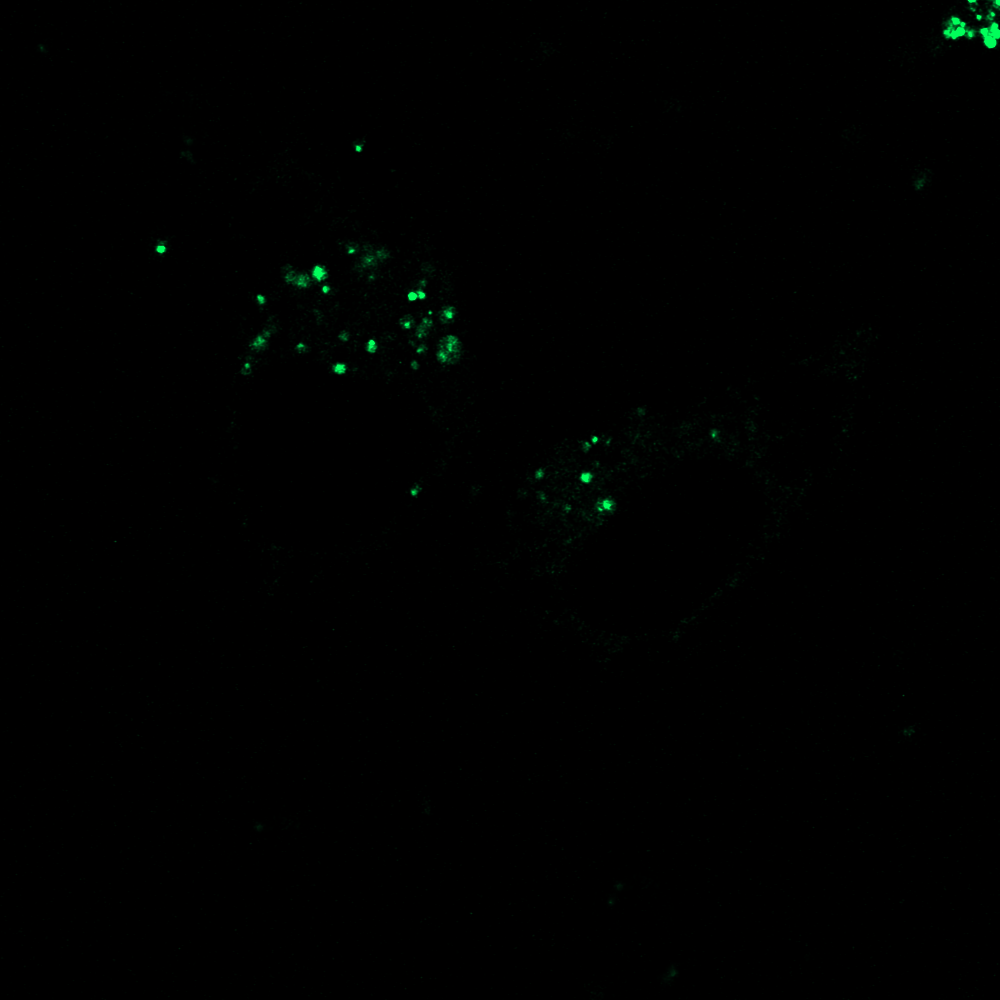

Supplement: Supplementary file 7 — Source data Fig. 4 [file 44318_2025_581_MOESM7_ESM.zip › Fig 4/4B/STV 0h FAM134C-2YC BMPR1a-2YN.tif]

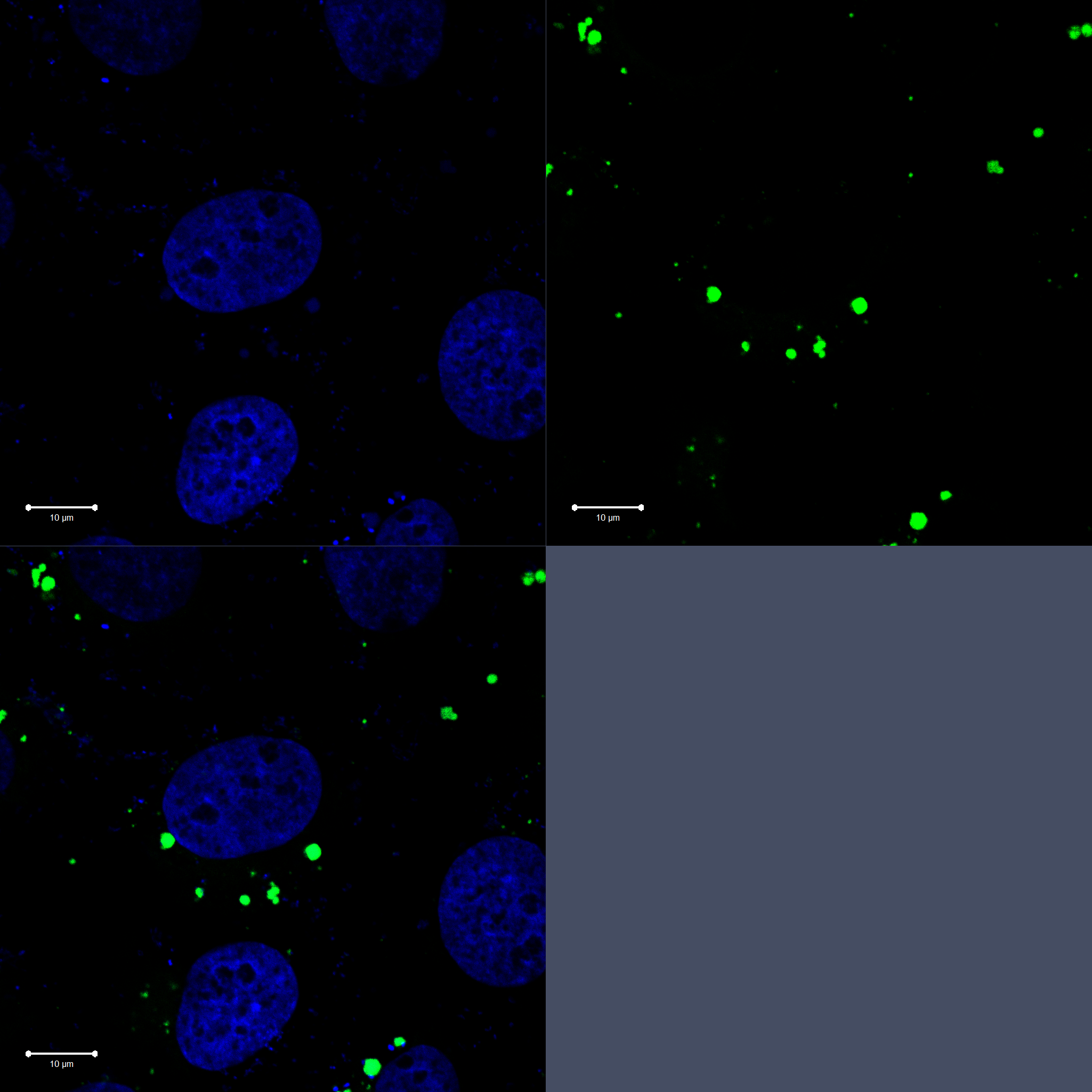

Supplement: Supplementary file 7 — Source data Fig. 4 [file 44318_2025_581_MOESM7_ESM.zip › Fig 4/4A/LC3-2YN FAM134C-2YC.tif]

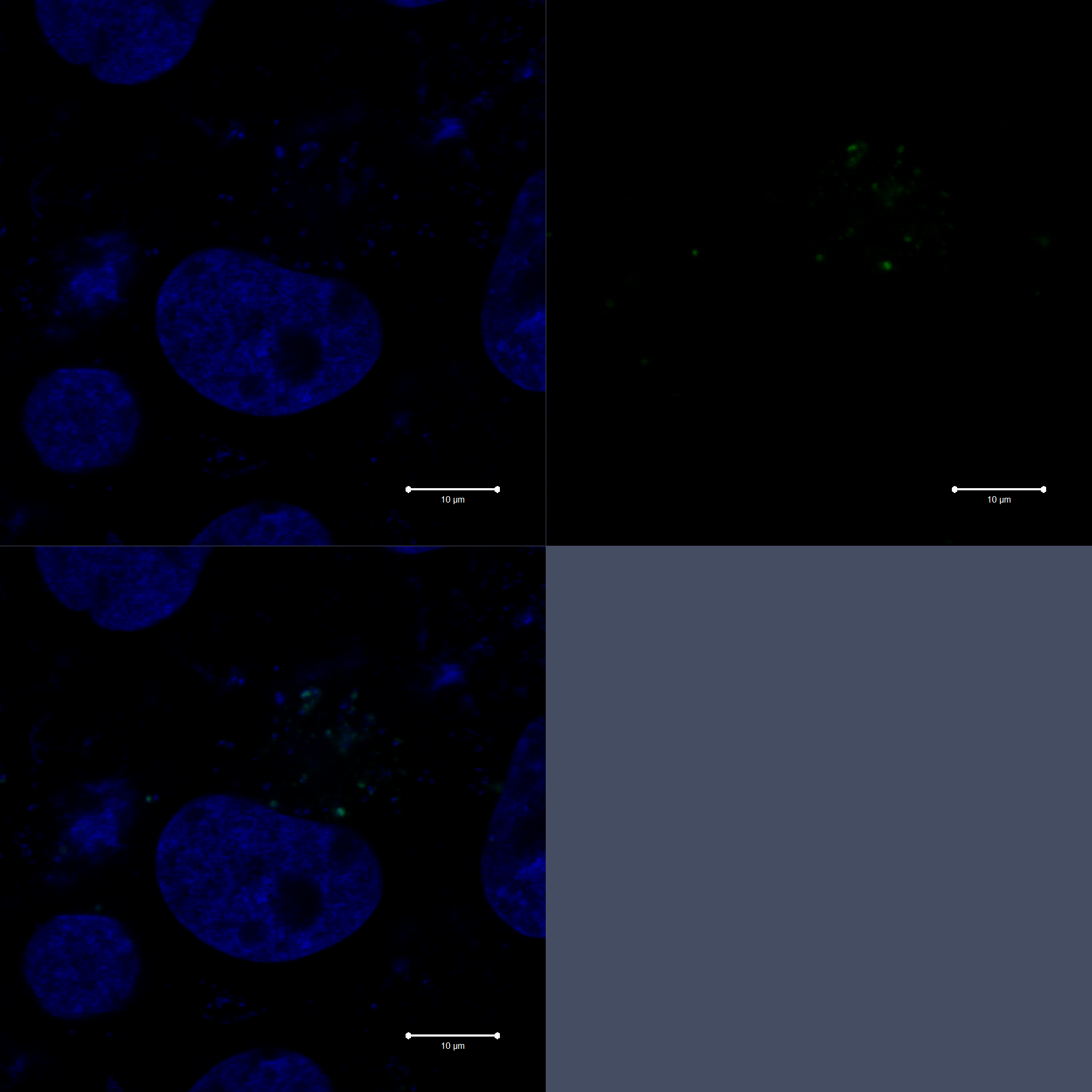

Supplement: Supplementary file 7 — Source data Fig. 4 [file 44318_2025_581_MOESM7_ESM.zip › Fig 4/4A/Vector-2YN FAM134C-2YC.tif]

BMPR1a

75kD  
50kD

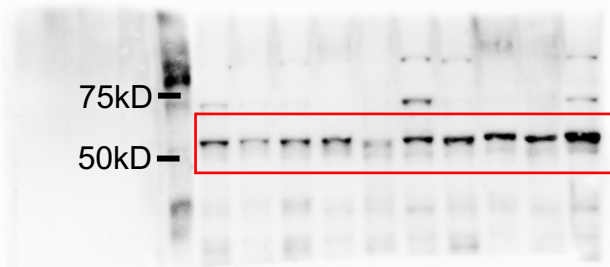

FAM134C

75kD  
50kD

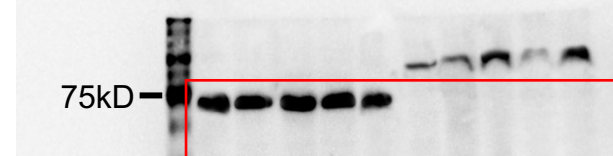

GAPDH

50kD  
36kD

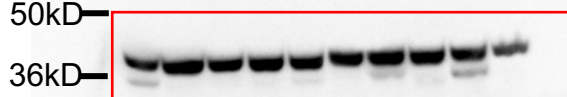

Supplement: Supplementary file 8 — Source data Fig. 5 [file 44318_2025_581_MOESM8_ESM.zip › Fig 5/5D.pdf]

BMPR1A

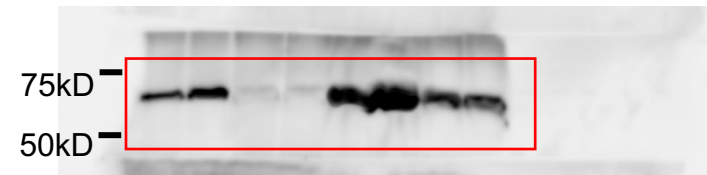

GAPDH

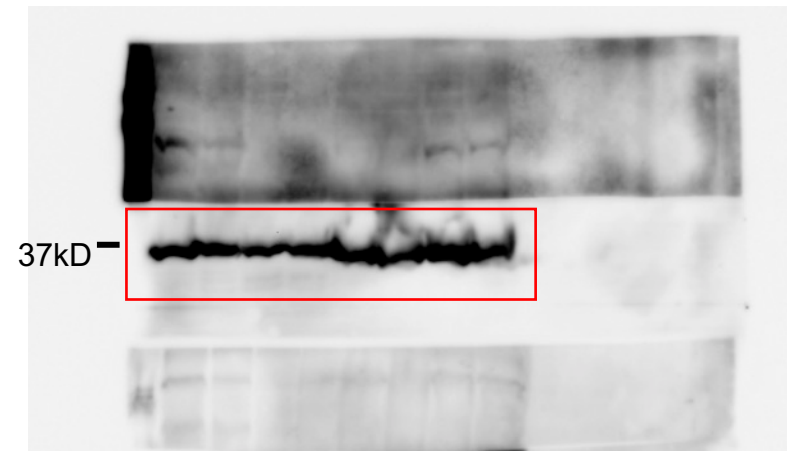

Supplement: Supplementary file 8 — Source data Fig. 5 [file 44318_2025_581_MOESM8_ESM.zip › Fig 5/5G.pdf]

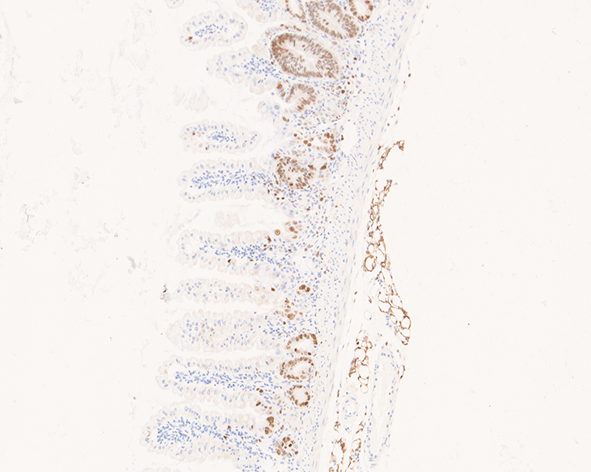

Supplement: Supplementary file 8 — Source data Fig. 5 [file 44318_2025_581_MOESM8_ESM.zip › Fig 5/5I/KO day4.tif]

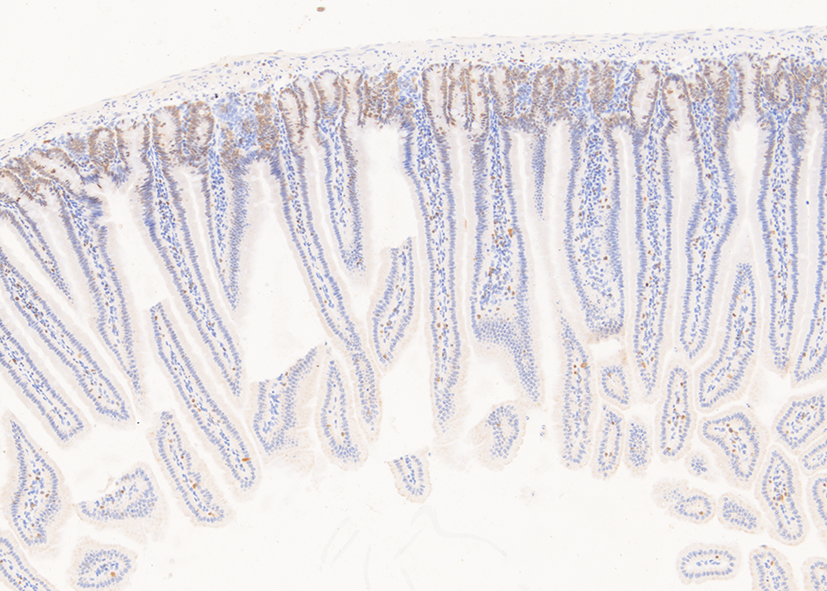

Supplement: Supplementary file 8 — Source data Fig. 5 [file 44318_2025_581_MOESM8_ESM.zip › Fig 5/5I/KO day0.tif]

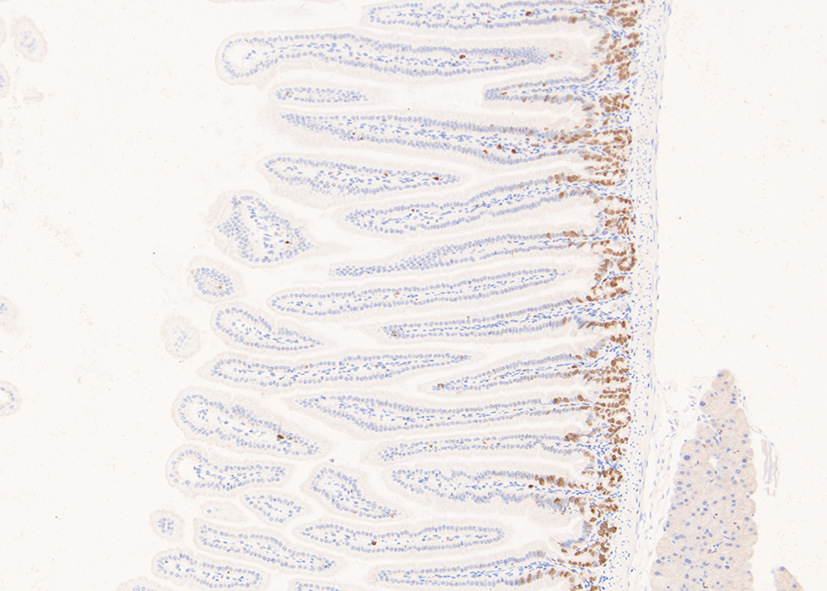

Supplement: Supplementary file 8 — Source data Fig. 5 [file 44318_2025_581_MOESM8_ESM.zip › Fig 5/5I/KO day1.tif]

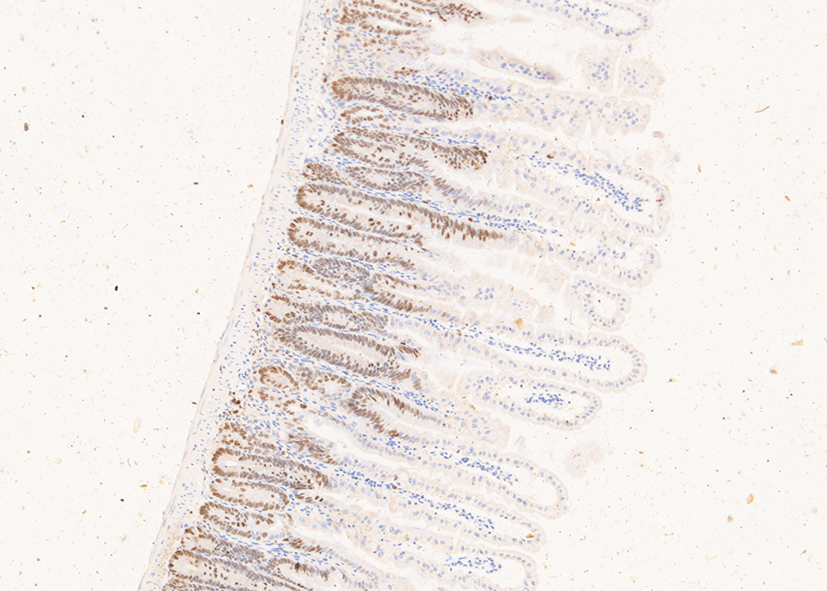

Supplement: Supplementary file 8 — Source data Fig. 5 [file 44318_2025_581_MOESM8_ESM.zip › Fig 5/5I/WT day4.tif]

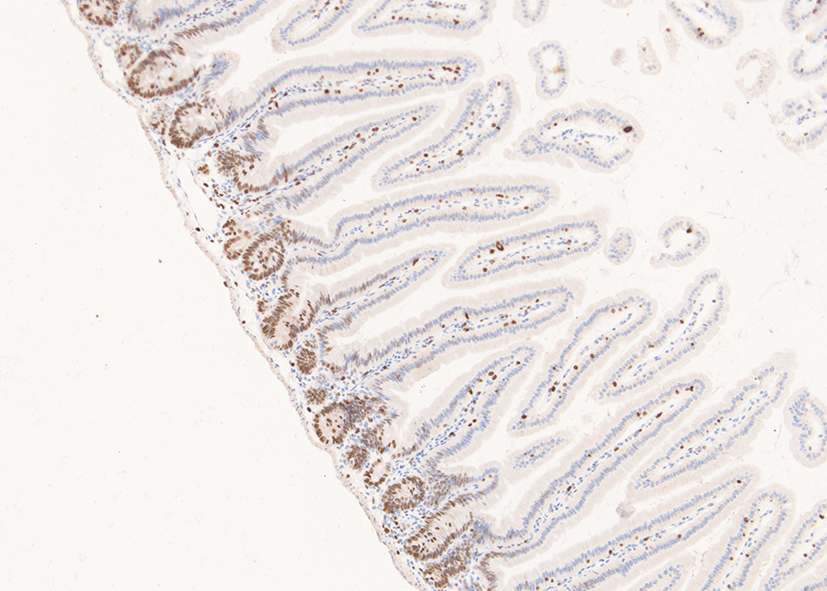

Supplement: Supplementary file 8 — Source data Fig. 5 [file 44318_2025_581_MOESM8_ESM.zip › Fig 5/5I/WT day0.tif]

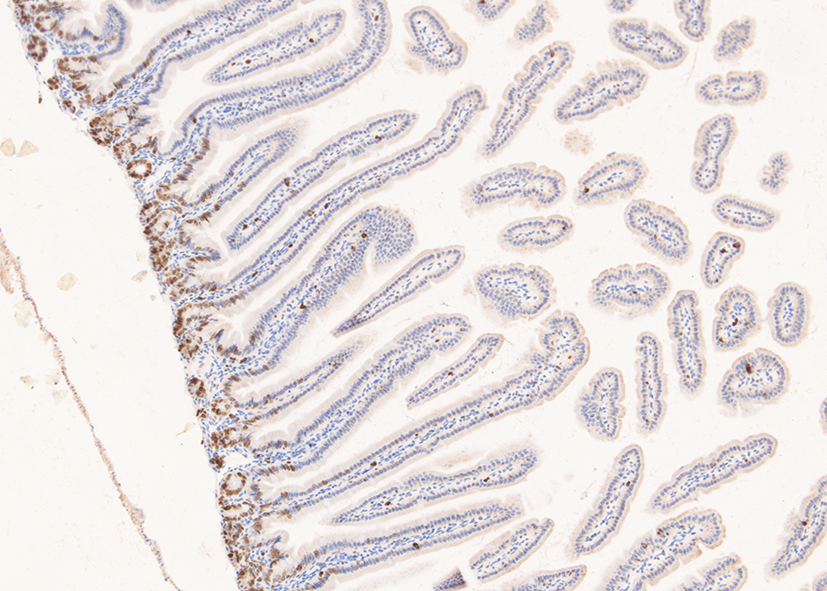

Supplement: Supplementary file 8 — Source data Fig. 5 [file 44318_2025_581_MOESM8_ESM.zip › Fig 5/5I/WT8 day1.tif]

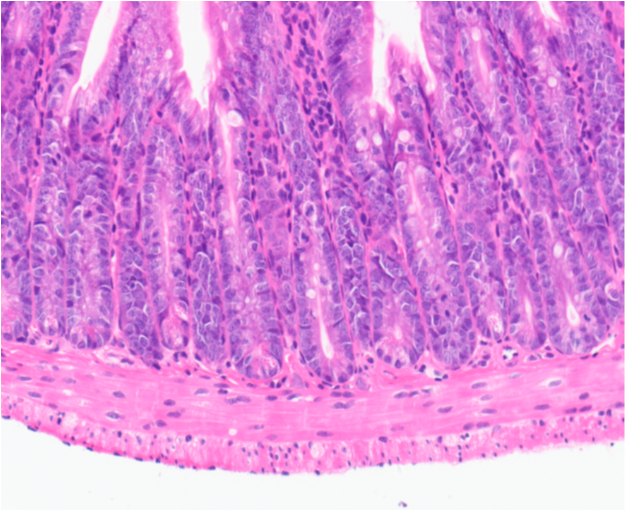

Supplement: Supplementary file 8 — Source data Fig. 5 [file 44318_2025_581_MOESM8_ESM.zip › Fig 5/5F/KO fasting HE.tif]

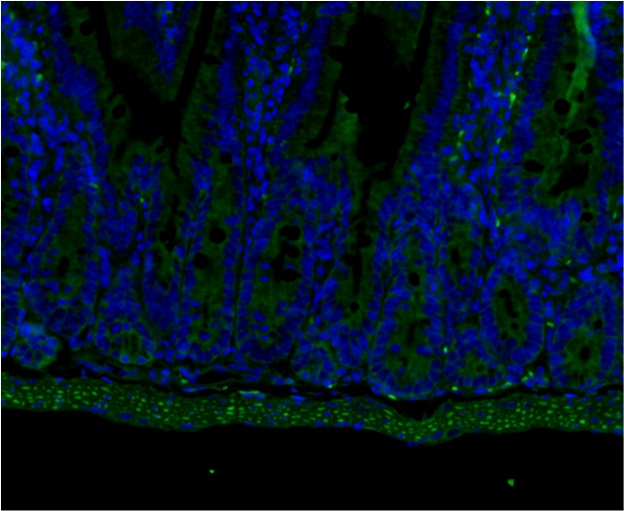

Supplement: Supplementary file 8 — Source data Fig. 5 [file 44318_2025_581_MOESM8_ESM.zip › Fig 5/5F/KO fasting p62.tif]

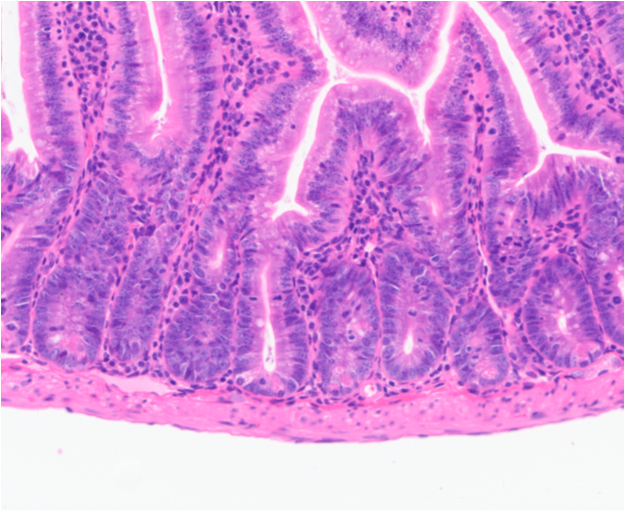

Supplement: Supplementary file 8 — Source data Fig. 5 [file 44318_2025_581_MOESM8_ESM.zip › Fig 5/5F/KO Feed HE.tif]

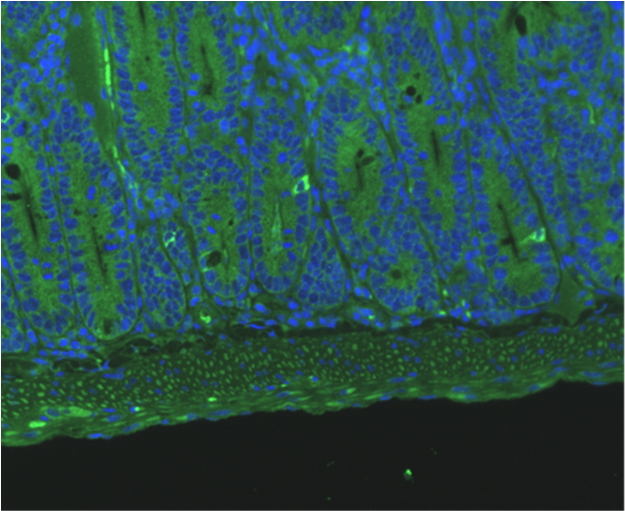

Supplement: Supplementary file 8 — Source data Fig. 5 [file 44318_2025_581_MOESM8_ESM.zip › Fig 5/5F/KO feed p62.tif]
