# Supplementary figures and images for: Reticulophagy receptor FAM134C restrains BMP receptor signaling (part 2 of 3)
Source: EMBO J. 2025 Oct 20;44(23):7154–80. doi: 10.1038/s44318-025-00581-3 (PMC12669696; doi:10.1038/s44318-025-00581-3)

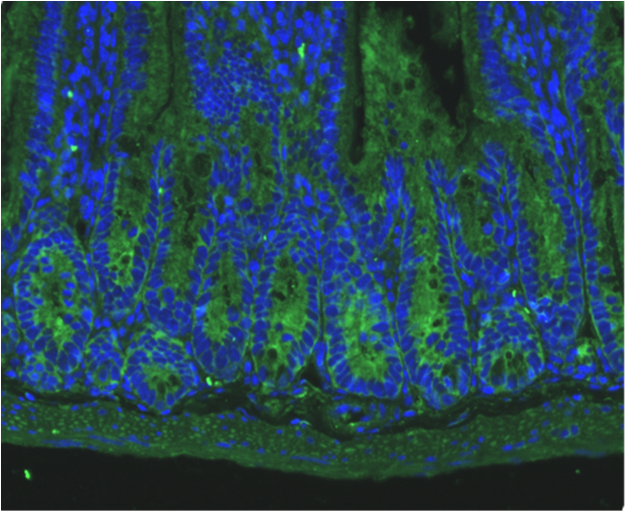

Supplement: Supplementary file 8 — Source data Fig. 5 [file 44318_2025_581_MOESM8_ESM.zip › Fig 5/5F/KO Fasting BMPR1A.tif]

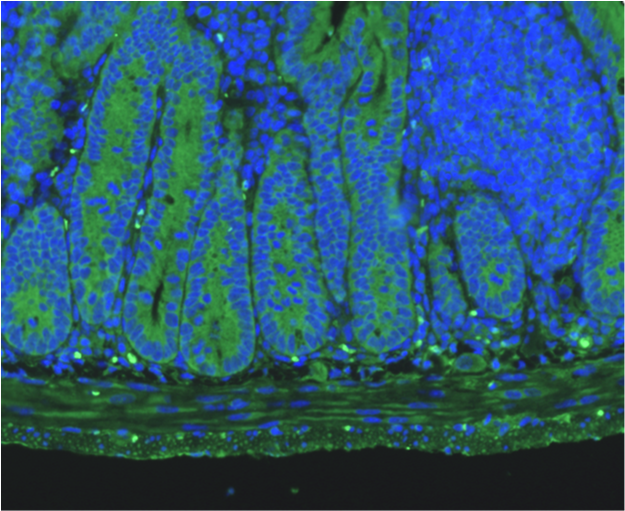

Supplement: Supplementary file 8 — Source data Fig. 5 [file 44318_2025_581_MOESM8_ESM.zip › Fig 5/5F/KO Feed BMPR1A.tif]

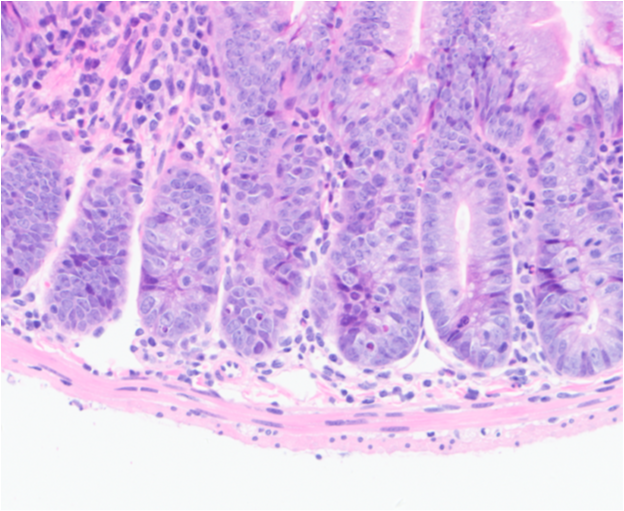

Supplement: Supplementary file 8 — Source data Fig. 5 [file 44318_2025_581_MOESM8_ESM.zip › Fig 5/5F/WT feed HE.tif]

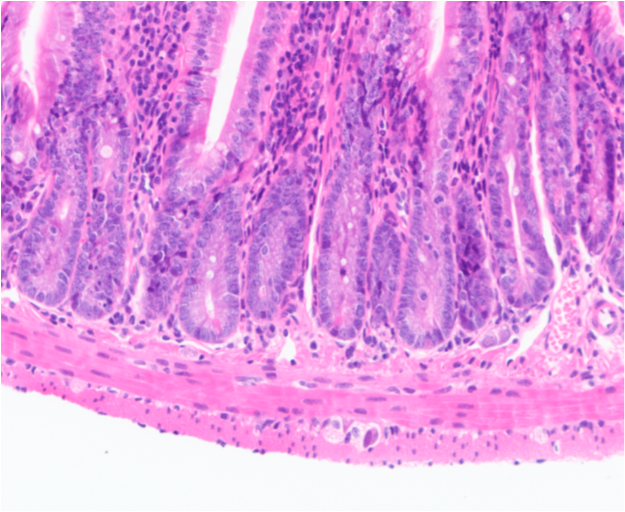

Supplement: Supplementary file 8 — Source data Fig. 5 [file 44318_2025_581_MOESM8_ESM.zip › Fig 5/5F/WT fasting HE.tif]

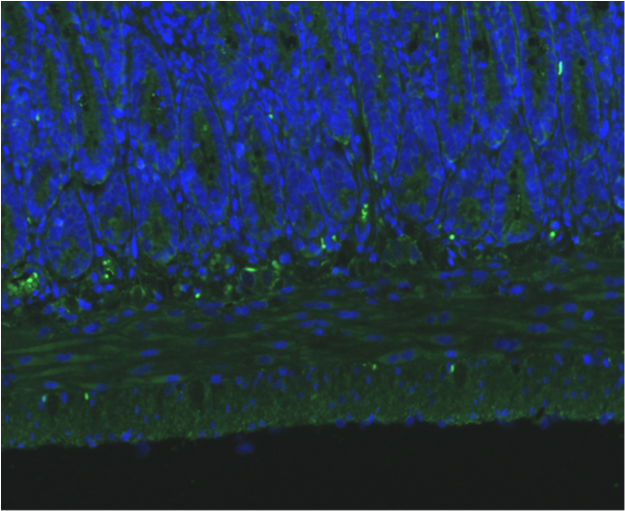

Supplement: Supplementary file 8 — Source data Fig. 5 [file 44318_2025_581_MOESM8_ESM.zip › Fig 5/5F/WT fasting p62.tif]

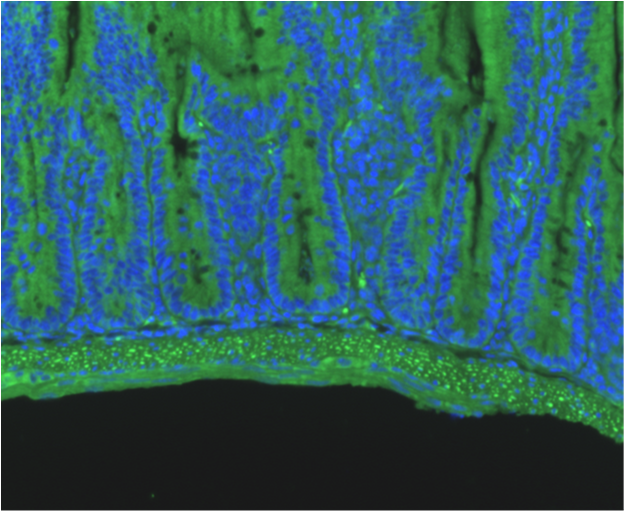

Supplement: Supplementary file 8 — Source data Fig. 5 [file 44318_2025_581_MOESM8_ESM.zip › Fig 5/5F/WT feed p62.tif]

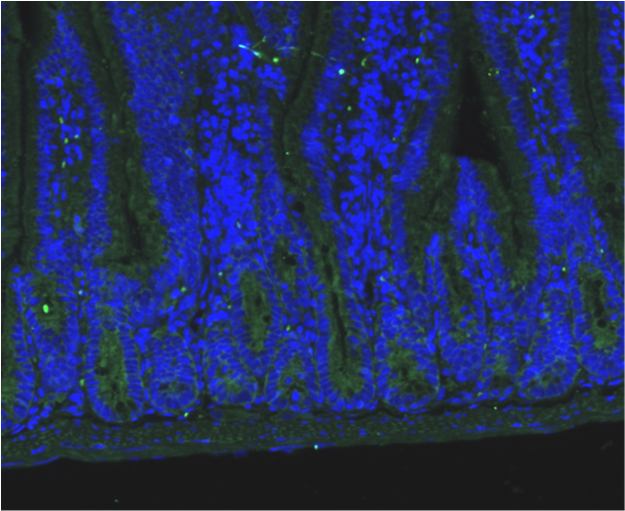

Supplement: Supplementary file 8 — Source data Fig. 5 [file 44318_2025_581_MOESM8_ESM.zip › Fig 5/5F/WT fasting BMPR1A.tif]

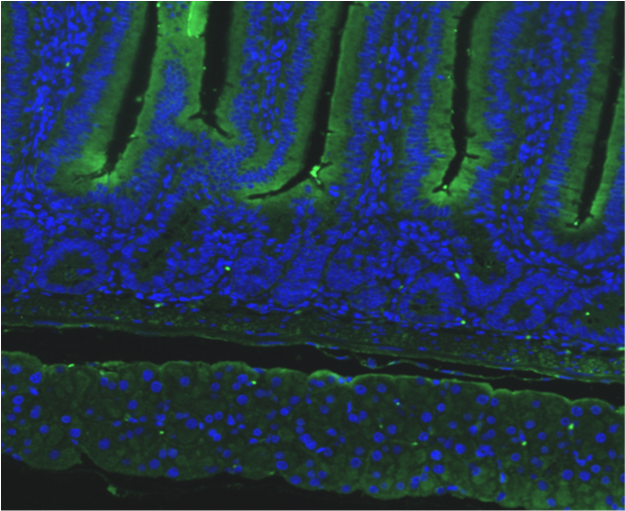

Supplement: Supplementary file 8 — Source data Fig. 5 [file 44318_2025_581_MOESM8_ESM.zip › Fig 5/5F/WT feed BMPR1A.tif]

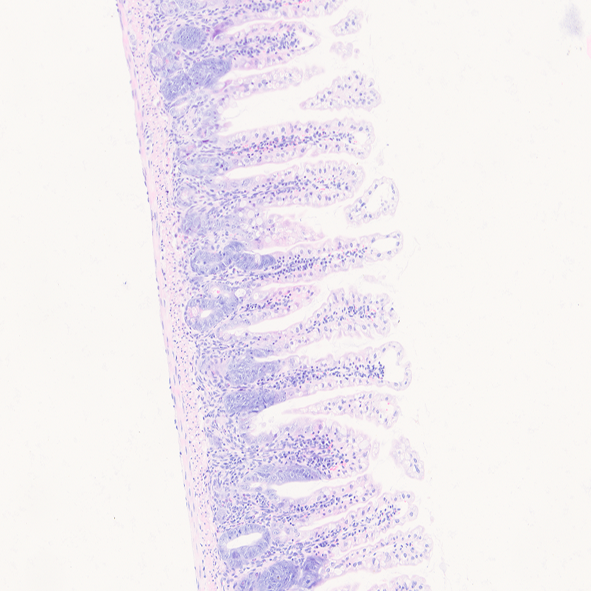

Supplement: Supplementary file 8 — Source data Fig. 5 [file 44318_2025_581_MOESM8_ESM.zip › Fig 5/5H/KO day4.tif]

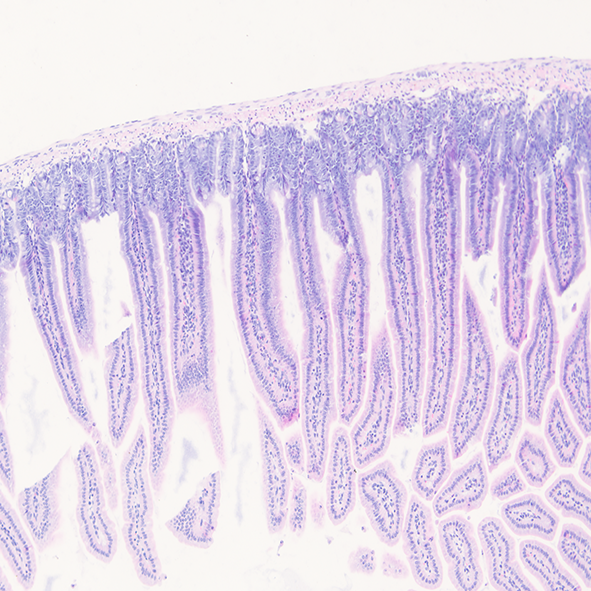

Supplement: Supplementary file 8 — Source data Fig. 5 [file 44318_2025_581_MOESM8_ESM.zip › Fig 5/5H/KO day0.tif]

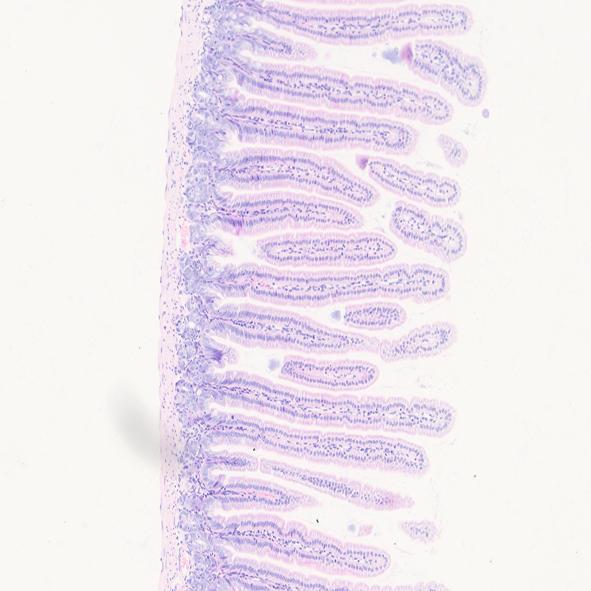

Supplement: Supplementary file 8 — Source data Fig. 5 [file 44318_2025_581_MOESM8_ESM.zip › Fig 5/5H/KO day1.tif]

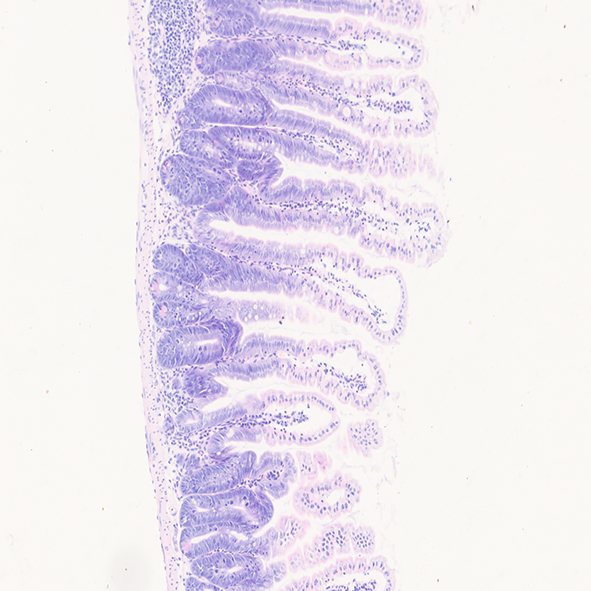

Supplement: Supplementary file 8 — Source data Fig. 5 [file 44318_2025_581_MOESM8_ESM.zip › Fig 5/5H/WT day4.tif]

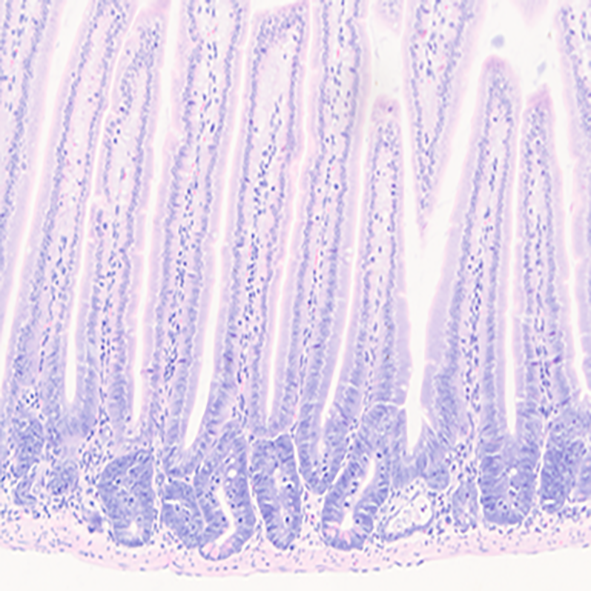

Supplement: Supplementary file 8 — Source data Fig. 5 [file 44318_2025_581_MOESM8_ESM.zip › Fig 5/5H/WT day0.tif]

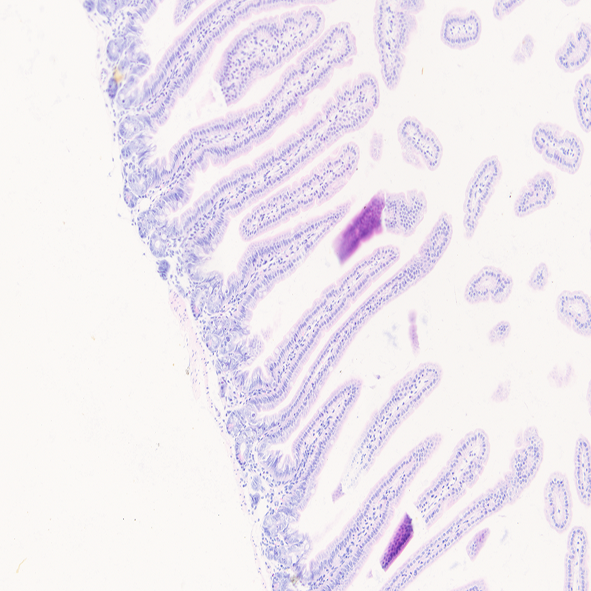

Supplement: Supplementary file 8 — Source data Fig. 5 [file 44318_2025_581_MOESM8_ESM.zip › Fig 5/5H/WT day1.tif]

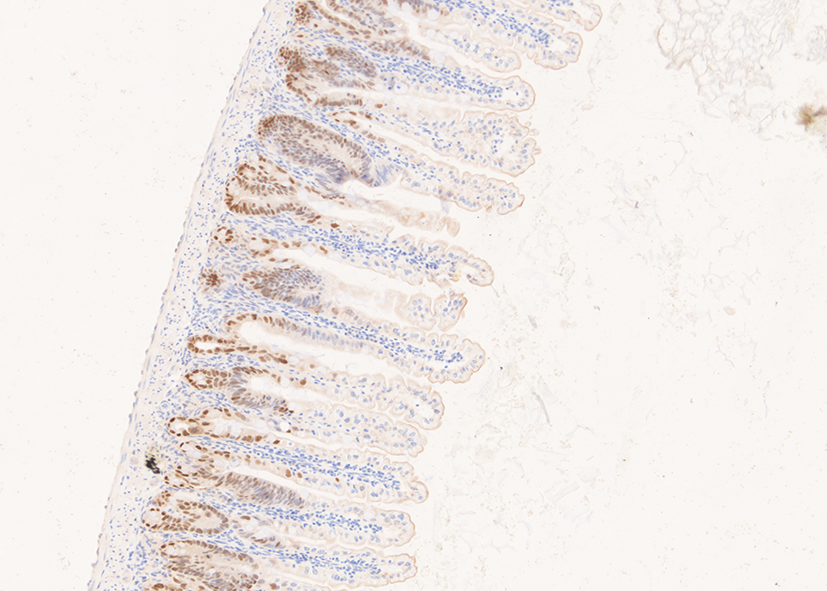

Supplement: Supplementary file 8 — Source data Fig. 5 [file 44318_2025_581_MOESM8_ESM.zip › Fig 5/5J/KO day4 SOX9.tif]

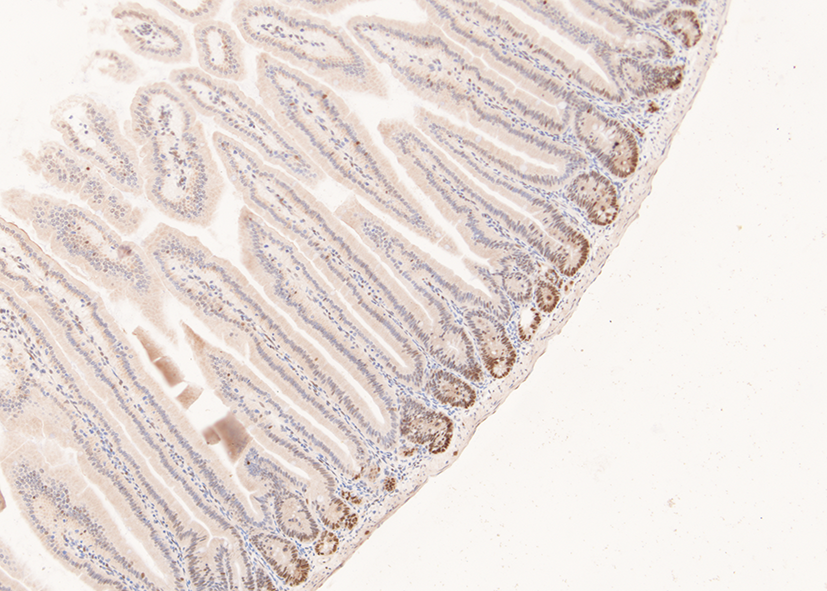

Supplement: Supplementary file 8 — Source data Fig. 5 [file 44318_2025_581_MOESM8_ESM.zip › Fig 5/5J/WT day0 SOX9.tif]

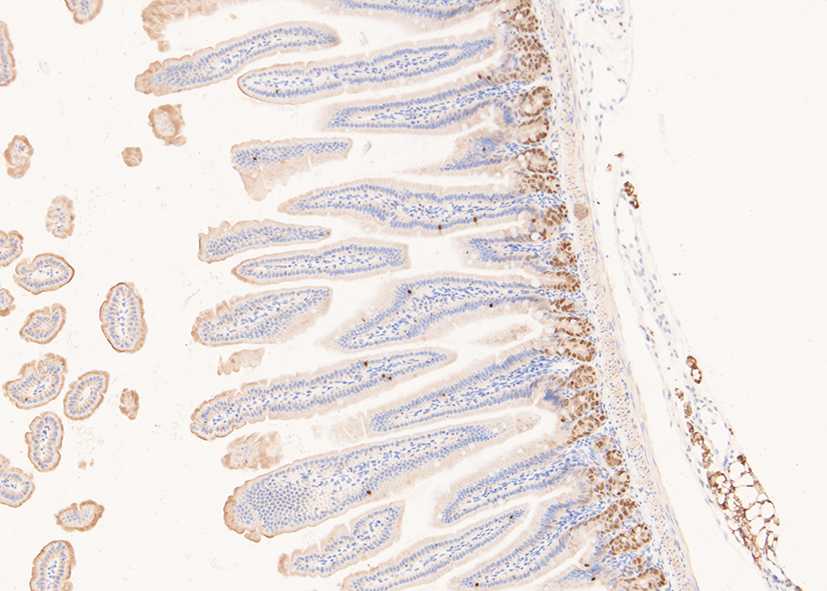

Supplement: Supplementary file 8 — Source data Fig. 5 [file 44318_2025_581_MOESM8_ESM.zip › Fig 5/5J/WT day1 SOX9.tif]

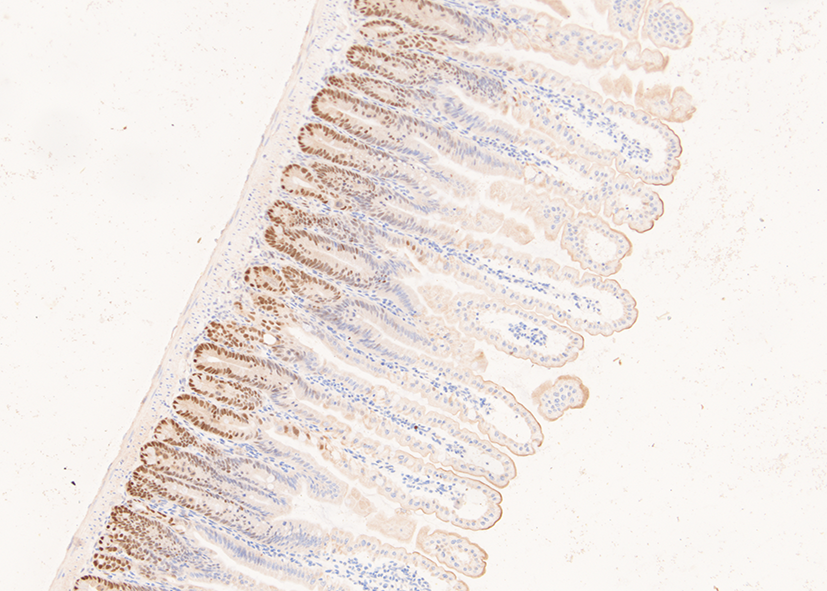

Supplement: Supplementary file 8 — Source data Fig. 5 [file 44318_2025_581_MOESM8_ESM.zip › Fig 5/5J/WT day4 SOX9.tif]

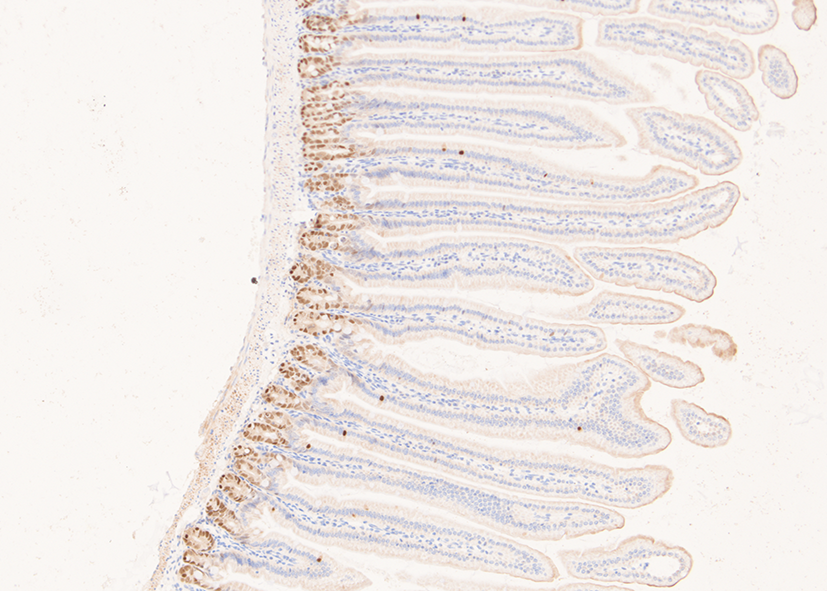

Supplement: Supplementary file 8 — Source data Fig. 5 [file 44318_2025_581_MOESM8_ESM.zip › Fig 5/5J/KO day1 SOX9.tif]

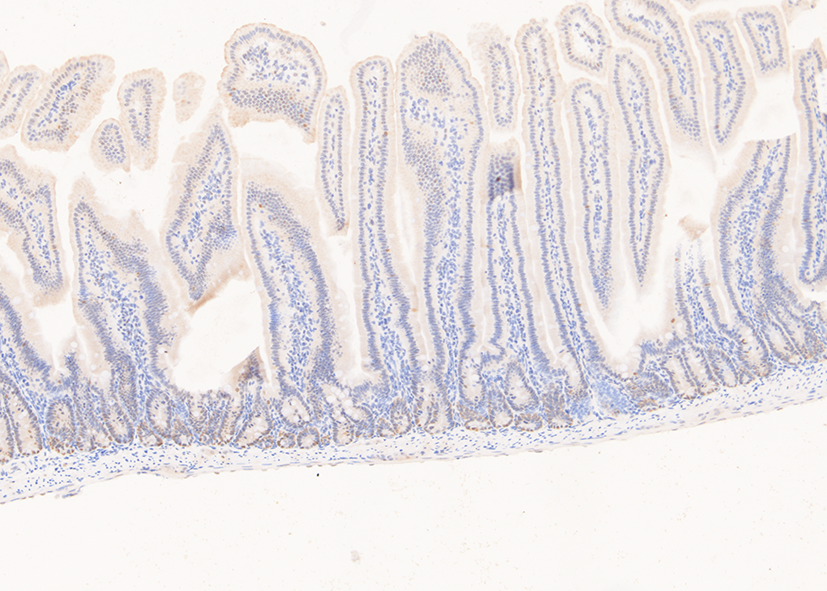

Supplement: Supplementary file 8 — Source data Fig. 5 [file 44318_2025_581_MOESM8_ESM.zip › Fig 5/5J/KO day0 SOX9_1.tif]

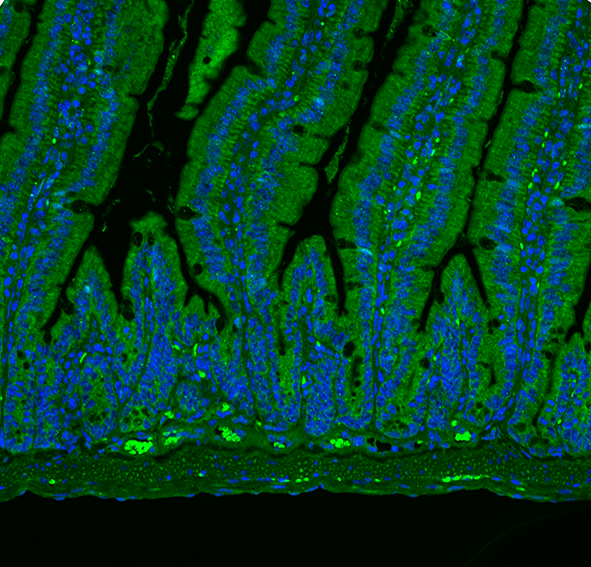

Supplement: Supplementary file 8 — Source data Fig. 5 [file 44318_2025_581_MOESM8_ESM.zip › Fig 5/5C/FAM134C KO p-S158 intestine.tif]

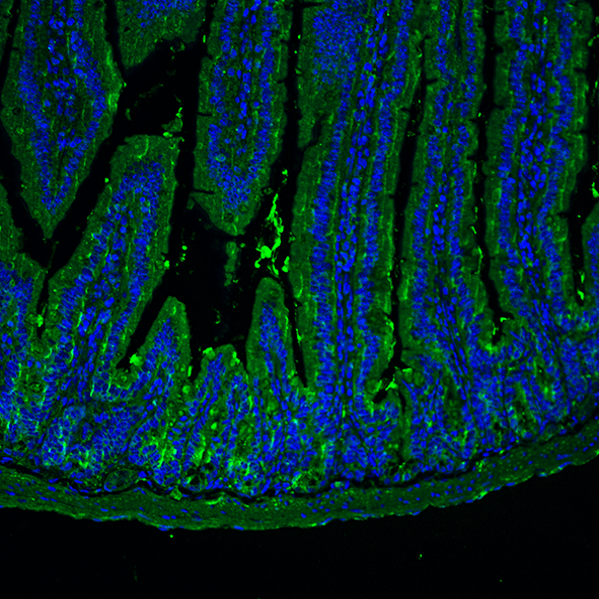

Supplement: Supplementary file 8 — Source data Fig. 5 [file 44318_2025_581_MOESM8_ESM.zip › Fig 5/5C/FAM134C WT BMPR1a intestine.tif]

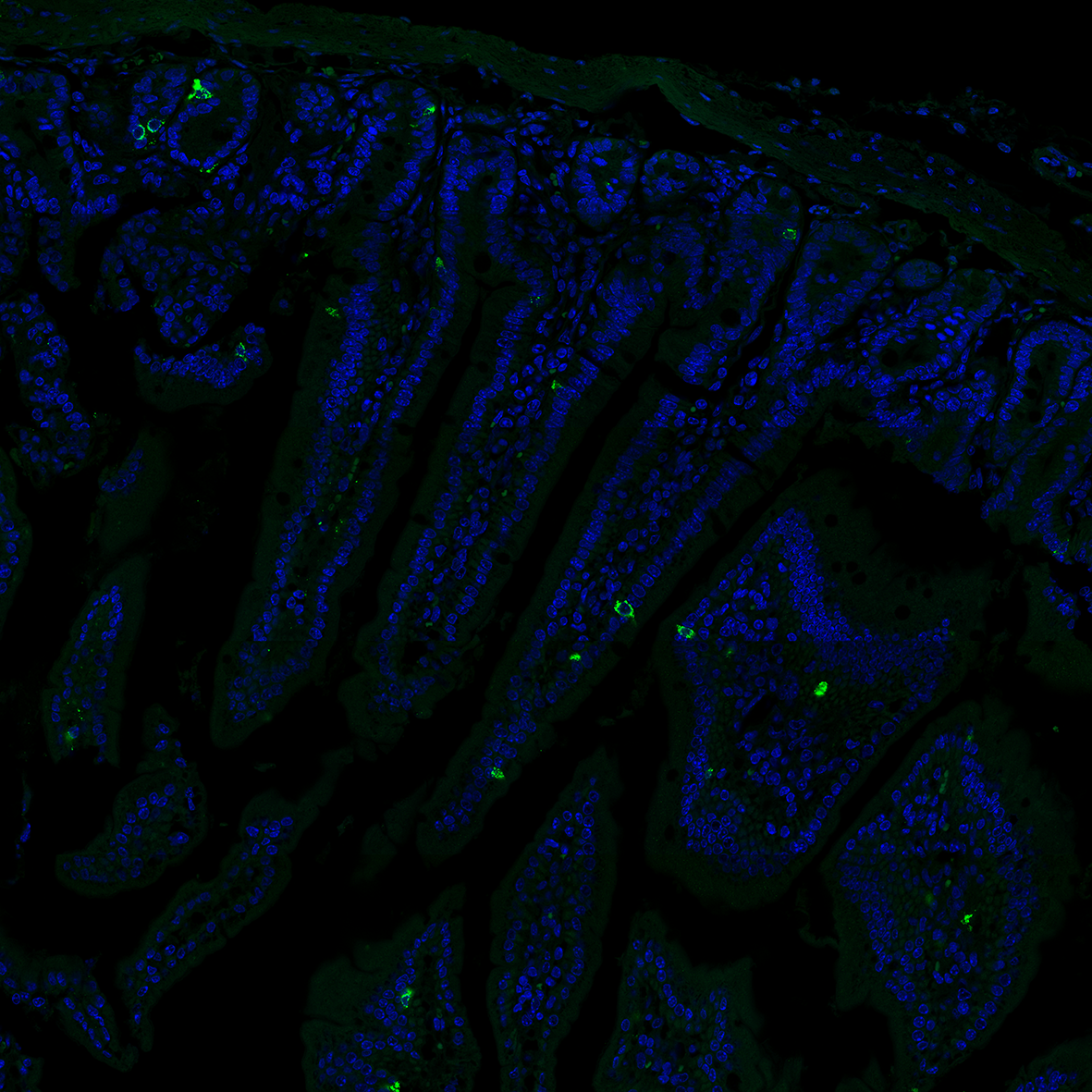

Supplement: Supplementary file 8 — Source data Fig. 5 [file 44318_2025_581_MOESM8_ESM.zip › Fig 5/5C/FAM134C WT ChgA.tif]

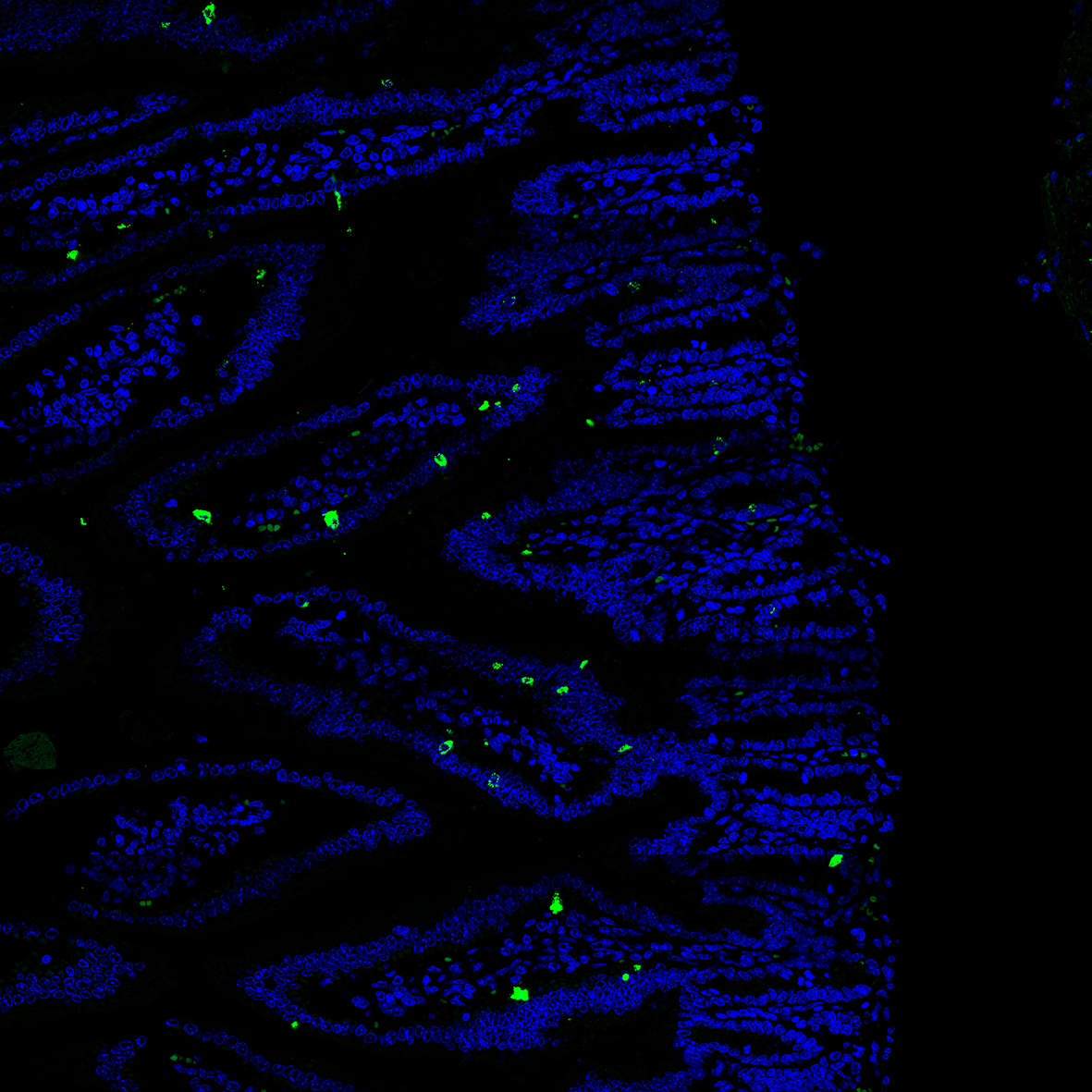

Supplement: Supplementary file 8 — Source data Fig. 5 [file 44318_2025_581_MOESM8_ESM.zip › Fig 5/5C/FAM134C KO ChgA.tif]

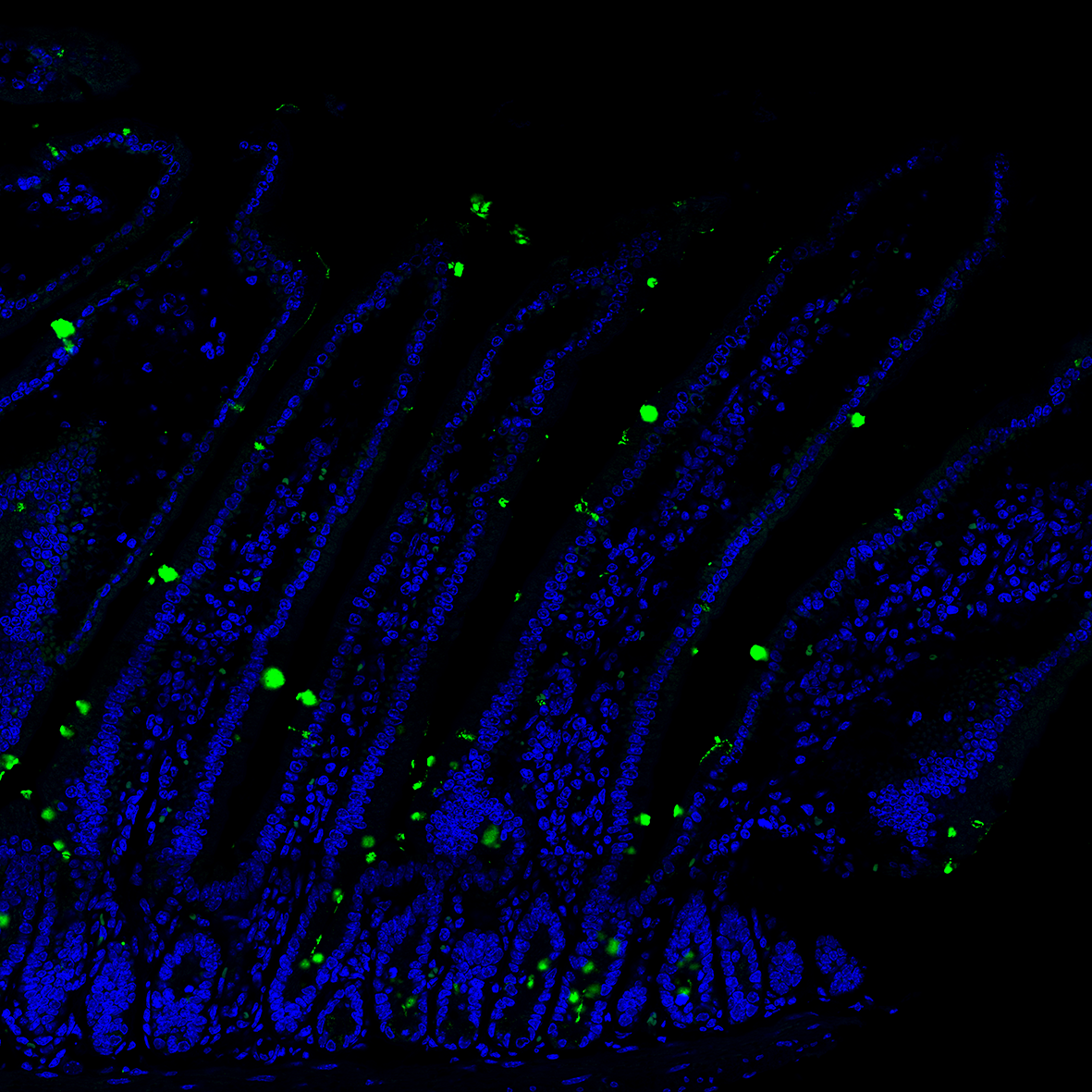

Supplement: Supplementary file 8 — Source data Fig. 5 [file 44318_2025_581_MOESM8_ESM.zip › Fig 5/5C/FAM134C WT MUC2.tif]

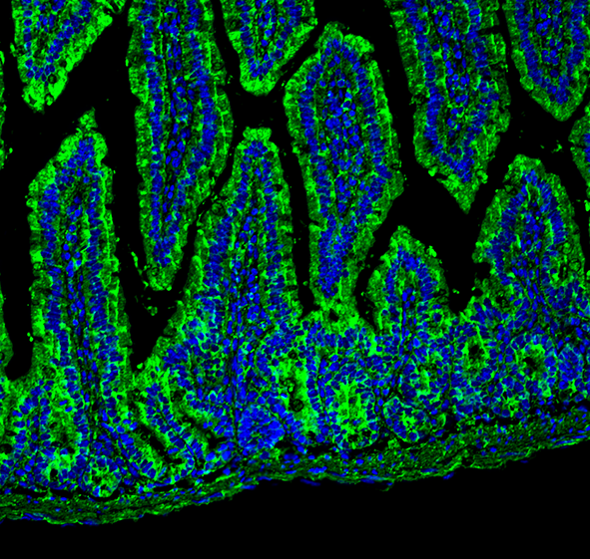

Supplement: Supplementary file 8 — Source data Fig. 5 [file 44318_2025_581_MOESM8_ESM.zip › Fig 5/5C/FAM134C KO BMPR1a intestine.tif]

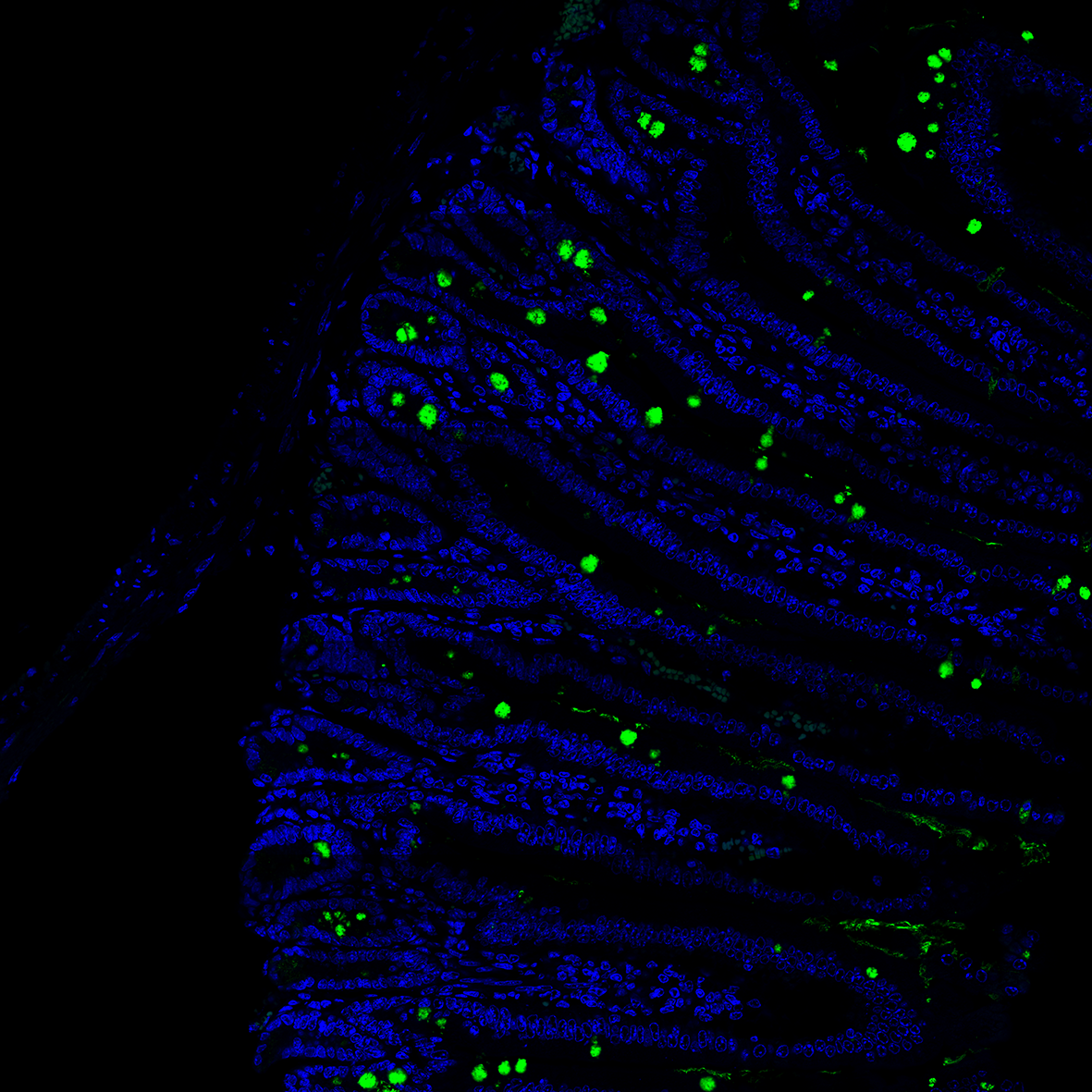

Supplement: Supplementary file 8 — Source data Fig. 5 [file 44318_2025_581_MOESM8_ESM.zip › Fig 5/5C/FAM134C KO MUC2.tif]

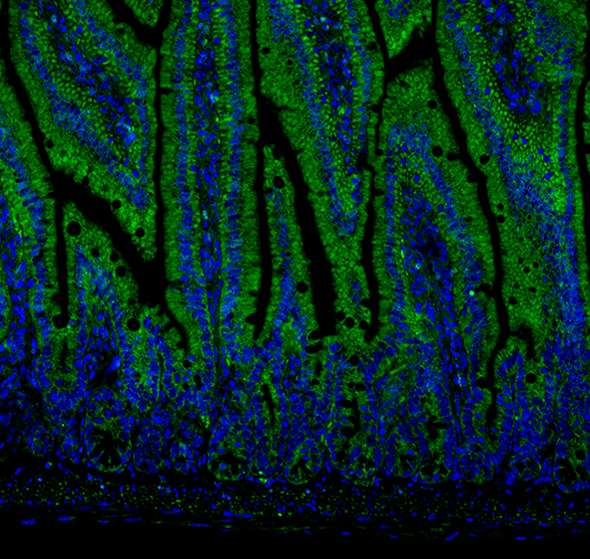

Supplement: Supplementary file 8 — Source data Fig. 5 [file 44318_2025_581_MOESM8_ESM.zip › Fig 5/5C/FAM134C WT p-S158 intestine.tif]

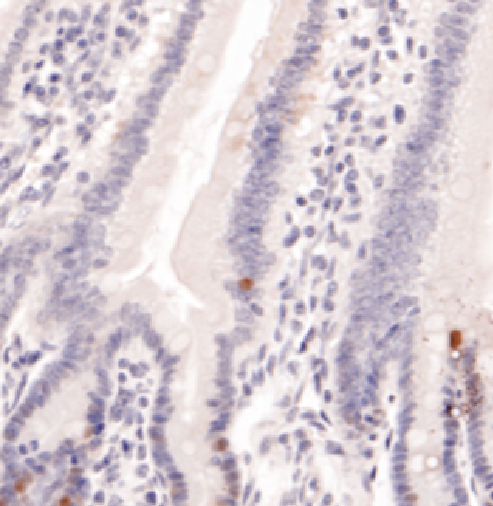

Supplement: Supplementary file 8 — Source data Fig. 5 [file 44318_2025_581_MOESM8_ESM.zip › Fig 5/5E/FAM134C KO Sox9 intestine.tif]

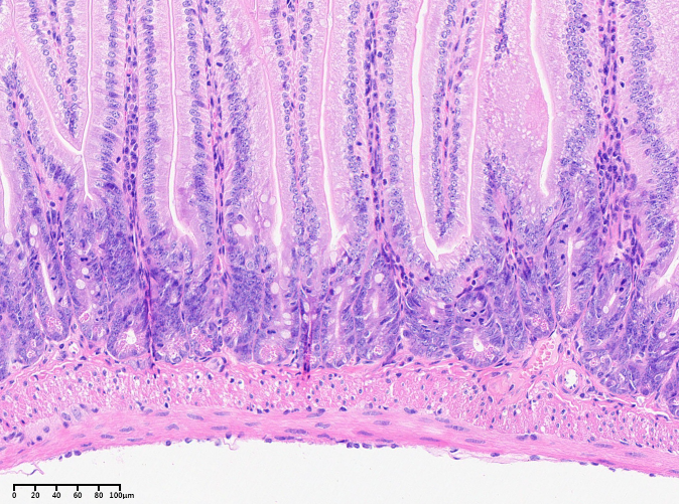

Supplement: Supplementary file 8 — Source data Fig. 5 [file 44318_2025_581_MOESM8_ESM.zip › Fig 5/5E/FAM134C KO HE intestine.tif]

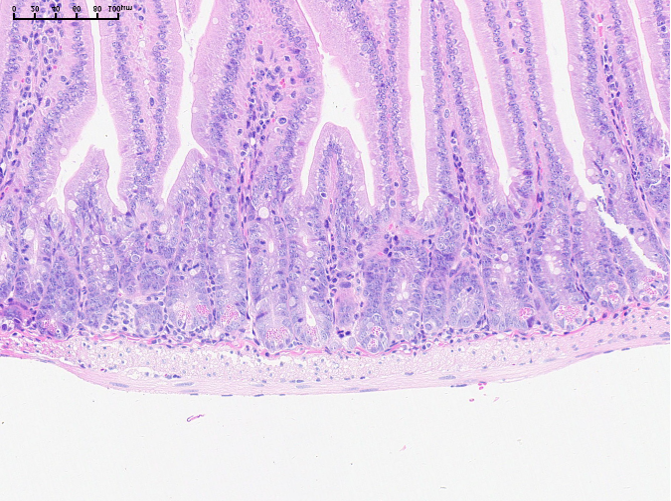

Supplement: Supplementary file 8 — Source data Fig. 5 [file 44318_2025_581_MOESM8_ESM.zip › Fig 5/5E/FAM134C WT HE intestine.tif]

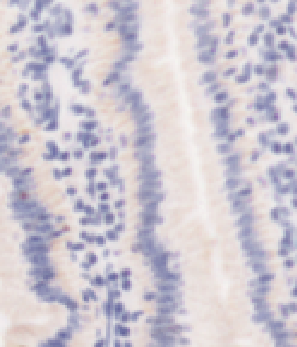

Supplement: Supplementary file 8 — Source data Fig. 5 [file 44318_2025_581_MOESM8_ESM.zip › Fig 5/5E/FAM134C WT Sox9 intestine.tif]

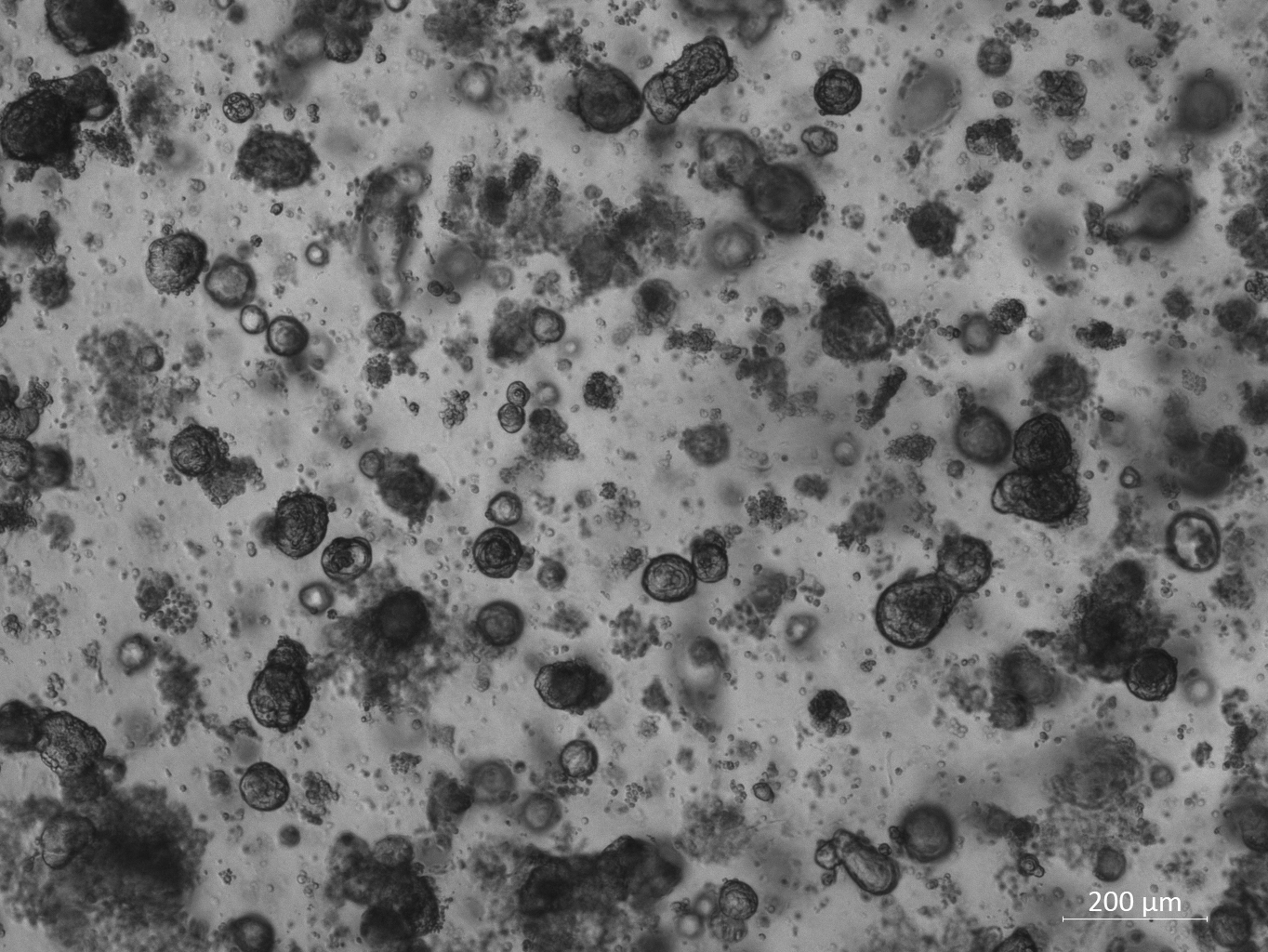

Supplement: Supplementary file 9 — Source data Fig. 6 [file 44318_2025_581_MOESM9_ESM.zip › Fig 6/6A/FAM134C KO day4-2.tif]

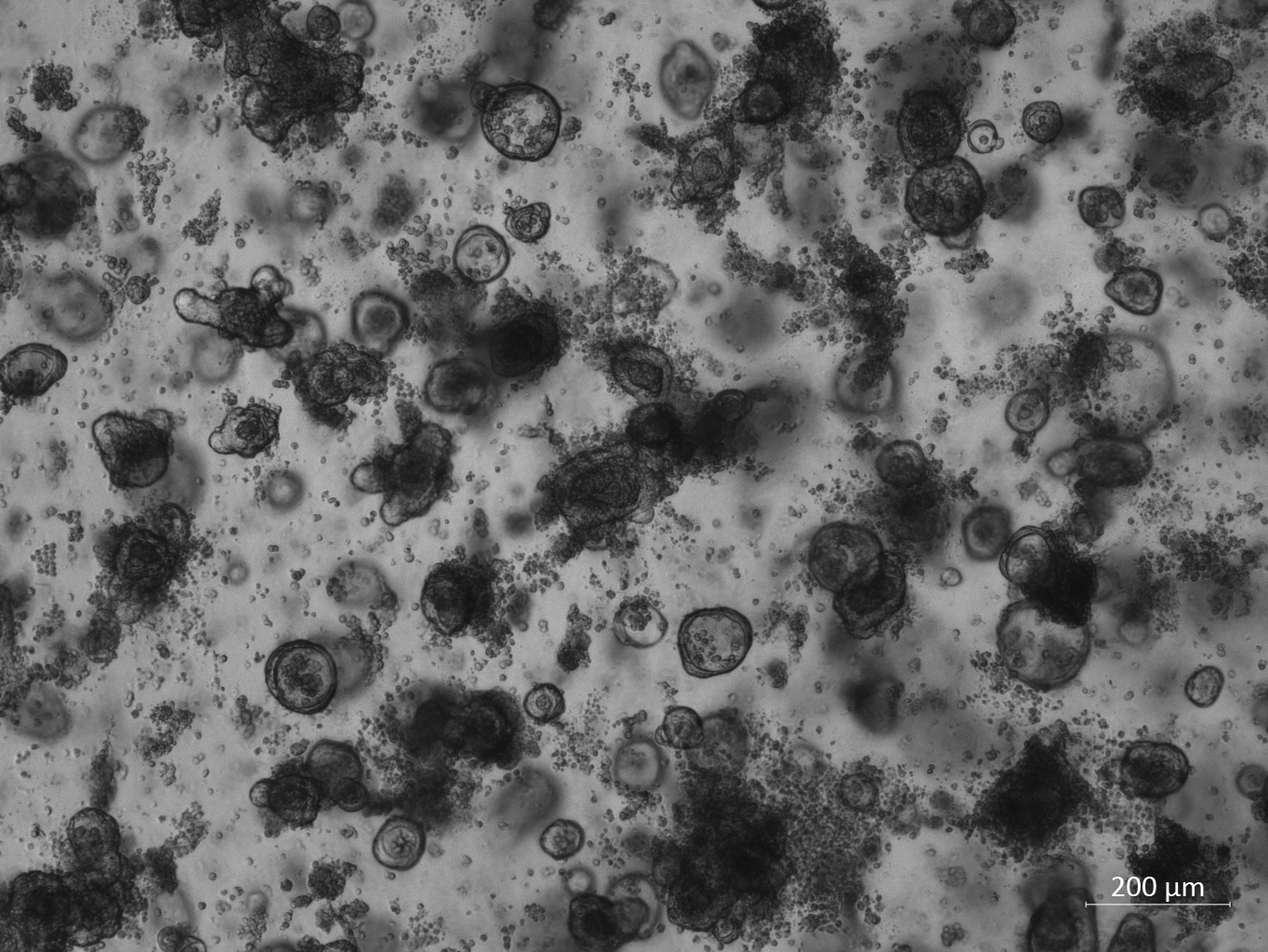

Supplement: Supplementary file 9 — Source data Fig. 6 [file 44318_2025_581_MOESM9_ESM.zip › Fig 6/6A/FAM134C KO day4-1.tif]

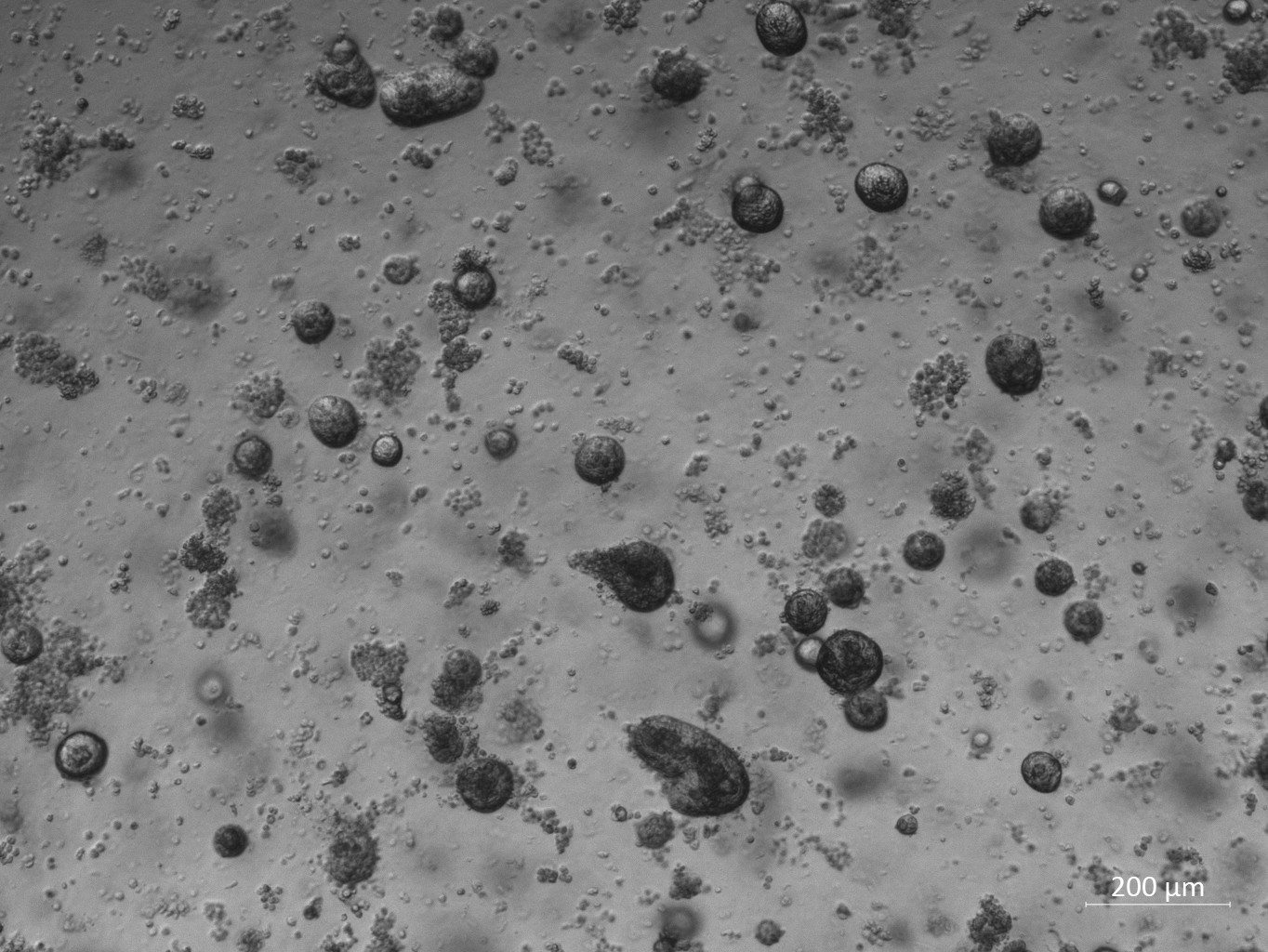

Supplement: Supplementary file 9 — Source data Fig. 6 [file 44318_2025_581_MOESM9_ESM.zip › Fig 6/6A/FAM134C WT day1-2.tif]

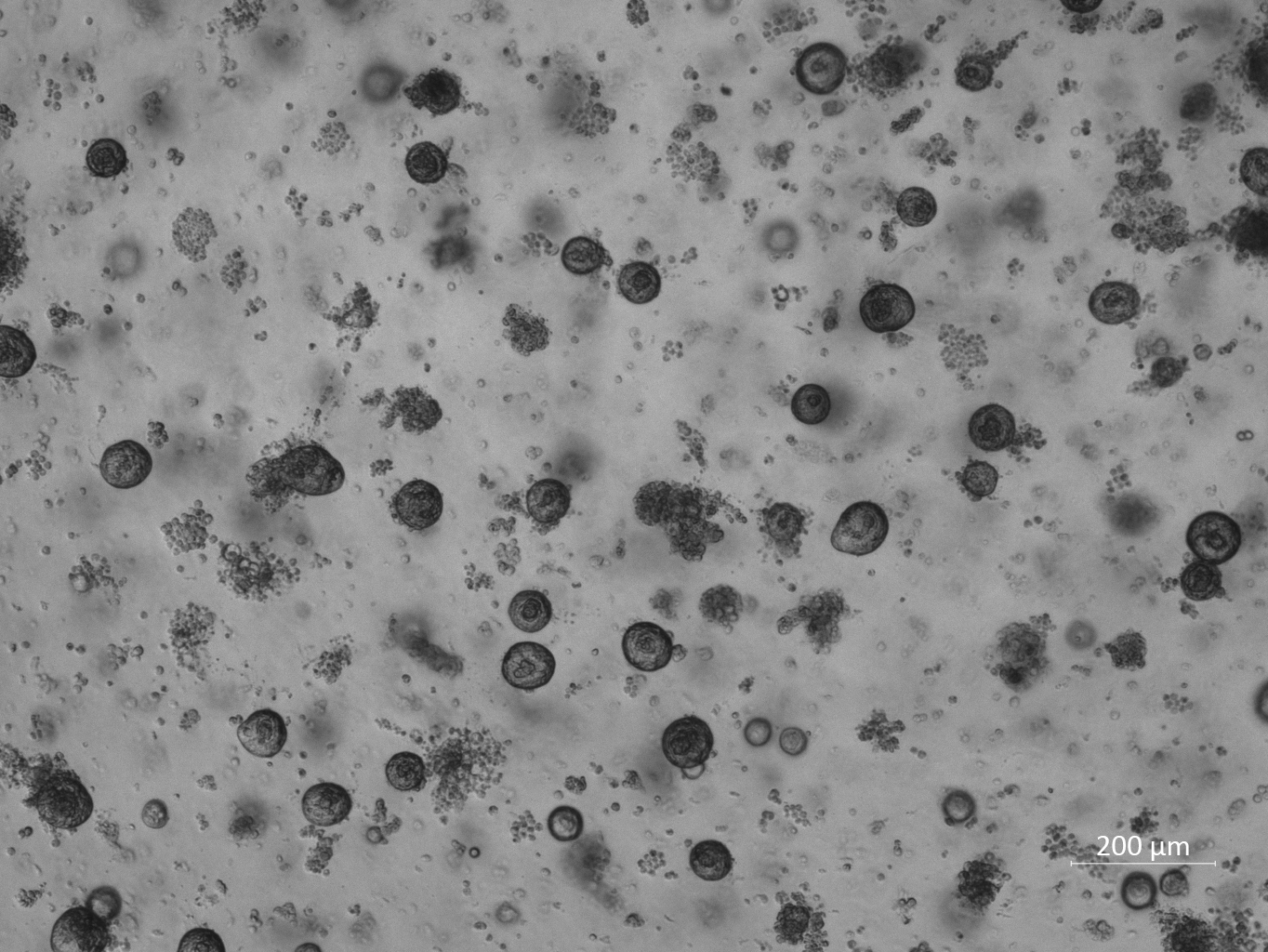

Supplement: Supplementary file 9 — Source data Fig. 6 [file 44318_2025_581_MOESM9_ESM.zip › Fig 6/6A/FAM134C WT day1-1.tif]

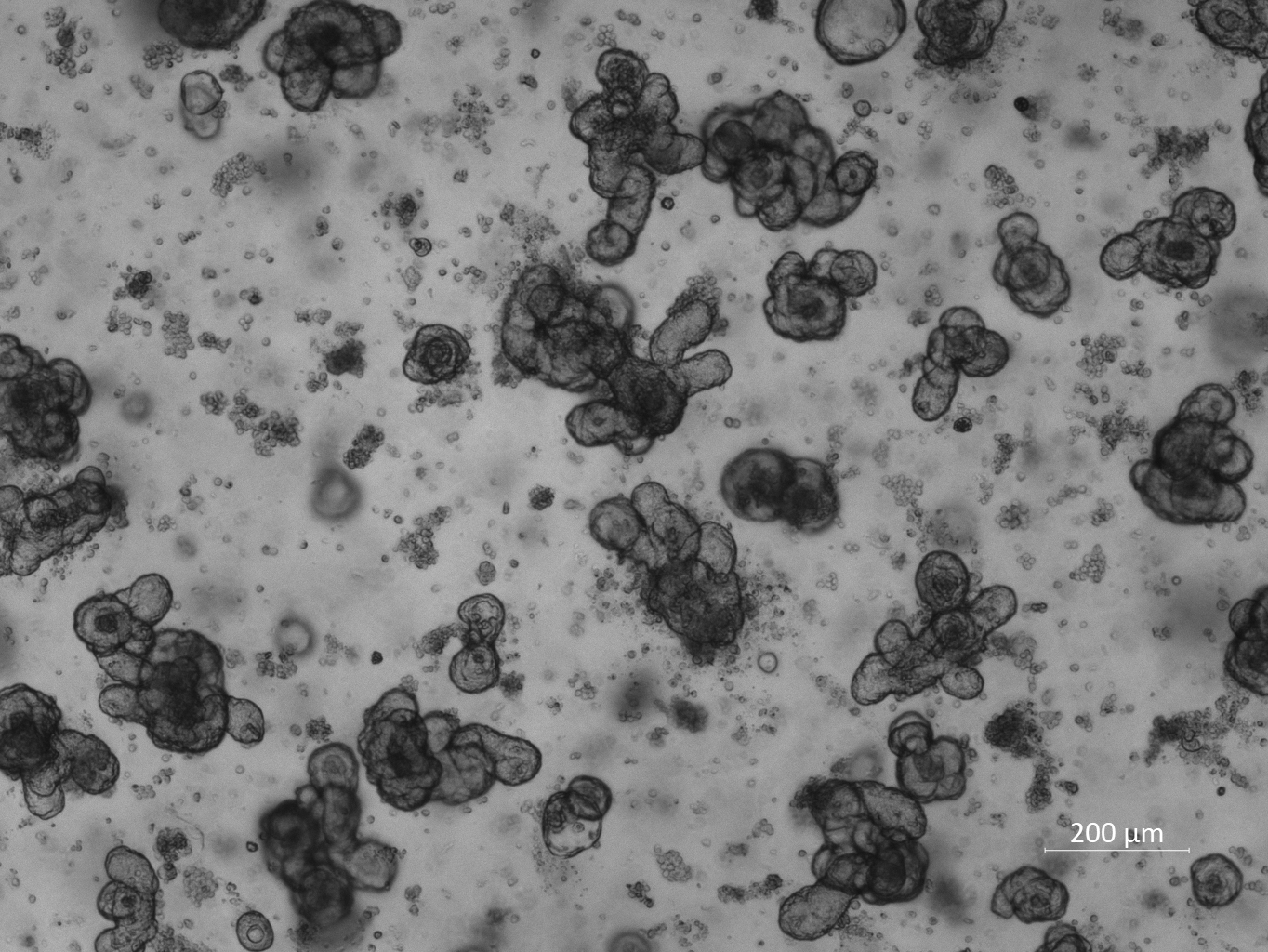

Supplement: Supplementary file 9 — Source data Fig. 6 [file 44318_2025_581_MOESM9_ESM.zip › Fig 6/6A/FAM134C WT day4-2.tif]

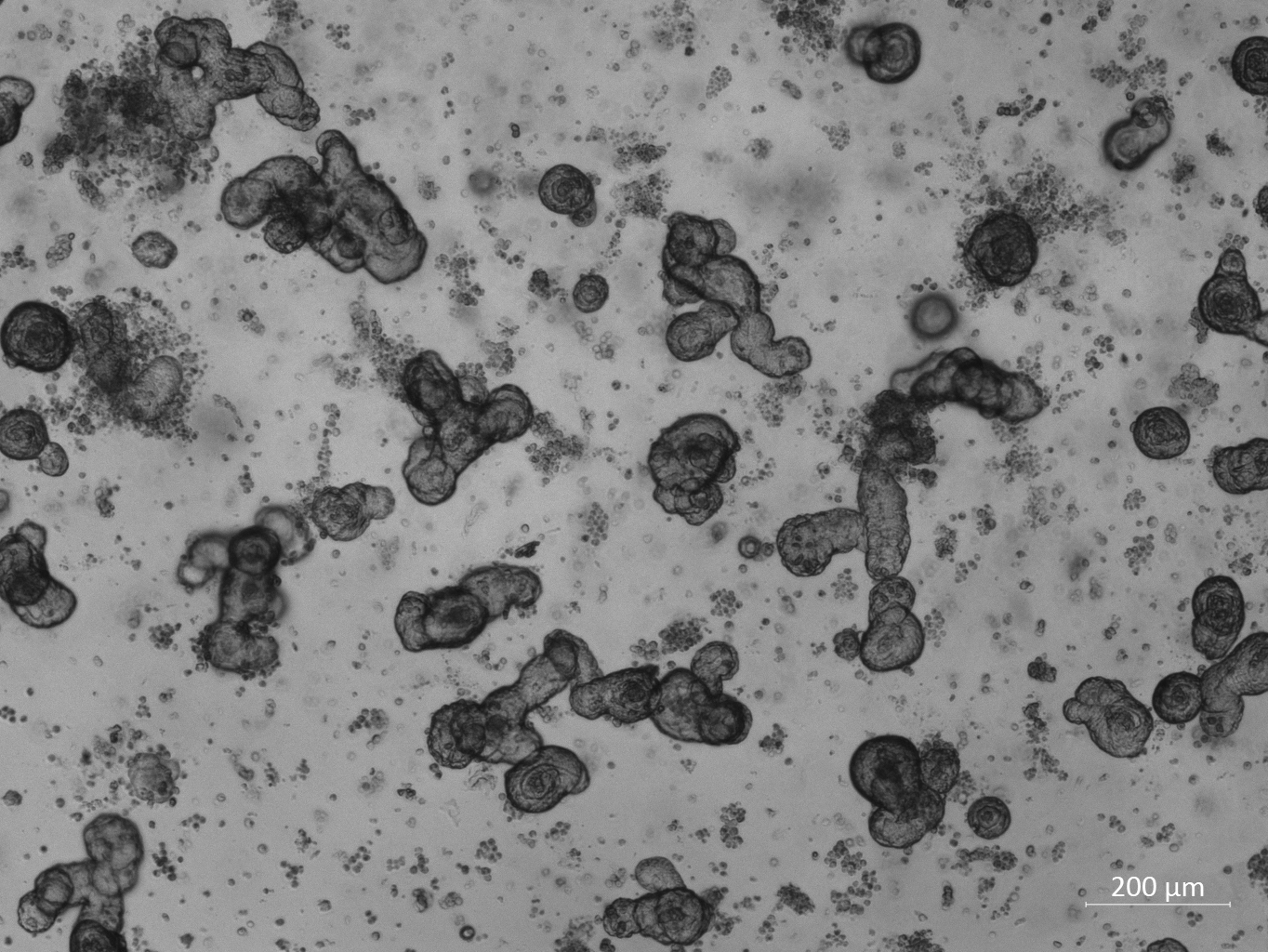

Supplement: Supplementary file 9 — Source data Fig. 6 [file 44318_2025_581_MOESM9_ESM.zip › Fig 6/6A/FAM134C WT day4-1.tif]

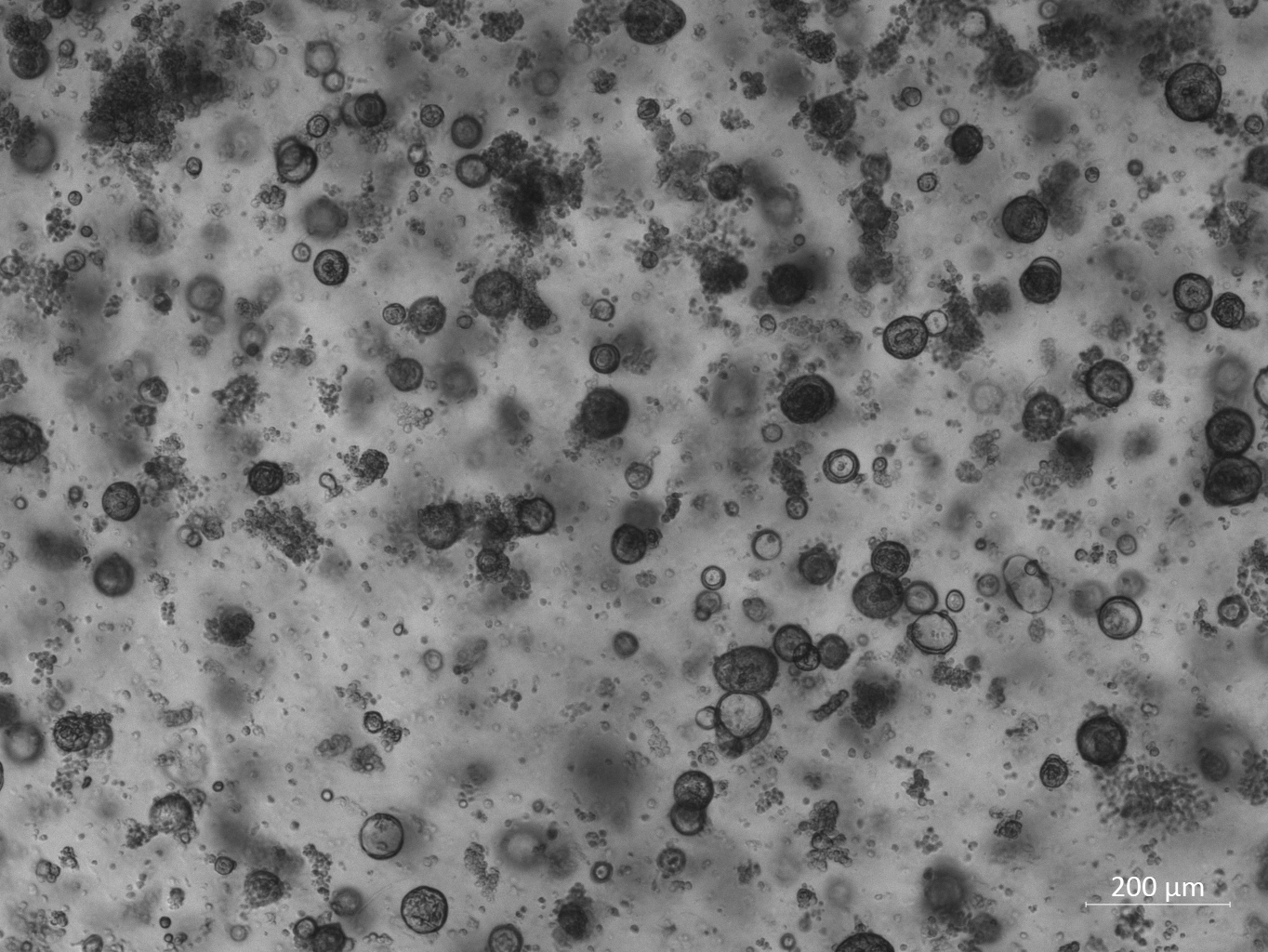

Supplement: Supplementary file 9 — Source data Fig. 6 [file 44318_2025_581_MOESM9_ESM.zip › Fig 6/6A/FAM134C KO day1-2.tif]

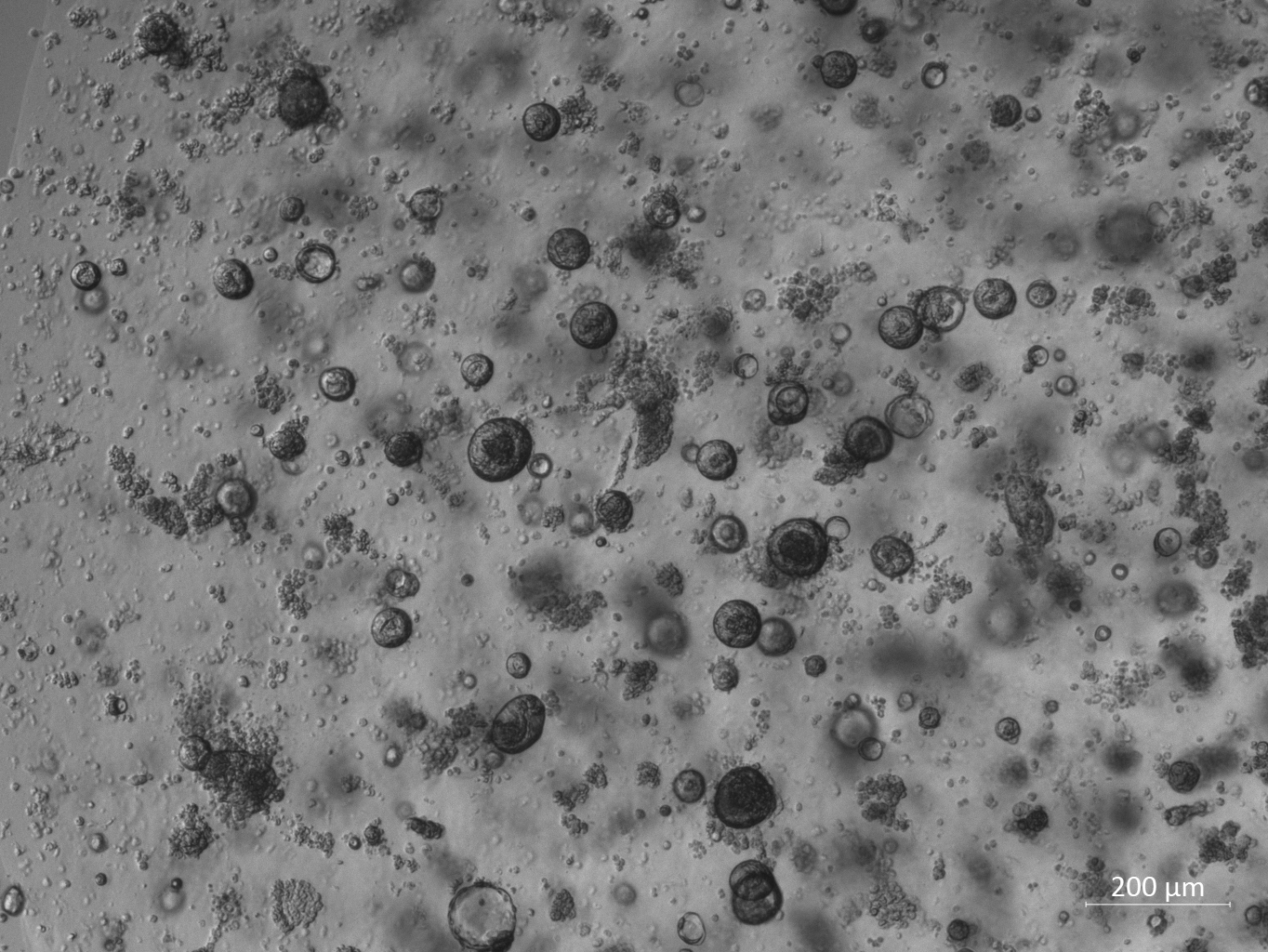

Supplement: Supplementary file 9 — Source data Fig. 6 [file 44318_2025_581_MOESM9_ESM.zip › Fig 6/6A/FAM134C KO day1-1.tif]

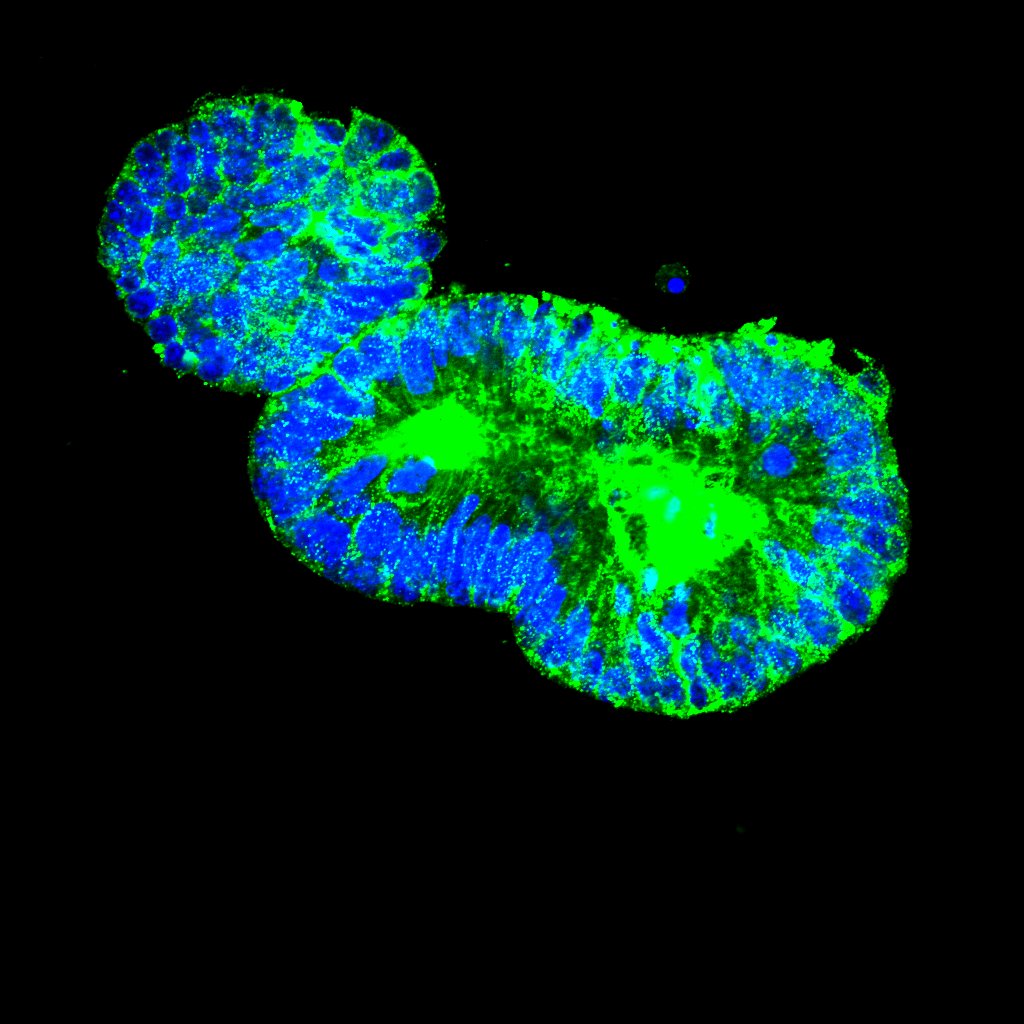

Supplement: Supplementary file 9 — Source data Fig. 6 [file 44318_2025_581_MOESM9_ESM.zip › Fig 6/6B /FAM134C KO organoid BMPR1a merged.tif]

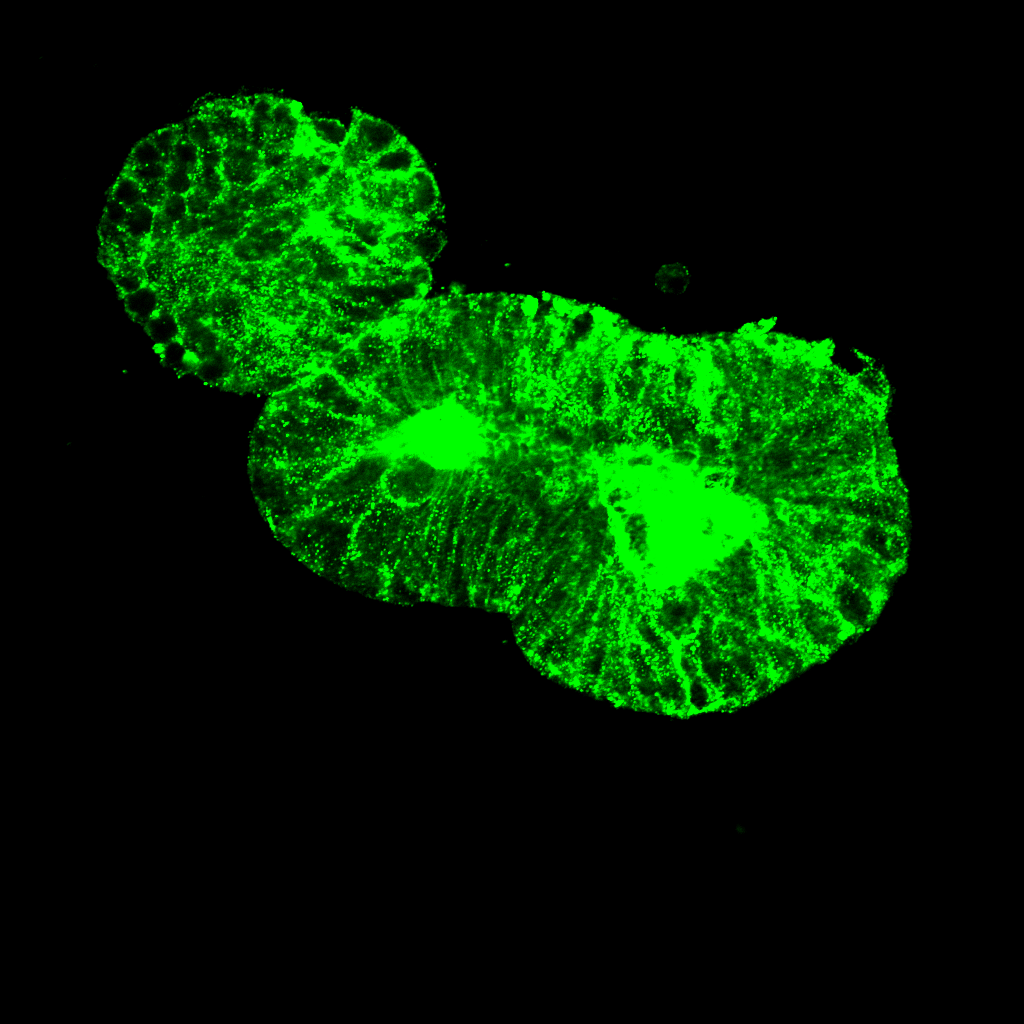

Supplement: Supplementary file 9 — Source data Fig. 6 [file 44318_2025_581_MOESM9_ESM.zip › Fig 6/6B /FAM134C KO organoid bmpr1a-488.tif]

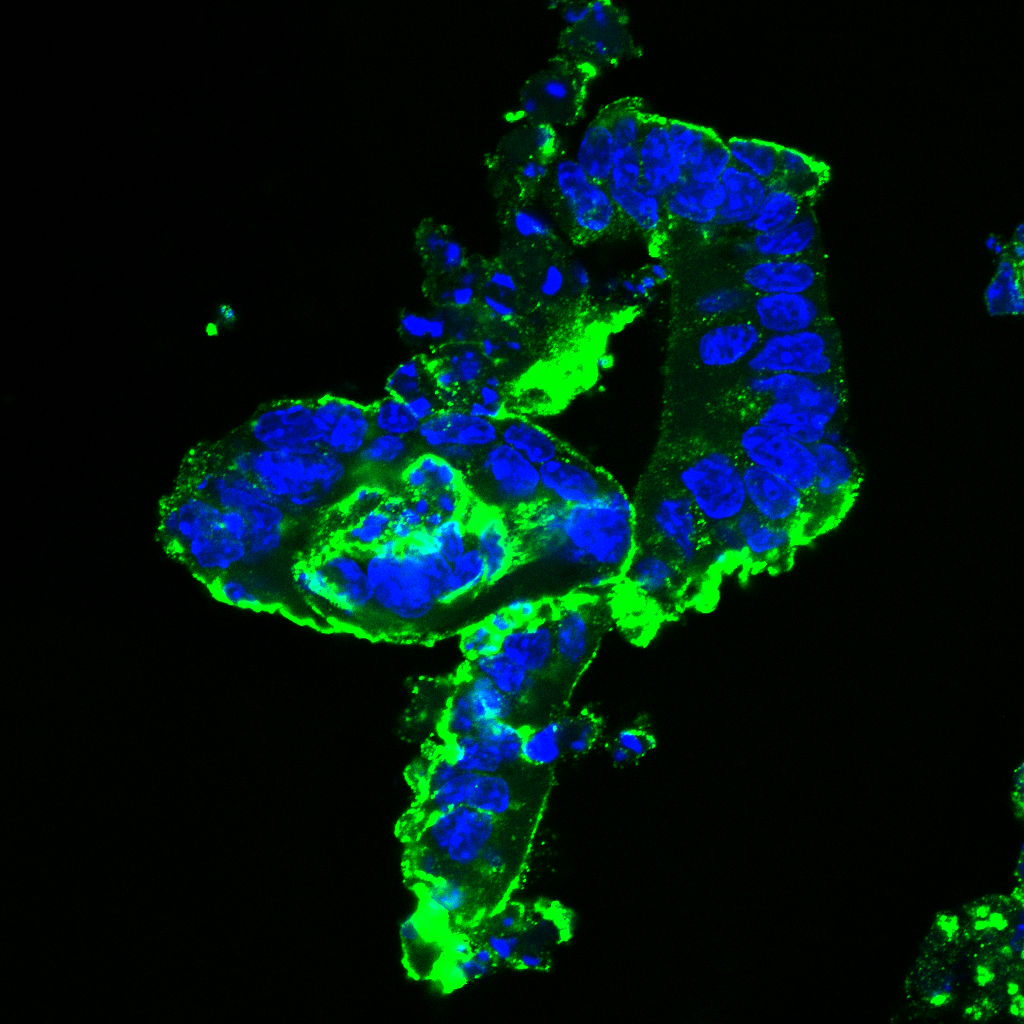

Supplement: Supplementary file 9 — Source data Fig. 6 [file 44318_2025_581_MOESM9_ESM.zip › Fig 6/6B /FAM134C WT organoid LGR5-488 merged.tif]

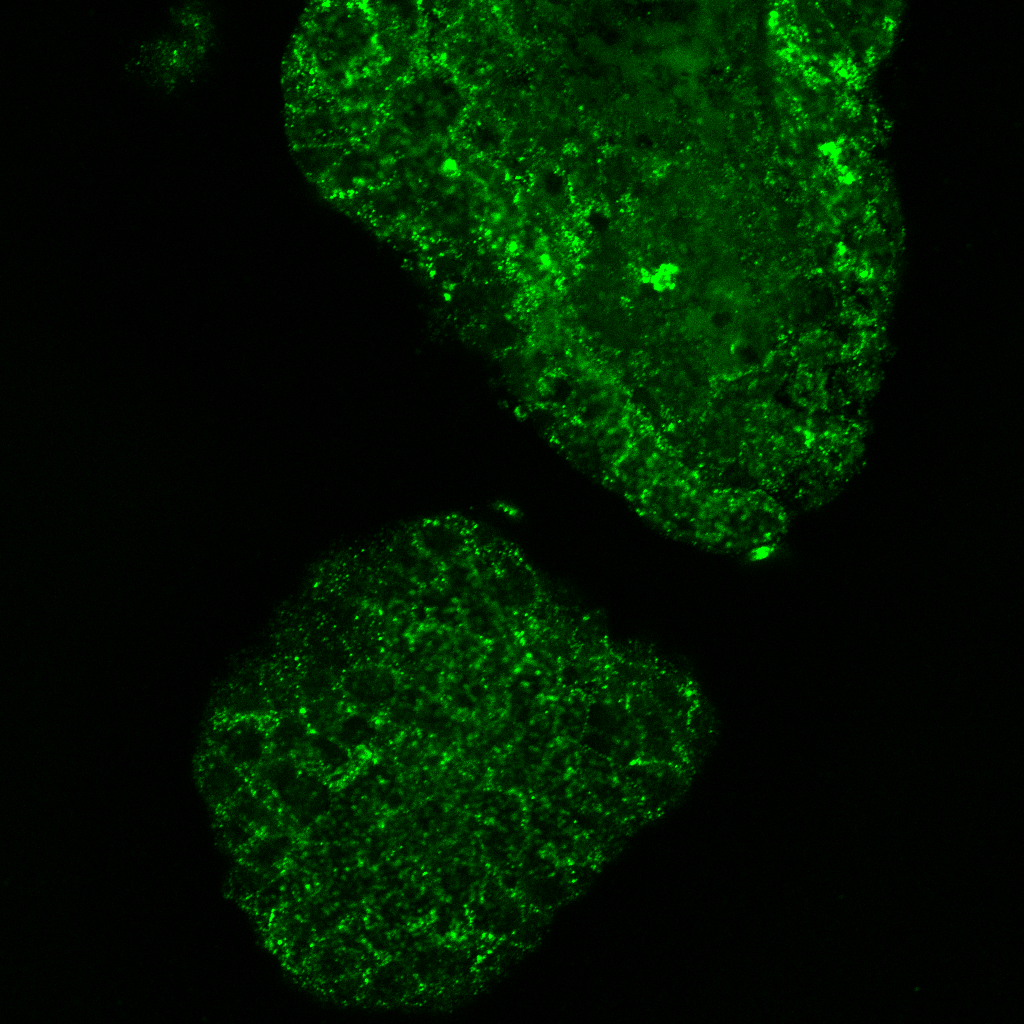

Supplement: Supplementary file 9 — Source data Fig. 6 [file 44318_2025_581_MOESM9_ESM.zip › Fig 6/6B /FAM134C KO organoid LGR5-488.tif]

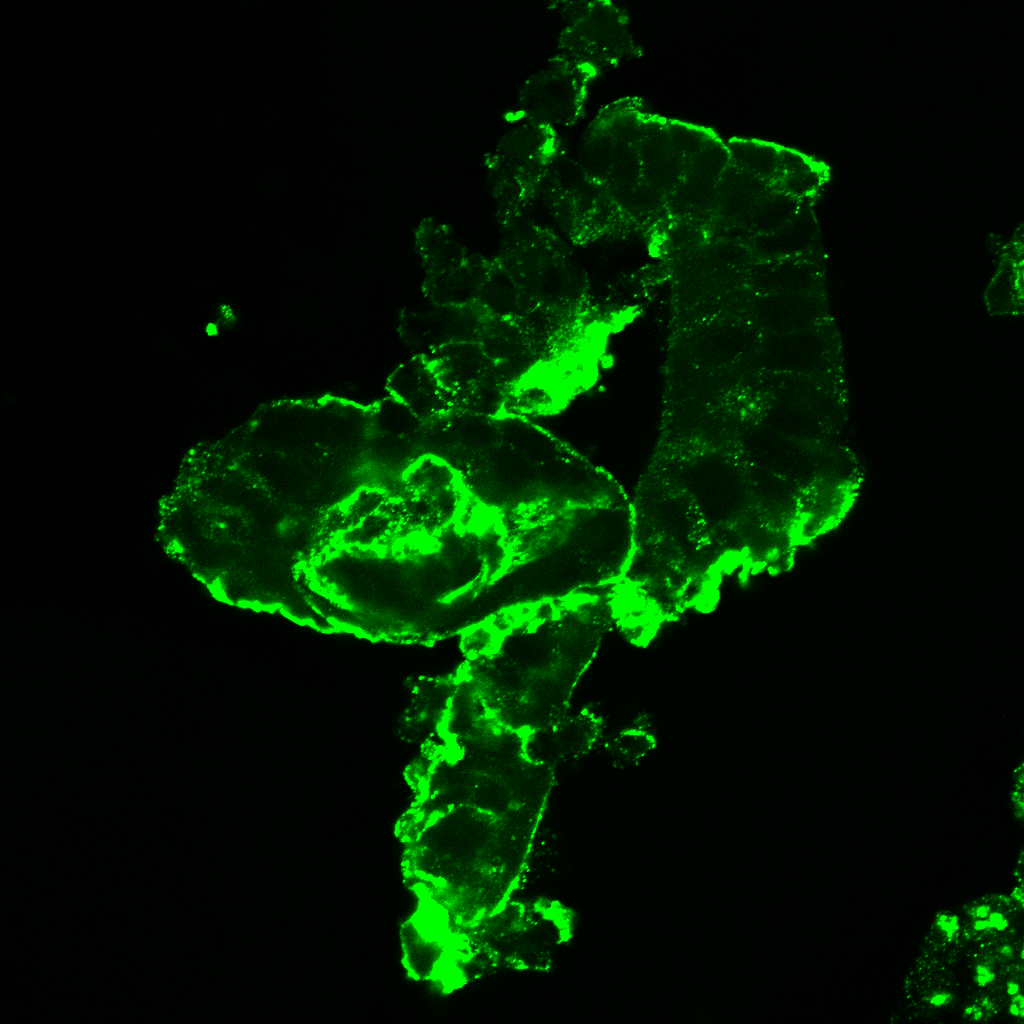

Supplement: Supplementary file 9 — Source data Fig. 6 [file 44318_2025_581_MOESM9_ESM.zip › Fig 6/6B /FAM134C WT organoid LGR5-488 .tif]

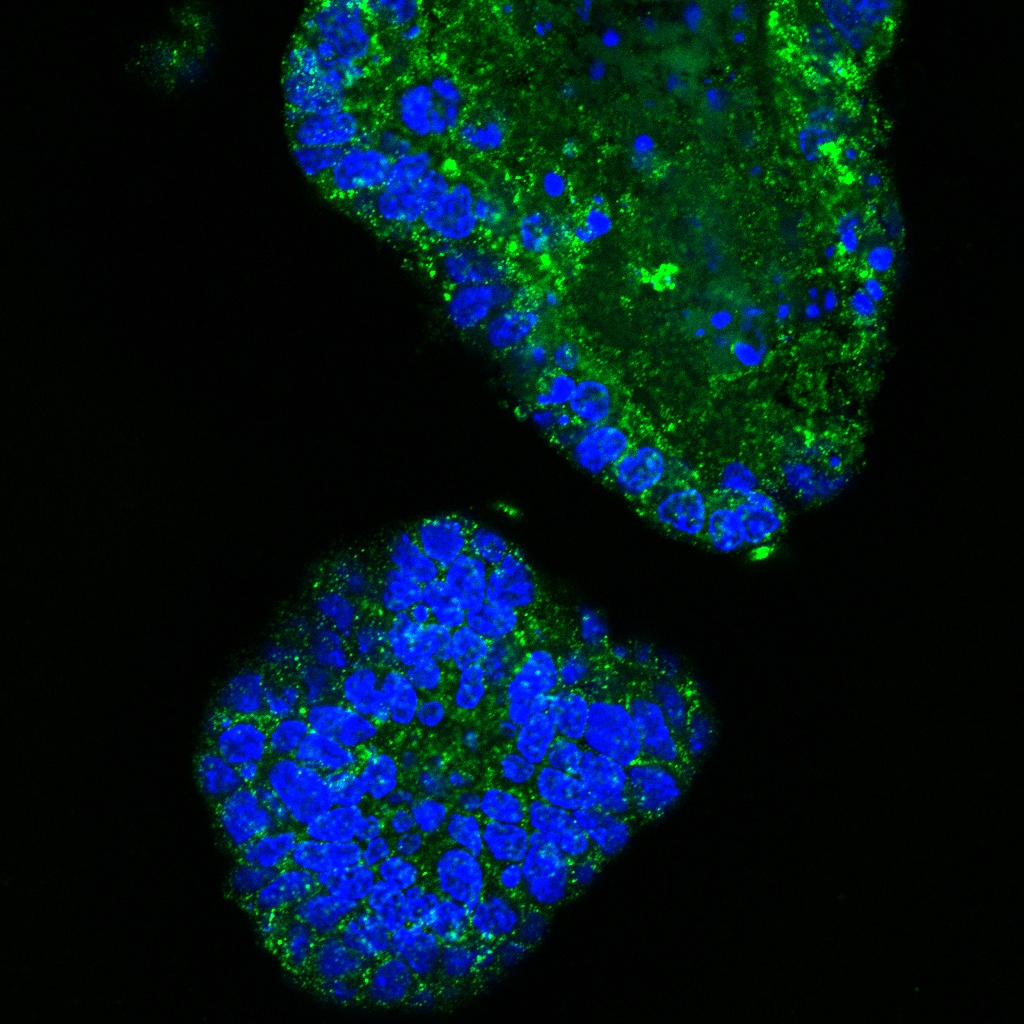

Supplement: Supplementary file 9 — Source data Fig. 6 [file 44318_2025_581_MOESM9_ESM.zip › Fig 6/6B /FAM134C KO organoid LGR5-488 merged.tif]

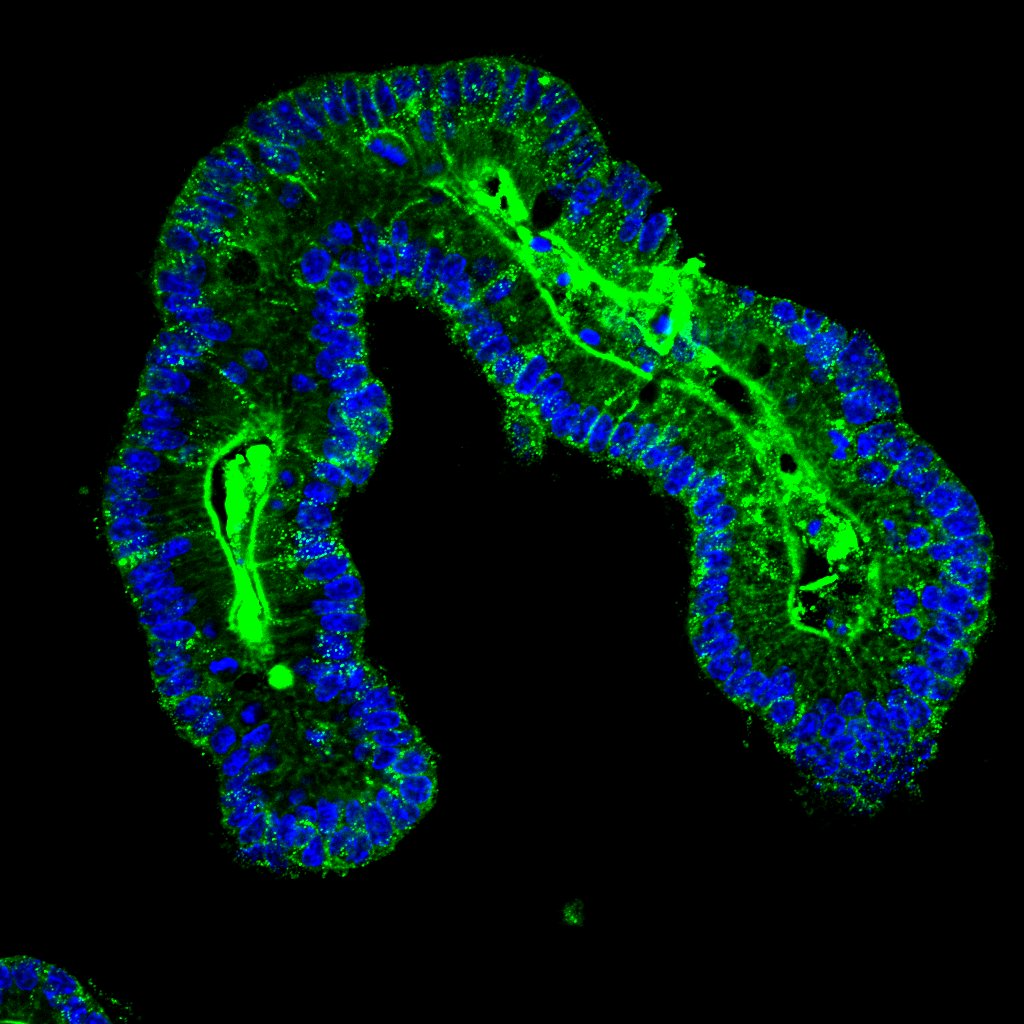

Supplement: Supplementary file 9 — Source data Fig. 6 [file 44318_2025_581_MOESM9_ESM.zip › Fig 6/6B /FAM134C WT organoid BMPR1a merged.tiff]

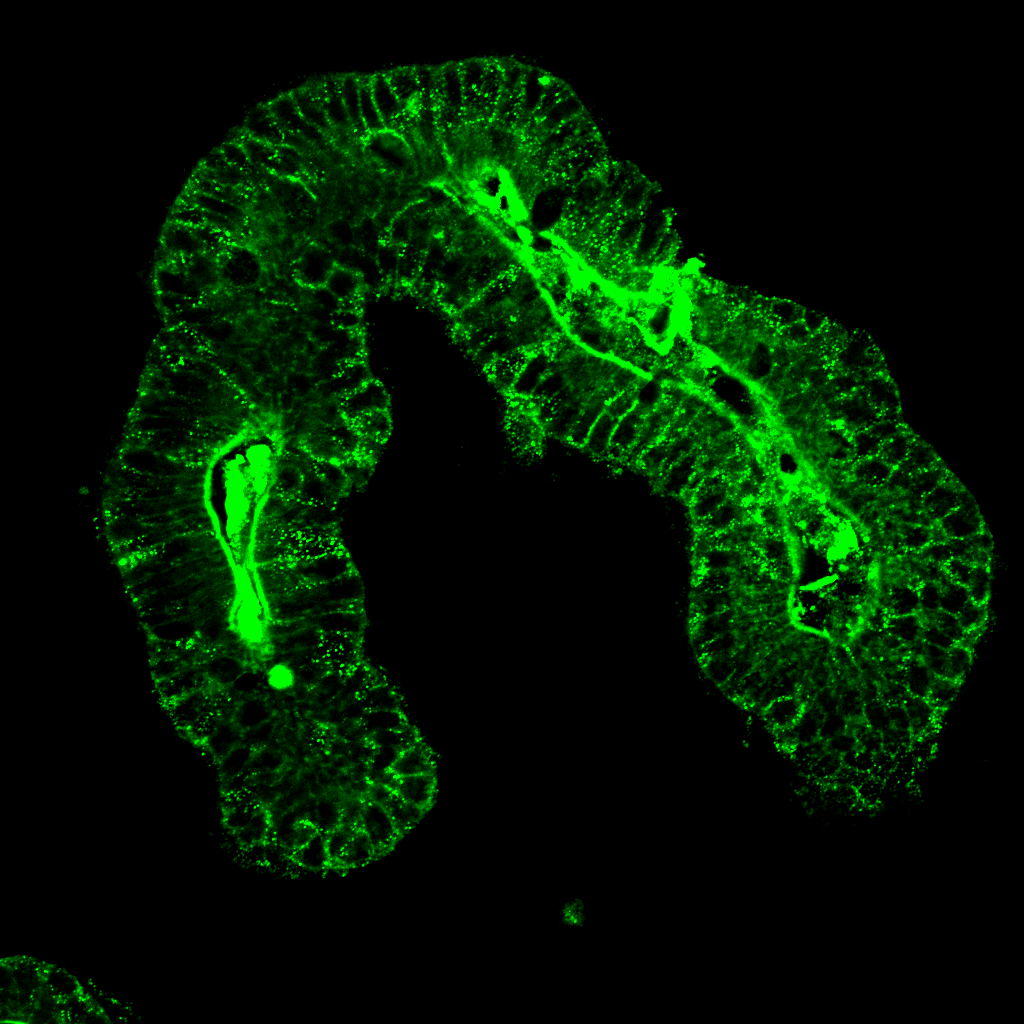

Supplement: Supplementary file 9 — Source data Fig. 6 [file 44318_2025_581_MOESM9_ESM.zip › Fig 6/6B /FAM134C WT organoid bmpr1a-488.tif]

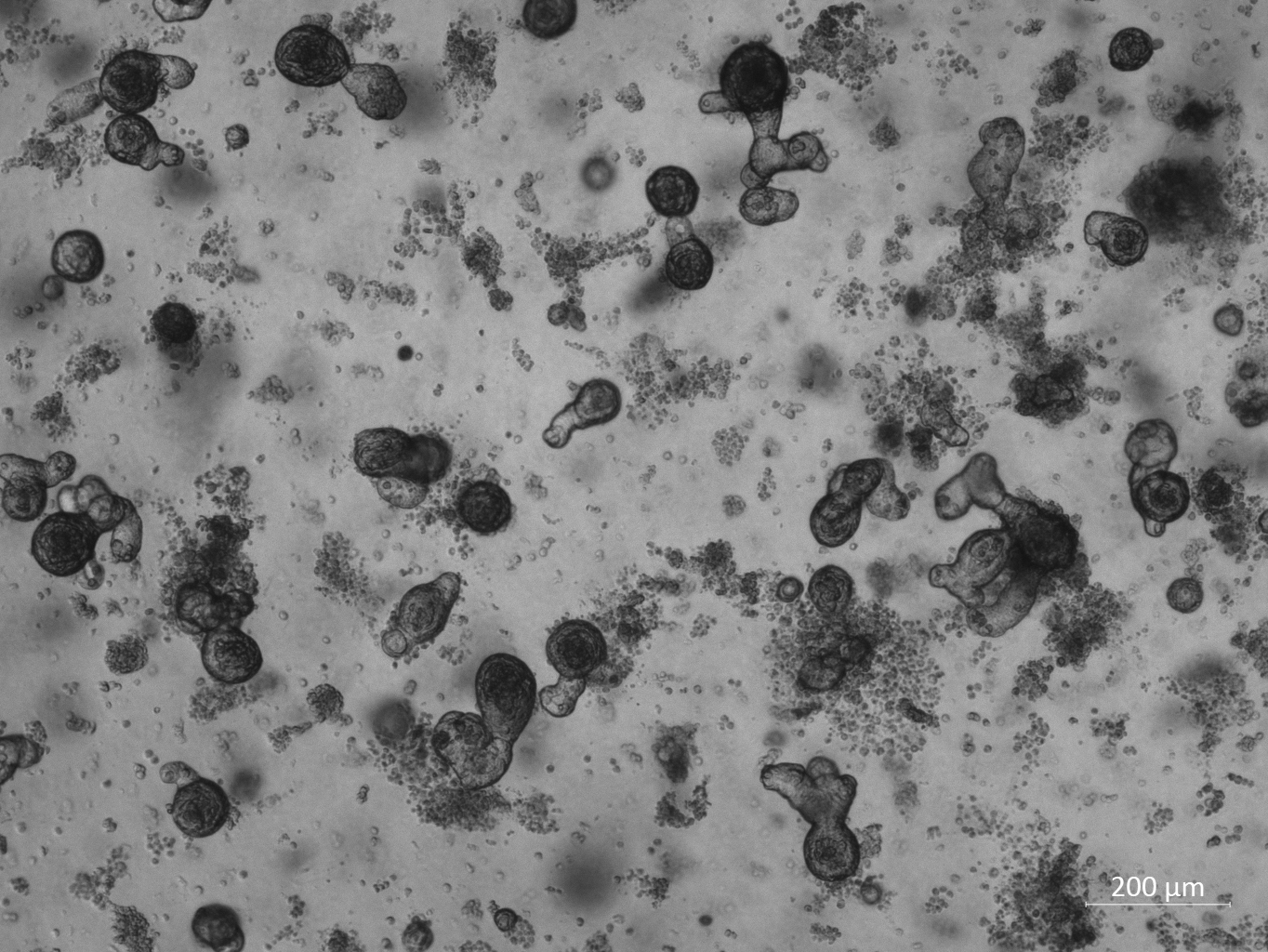

Supplement: Supplementary file 9 — Source data Fig. 6 [file 44318_2025_581_MOESM9_ESM.zip › Fig 6/6E/KO day4 noggin 500.tif]

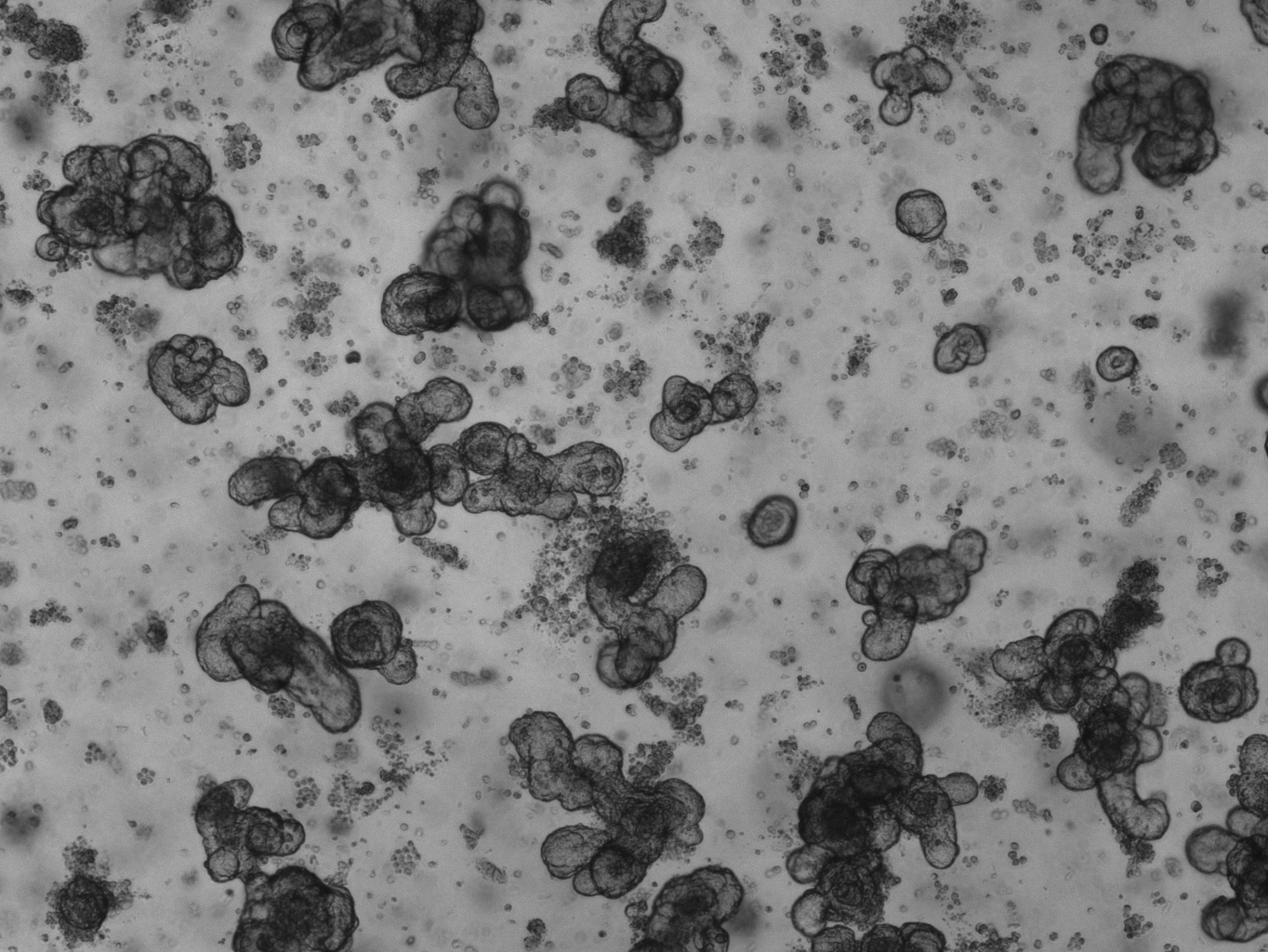

Supplement: Supplementary file 9 — Source data Fig. 6 [file 44318_2025_581_MOESM9_ESM.zip › Fig 6/6E/WT day4 noggin 700.tiff]

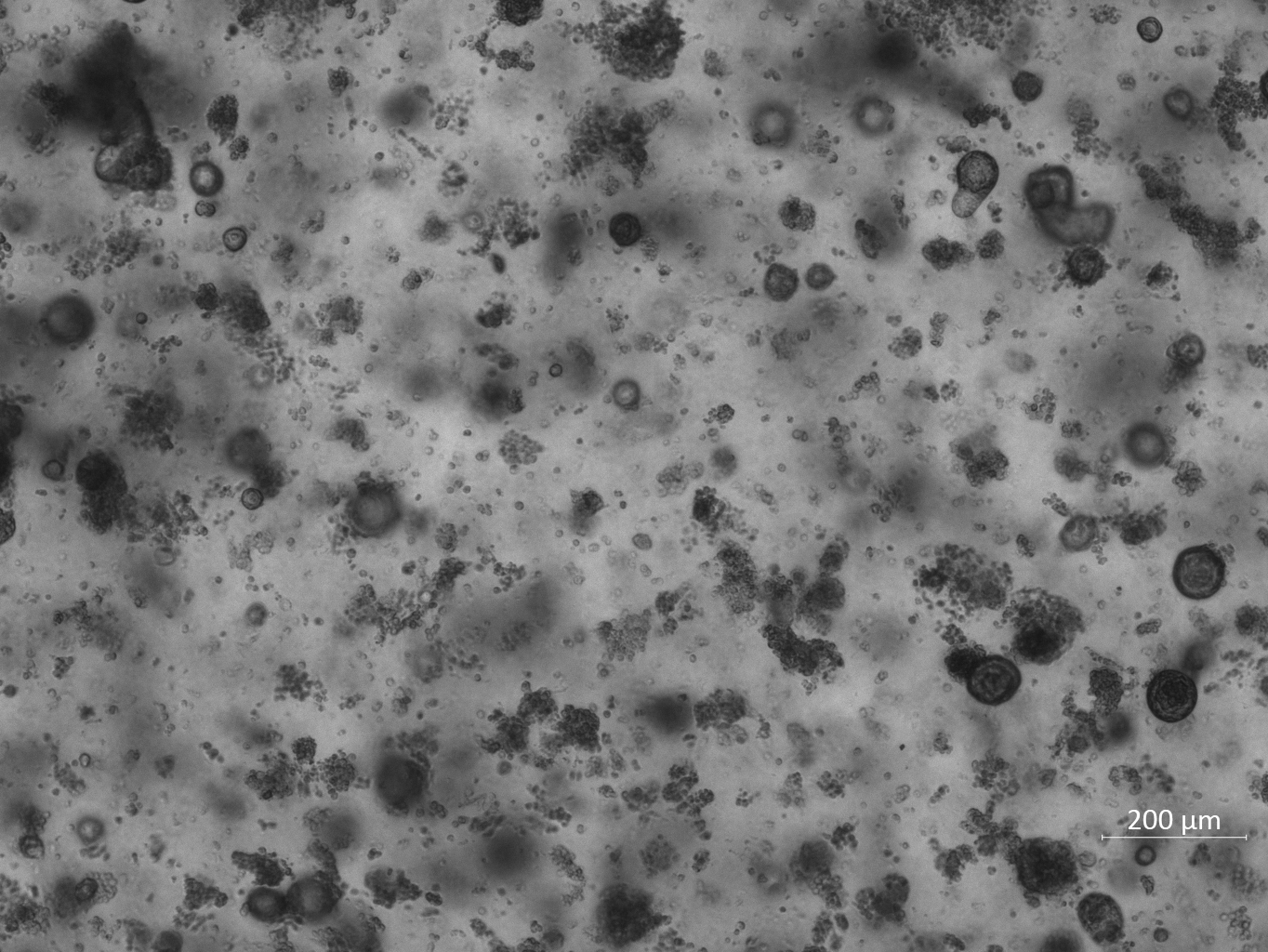

Supplement: Supplementary file 9 — Source data Fig. 6 [file 44318_2025_581_MOESM9_ESM.zip › Fig 6/6E/KO day4 noggin 50.tif]

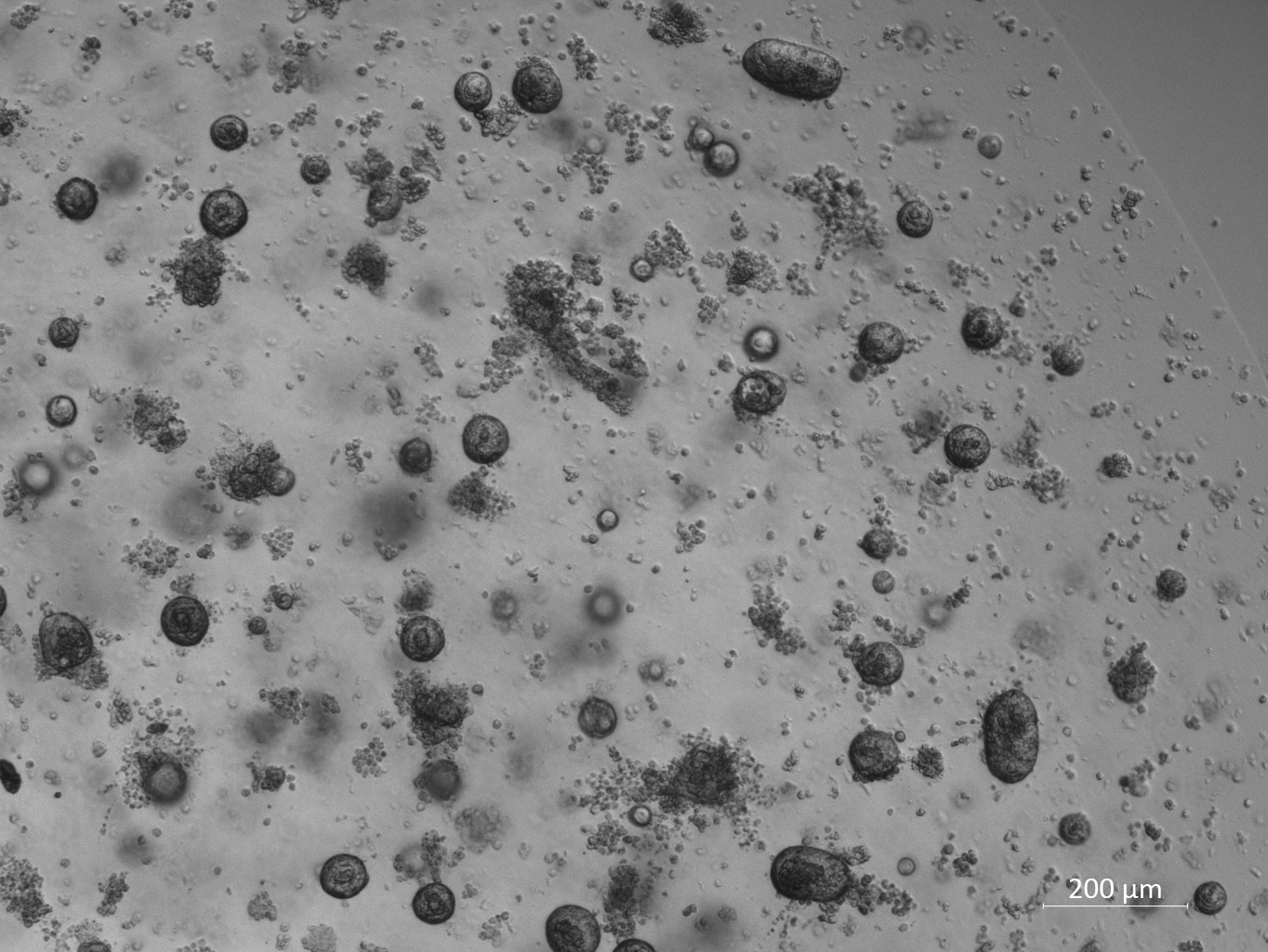

Supplement: Supplementary file 9 — Source data Fig. 6 [file 44318_2025_581_MOESM9_ESM.zip › Fig 6/6E/WT day1 noggin 0.tif]

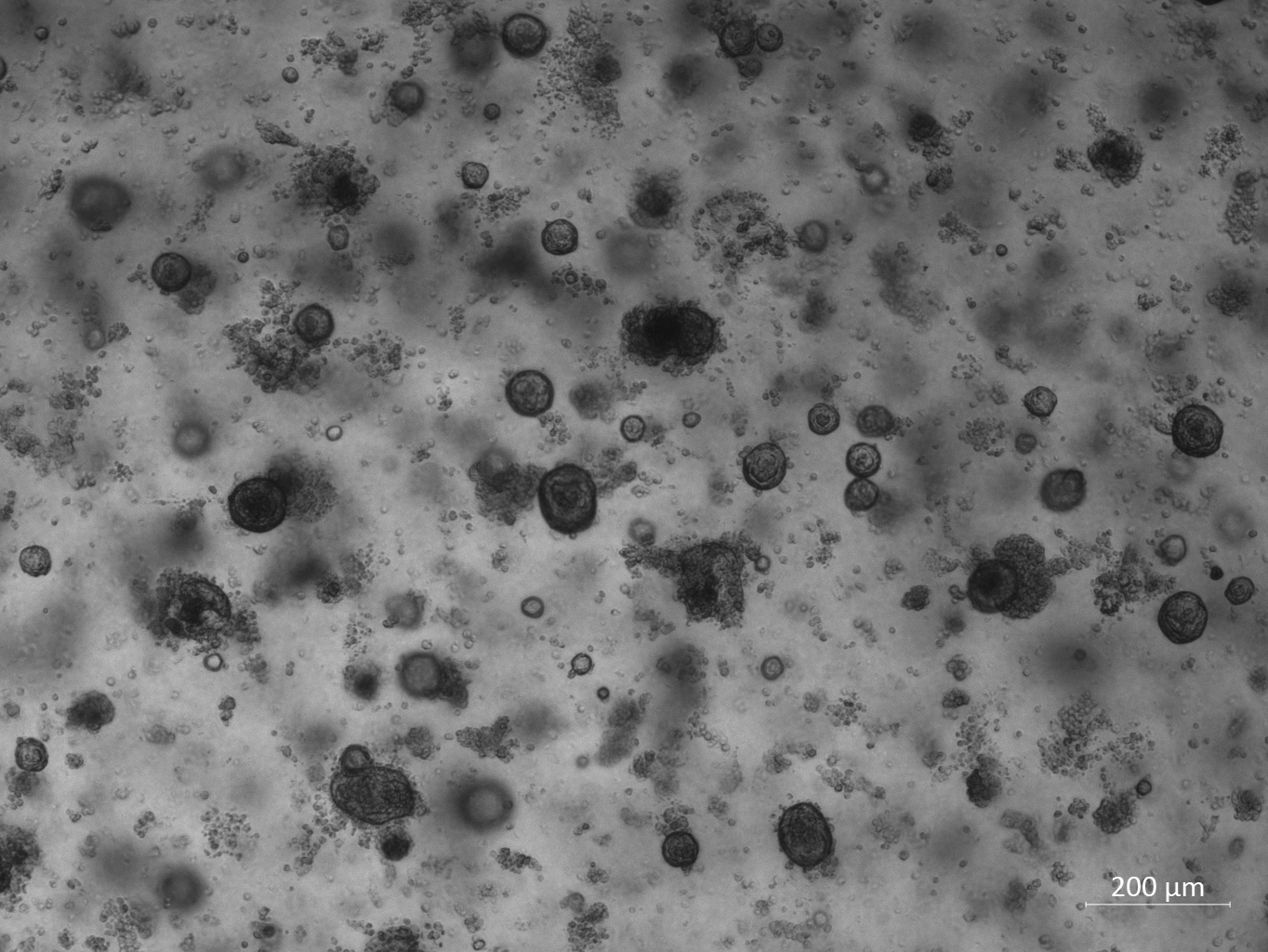

Supplement: Supplementary file 9 — Source data Fig. 6 [file 44318_2025_581_MOESM9_ESM.zip › Fig 6/6E/KO day4 noggin 100.tif]

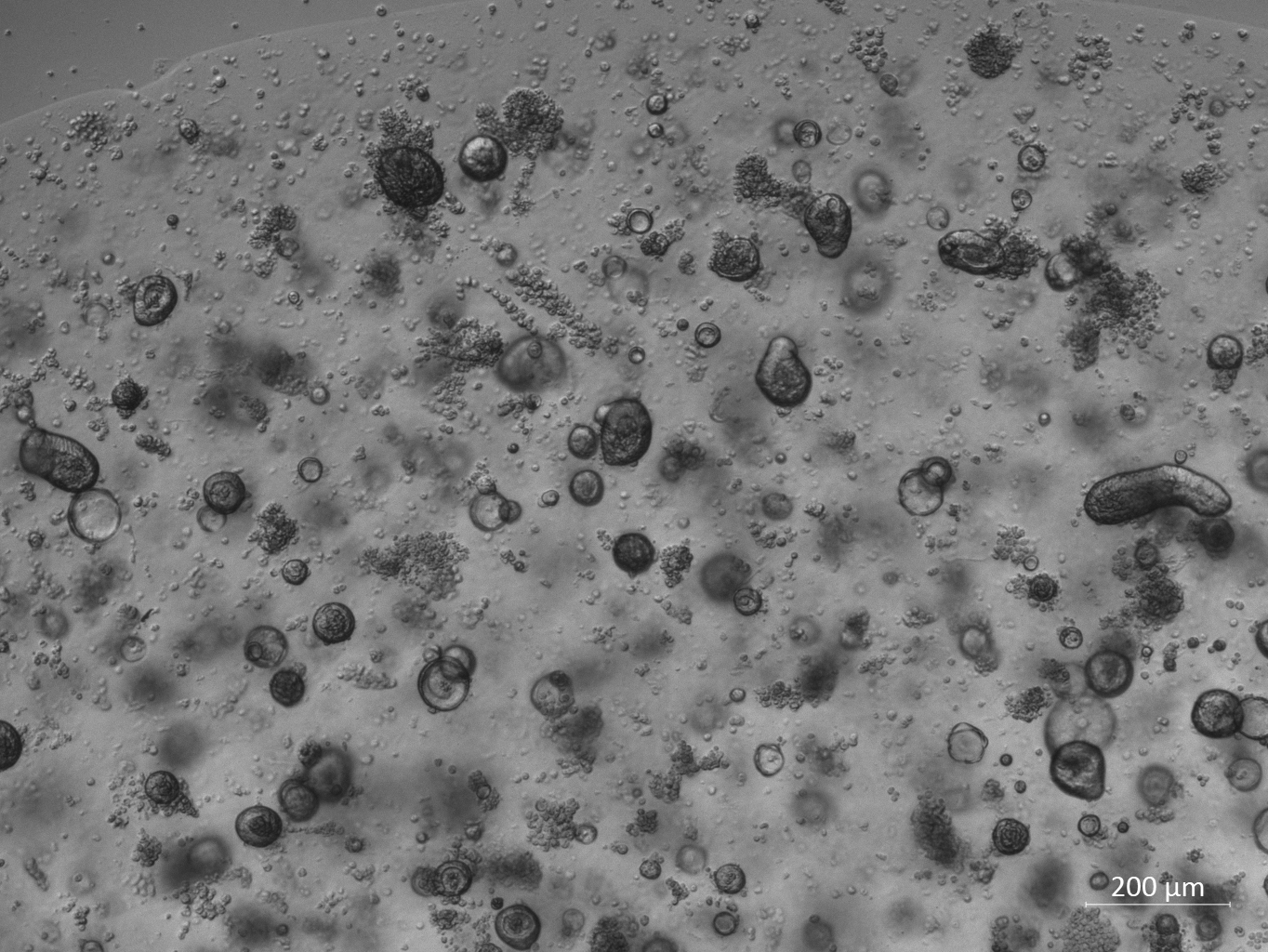

Supplement: Supplementary file 9 — Source data Fig. 6 [file 44318_2025_581_MOESM9_ESM.zip › Fig 6/6E/KO day1 noggin 100.tif]

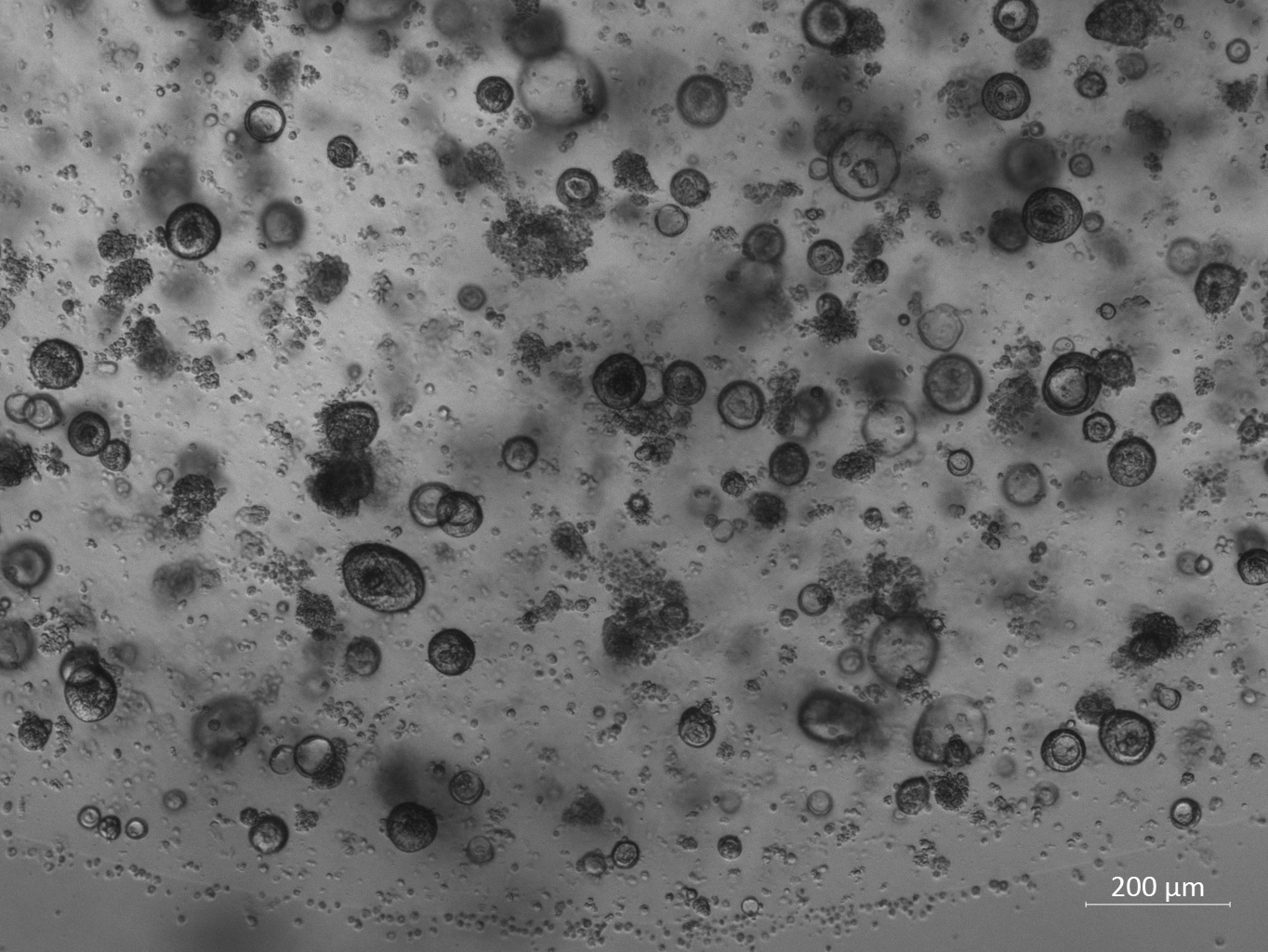

Supplement: Supplementary file 9 — Source data Fig. 6 [file 44318_2025_581_MOESM9_ESM.zip › Fig 6/6E/WT day1 noggin 50.tif]

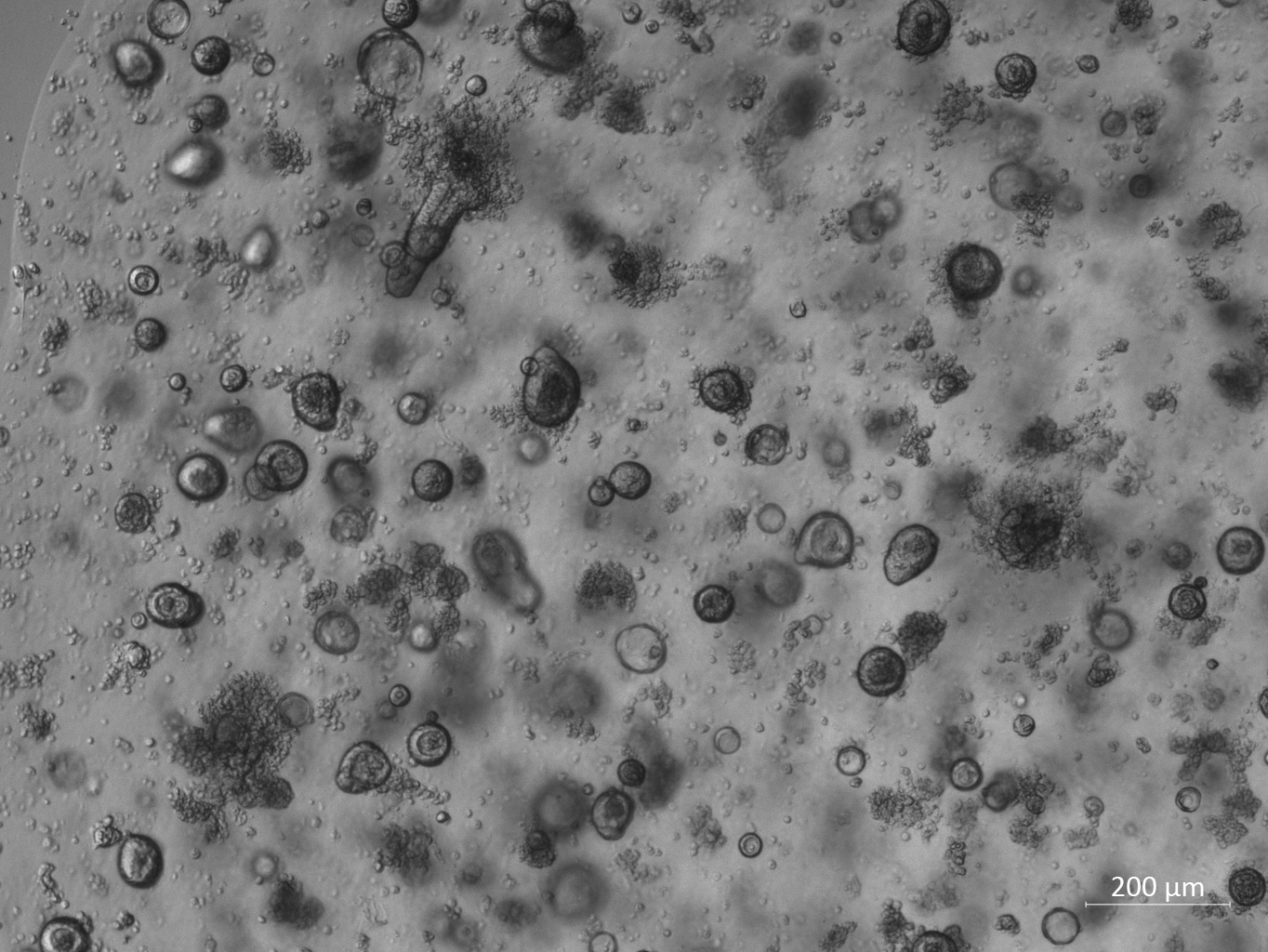

Supplement: Supplementary file 9 — Source data Fig. 6 [file 44318_2025_581_MOESM9_ESM.zip › Fig 6/6E/KO day1 noggin 500.tif]

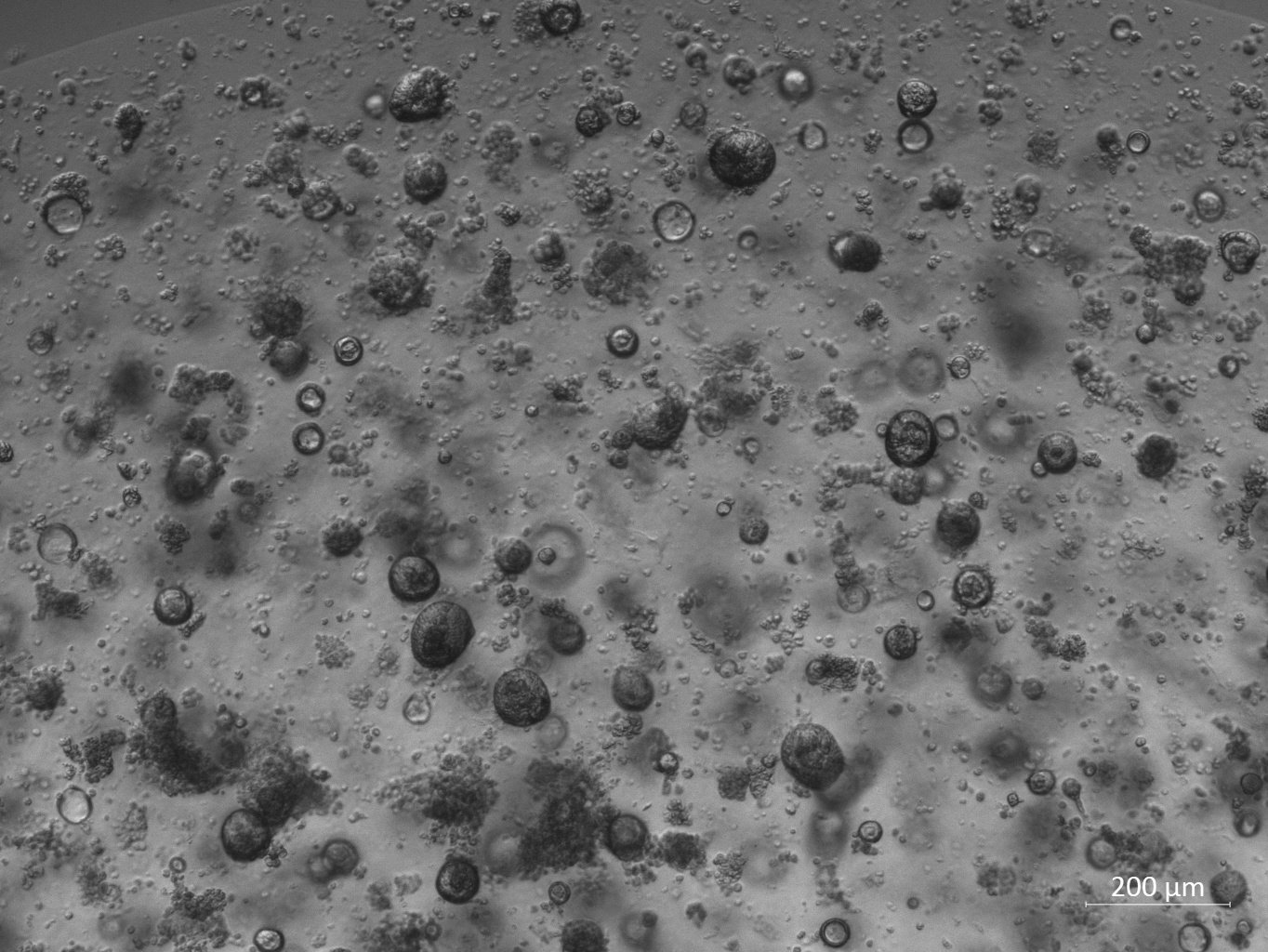

Supplement: Supplementary file 9 — Source data Fig. 6 [file 44318_2025_581_MOESM9_ESM.zip › Fig 6/6E/KO day1 noggin 0.tif]

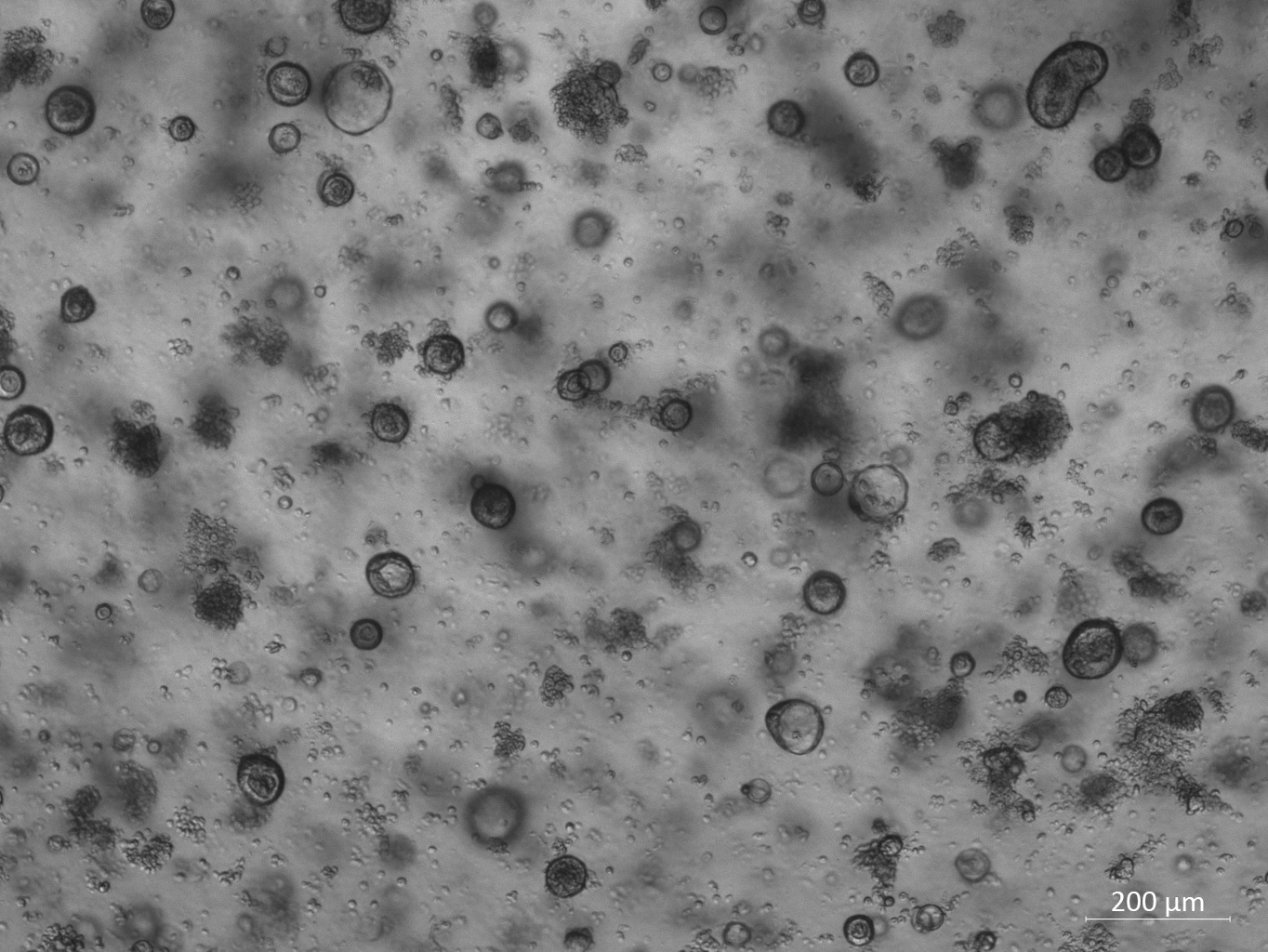

Supplement: Supplementary file 9 — Source data Fig. 6 [file 44318_2025_581_MOESM9_ESM.zip › Fig 6/6E/KO day1 noggin 700.tif]

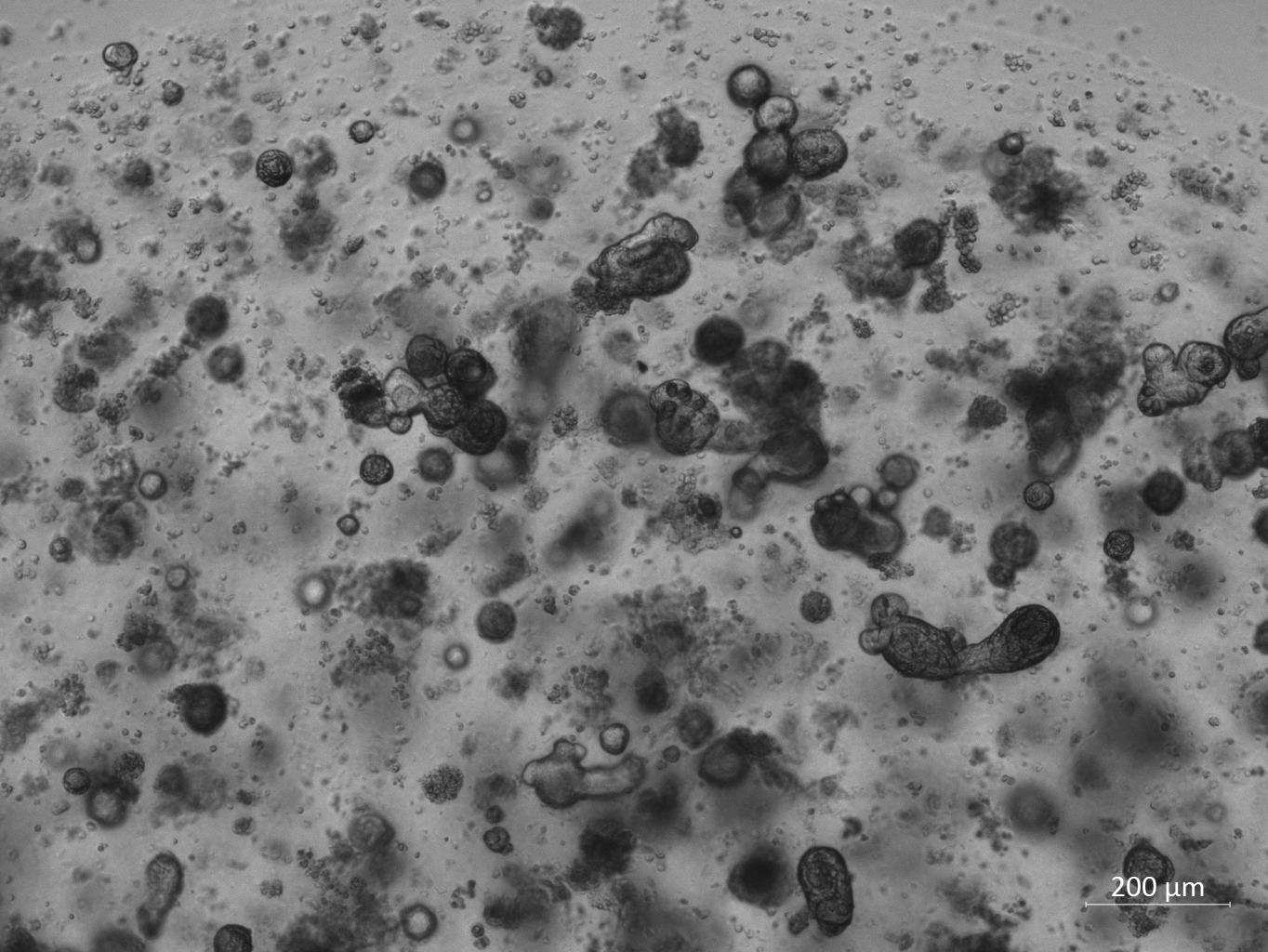

Supplement: Supplementary file 9 — Source data Fig. 6 [file 44318_2025_581_MOESM9_ESM.zip › Fig 6/6E/WT day4 noggin 50.tif]

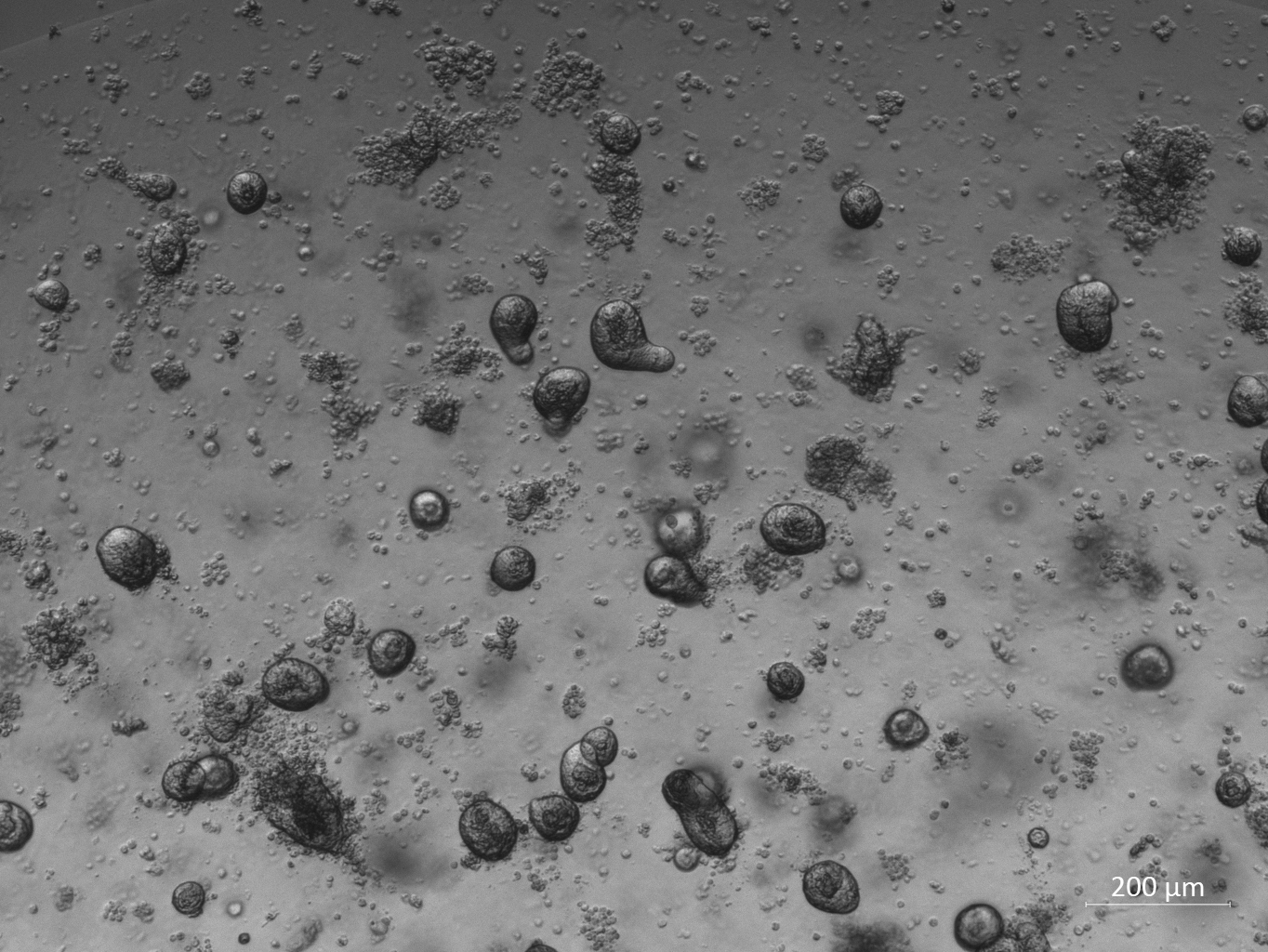

Supplement: Supplementary file 9 — Source data Fig. 6 [file 44318_2025_581_MOESM9_ESM.zip › Fig 6/6E/WT day1 noggin 100.tif]

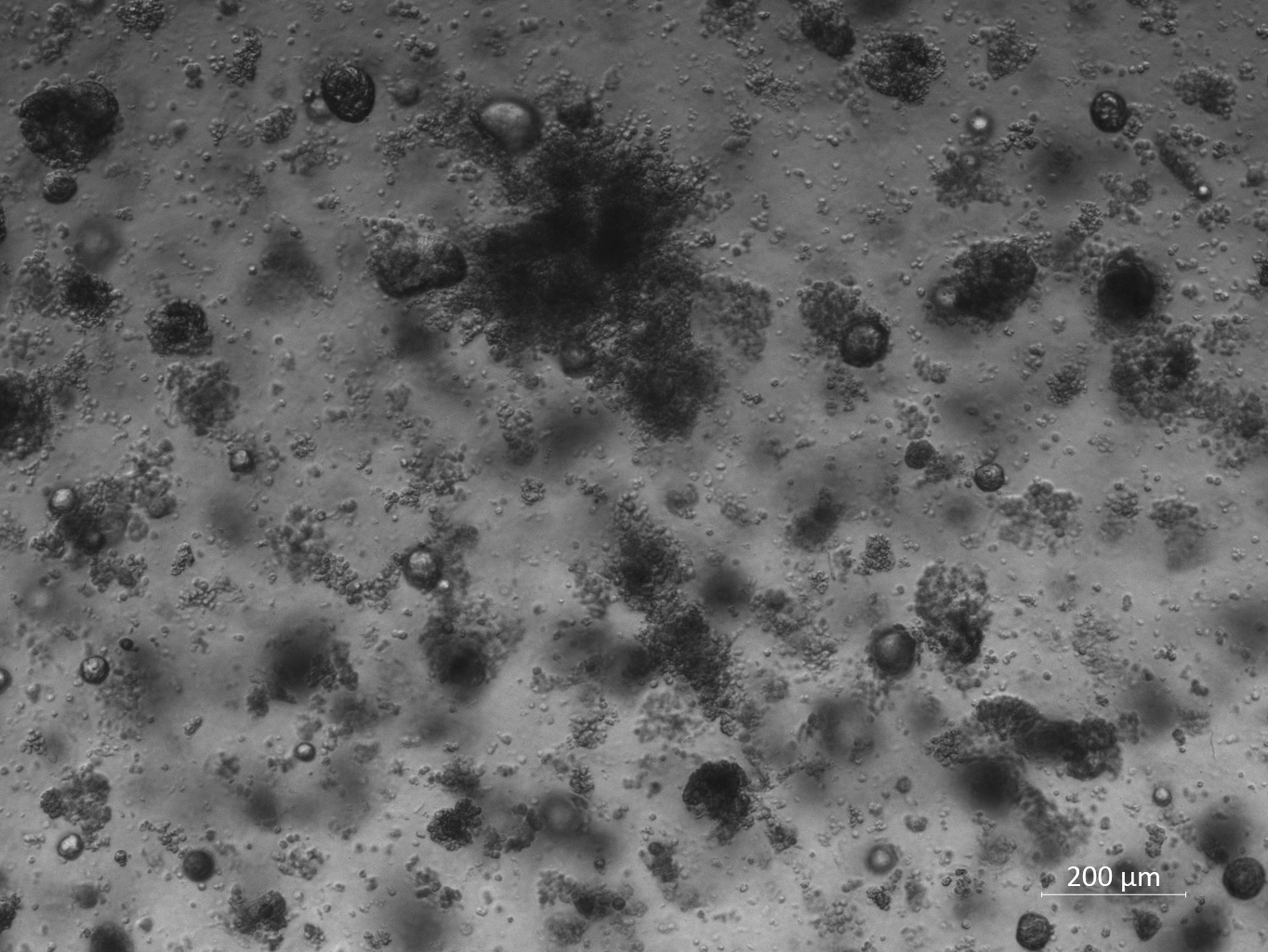

Supplement: Supplementary file 9 — Source data Fig. 6 [file 44318_2025_581_MOESM9_ESM.zip › Fig 6/6E/KO day4 noggin 0.tif]

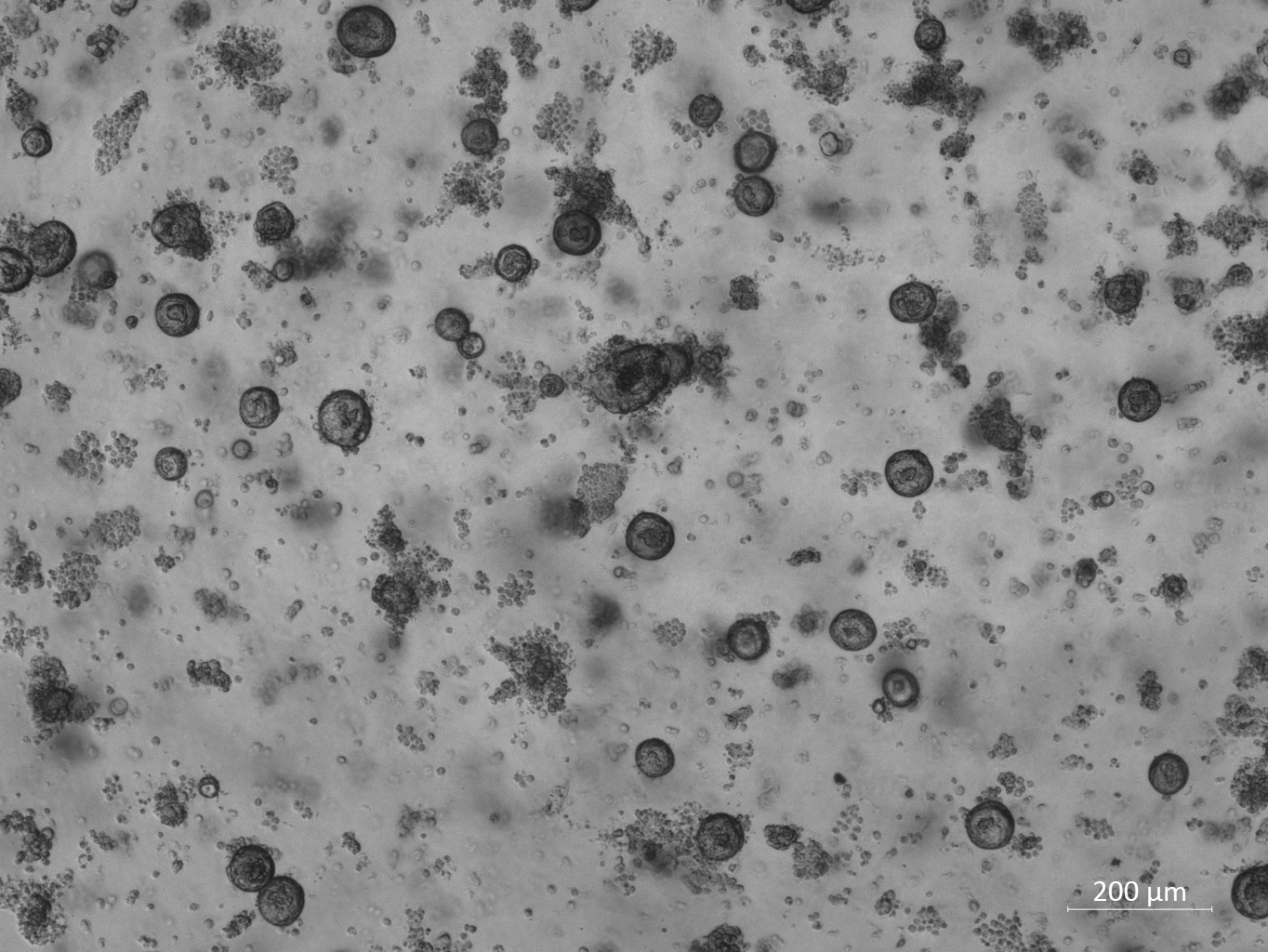

Supplement: Supplementary file 9 — Source data Fig. 6 [file 44318_2025_581_MOESM9_ESM.zip › Fig 6/6E/WT day1 noggin 700.tif]

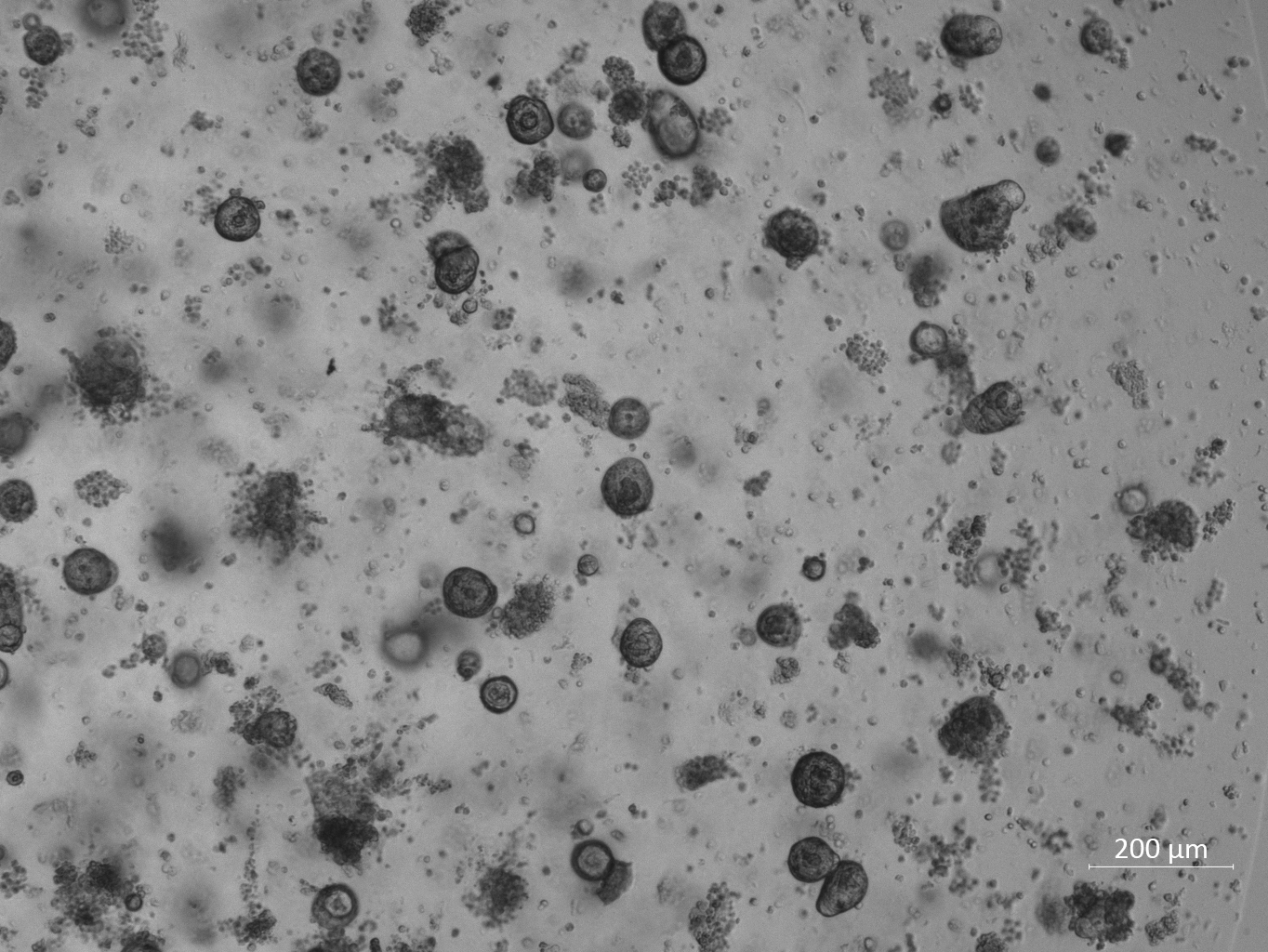

Supplement: Supplementary file 9 — Source data Fig. 6 [file 44318_2025_581_MOESM9_ESM.zip › Fig 6/6E/WT day1 noggin 500.tif]

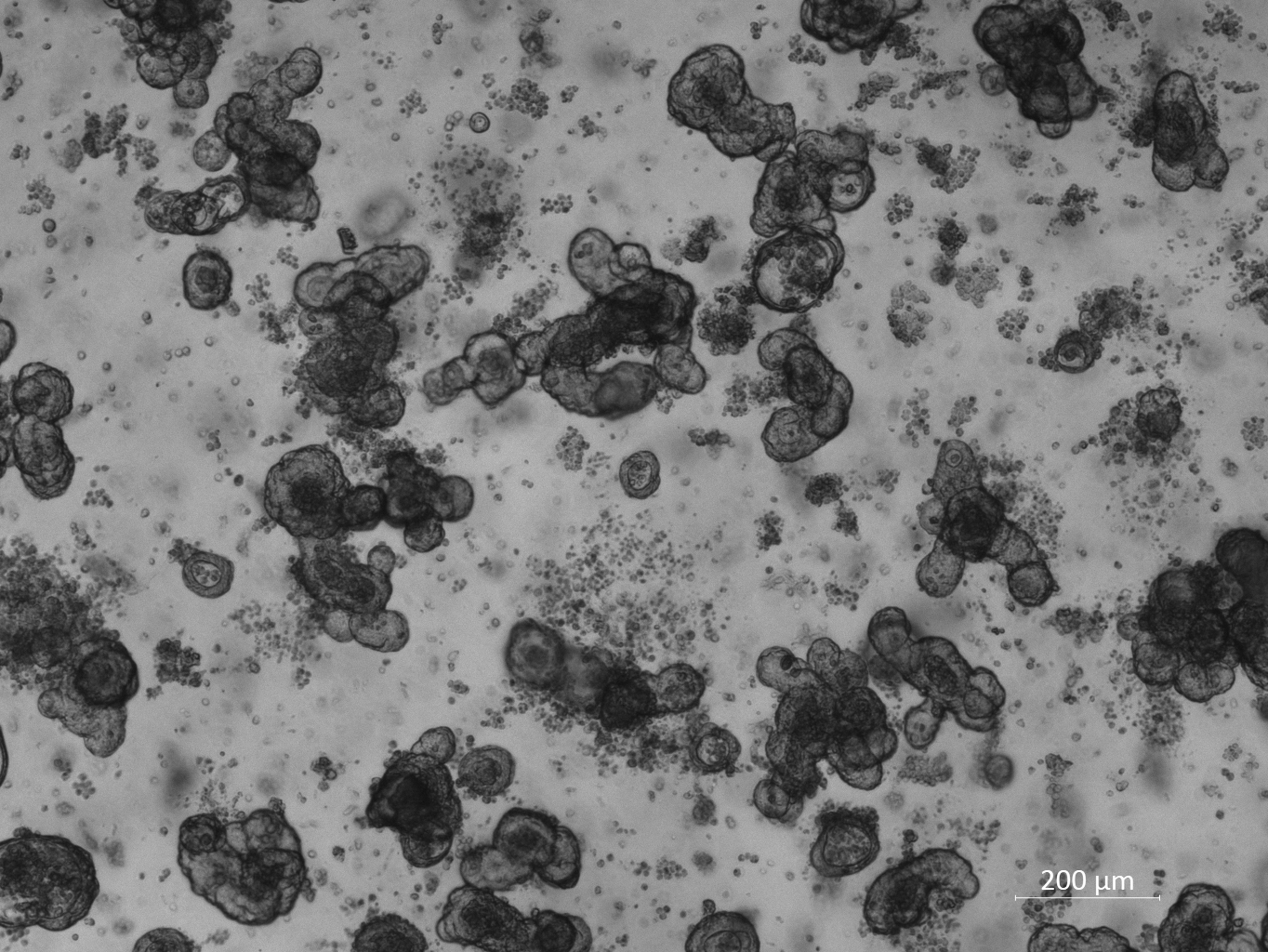

Supplement: Supplementary file 9 — Source data Fig. 6 [file 44318_2025_581_MOESM9_ESM.zip › Fig 6/6E/WT day4 noggin 500.tif]

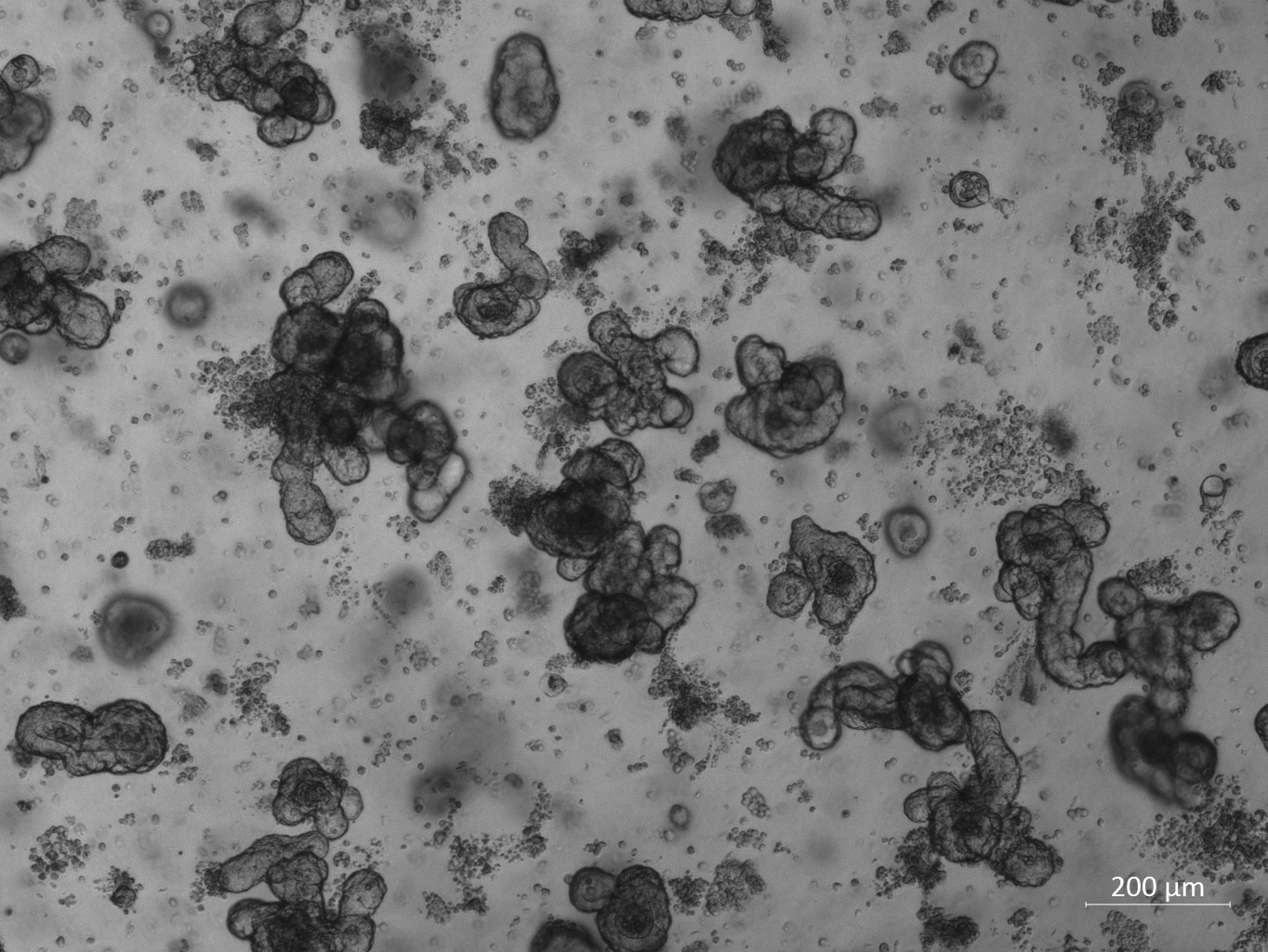

Supplement: Supplementary file 9 — Source data Fig. 6 [file 44318_2025_581_MOESM9_ESM.zip › Fig 6/6E/WT1 day4 100 noggin.tif]

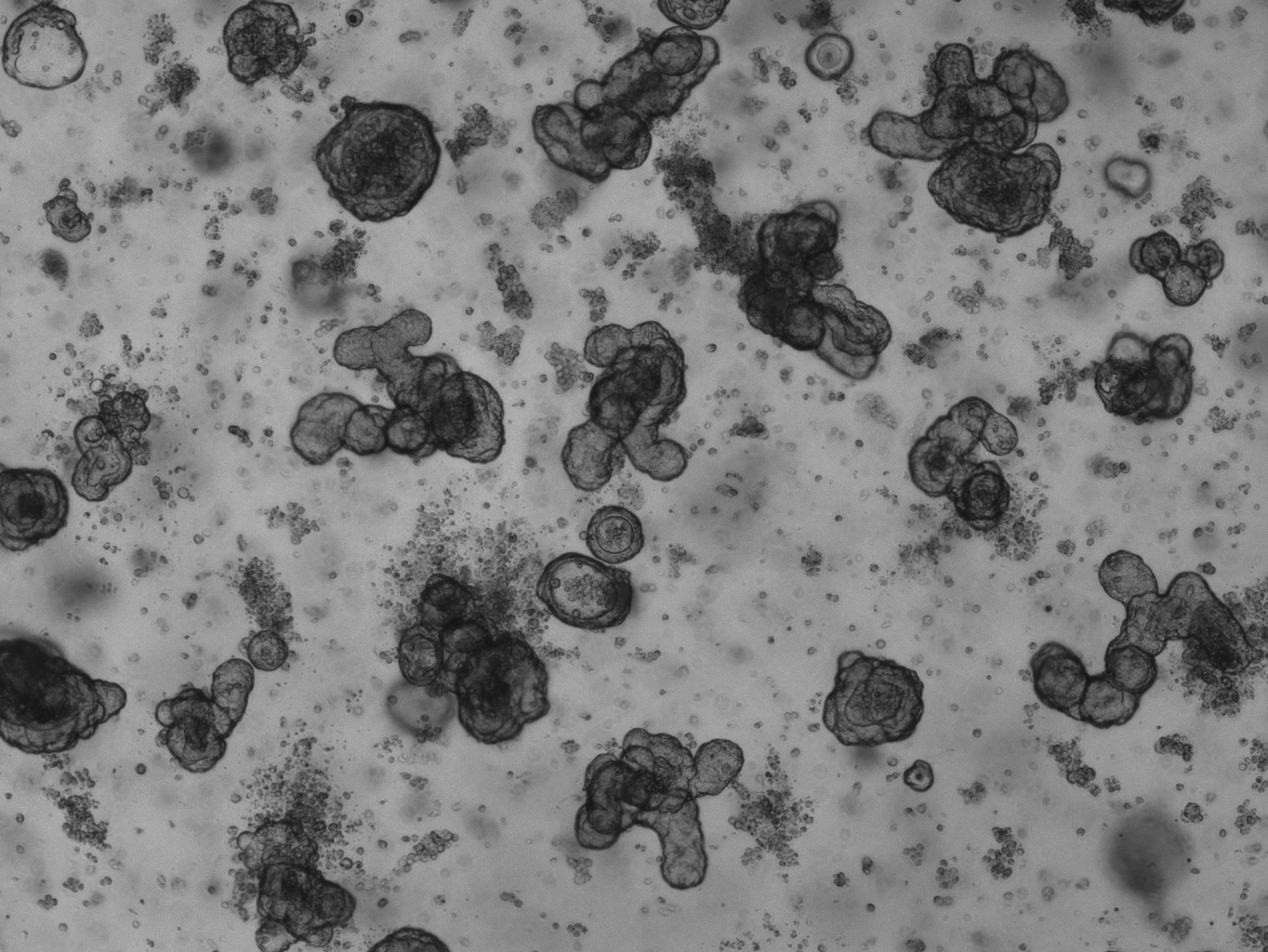

Supplement: Supplementary file 9 — Source data Fig. 6 [file 44318_2025_581_MOESM9_ESM.zip › Fig 6/6E/KO day4 noggin 700.tiff]

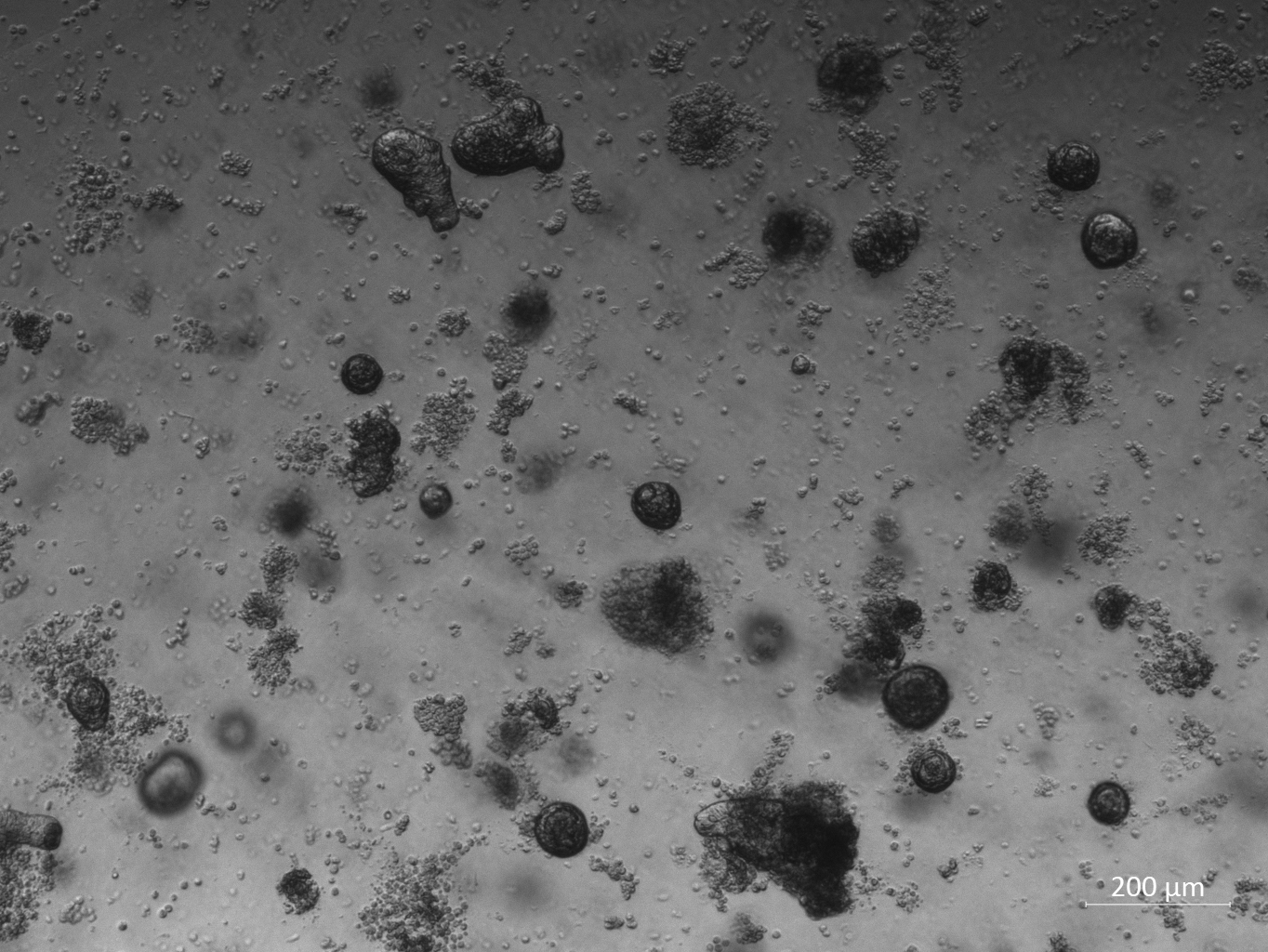

Supplement: Supplementary file 9 — Source data Fig. 6 [file 44318_2025_581_MOESM9_ESM.zip › Fig 6/6E/WT day4 noggin 0.tif]

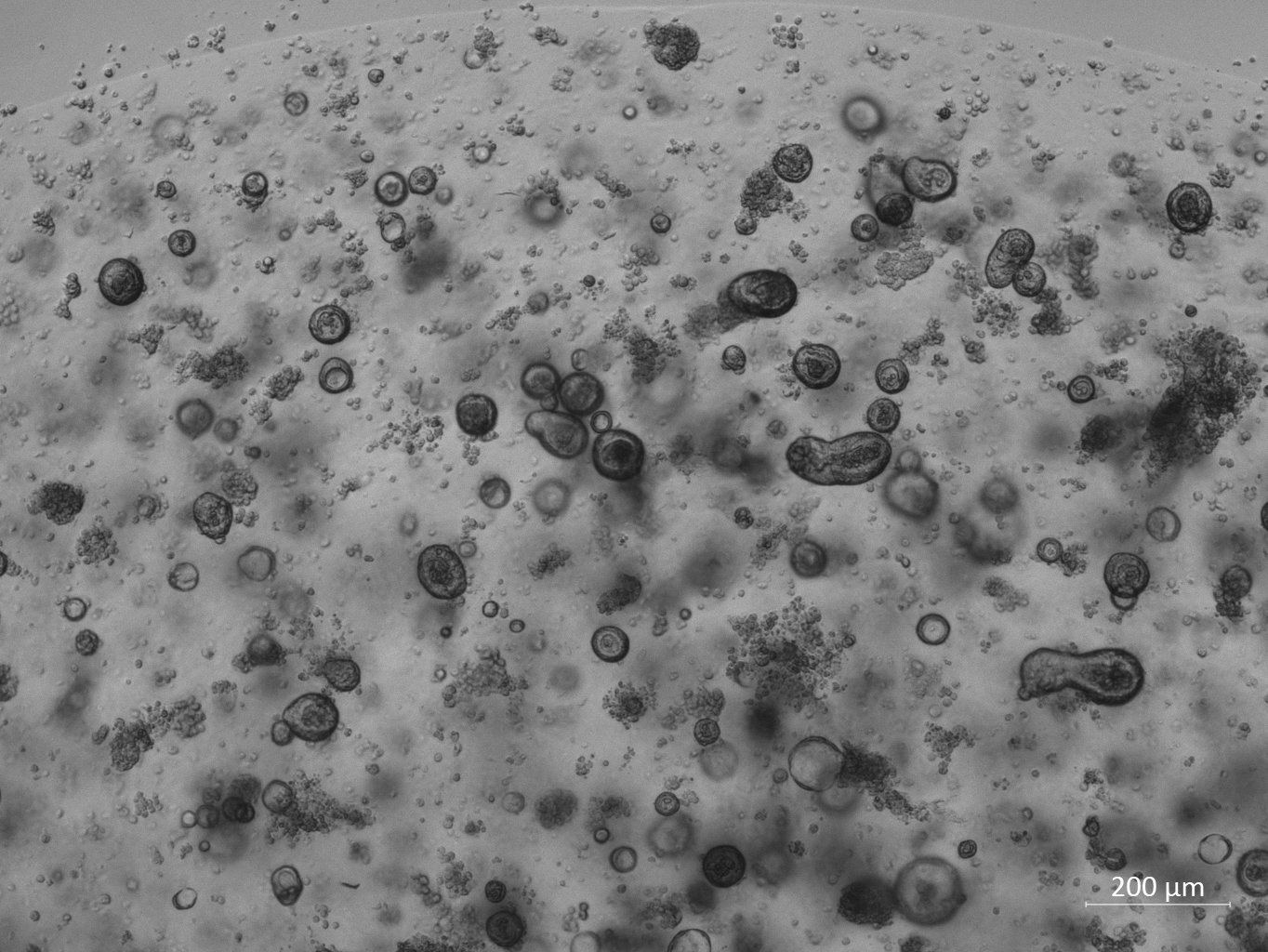

Supplement: Supplementary file 9 — Source data Fig. 6 [file 44318_2025_581_MOESM9_ESM.zip › Fig 6/6E/KO day1 noggin 50.tif]

FAM134C

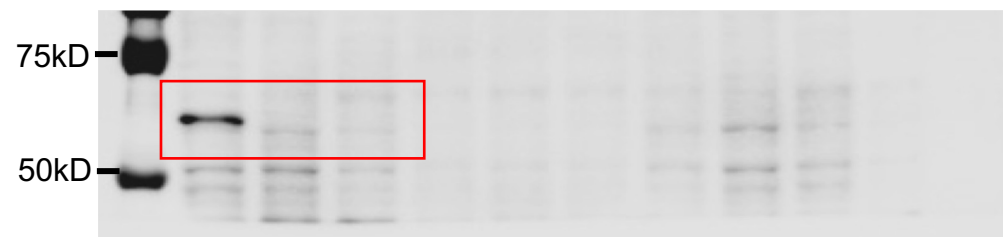

GAPDH

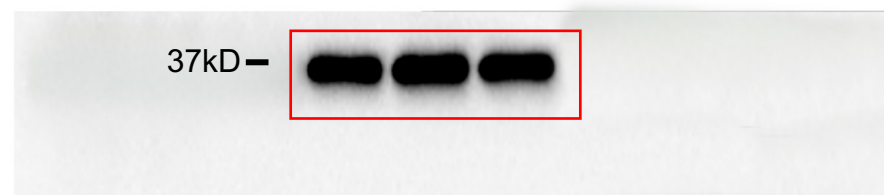

Supplement: Supplementary file 10 — Figure EV Source Data, Movie EV1 and Movie EV2 Source Data [file 44318_2025_581_MOESM10_ESM.zip › Fig EV1/S1C blot.pdf]

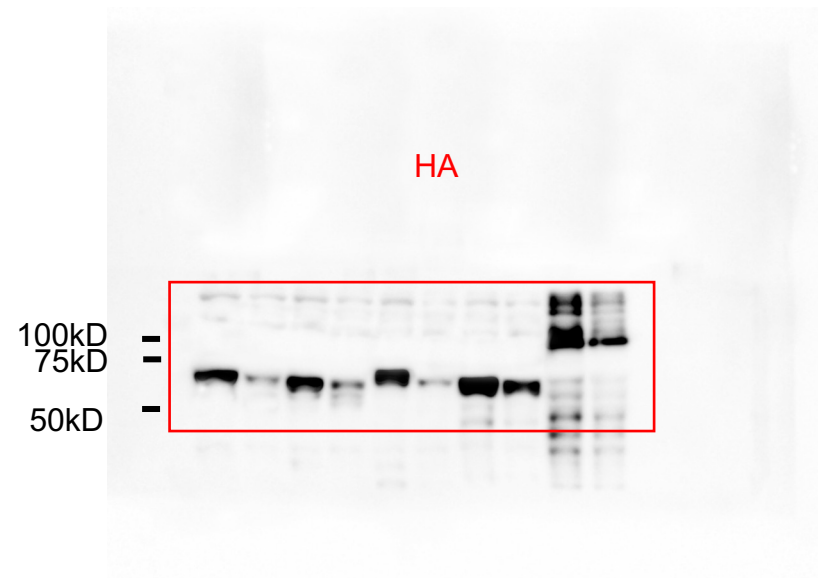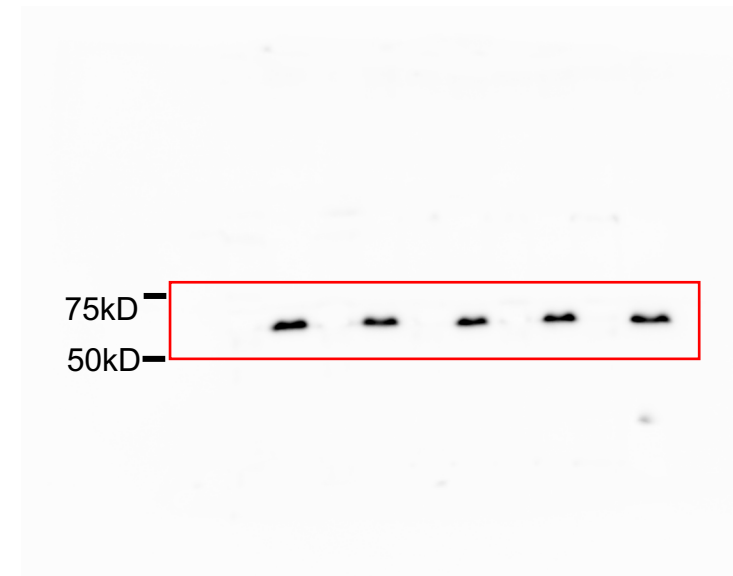

Supplement: Supplementary file 10 — Figure EV Source Data, Movie EV1 and Movie EV2 Source Data [file 44318_2025_581_MOESM10_ESM.zip › Fig EV1/S1E blot.pdf]

BMPR1a

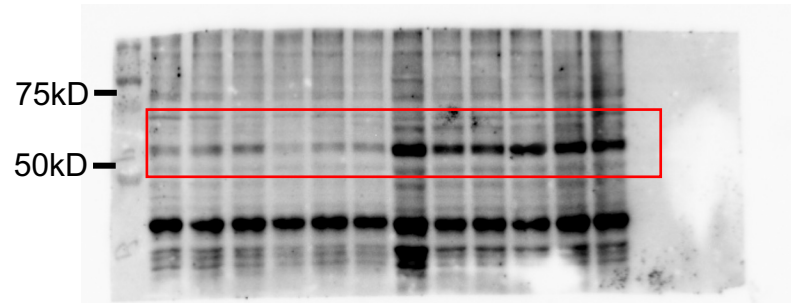

IB: FAM134C

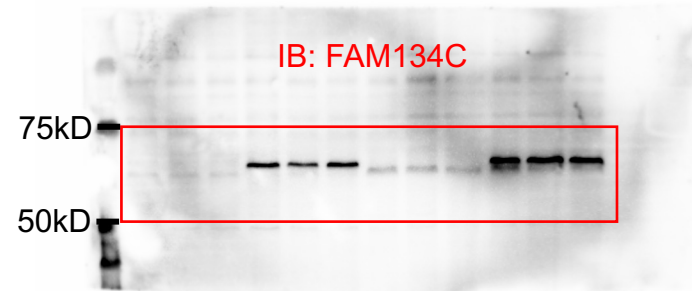

GAPDH

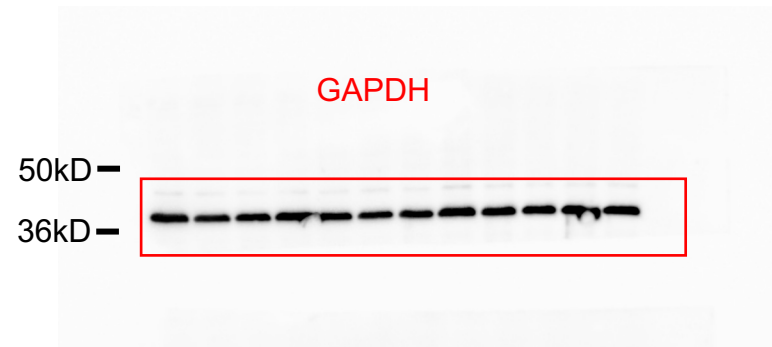

p-S1/5/8

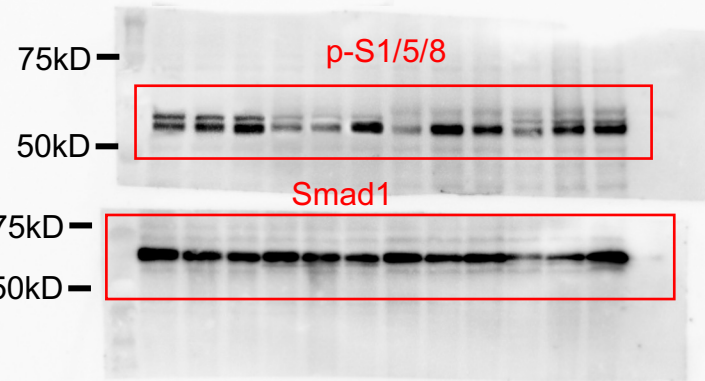

Smad1

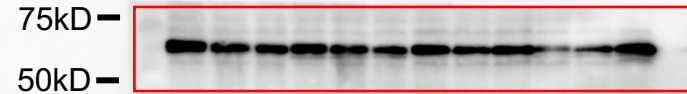

Supplement: Supplementary file 10 — Figure EV Source Data, Movie EV1 and Movie EV2 Source Data [file 44318_2025_581_MOESM10_ESM.zip › Fig EV1/S1H blot.pdf]

# Streptavidin Pulldown

HA

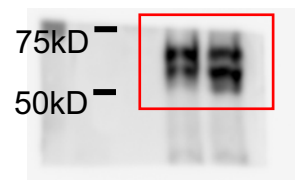

Flag

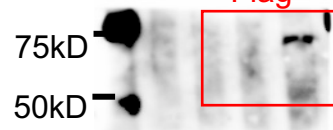

# Whole Cell Lysate

HA

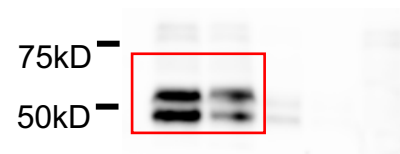

Flag

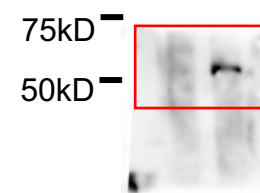

Supplement: Supplementary file 10 — Figure EV Source Data, Movie EV1 and Movie EV2 Source Data [file 44318_2025_581_MOESM10_ESM.zip › Fig EV2/S2 B.pdf]

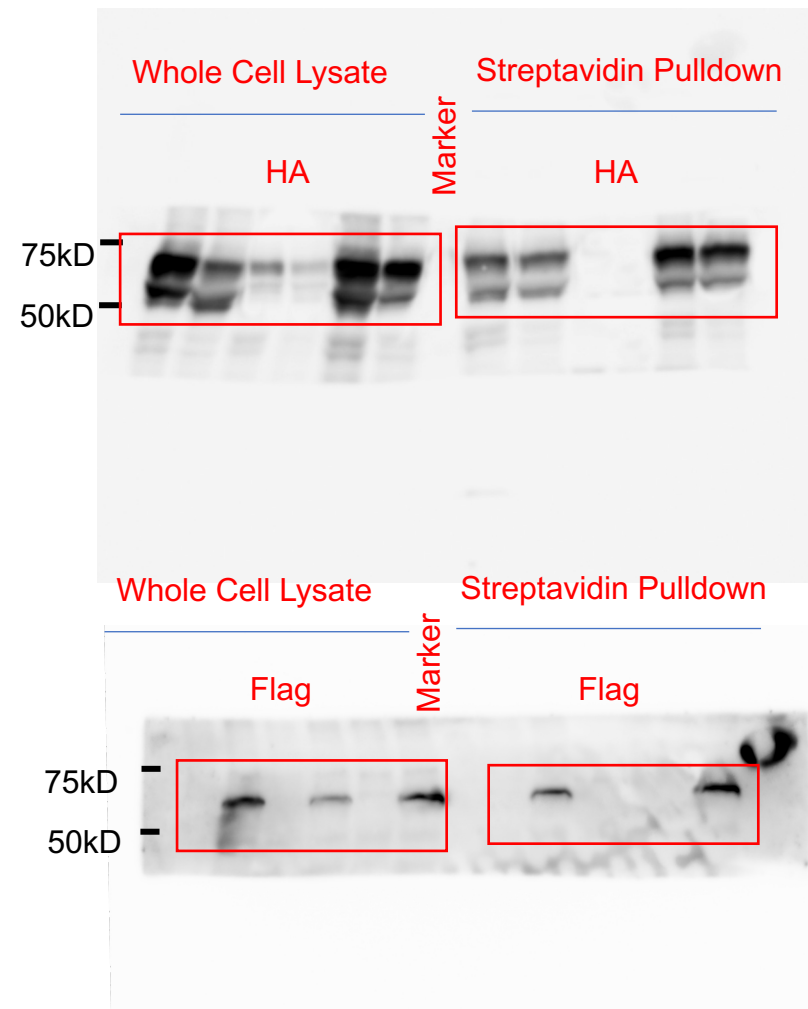

Supplement: Supplementary file 10 — Figure EV Source Data, Movie EV1 and Movie EV2 Source Data [file 44318_2025_581_MOESM10_ESM.zip › Fig EV2/S2 C.pdf]

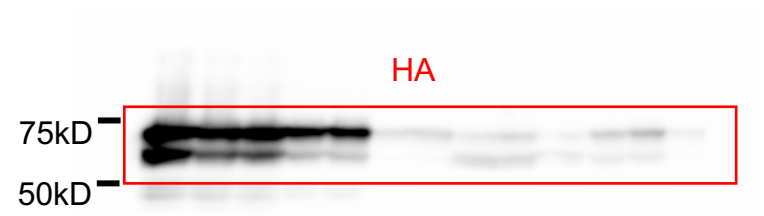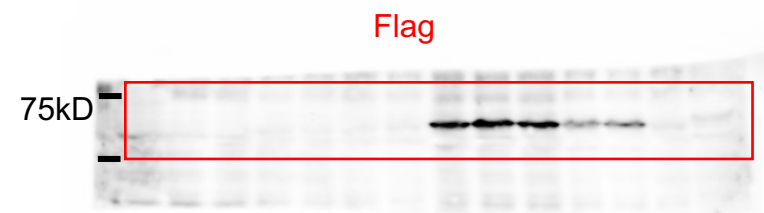

50kD

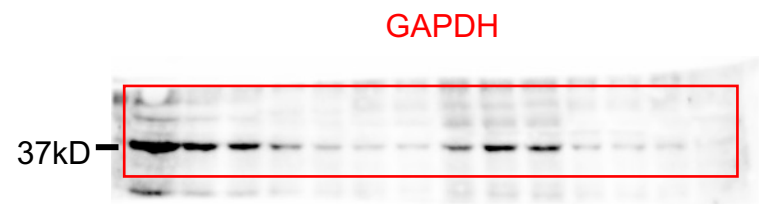

Supplement: Supplementary file 10 — Figure EV Source Data, Movie EV1 and Movie EV2 Source Data [file 44318_2025_581_MOESM10_ESM.zip › Fig EV2/S2 D.pdf]

HA

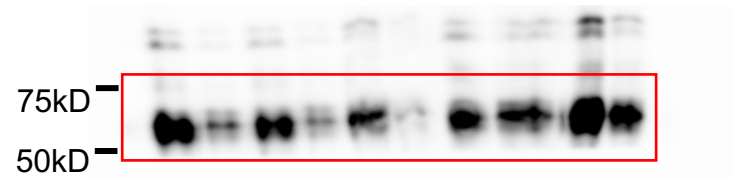

lamp1

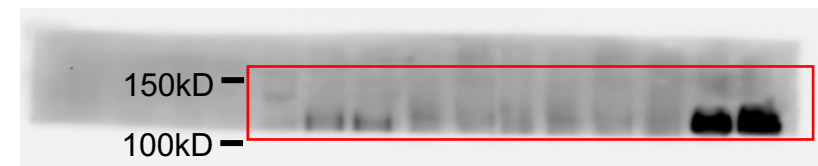

Flag

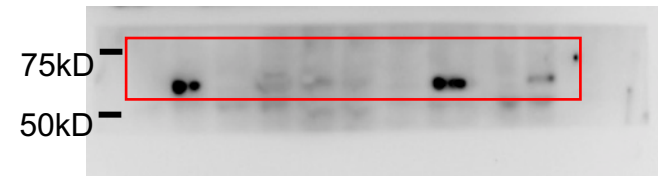

EEA1

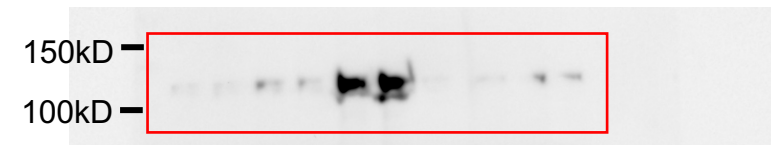

E-cadherin

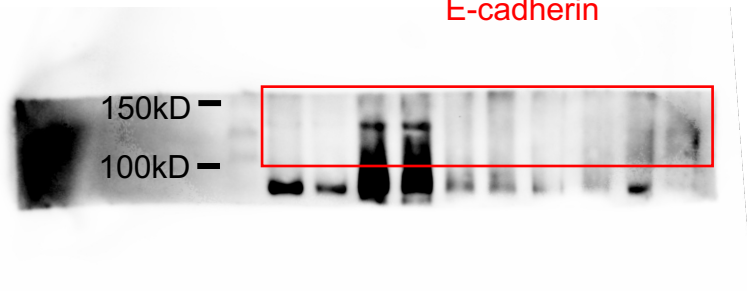

PDAI3

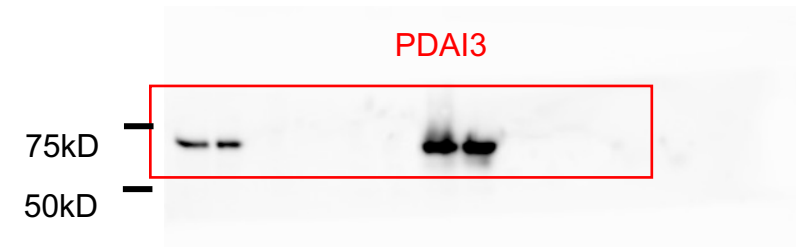

Supplement: Supplementary file 10 — Figure EV Source Data, Movie EV1 and Movie EV2 Source Data [file 44318_2025_581_MOESM10_ESM.zip › Fig EV2/S2 E.pdf]

HA

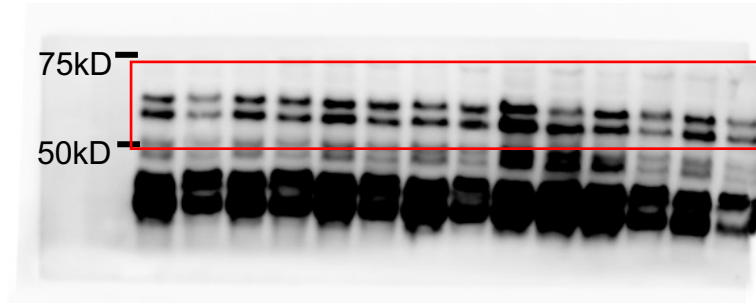

Flag

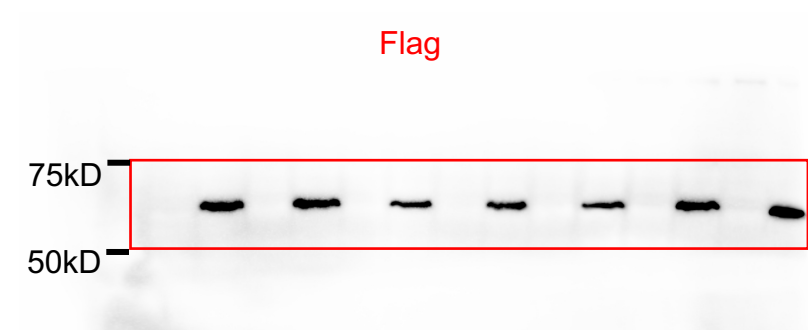

Supplement: Supplementary file 10 — Figure EV Source Data, Movie EV1 and Movie EV2 Source Data [file 44318_2025_581_MOESM10_ESM.zip › Fig EV2/S2 F.pdf]

WCL  
IB: FAM134C

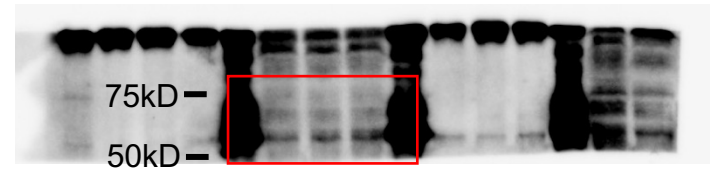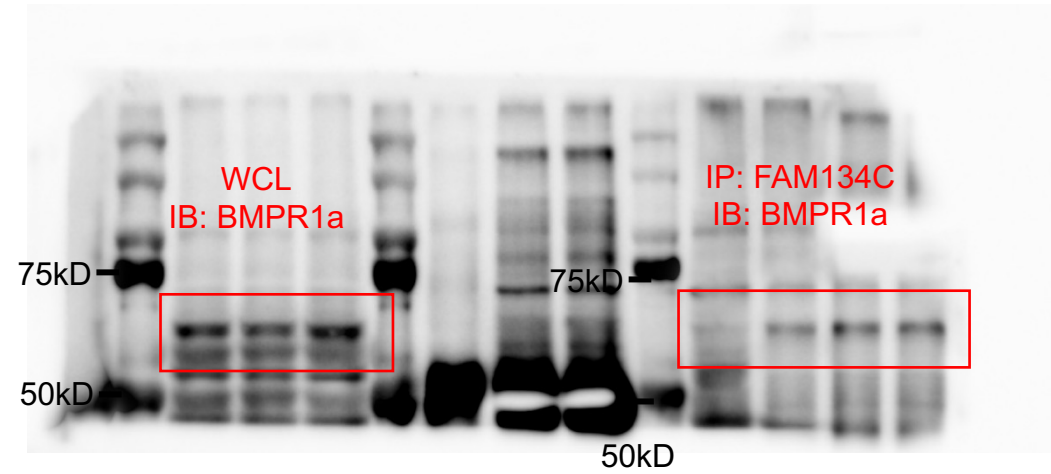

IP: FAM134C  
IB: FAM134C

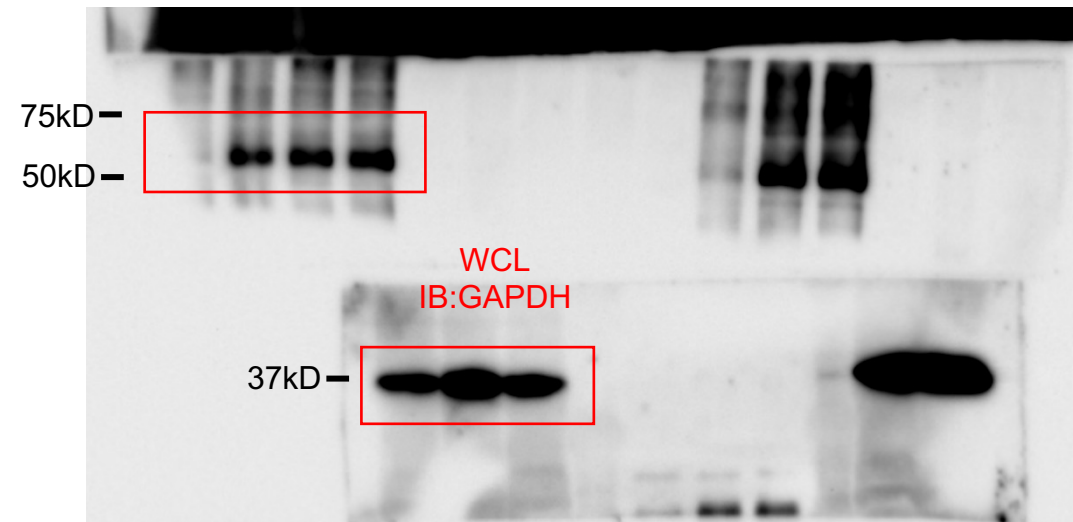

Supplement: Supplementary file 10 — Figure EV Source Data, Movie EV1 and Movie EV2 Source Data [file 44318_2025_581_MOESM10_ESM.zip › Fig EV3/S3 A Blot.pdf]

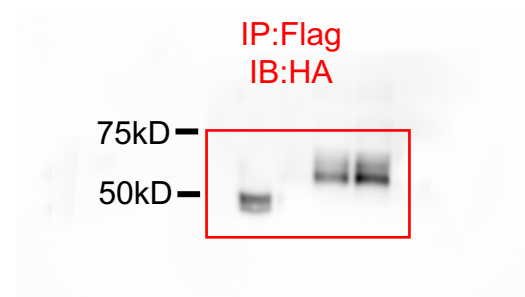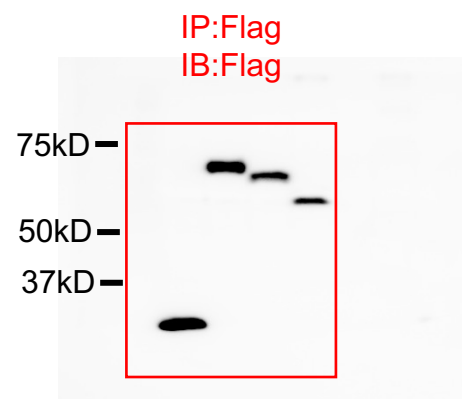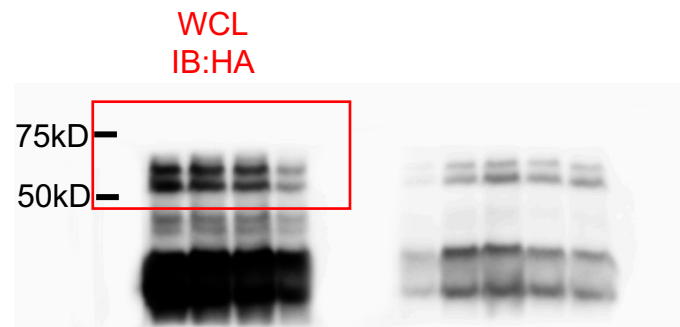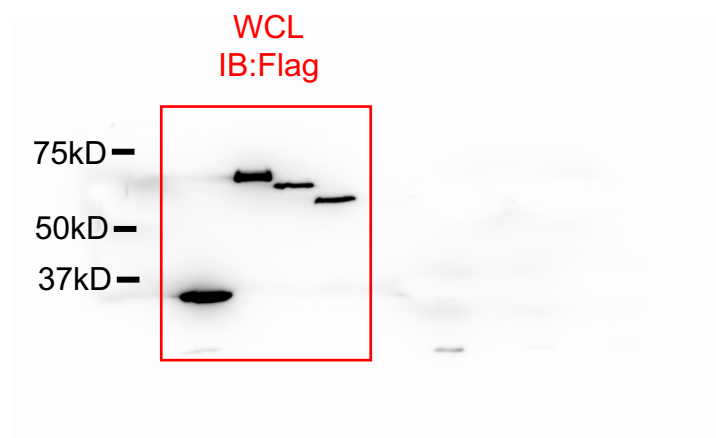

Supplement: Supplementary file 10 — Figure EV Source Data, Movie EV1 and Movie EV2 Source Data [file 44318_2025_581_MOESM10_ESM.zip › Fig EV3/S3 B Blot.pdf]

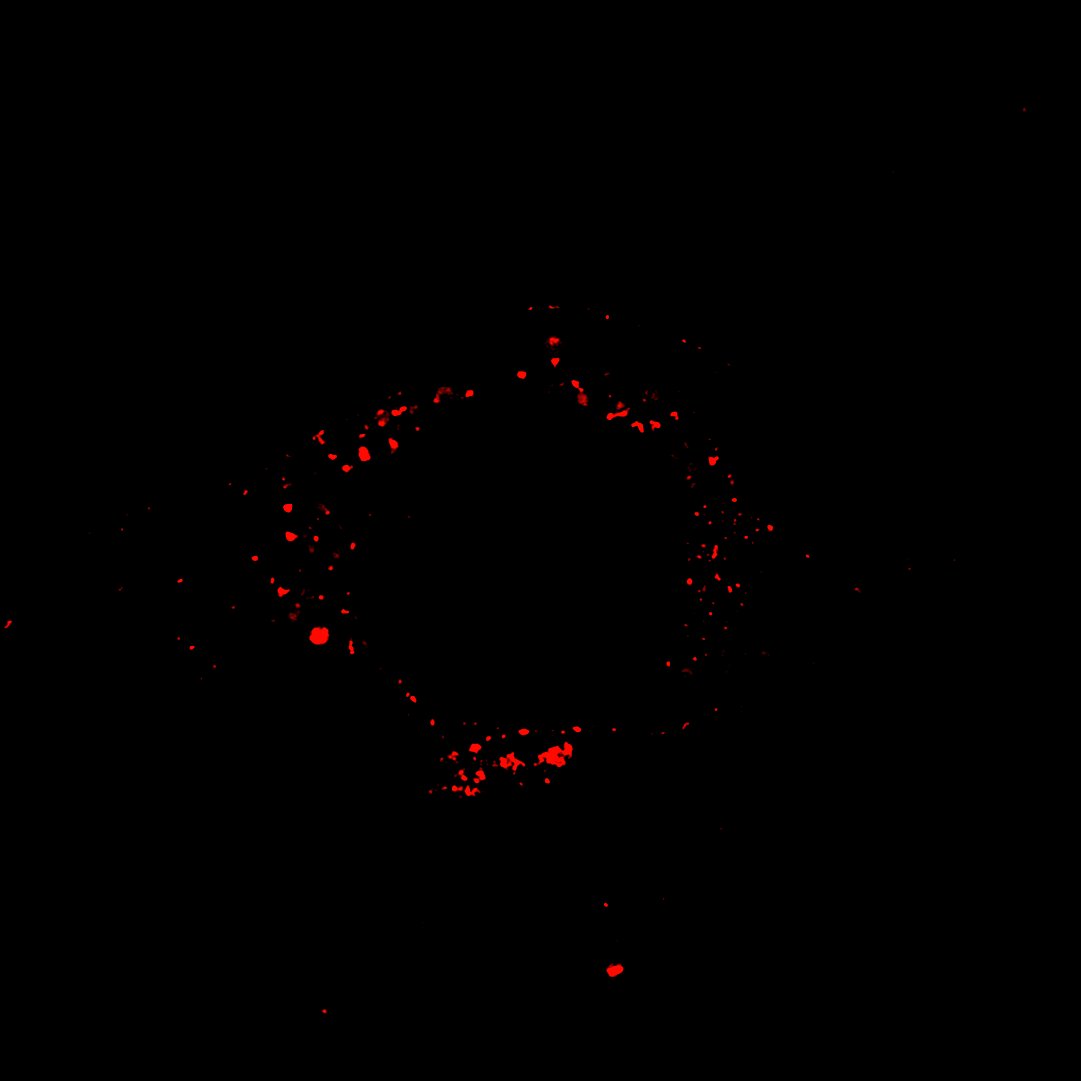

Supplement: Supplementary file 10 — Figure EV Source Data, Movie EV1 and Movie EV2 Source Data [file 44318_2025_581_MOESM10_ESM.zip › Fig EV3/S3 C/U2OS clatherin-mcherry 134c-yn ALK3-yc-mcherry.jpg]

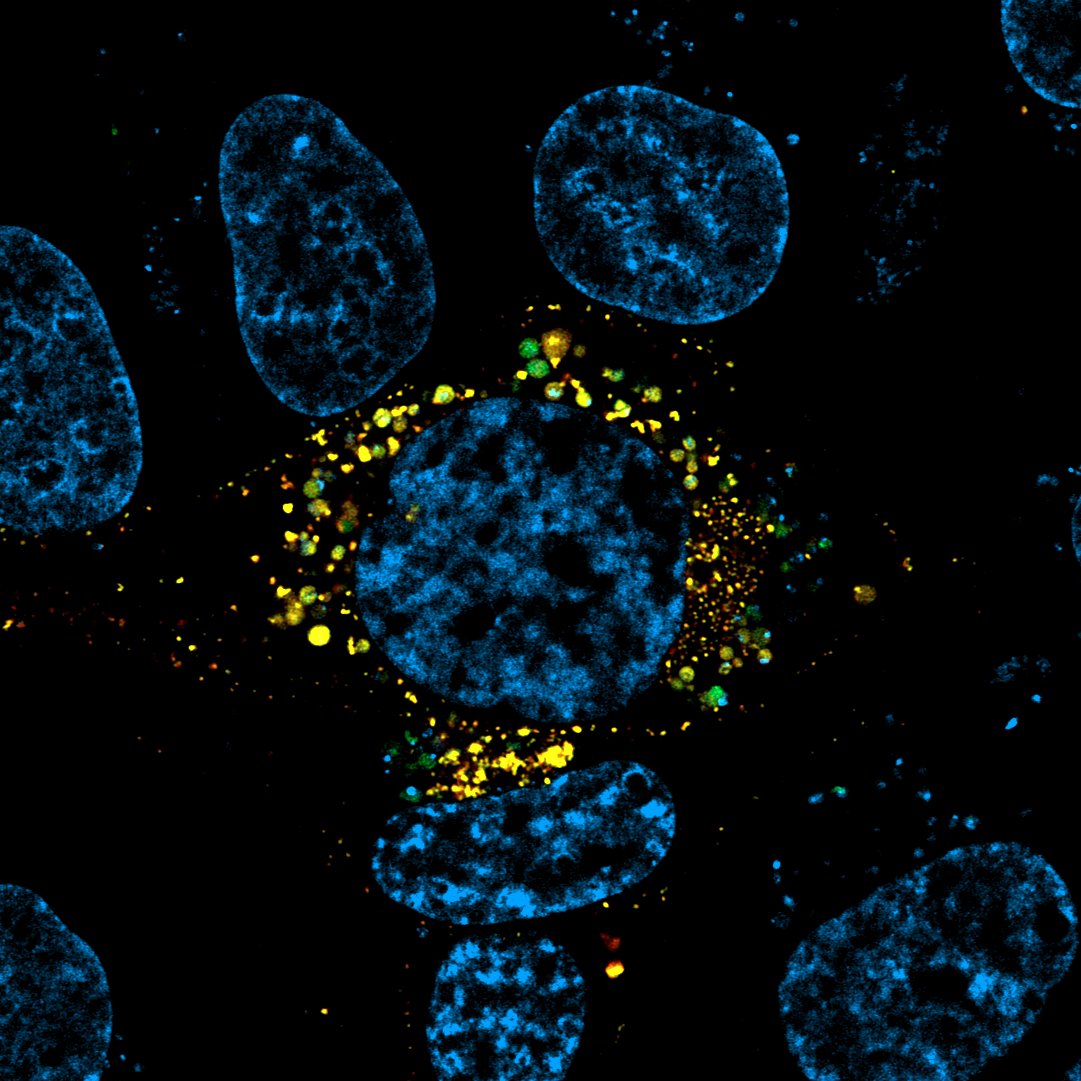

Supplement: Supplementary file 10 — Figure EV Source Data, Movie EV1 and Movie EV2 Source Data [file 44318_2025_581_MOESM10_ESM.zip › Fig EV3/S3 C/U2OS clatherin-mcherry 134c-yn ALK3-yc-merged.jpg]

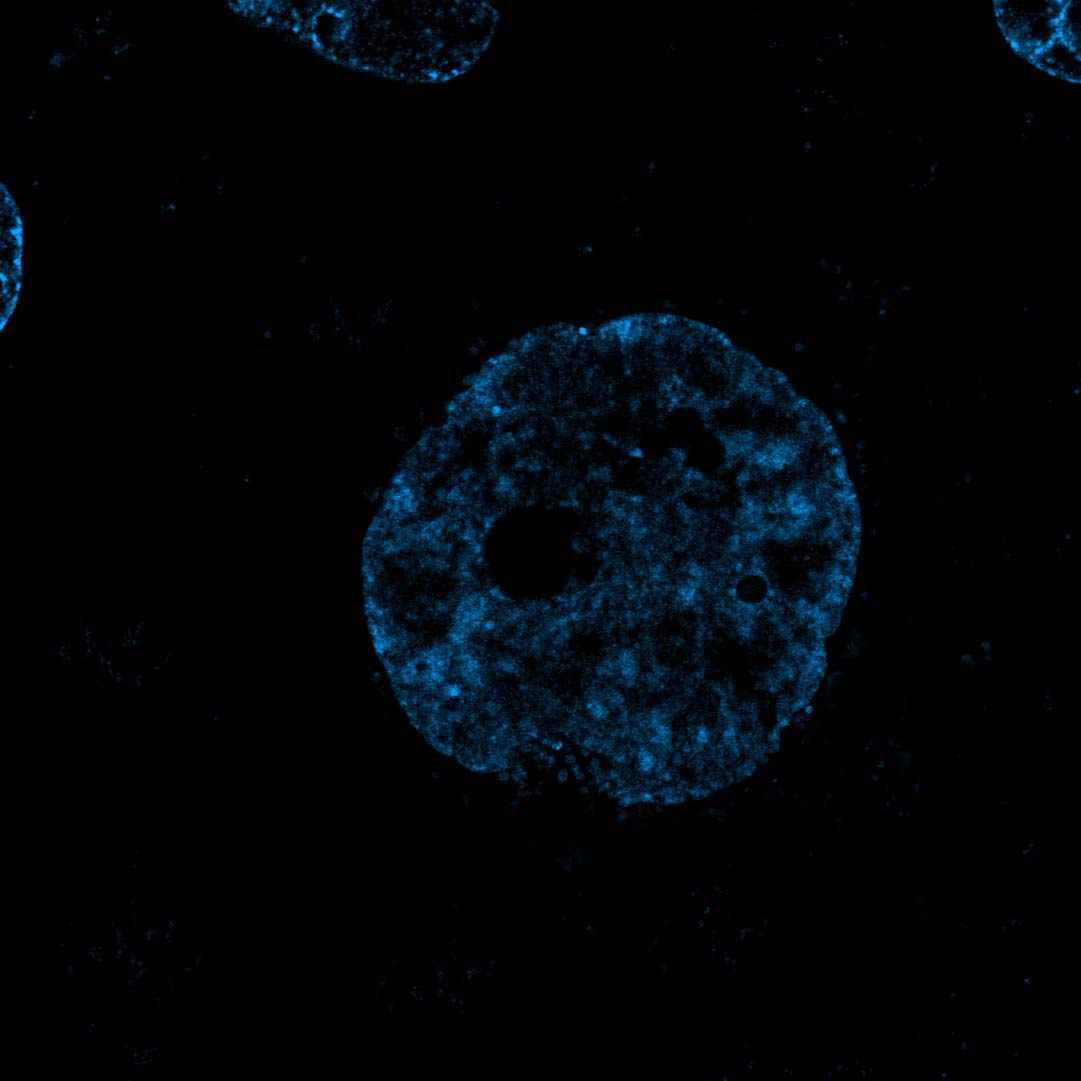

Supplement: Supplementary file 10 — Figure EV Source Data, Movie EV1 and Movie EV2 Source Data [file 44318_2025_581_MOESM10_ESM.zip › Fig EV3/S3 C/U2OS clatherin-mcherry 134c-yn ALK3-yc-nuclear.jpg]

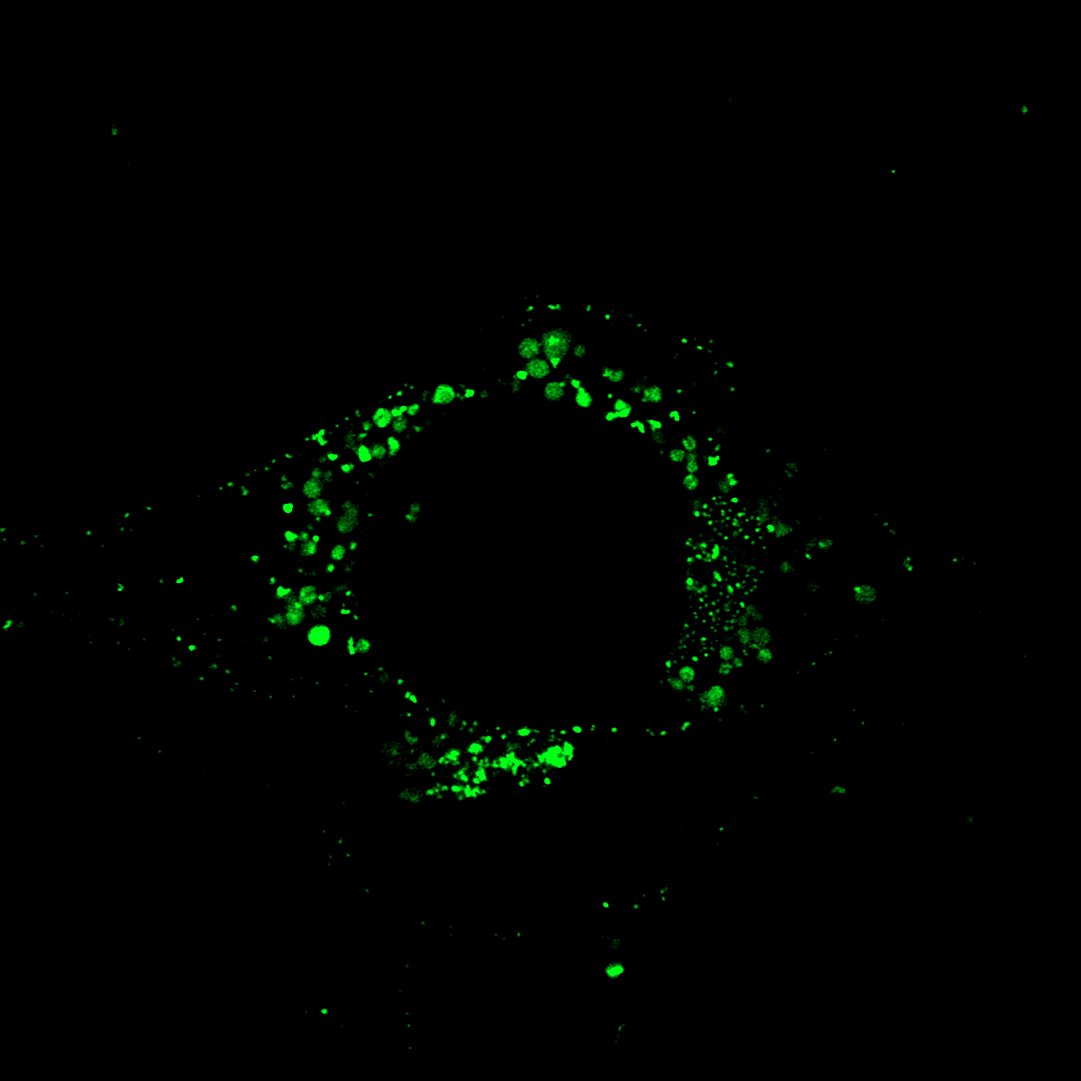

Supplement: Supplementary file 10 — Figure EV Source Data, Movie EV1 and Movie EV2 Source Data [file 44318_2025_581_MOESM10_ESM.zip › Fig EV3/S3 C/U2OS clatherin-mcherry 134c-yn ALK3-yc.jpg]

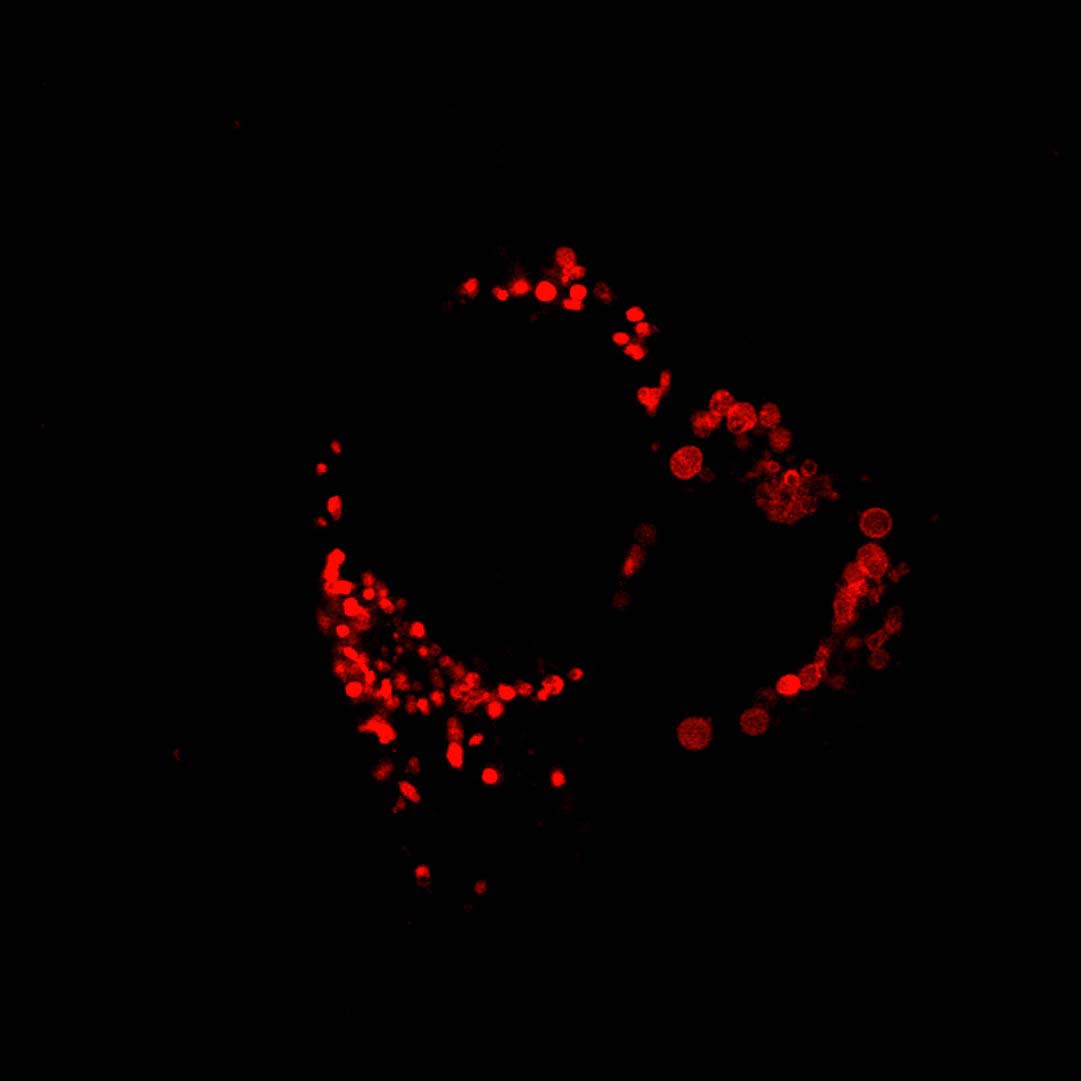

Supplement: Supplementary file 10 — Figure EV Source Data, Movie EV1 and Movie EV2 Source Data [file 44318_2025_581_MOESM10_ESM.zip › Fig EV3/S3 C/U2OS lamp1-mcherry 134c-yn ALK3-yc-mcherry.jpg]

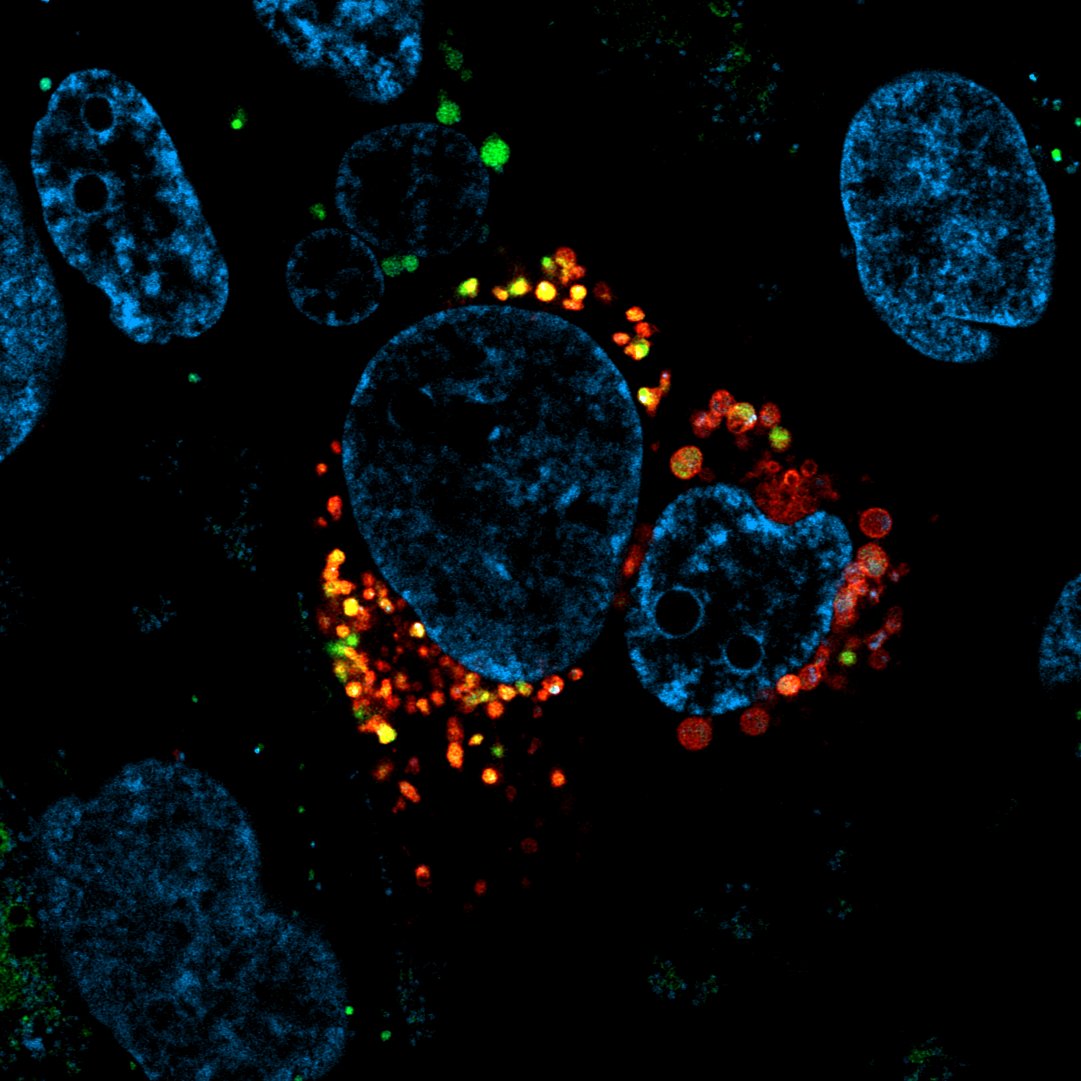

Supplement: Supplementary file 10 — Figure EV Source Data, Movie EV1 and Movie EV2 Source Data [file 44318_2025_581_MOESM10_ESM.zip › Fig EV3/S3 C/U2OS lamp1-mcherry 134c-yn ALK3-yc-merged.jpg]

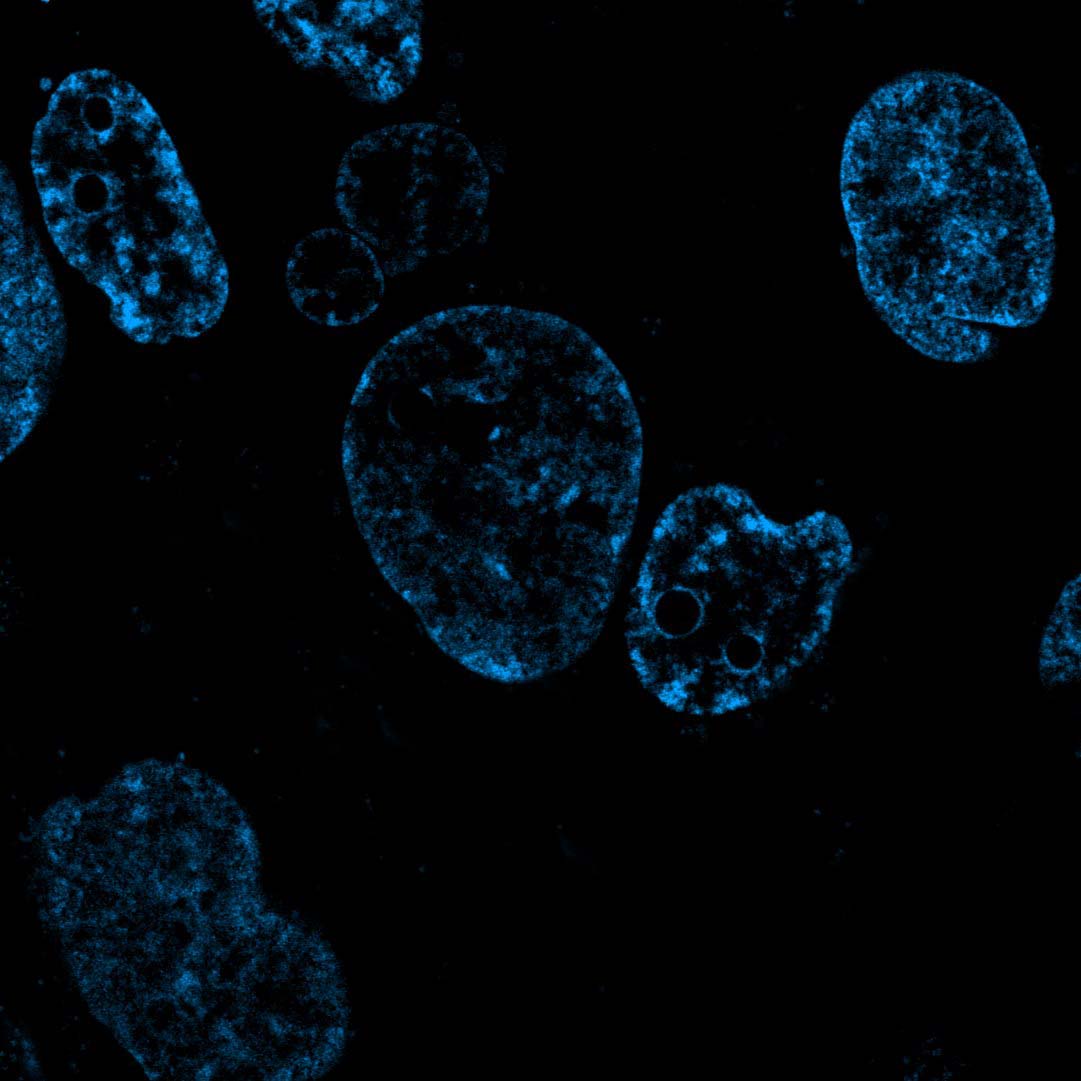

Supplement: Supplementary file 10 — Figure EV Source Data, Movie EV1 and Movie EV2 Source Data [file 44318_2025_581_MOESM10_ESM.zip › Fig EV3/S3 C/U2OS lamp1-mcherry 134c-yn ALK3-yc-Nuclear.jpg]

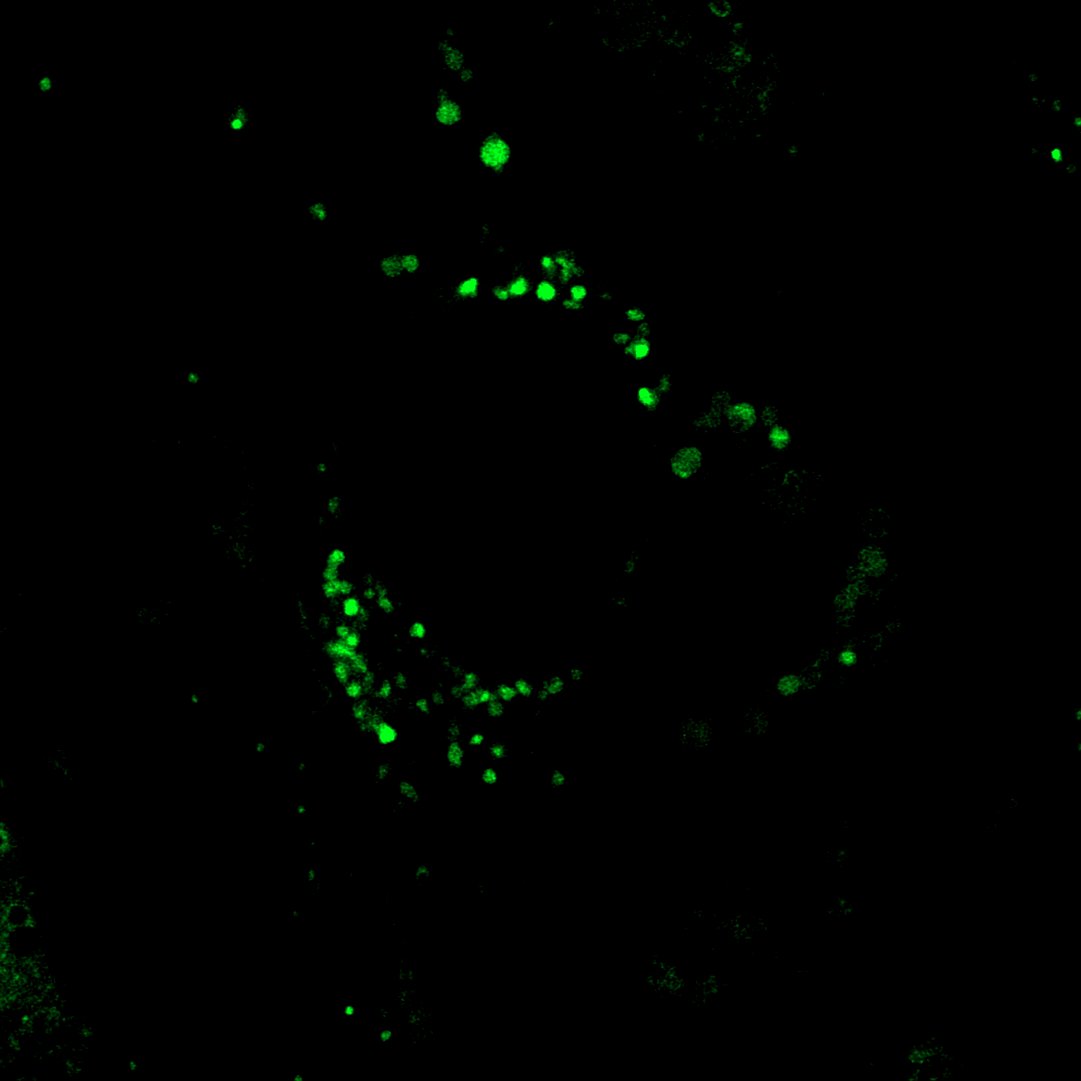

Supplement: Supplementary file 10 — Figure EV Source Data, Movie EV1 and Movie EV2 Source Data [file 44318_2025_581_MOESM10_ESM.zip › Fig EV3/S3 C/U2OS lamp1-mcherry 134c-yn ALK3-yc.jpg]

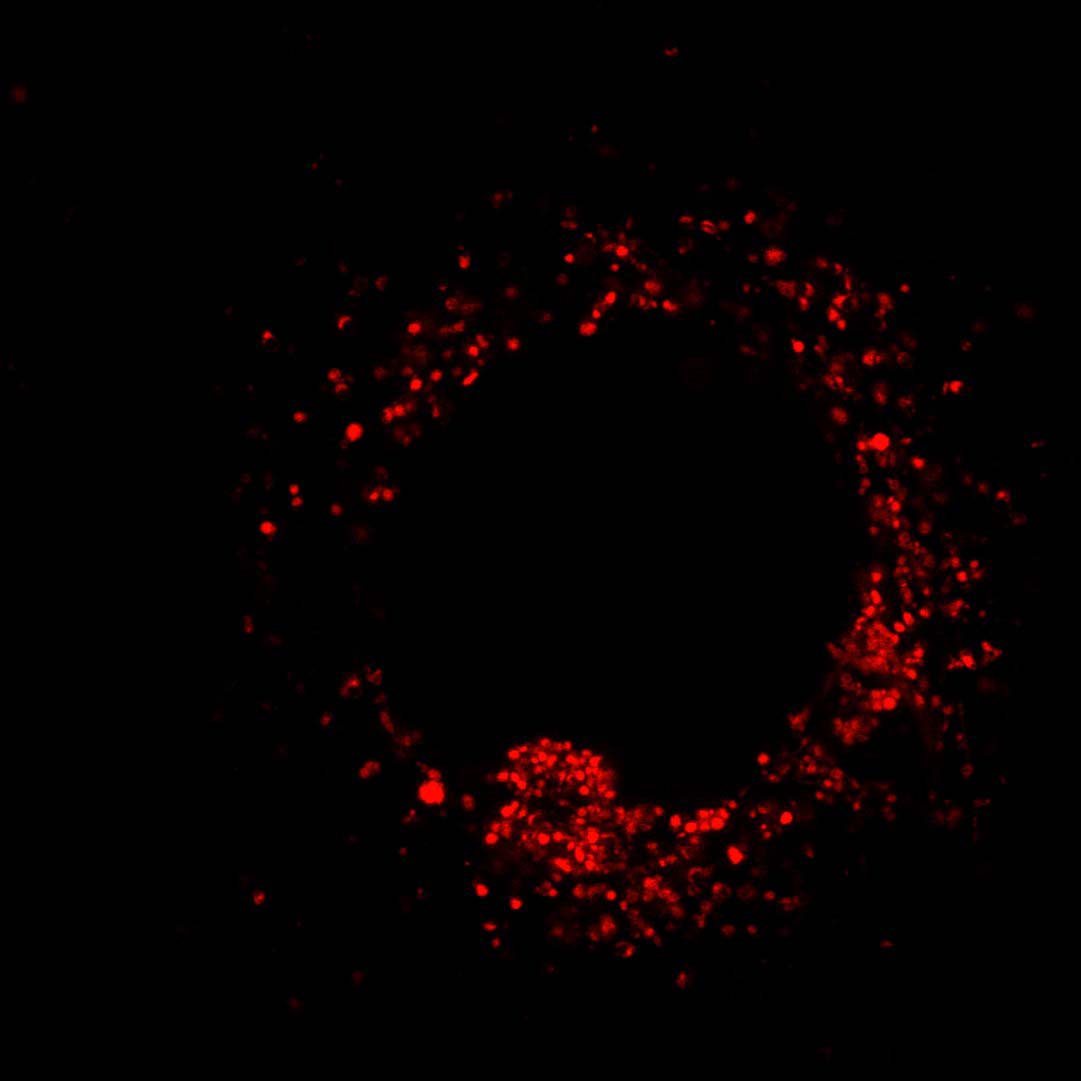

Supplement: Supplementary file 10 — Figure EV Source Data, Movie EV1 and Movie EV2 Source Data [file 44318_2025_581_MOESM10_ESM.zip › Fig EV3/S3 C/U2OS rab7-mcherry 134c-yn ALK3-yc-mcherry.jpg]

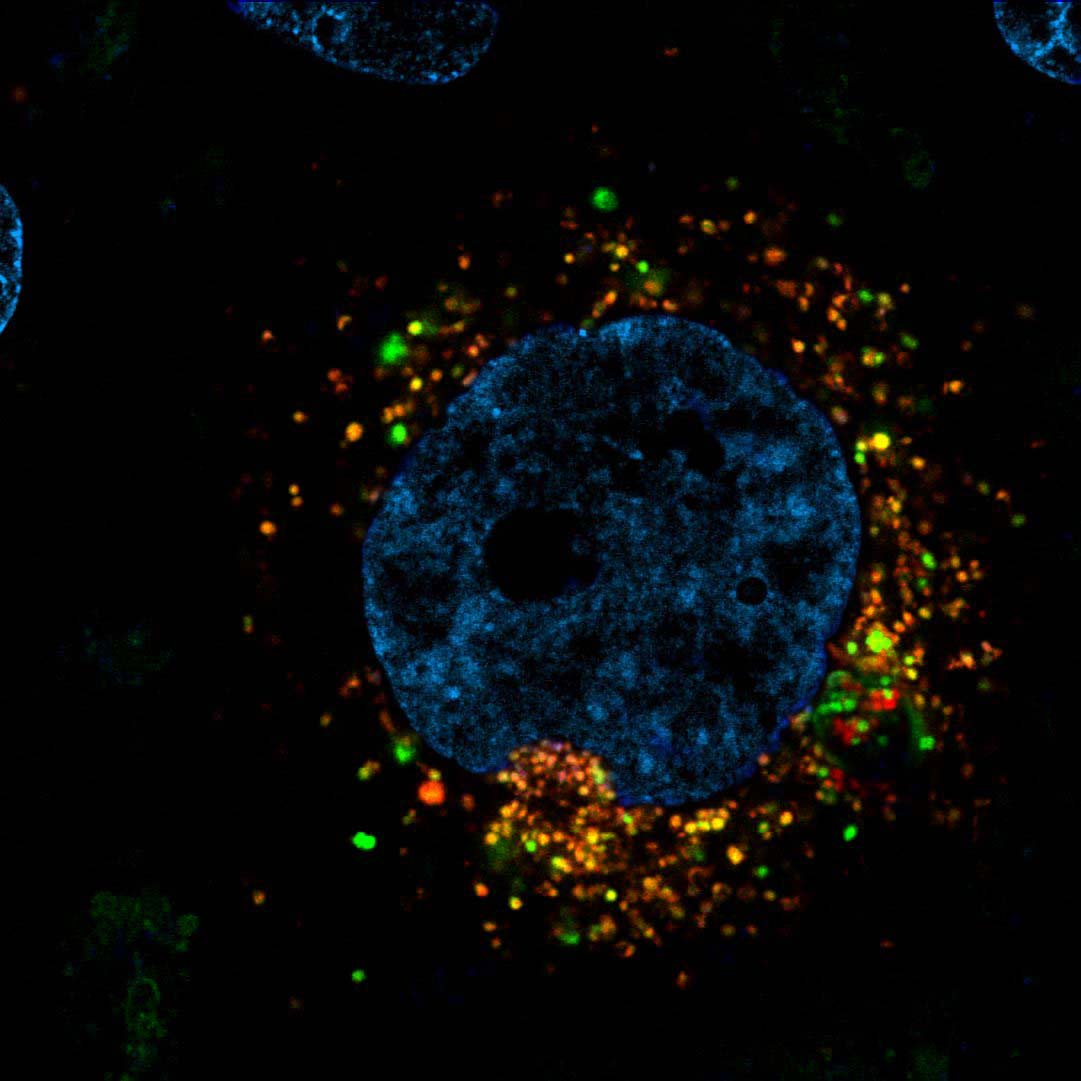

Supplement: Supplementary file 10 — Figure EV Source Data, Movie EV1 and Movie EV2 Source Data [file 44318_2025_581_MOESM10_ESM.zip › Fig EV3/S3 C/U2OS rab7-mcherry 134c-yn ALK3-yc-merged.jpg]

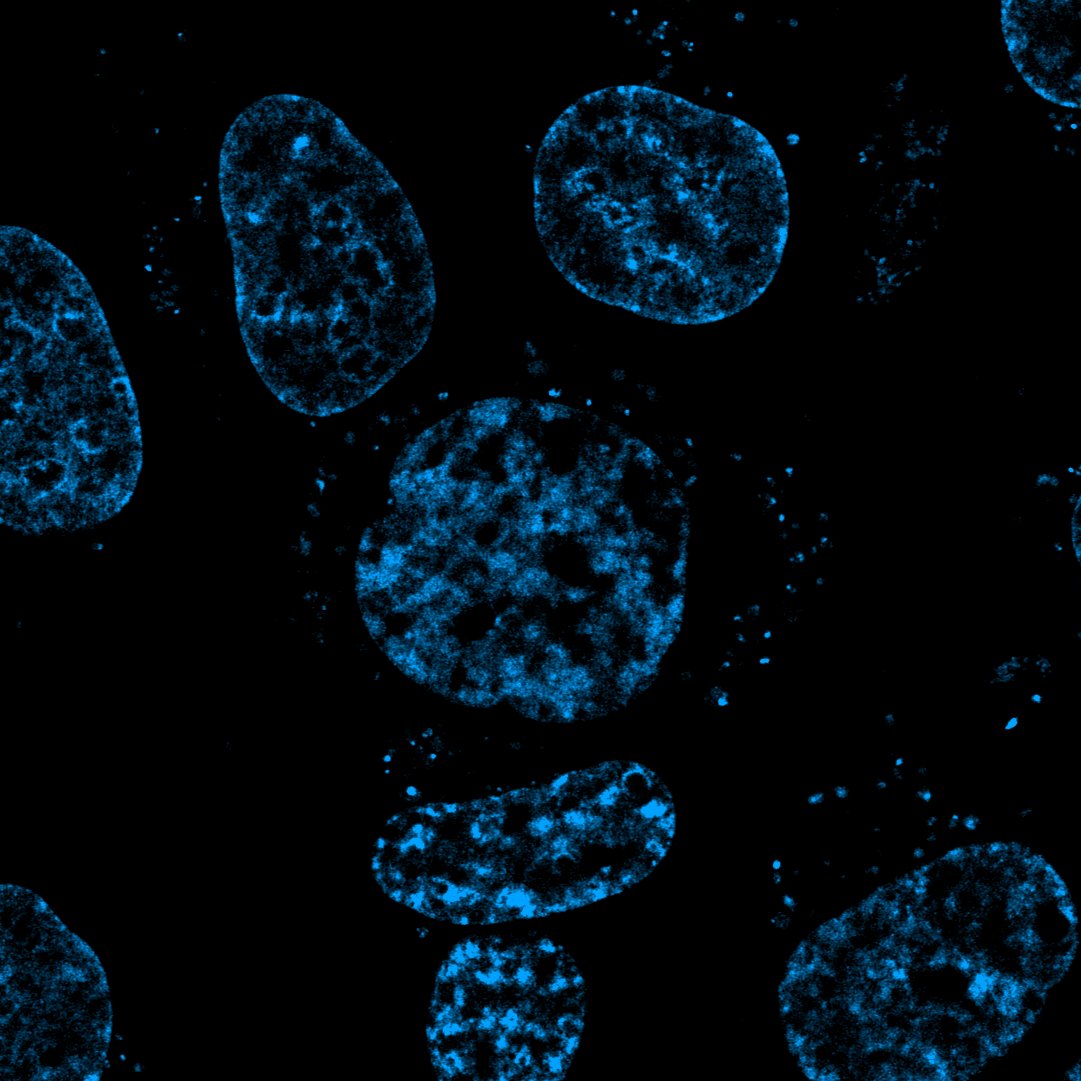

Supplement: Supplementary file 10 — Figure EV Source Data, Movie EV1 and Movie EV2 Source Data [file 44318_2025_581_MOESM10_ESM.zip › Fig EV3/S3 C/U2OS rab7-mcherry 134c-yn ALK3-yc-Nuclear.jpg]

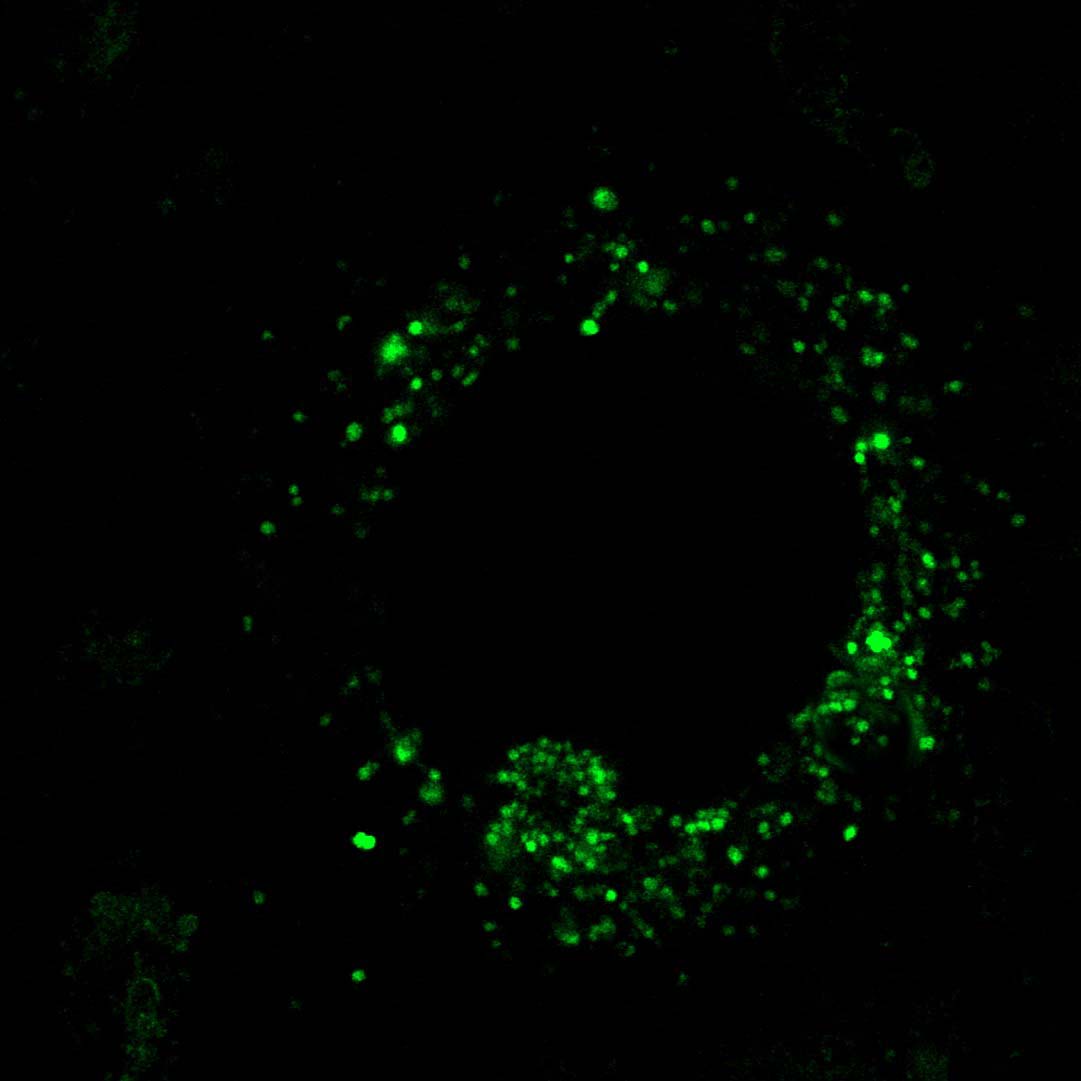

Supplement: Supplementary file 10 — Figure EV Source Data, Movie EV1 and Movie EV2 Source Data [file 44318_2025_581_MOESM10_ESM.zip › Fig EV3/S3 C/U2OS rab7-mcherry 134c-yn ALK3-yc.jpg]

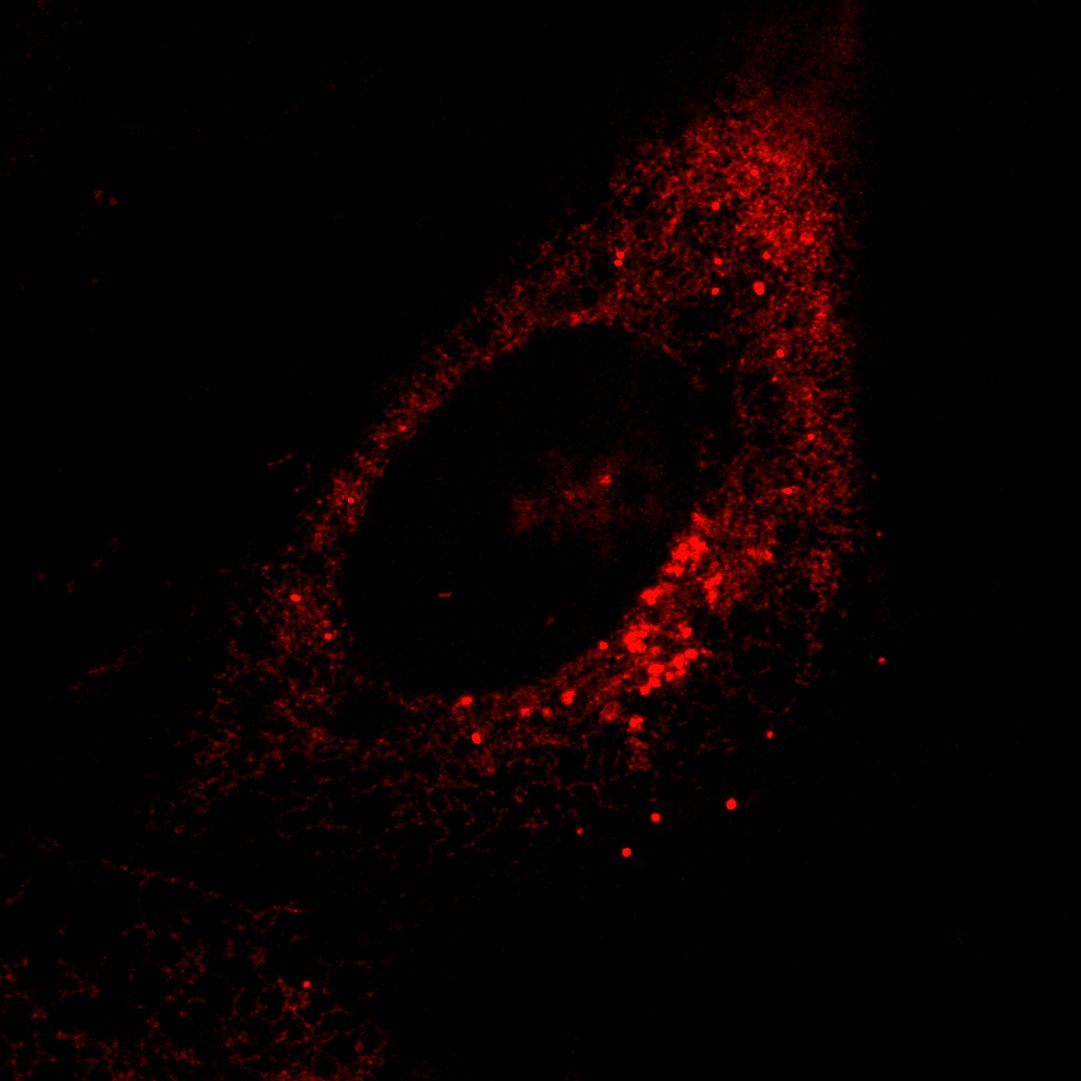

Supplement: Supplementary file 10 — Figure EV Source Data, Movie EV1 and Movie EV2 Source Data [file 44318_2025_581_MOESM10_ESM.zip › Fig EV3/S3 C/U2OS RTN4-mcherry 134c-yn ALK3-yc-mcherry.jpg]

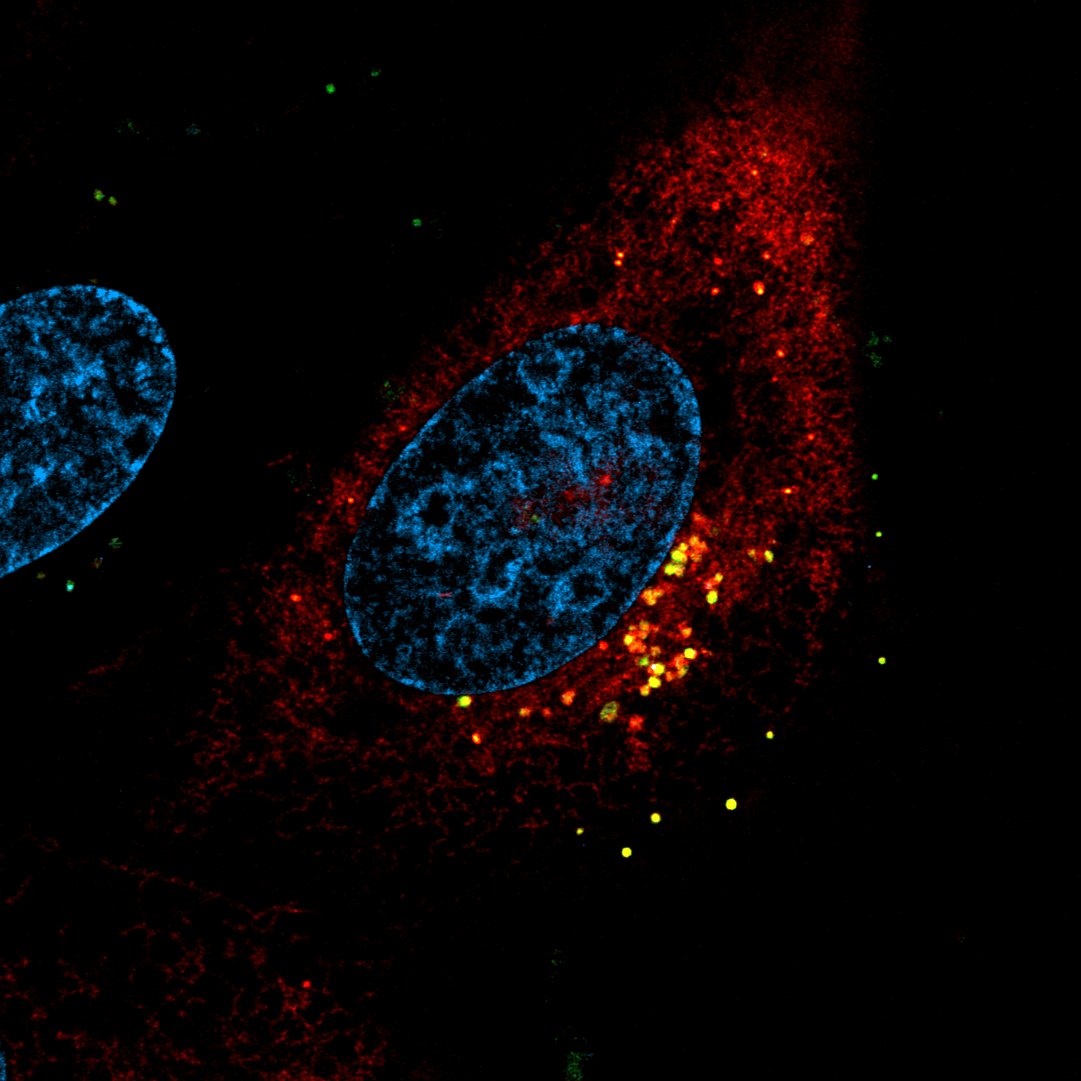

Supplement: Supplementary file 10 — Figure EV Source Data, Movie EV1 and Movie EV2 Source Data [file 44318_2025_581_MOESM10_ESM.zip › Fig EV3/S3 C/U2OS RTN4-mcherry 134c-yn ALK3-yc-merged.jpg]

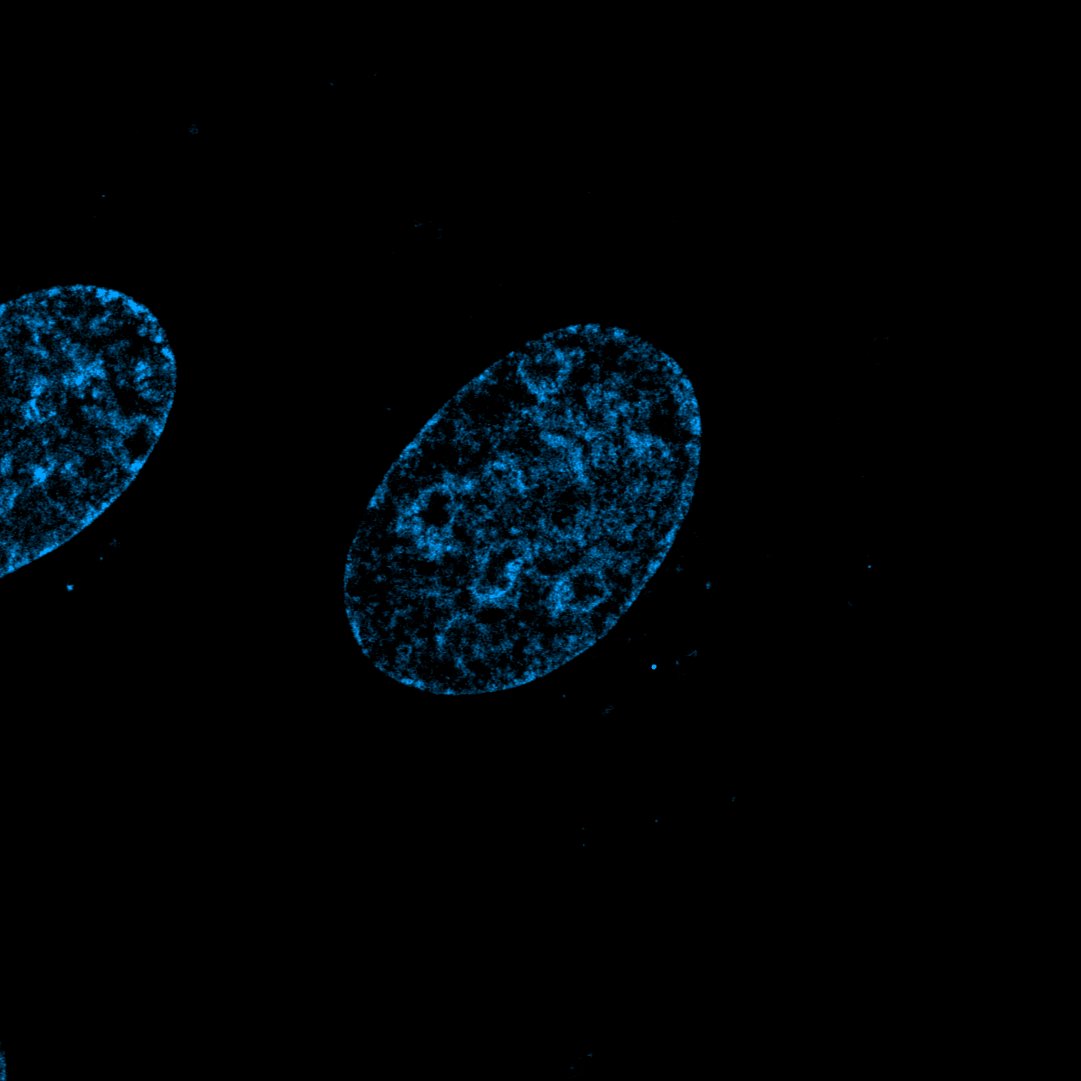

Supplement: Supplementary file 10 — Figure EV Source Data, Movie EV1 and Movie EV2 Source Data [file 44318_2025_581_MOESM10_ESM.zip › Fig EV3/S3 C/U2OS RTN4-mcherry 134c-yn ALK3-yc-Nuclear.jpg]

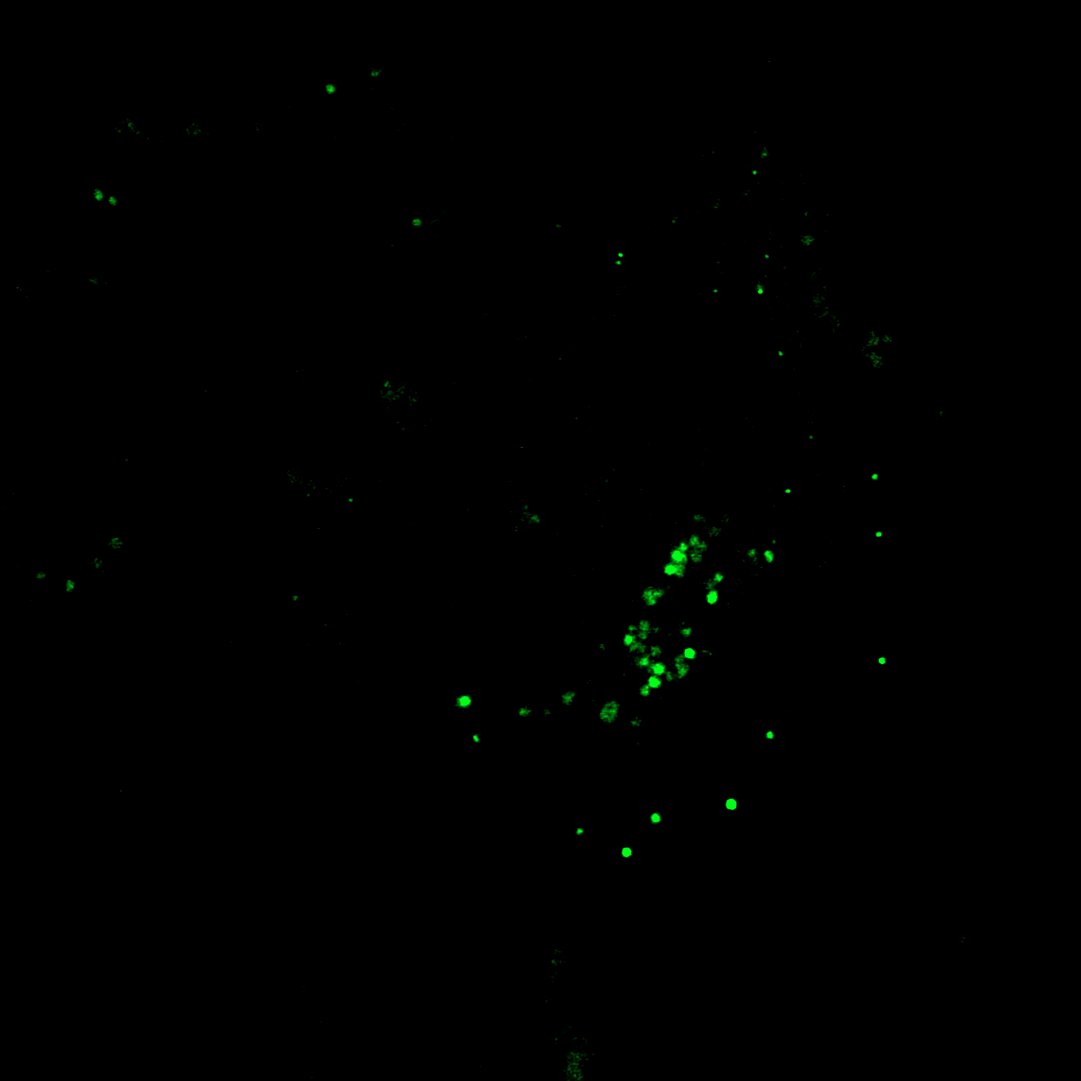

Supplement: Supplementary file 10 — Figure EV Source Data, Movie EV1 and Movie EV2 Source Data [file 44318_2025_581_MOESM10_ESM.zip › Fig EV3/S3 C/U2OS RTN4-mcherry 134c-yn ALK3-yc.jpg]

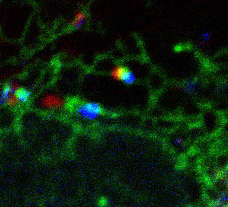

Supplement: Supplementary file 10 — Figure EV Source Data, Movie EV1 and Movie EV2 Source Data [file 44318_2025_581_MOESM10_ESM.zip › Fig EV4/S4 Movie 0 01 62.jpg]

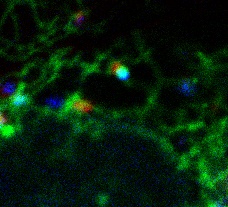

Supplement: Supplementary file 10 — Figure EV Source Data, Movie EV1 and Movie EV2 Source Data [file 44318_2025_581_MOESM10_ESM.zip › Fig EV4/S4 Movie 0 01 81.jpg]

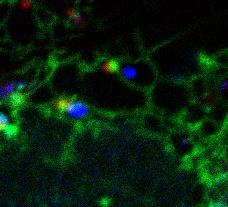

Supplement: Supplementary file 10 — Figure EV Source Data, Movie EV1 and Movie EV2 Source Data [file 44318_2025_581_MOESM10_ESM.zip › Fig EV4/S4 Movie 0 01 82.jpg]

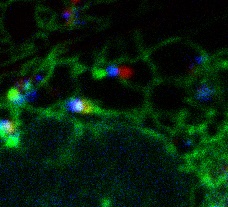

Supplement: Supplementary file 10 — Figure EV Source Data, Movie EV1 and Movie EV2 Source Data [file 44318_2025_581_MOESM10_ESM.zip › Fig EV4/S4 Movie 0 02 22.jpg]

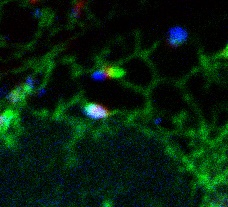

Supplement: Supplementary file 10 — Figure EV Source Data, Movie EV1 and Movie EV2 Source Data [file 44318_2025_581_MOESM10_ESM.zip › Fig EV4/S4 Movie 0 02 66.jpg]

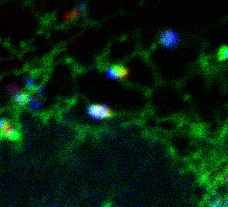

Supplement: Supplementary file 10 — Figure EV Source Data, Movie EV1 and Movie EV2 Source Data [file 44318_2025_581_MOESM10_ESM.zip › Fig EV4/S4 Movie 0 02 82.jpg]
