# Supplementary figures and images for: Reticulophagy receptor FAM134C restrains BMP receptor signaling (part 3 of 3)
Source: EMBO J. 2025 Oct 20;44(23):7154–80. doi: 10.1038/s44318-025-00581-3 (PMC12669696; doi:10.1038/s44318-025-00581-3)

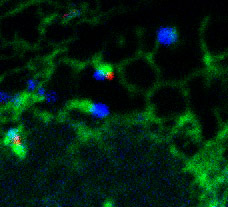

Supplement: Supplementary file 10 — Figure EV Source Data, Movie EV1 and Movie EV2 Source Data [file 44318_2025_581_MOESM10_ESM.zip › Fig EV4/S4 Movie 0 03 23.jpg]

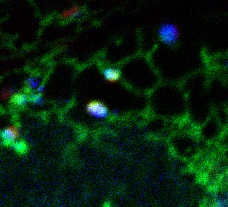

Supplement: Supplementary file 10 — Figure EV Source Data, Movie EV1 and Movie EV2 Source Data [file 44318_2025_581_MOESM10_ESM.zip › Fig EV4/S4 Movie 0 03 82.jpg]

IB: BMPR1a

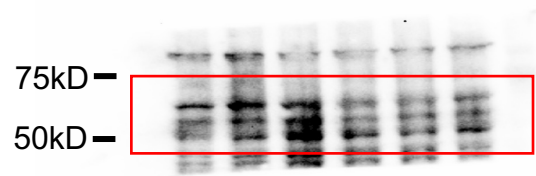

IB: FAM134C

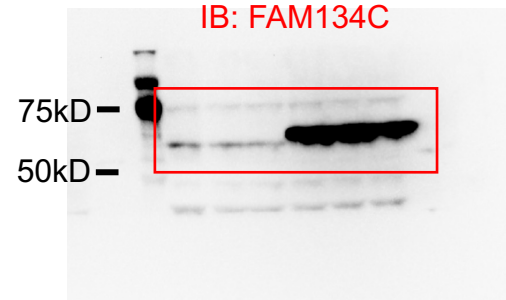

IB: p-S1/5/8

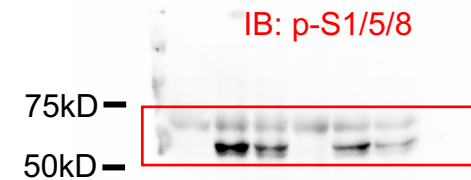

IB : GAPDH

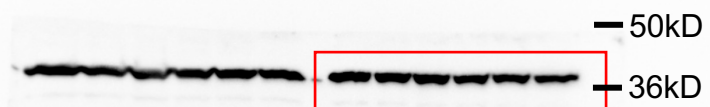

IB: ID1

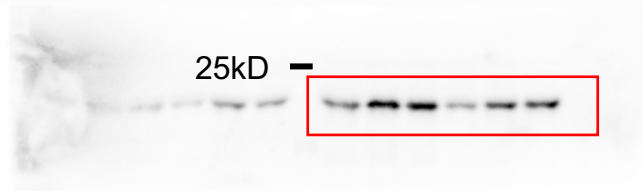

IB: Smad1

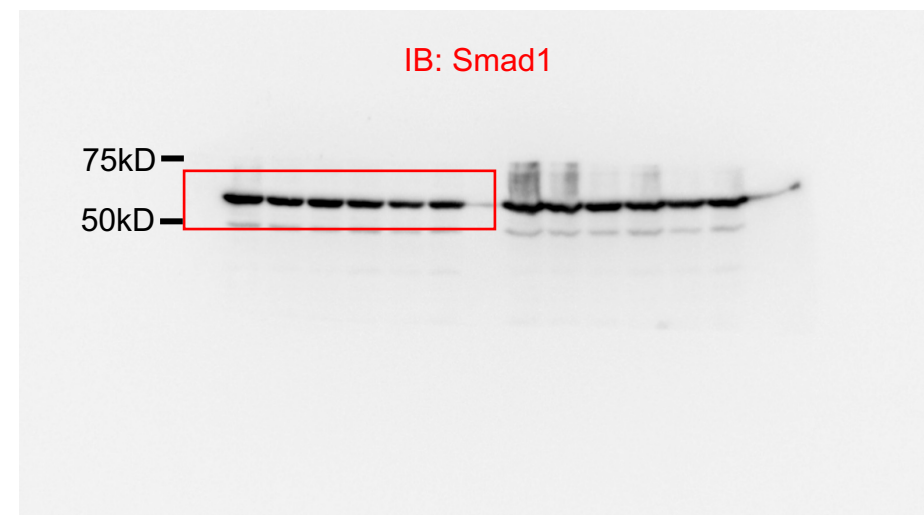

BMPR1a

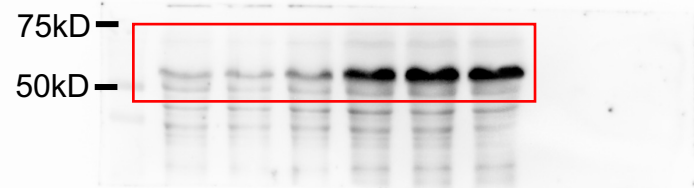

FAM134C

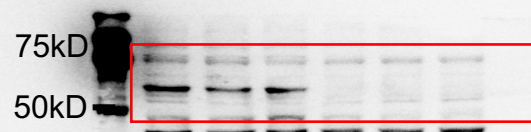

p-S1/5/8

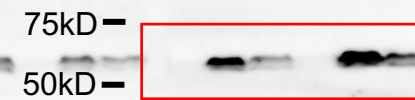

GAPDH

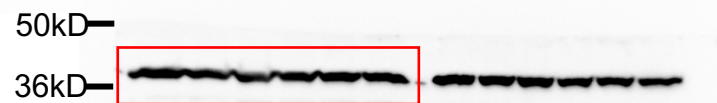

ID1

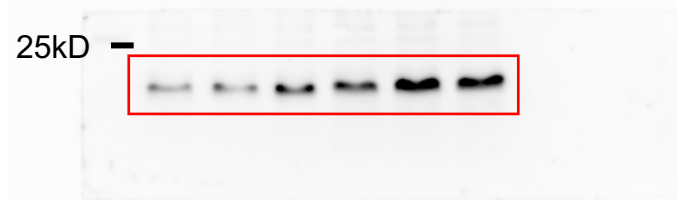

Smad1

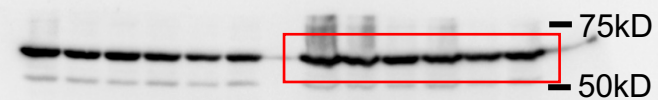

Supplement: Supplementary file 10 — Figure EV Source Data, Movie EV1 and Movie EV2 Source Data [file 44318_2025_581_MOESM10_ESM.zip › Fig EV5/S5 A blot.pdf]

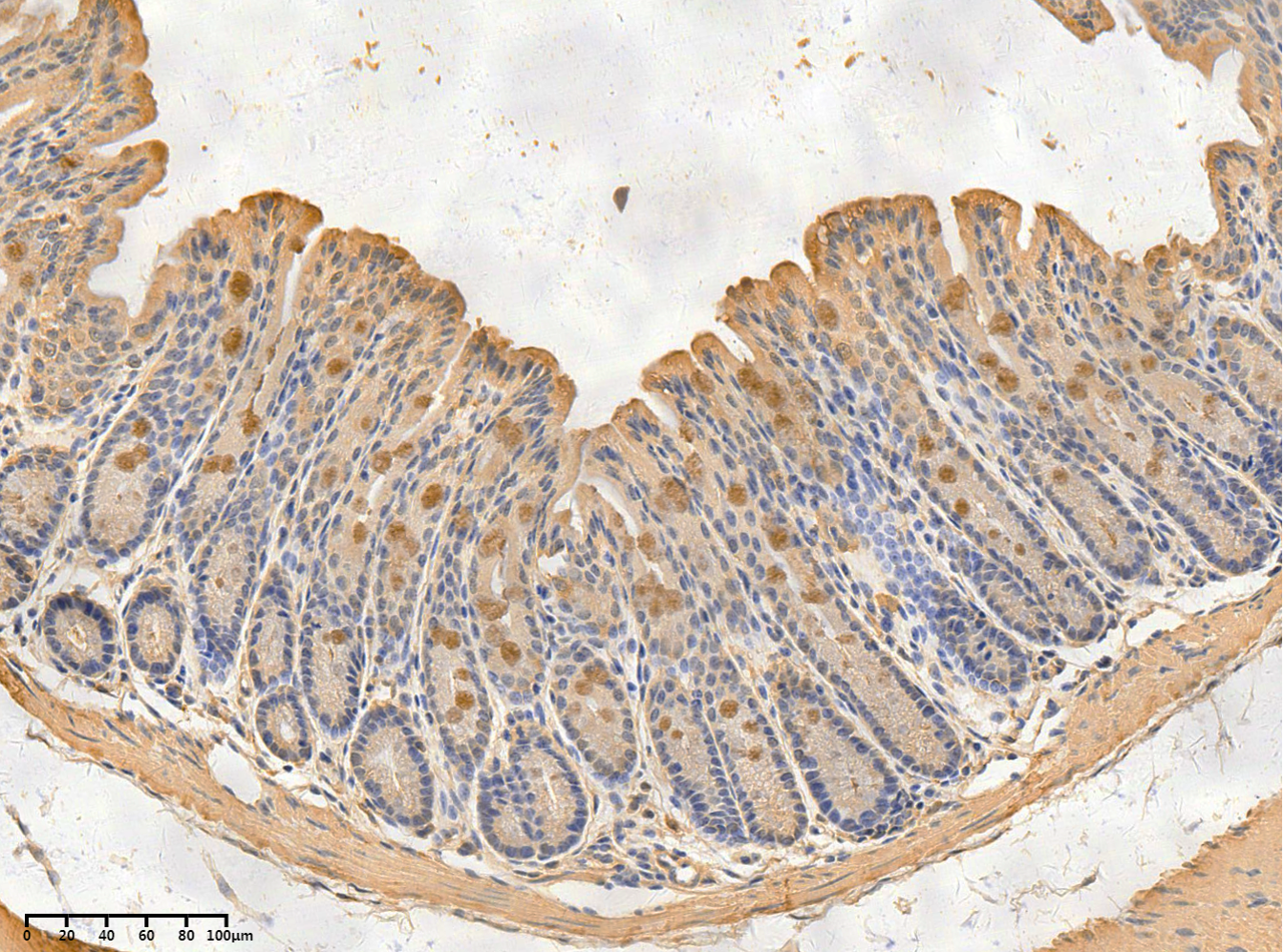

Supplement: Supplementary file 10 — Figure EV Source Data, Movie EV1 and Movie EV2 Source Data [file 44318_2025_581_MOESM10_ESM.zip › Fig EV5/S5 B/FAM134C KO BMPR1a colon.tif]

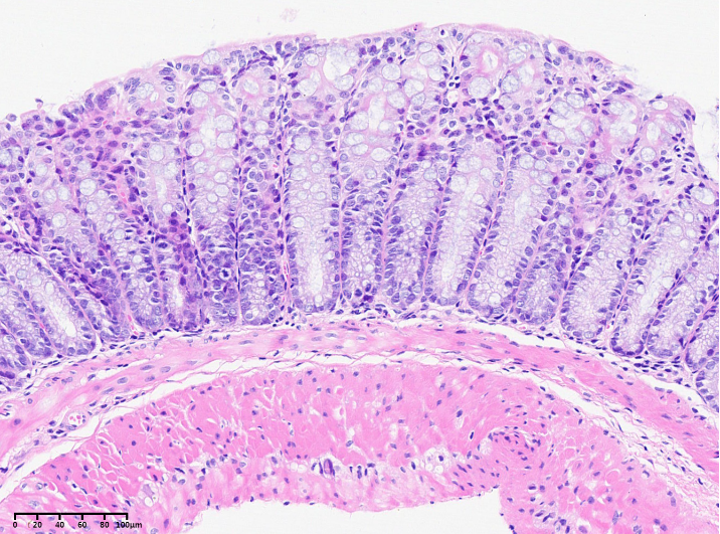

Supplement: Supplementary file 10 — Figure EV Source Data, Movie EV1 and Movie EV2 Source Data [file 44318_2025_581_MOESM10_ESM.zip › Fig EV5/S5 B/FAM134C KO HE colon.tif]

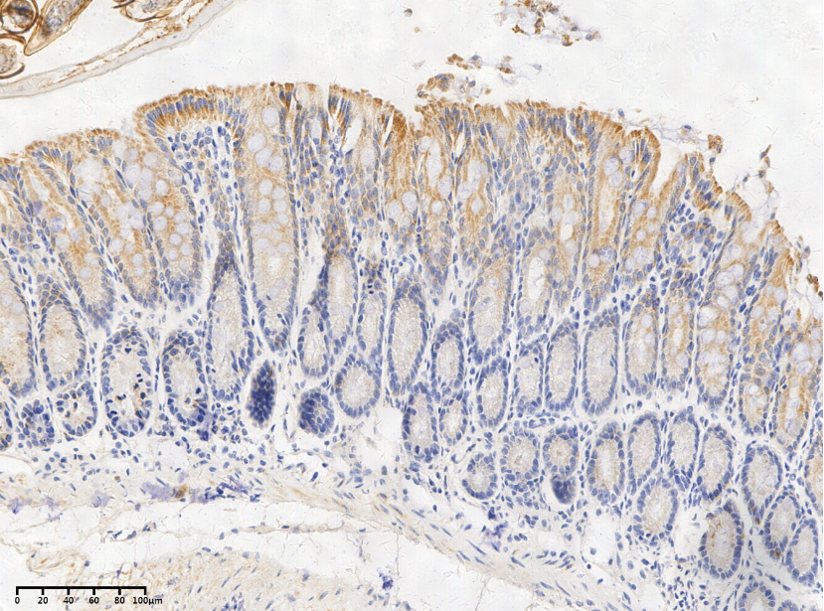

Supplement: Supplementary file 10 — Figure EV Source Data, Movie EV1 and Movie EV2 Source Data [file 44318_2025_581_MOESM10_ESM.zip › Fig EV5/S5 B/FAM134C KO p-Smad158 colon.tif]

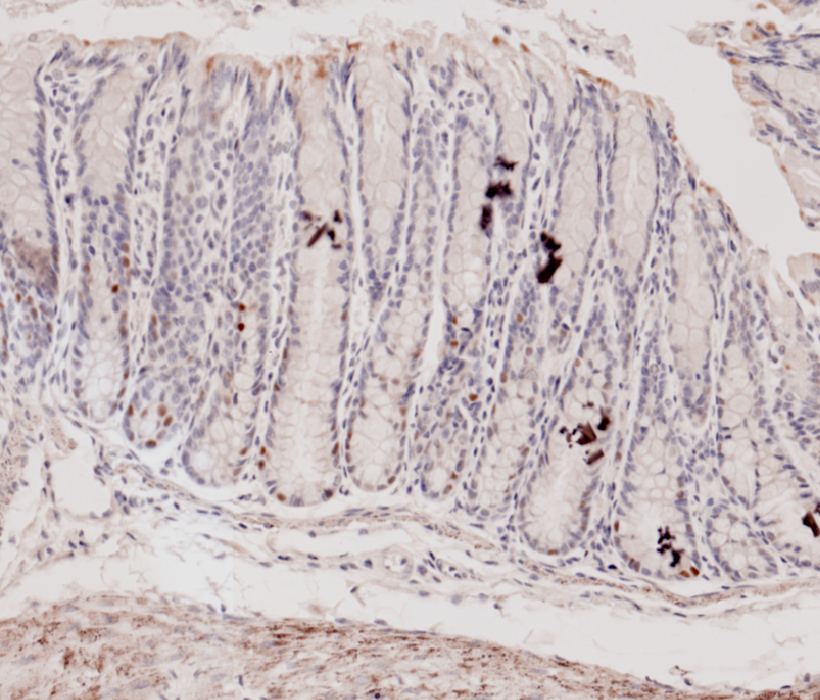

Supplement: Supplementary file 10 — Figure EV Source Data, Movie EV1 and Movie EV2 Source Data [file 44318_2025_581_MOESM10_ESM.zip › Fig EV5/S5 B/FAM134C KO Sox9 colon.tif]

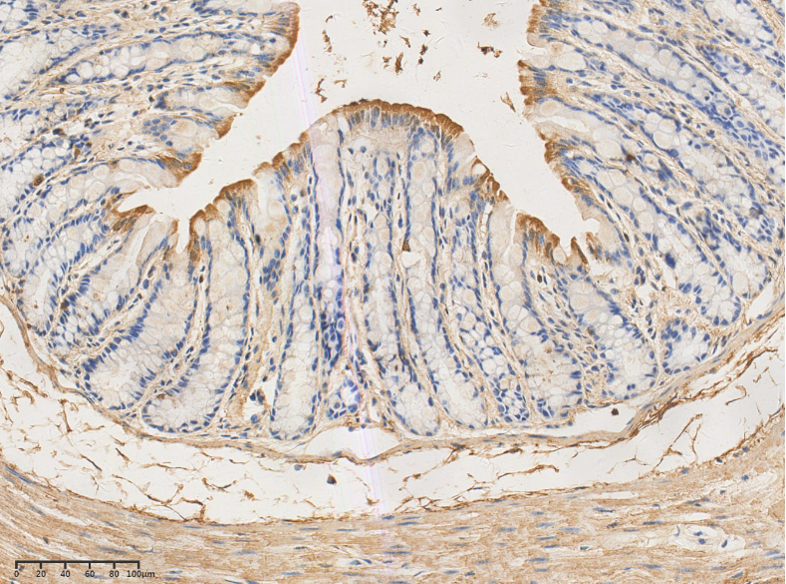

Supplement: Supplementary file 10 — Figure EV Source Data, Movie EV1 and Movie EV2 Source Data [file 44318_2025_581_MOESM10_ESM.zip › Fig EV5/S5 B/FAM134C WT BMPR1a colon.tif]

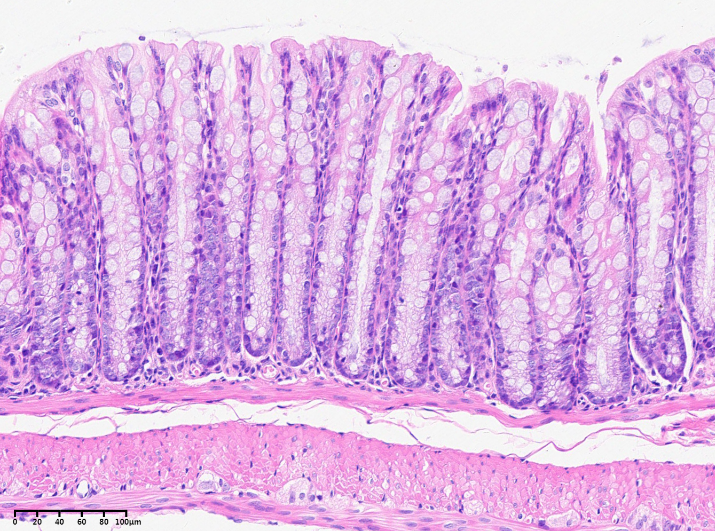

Supplement: Supplementary file 10 — Figure EV Source Data, Movie EV1 and Movie EV2 Source Data [file 44318_2025_581_MOESM10_ESM.zip › Fig EV5/S5 B/FAM134C WT HE colon.tif]

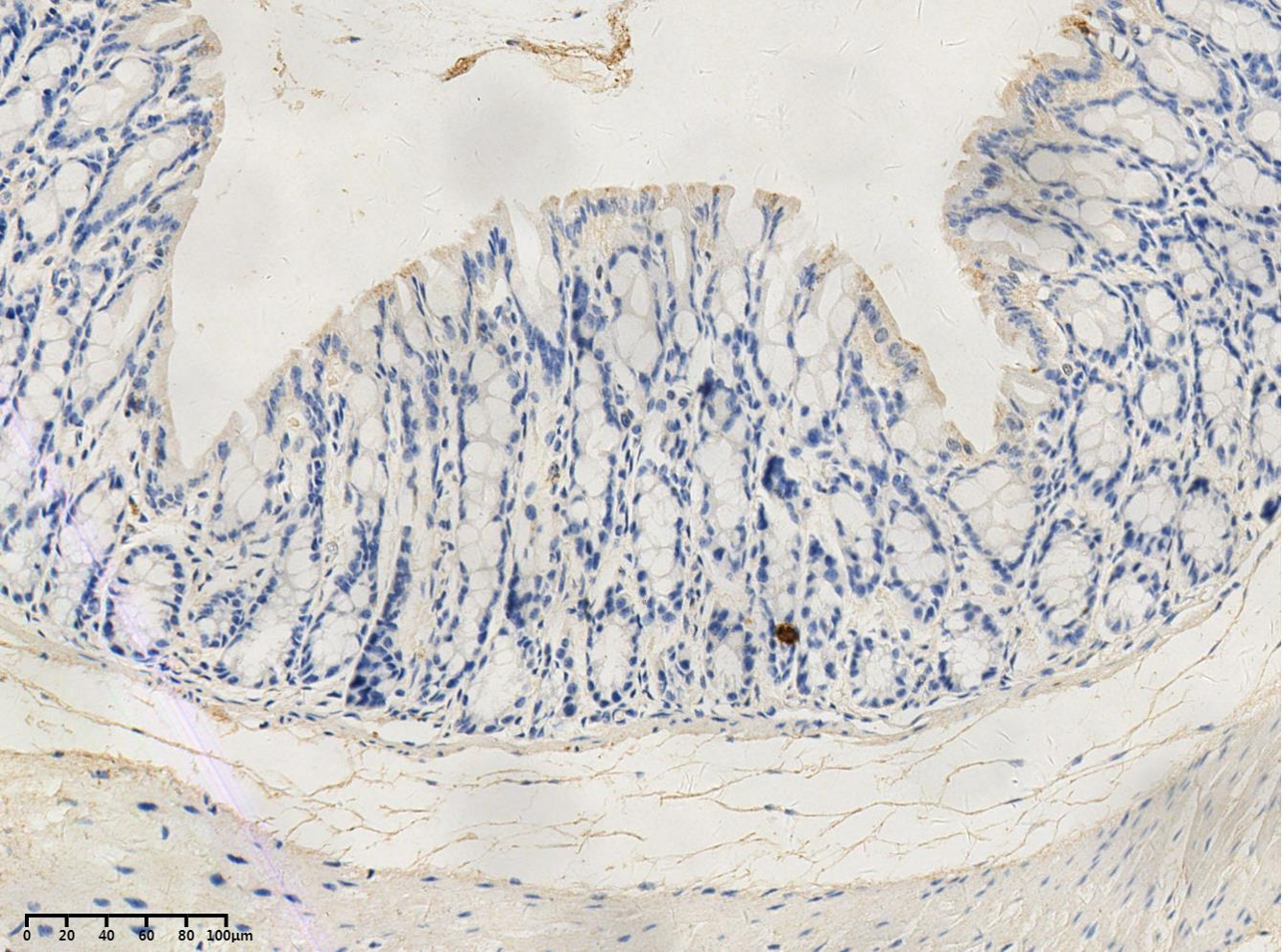

Supplement: Supplementary file 10 — Figure EV Source Data, Movie EV1 and Movie EV2 Source Data [file 44318_2025_581_MOESM10_ESM.zip › Fig EV5/S5 B/FAM134C WT p-Smad158 colon.tif]

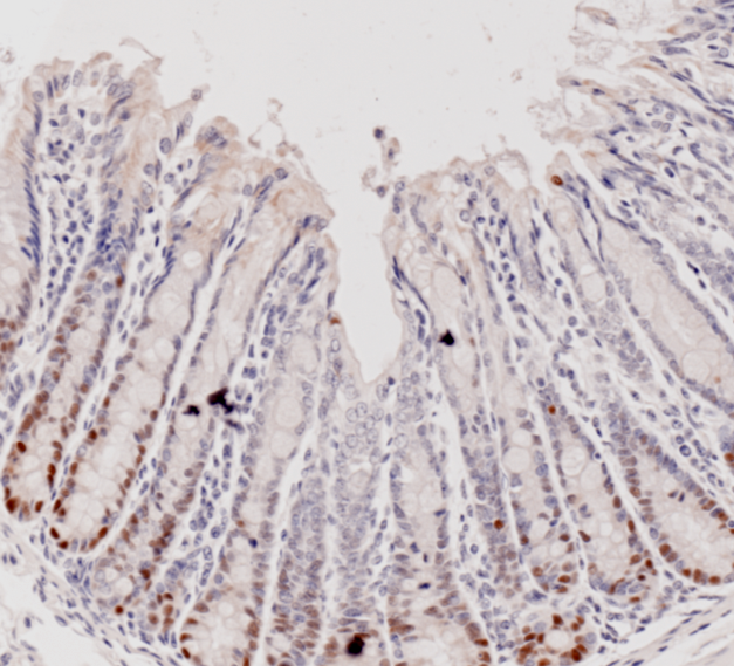

Supplement: Supplementary file 10 — Figure EV Source Data, Movie EV1 and Movie EV2 Source Data [file 44318_2025_581_MOESM10_ESM.zip › Fig EV5/S5 B/FAM134C WT Sox9 colon.tif]

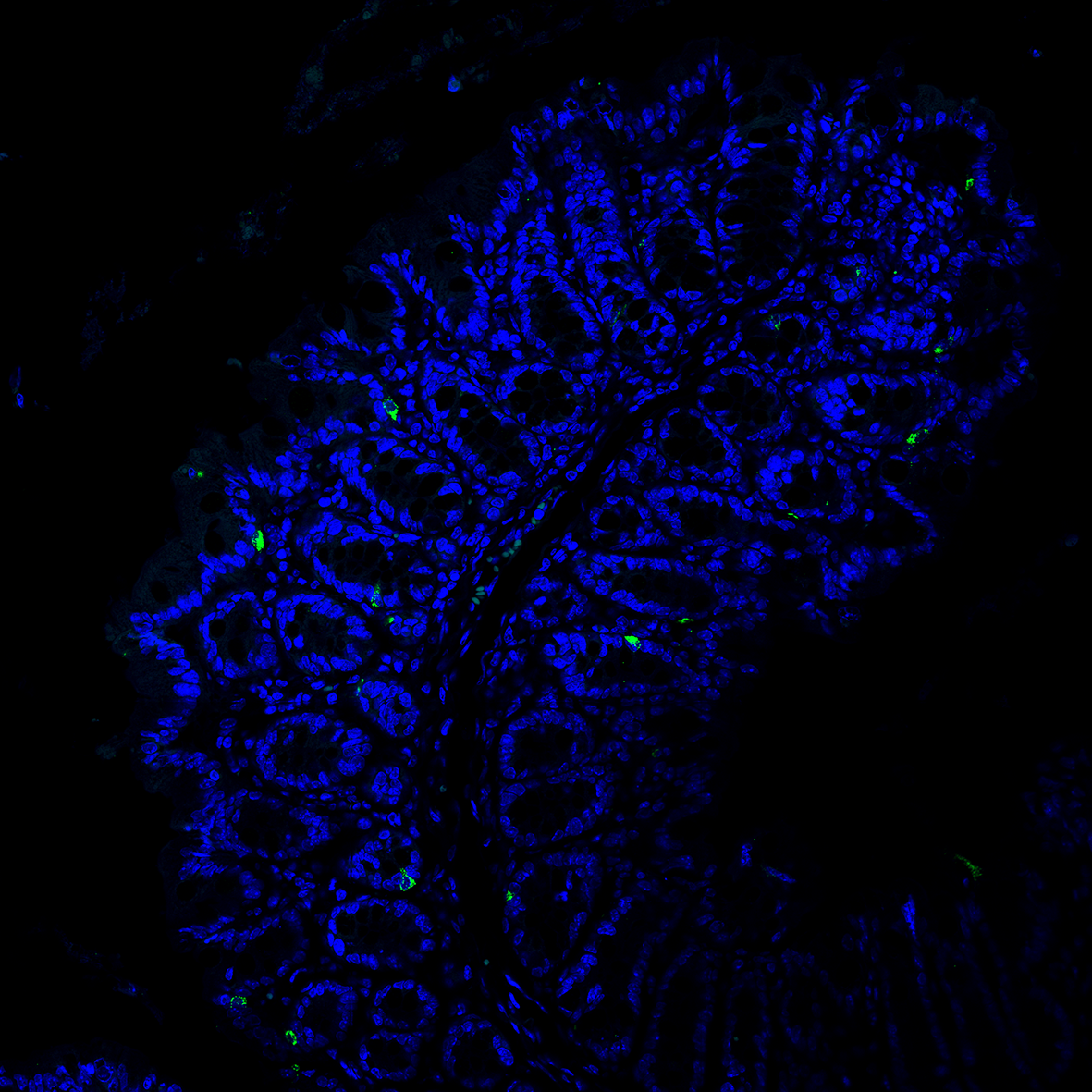

Supplement: Supplementary file 10 — Figure EV Source Data, Movie EV1 and Movie EV2 Source Data [file 44318_2025_581_MOESM10_ESM.zip › Fig EV5/S5 C/FAM134c KO colon chga.tif]

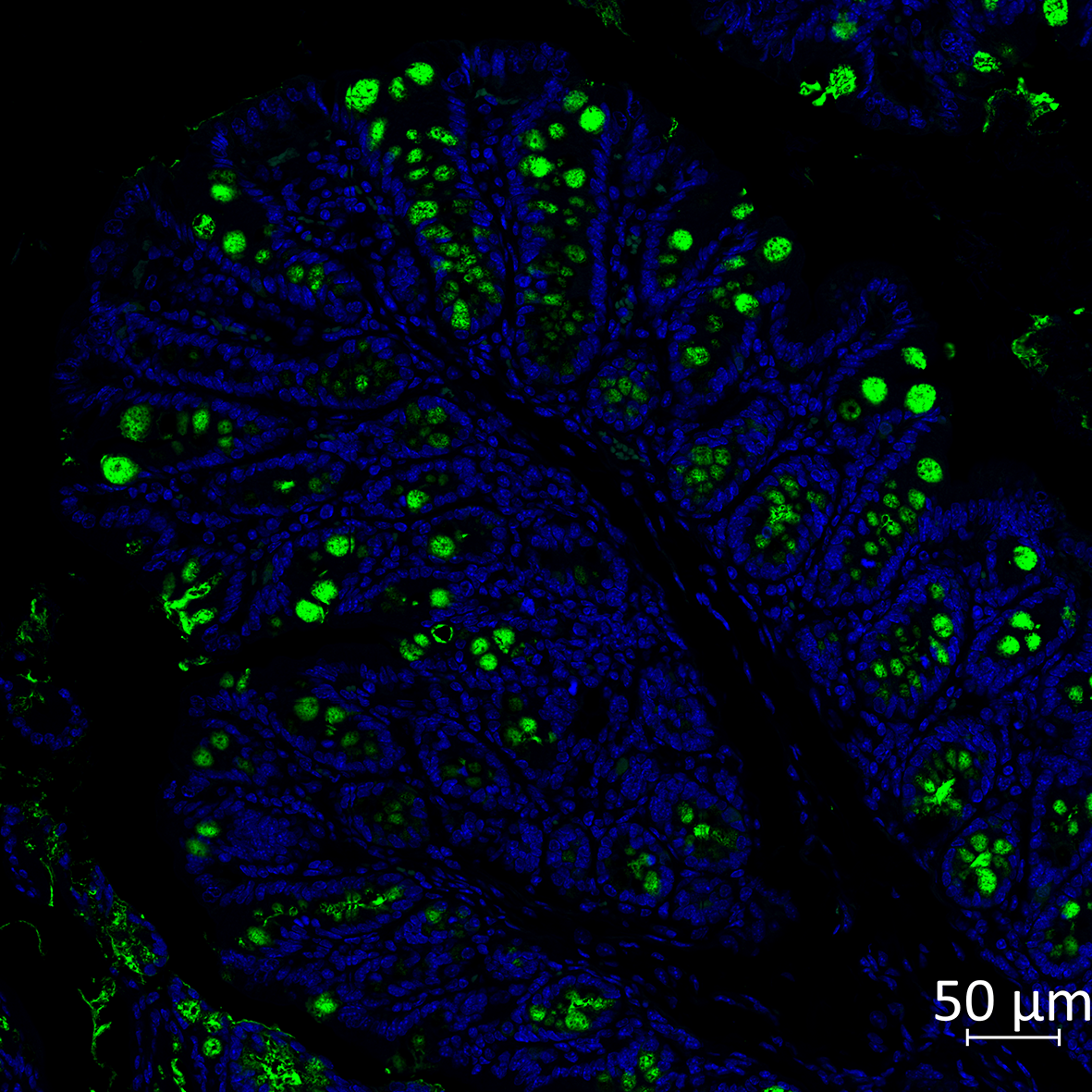

Supplement: Supplementary file 10 — Figure EV Source Data, Movie EV1 and Movie EV2 Source Data [file 44318_2025_581_MOESM10_ESM.zip › Fig EV5/S5 C/FAM134c KO colon Muc2.tif]

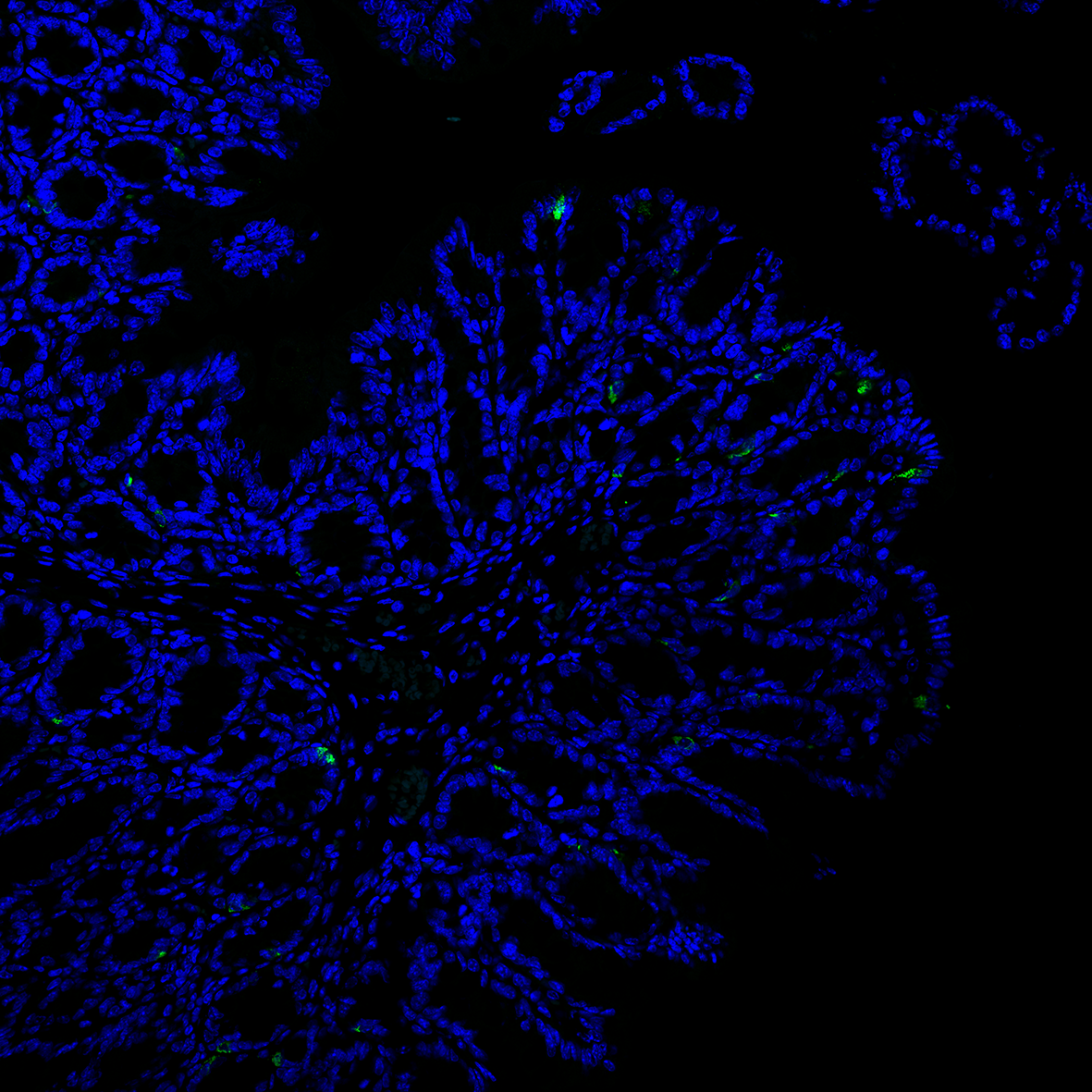

Supplement: Supplementary file 10 — Figure EV Source Data, Movie EV1 and Movie EV2 Source Data [file 44318_2025_581_MOESM10_ESM.zip › Fig EV5/S5 C/FAM134c WT colon chga.tif]

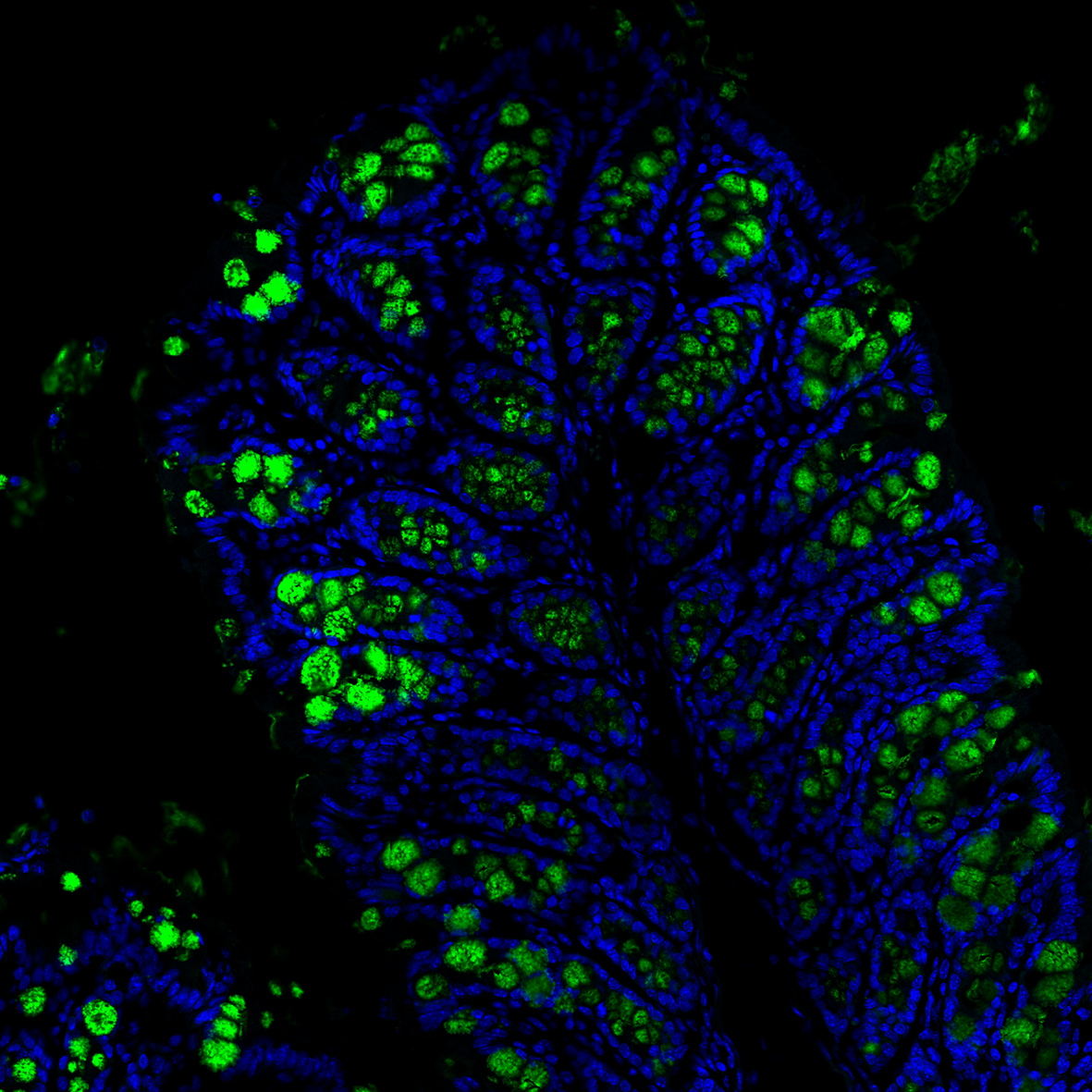

Supplement: Supplementary file 10 — Figure EV Source Data, Movie EV1 and Movie EV2 Source Data [file 44318_2025_581_MOESM10_ESM.zip › Fig EV5/S5 C/FAM134c WT colon MUC2.tif]

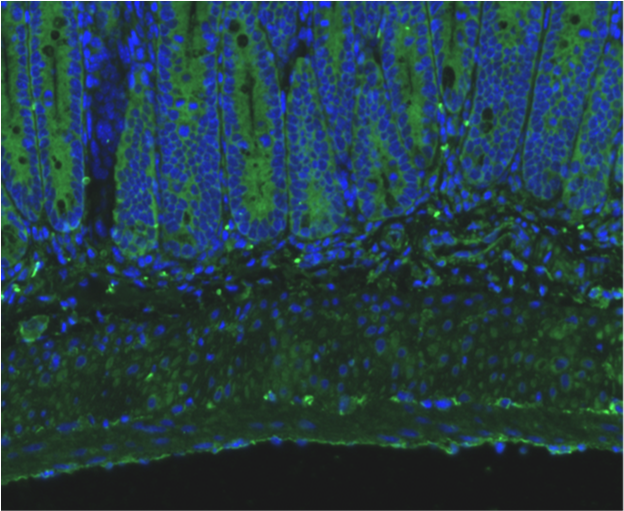

Supplement: Supplementary file 10 — Figure EV Source Data, Movie EV1 and Movie EV2 Source Data [file 44318_2025_581_MOESM10_ESM.zip › Fig EV5/S5 E/KO Fasting BMPR1A.tif]

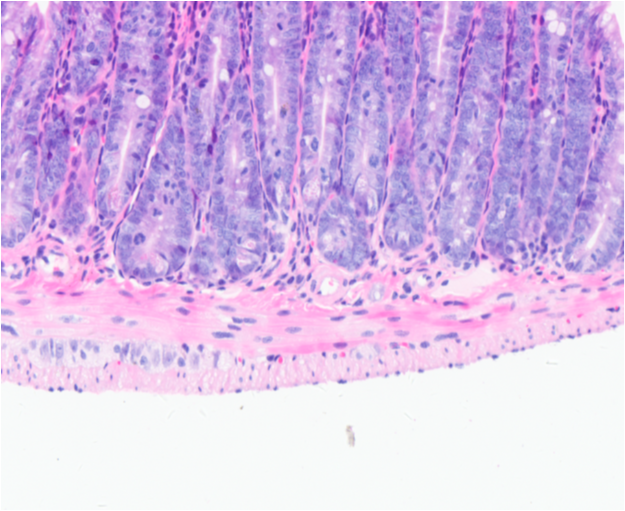

Supplement: Supplementary file 10 — Figure EV Source Data, Movie EV1 and Movie EV2 Source Data [file 44318_2025_581_MOESM10_ESM.zip › Fig EV5/S5 E/KO Fasting HE.tif]

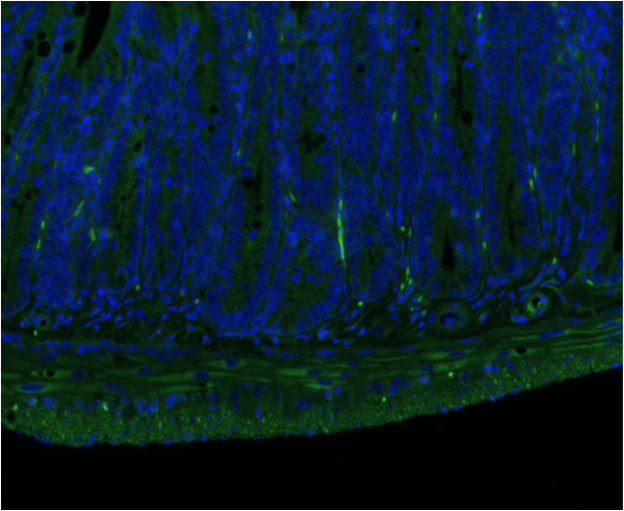

Supplement: Supplementary file 10 — Figure EV Source Data, Movie EV1 and Movie EV2 Source Data [file 44318_2025_581_MOESM10_ESM.zip › Fig EV5/S5 E/KO Fasting p62.tif]

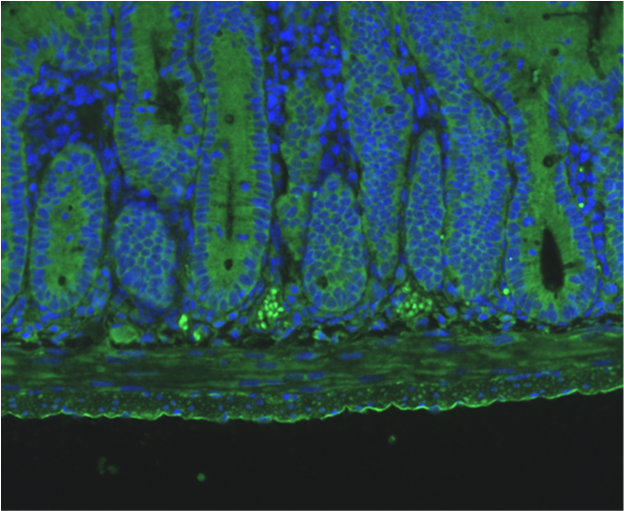

Supplement: Supplementary file 10 — Figure EV Source Data, Movie EV1 and Movie EV2 Source Data [file 44318_2025_581_MOESM10_ESM.zip › Fig EV5/S5 E/KO Feed BMPR1A.tif]

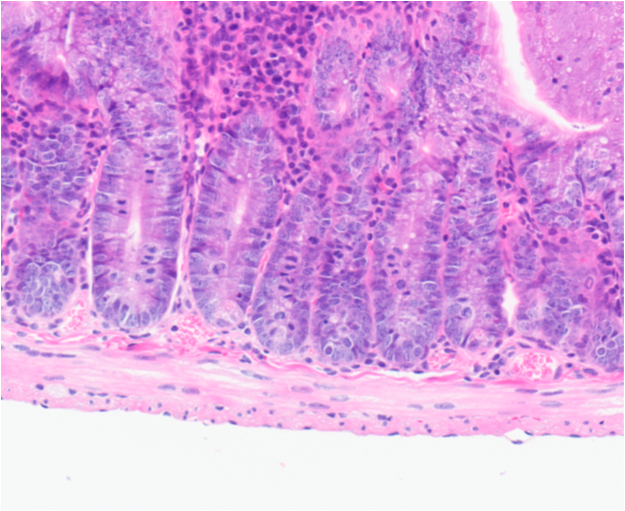

Supplement: Supplementary file 10 — Figure EV Source Data, Movie EV1 and Movie EV2 Source Data [file 44318_2025_581_MOESM10_ESM.zip › Fig EV5/S5 E/KO Feed HE.tif]

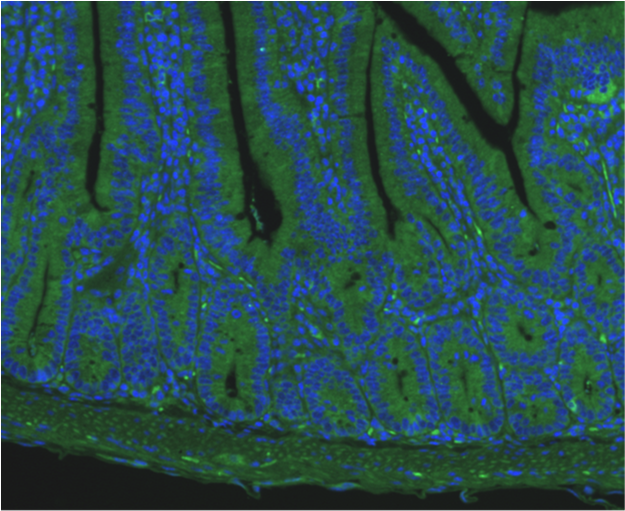

Supplement: Supplementary file 10 — Figure EV Source Data, Movie EV1 and Movie EV2 Source Data [file 44318_2025_581_MOESM10_ESM.zip › Fig EV5/S5 E/KO Feed p62.tif]

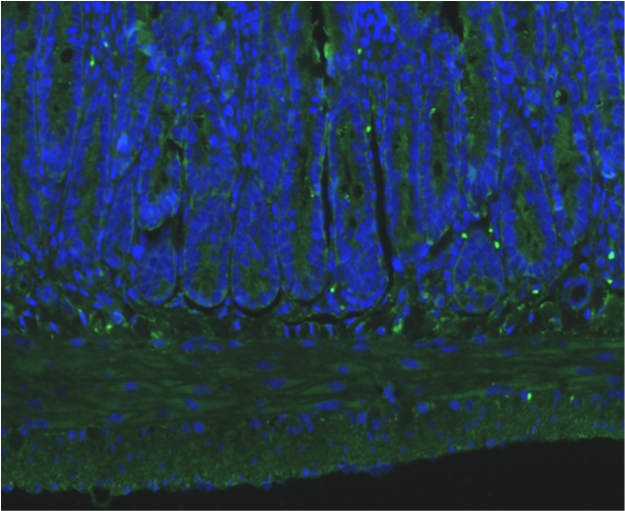

Supplement: Supplementary file 10 — Figure EV Source Data, Movie EV1 and Movie EV2 Source Data [file 44318_2025_581_MOESM10_ESM.zip › Fig EV5/S5 E/WT Fasting BMPR1A.tif]

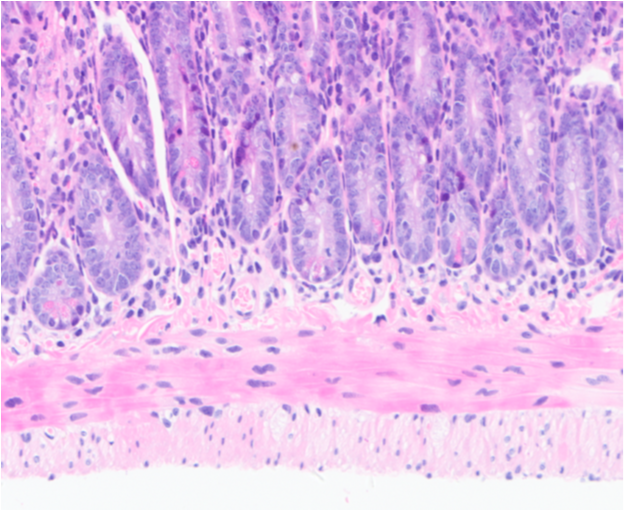

Supplement: Supplementary file 10 — Figure EV Source Data, Movie EV1 and Movie EV2 Source Data [file 44318_2025_581_MOESM10_ESM.zip › Fig EV5/S5 E/WT Fasting HE.tif]

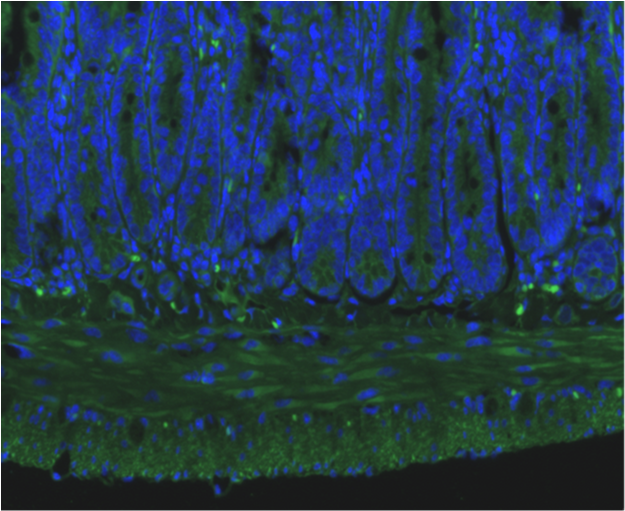

Supplement: Supplementary file 10 — Figure EV Source Data, Movie EV1 and Movie EV2 Source Data [file 44318_2025_581_MOESM10_ESM.zip › Fig EV5/S5 E/WT fasting p62.tif]

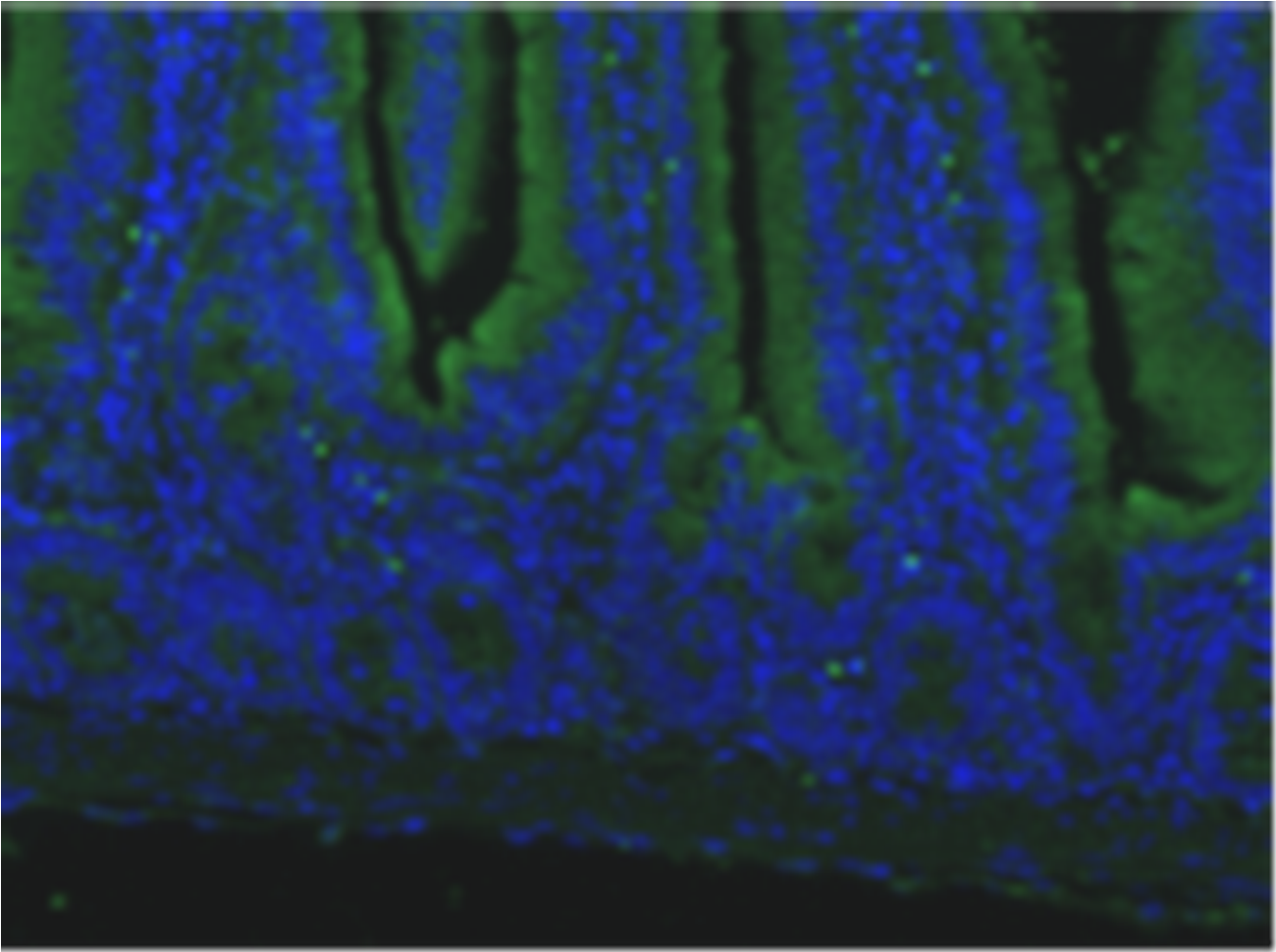

Supplement: Supplementary file 10 — Figure EV Source Data, Movie EV1 and Movie EV2 Source Data [file 44318_2025_581_MOESM10_ESM.zip › Fig EV5/S5 E/WT Feed BMPR1A.tif]

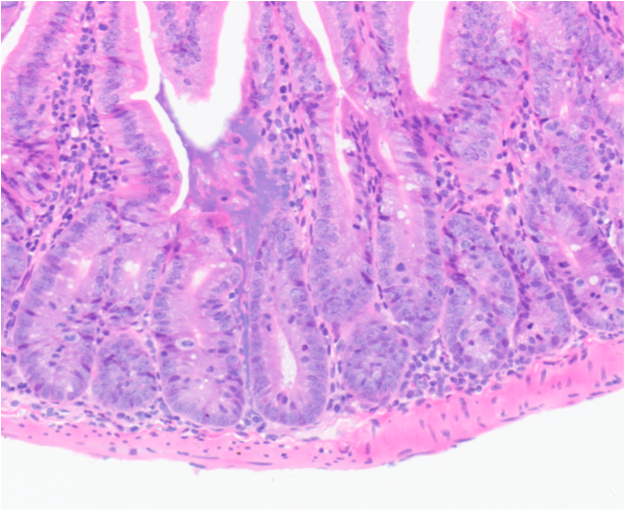

Supplement: Supplementary file 10 — Figure EV Source Data, Movie EV1 and Movie EV2 Source Data [file 44318_2025_581_MOESM10_ESM.zip › Fig EV5/S5 E/WT Feed HE.tif]

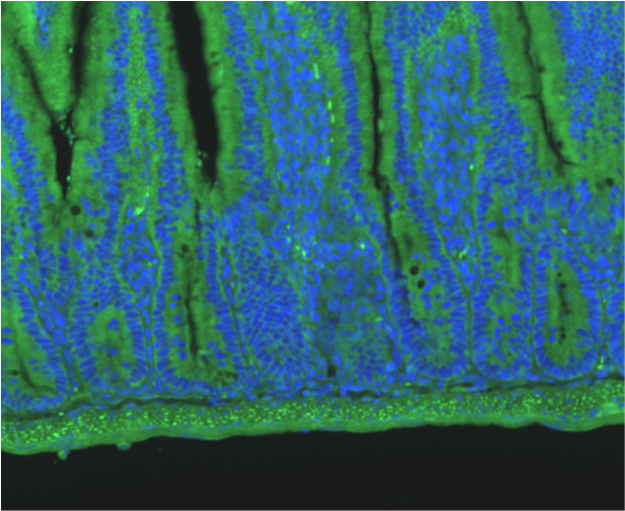

Supplement: Supplementary file 10 — Figure EV Source Data, Movie EV1 and Movie EV2 Source Data [file 44318_2025_581_MOESM10_ESM.zip › Fig EV5/S5 E/WT Feed p62.tif]
