# Supplementary material for: Reticulophagy receptor FAM134C restrains BMP receptor signaling
Source: EMBO J. 2025 Oct 20;44(23):7154–80. doi: 10.1038/s44318-025-00581-3 (PMC12669696; doi:10.1038/s44318-025-00581-3)
Supplement: Supplementary file 11 — Expanded View Figures [file 44318_2025_581_MOESM11_ESM.pdf]

Expanded View Figures

Figure EV1. FAM134C degrades BMPR1A by autophagy.

(**A**, **B**) Quantitative RT-PCR analysis of FAM134C and BMPR1A mRNAs in U2OS cells stably expressing FAM134C-FLAG or carrying an empty vector control. mean ± SD ($n = 3$ independent experiments). (**C**) Expression of FAM134C and GAPDH were measured in wildtype (WT) or FAM134C-KO U2OS cells by Western blotting. (**D**) Quantitative RT-PCR analysis of BMPR1A mRNA in WT or FAM134C-KO U2OS cells. mean ± SD ($n = 3$ independent experiments). (**E**) HEK293T cells were co-transfected with vectors encoding FAM134C-FLAG and ALK1-HA, ALK2-HA, ALK3-HA, ALK6-HA, or BMPR2-HA, then FAM134C was detected by anti-FLAG antibody and the protein ALK1, ALK2, ALK3, ALK6, or BMPR2 was detected by HA antibody. (**F**) Quantitative RT-PCR analysis of ATG5 mRNA in U2OS with stable knockdown of ATG5 or control. mean ± SD ($n = 3$ independent experiments). (**G**) Quantitative RT-PCR analysis of BECN1 mRNA in U2OS with stable knockdown of BECN1 or control. mean ± SD ($n = 3$ independent experiments). (**H**) BECN1 downregulation blocks the degradation of BMPR1A by FAM134C. U2OS cells with BECN1 stable knockdown were transfected with FAM134C-FLAG or the FLAG vector. Twenty-four hours after transfection, cells were treated with BMP2 (50 ng/ml) for 0.5 or 1 h. Levels of BMPR1A, p-Smad1/5/8, Smad1, FAM134C, and GAPDH were measured by Western blotting. Source data are available online for this figure.

© The Author(s)

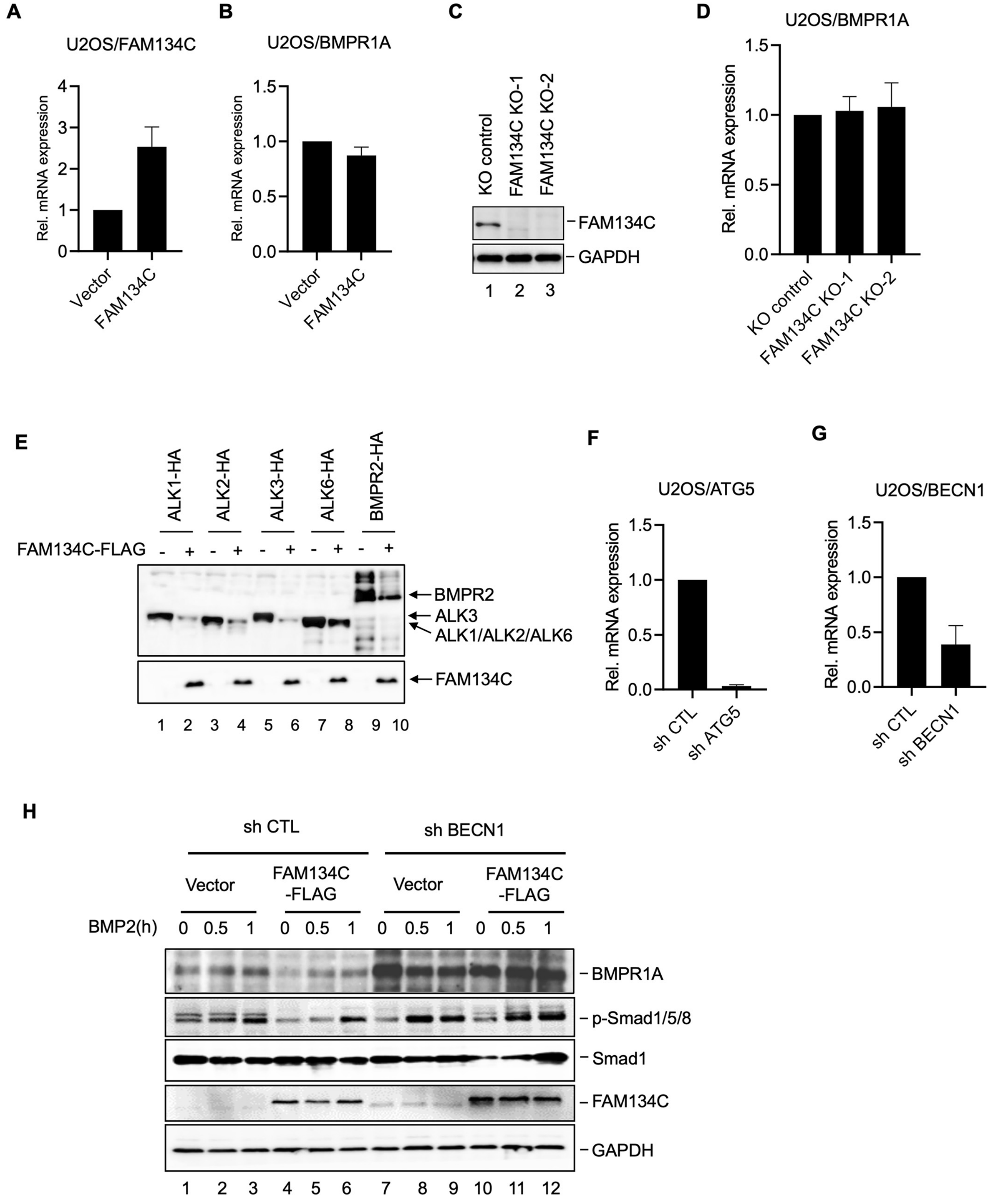
A
U2OS/FAM134C
Rel. mRNA expression
Vector
FAM134C
B
U2OS/BMPR1A
Rel. mRNA expression
Vector
FAM134C
C
KO control
FAM134C KO-1
FAM134C KO-2
FAM134C
GAPDH
1 2 3
D
U2OS/BMPR1A
Rel. mRNA expression
KO control
FAM134C KO-1
FAM134C KO-2
E
ALK1-HA
ALK2-HA
ALK3-HA
ALK6-HA
BMPR2-HA
FAM134C-FLAG
BMPR2
ALK3
ALK1/ALK2/ALK6
FAM134C
1 2 3 4 5 6 7 8 9 10
F
U2OS/ATG5
Rel. mRNA expression
sh CTL
sh ATG5
G
U2OS/BECN1
Rel. mRNA expression
sh CTL
sh BECN1
H
sh CTL
sh BECN1
Vector
FAM134C -FLAG
BMP2(h)
BMPR1A
p-Smad1/5/8
Smad1
FAM134C
GAPDH
1 2 3 4 5 6 7 8 9 10 11 12

 © The Author(s)

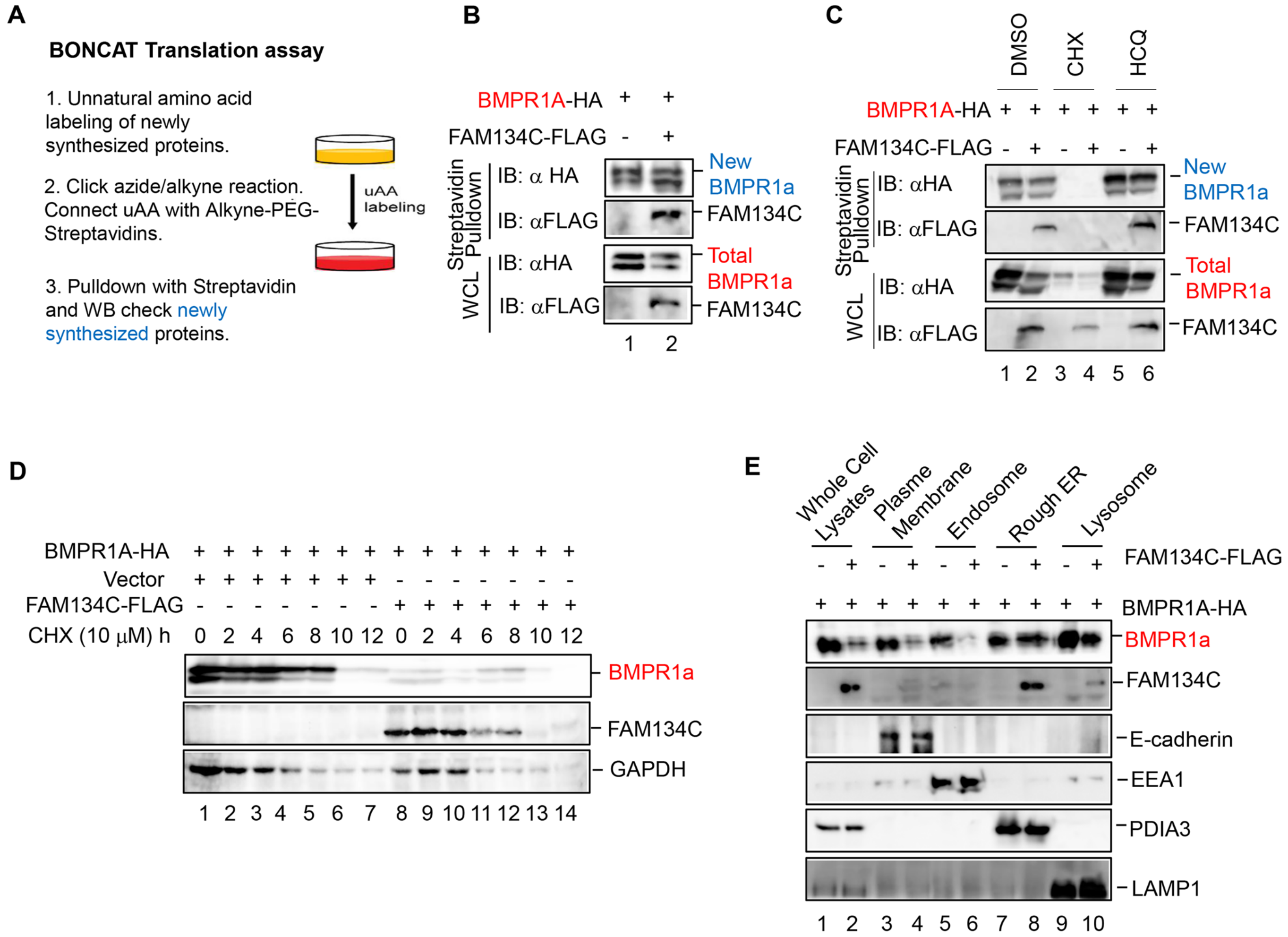

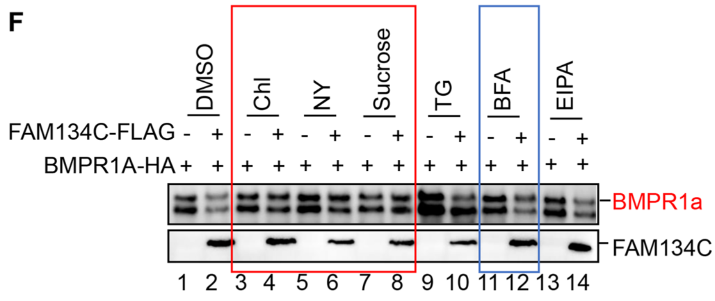

G

Inhibitors	Mode of actions
**Chlorpromazine (Chl)**	Blocks Clathrin-mediated endocytosis pathway.
**Nystatin (NY)**	Blocks Caveolae-mediated endocytosis pathway.
**Sucrose**	Inhibits receptor-mediated endocytosis due to its effects on osmotic conditions and membrane fluidity.
**Brefeldin A (BFA)**	Blocks the transport of secreted proteins and membrane proteins from the endoplasmic reticulum to the Golgi apparatus.
**Ethyl-isopropyl amiloride (EIPA)**	Blocks macropinocytosis.
**Thapsigargin (TG)**	An endoplasmic reticulum (ER) stress inducer.

© The Author(s)

Figure EV2. FAM134C targets membrane-bound BMPR1A for degradation.

(**A**) Schema of the BONCAT translation assay. (**B**, **C**) FAM134C does not degrade newly synthesized BMPR1A. HEK293T cells were co-transfected with FAM134C-FLAG and BMPR1A-HA and/or empty vector control for 24 h. In (**C**), transfected cells were further treated with cycloheximide (10 μM) or HCQ (50 nM) for 8 h. The newly synthesized protein was collected by Streptavidin agarose beads. BMPR1A expression was detected by Western blotting. (**D**) FAM134C overexpression causes BMPR1A degradation. HEK293T cells were co-transfected with FAM134C-FLAG and BMPR1A-HA, and treated with cycloheximide (CHX, 10 mM) for the indicated times. Expression of BMPR1A, FAM134C, and GAPDH were measured by Western blotting. (**E**) FAM134C reduces the levels of BMPR1A on the plasma membrane and endosomes, but not the ER. HCQ blocks BMPR1A degradation in the lysosome. Organelles were collected and analyzed for BMPR1A by Western blotting in HEK293T cells expressing FAM134C-FLAG and BMPR1A-HA. Isolation of the plasma membrane, endosome, ER, and lysosome is described in the Materials and Methods section. Expression of BMPR1A, FAM134C, E-cadherin (membrane marker), EEA1 (endosome marker), PDIA3 (ER marker), and LAMP1 (lysosome marker) were measured by Western blotting. (**F**) FAM134C degrades internalized BMPR1A. HEK293T cells were co-transfected with FAM134C-FLAG and BMPR1A-HA, and treated with Chlorpromazine (Chl, 50 nM), Nystatin (NY, 50 nM), Thapsigargin (TG, 500 mM), or ethyl-isopropyl amiloride (EIPA, 10 mM) for 15 h, sucrose (0.2 M) and Brefeldin A (BFA, 10 mg/ml) for 2 h. Levels of BMPR1A and FAM134C-FLAG were measured by Western Blotting. (**G**) Inhibitors used in (**F**) above. Source data are available online for this figure.

 © The Author(s)

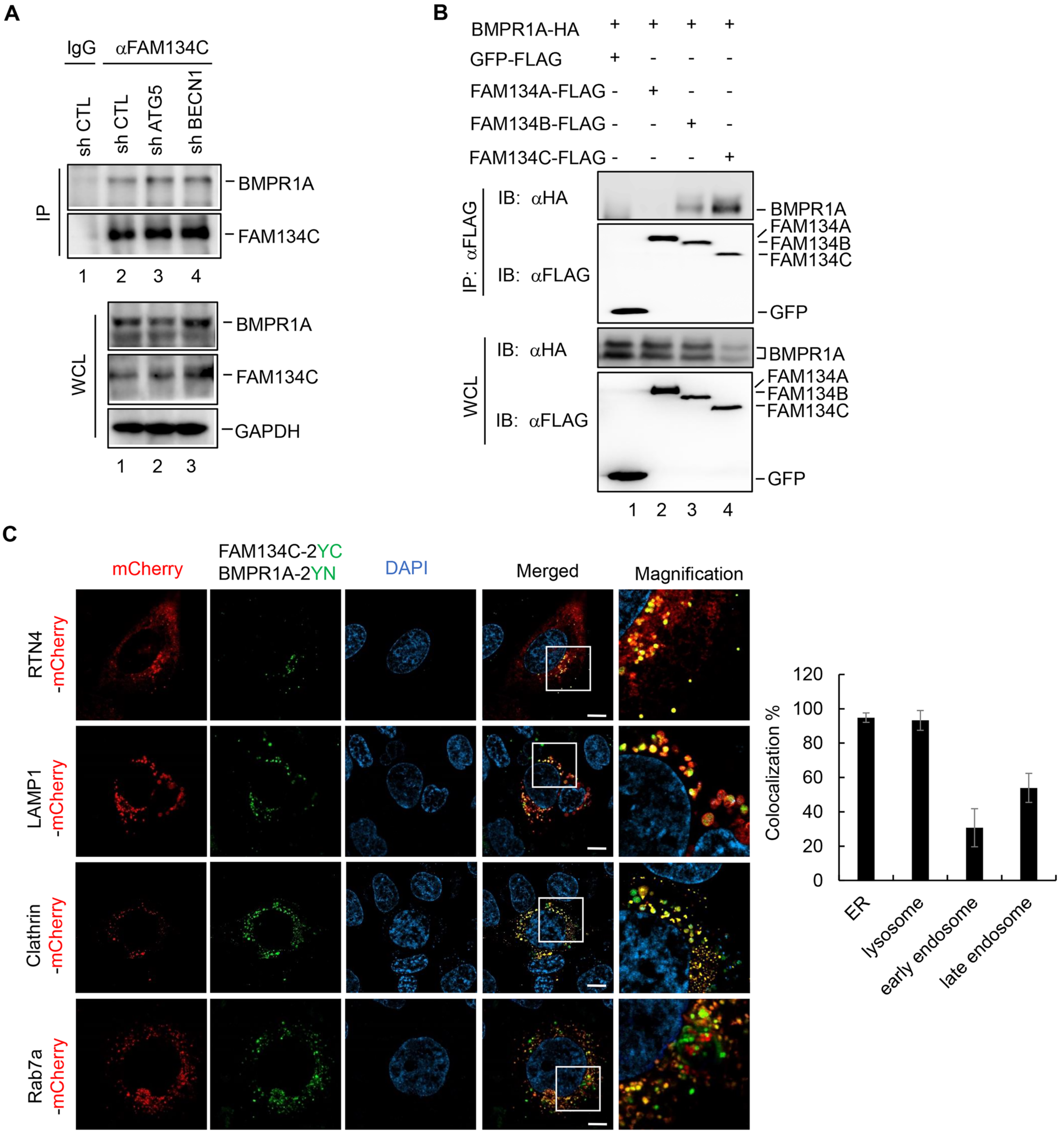

Figure EV3. FAM134C interacts with BMPR1A.

(**A**) ATG5 or BECN1 downregulation had no effect on the interaction between FAM134C and BMPR1A. ATG5 or BECN1 were stably knocked down in U2OS cells, and then FAM134C was immunoprecipitated by anti-FAM134C and anti-BMPR1A antibodies was detected by Western blotting. (**B**) BMPR1A interacts with FAM134C and to a lesser extent FAM134B, but not FAM134A. HEK293T cells were transfected with vectors encoding BMPR1A-HA and FAM134A-FLAG, FAM134B-FLAG, or FAM134C-FLAG, and then FAM134A/B/C were immunoprecipitated by anti-FLAG antibody and FAM134-bound BMPR1A was detected by HA antibody. (**C**) FAM134C is colocalized with BMPR1A. U2OS cells were co-transfected with RTN4-mCherry, LAMP1-mCherry, Clathrin-mCherry, or Rab7a-mCherry together with YC-tagged FAM134C and YN-tagged BMPR1A. Cells were treated with HCQ for 2 h and then analyzed using Zeiss LSM880. The green YFP fluorescence signal indicates the FAM134C-BMPR1A interaction. DAPI (blue fluorescence) was used to stain nuclei (scale bar, 5 µm). The percentage of the FAM134C-BMPR1A interaction colocalized with an organelle was quantified by ImageJ. Bar and error bars show the mean ± SD ($n = 3$ independent experiments; each with three technical replicates). Source data are available online for this figure.

© The Author(s)

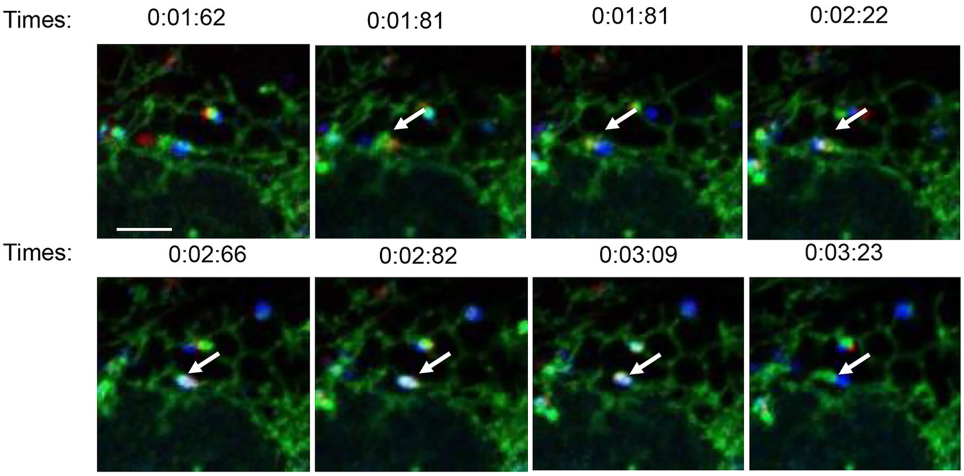

Figure EV4. FAM134C targets BMPR1A into autophagosomes through LC3.

Live cell image shows that FAM134C targets membrane-bound BMPR1A to lysosomes for degradation. FAM134C-KO U2OS cells stably express FAM134C-GFP and BMPR1A-mCherry. Lysotracker (blue fluorescence) was used to stain the lysosome. Cells were analyzed under Zeiss LSM880 (scale bar, 1 µm). Source data are available online for this figure.

 © The Author(s)

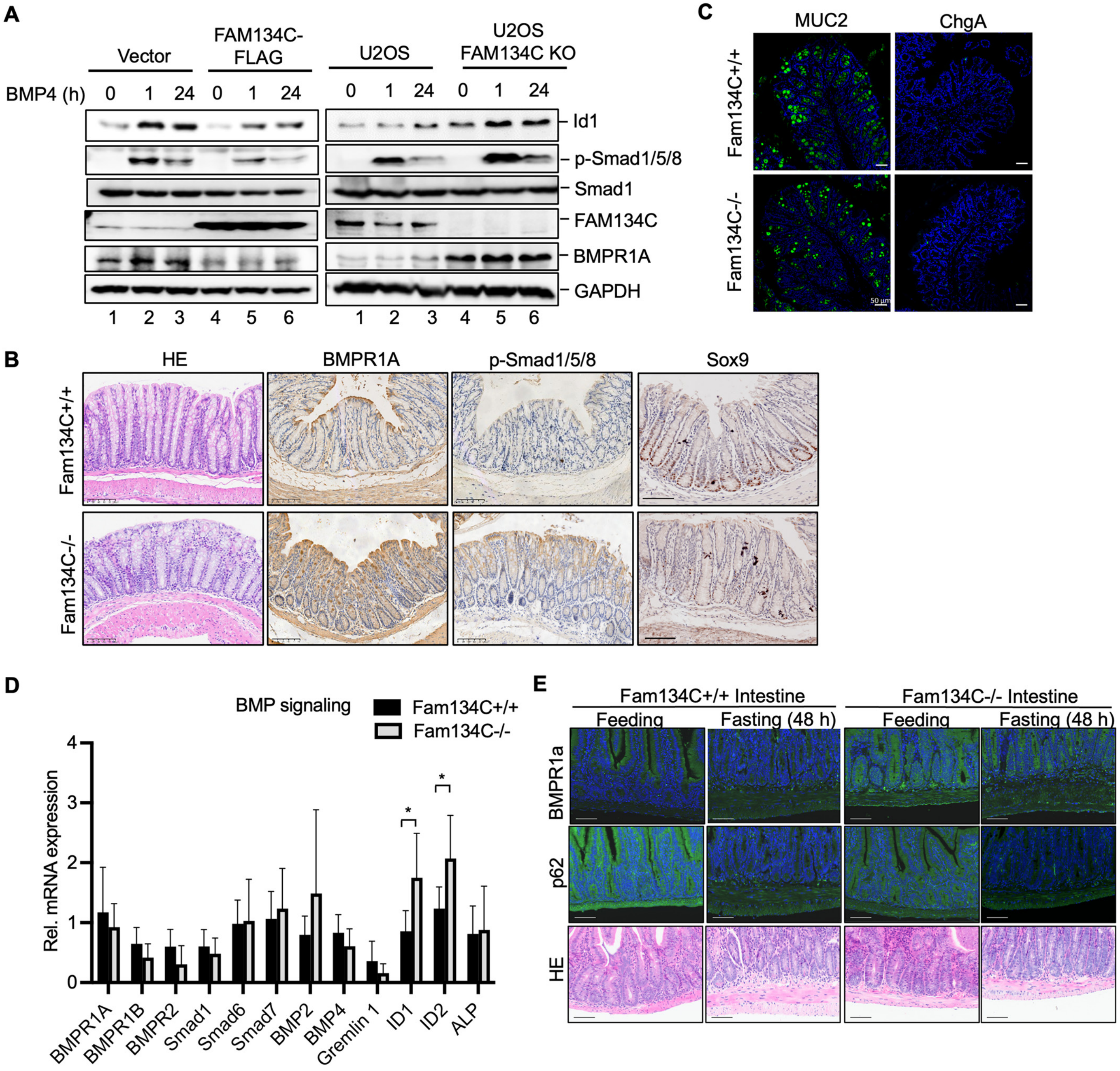

Figure EV5. Lack of FAM134C increases BMP signaling in mouse colons.

(**A**) FAM134C inhibits BMP responses. U2OS cells stably expressing FAM134C-FLAG or with FAM134C knockout or parental cells were treated with BMP (50 ng/ml) for 1 and 24 h. Levels of Id1, p-Smad1/5/8, Smad1, FAM134C, BMPR1A, and GAPDH were measured by Western blotting. (**B**) Hematoxylin-eosin staining and immunohistochemical staining of BMPR1A, p-Smad1/5/8 and Sox9 in colons of WT and FAM134C knockout mice (scale bar, 50 mm). Colon sections were collected at week 8. Images are representative of $n = 6$ mice per genotype. (**C**) Immunohistochemical staining of MUC2 and ChgA in colons of WT and FAM134C knockout mice. Colon sections were collected at week 8 (scale bar, 100 mm). Images are representative of $n = 6$ mice per genotype. (**D**) Quantitative RT-PCR analysis of the indicated genes in the colons of WT and FAM134C knockout mice. Statistical analysis by unpaired two-tailed Student's *t*-test; *$p < 0.05$, **$p < 0.01$, ***$p < 0.001$; mean ± SD ($n = 3$ mice per genotype; each with two technical replicates). Id1 expression, WT vs KO $p = 2.36E-02$; Id2 expression, WT vs KO $p = 2.87E-02$. (**E**) Immunofluorescence staining of BMPR1A and p62, and Hematoxylin-eosin staining in small intestines of WT and FAM134C knockout mice with or without fasting. Proximal jejunum sections were made at week 8. DAPI (blue fluorescence) was used to stain nuclei (scale bar, 100 mm). Source data are available online for this figure.
